# mRNA–miRNA bipartite networks reconstruction in different tissues of bladder cancer based on gene co-expression network analysis

**DOI:** 10.1038/s41598-022-09920-4

**Published:** 2022-04-07

**Authors:** Zahra Abedi, Habib MotieGhader, Sahar Sadat Hosseini, Mohammad Ali Sheikh Beig Goharrizi, Ali Masoudi-Nejad

**Affiliations:** 1 Laboratory of Systems Biology and Bioinformatics (LBB), Institute of Biochemistry and Biophysics, University of Tehran, Tehran, Iran; 2 Department of Biology, Tabriz Branch, Islamic Azad University, Tabriz, Iran; 3 Atherosclerosis Research Center, University of Medical Sciences Tehran, Tehran, Iran

**Keywords:** Cancer genetics, Genetic interaction, Computational biology and bioinformatics

## Abstract

Bladder cancer (BC) is one of the most important cancers worldwide, and if it is diagnosed early, its progression in humans can be prevented and long-term survival will be achieved accordingly. This study aimed to identify novel micro-RNA (miRNA) and gene-based biomarkers for diagnosing BC. The microarray dataset of BC tissues (GSE13507) listed in the GEO database was analyzed for this purpose. The gene expression data from three BC tissues including 165 primary bladder cancer (PBC), 58 normal looking-bladder mucosae surrounding cancer (NBMSC), and 23 recurrent non-muscle invasive tumor tissues (RNIT) were used to reconstruct gene co-expression networks. After preprocessing and normalization, deferentially expressed genes (DEGs) were obtained and used to construct the weighted gene co-expression network (WGCNA). Gene co-expression modules and low-preserved modules were extracted among BC tissues using network clustering. Next, the experimentally validated mRNA-miRNA interaction information were used to reconstruct three mRNA-miRNA bipartite networks. Reactome pathway database and Gene ontology (GO) was subsequently performed for the extracted genes of three bipartite networks and miRNAs, respectively. To further analyze the data, ten hub miRNAs (miRNAs with the highest degree) were selected in each bipartite network to reconstruct three bipartite subnetworks. Finally, the obtained biomarkers were comprehensively investigated and discussed in authentic studies. The obtained results from our study indicated a group of genes including *PPARD*, *CST4, CSNK1E, PTPN14, ETV6,* and *ADRM1* as well as novel miRNAs (e.g., *miR-16-5p, miR-335-5p, miR-124-3p, and let-7b-5p*) which might be potentially associated with BC and could be a potential biomarker. Afterward, three drug-gene interaction networks were reconstructed to explore candidate drugs for the treatment of BC. The hub miRNAs in the mRNA-miRNA bipartite network played a fundamental role in BC progression; however, these findings need further investigation.

## Introduction

Bladder cancer (BC) is the second most common cancer originating from the epithelium of the urinary bladder with a high rate of morbidity, mortality, and metastasis^1^. To perform cancer therapy, early diagnosis is extremely necessary since cancer mortality can be significantly reduced by early detection. Thus, many studies have been conducted to discover new biomarkers^2^. Moreover, the miRNA expression level is considered as a diagnostic biomarker for different cancers^3^. In addition, developing gene biomarkers contributes to finding novel therapeutic targets and prognosis of BC^4^. Nowadays, various cancer biomarkers such as DNA, RNA, protein- and epigenetic- based biomarkers are being used to diagnose cancers^5^. The RNA-based biomarkers including coding and non-coding RNA expression levels are more important and play significant roles in many biological processes^6^. One of the most useful types of such biomarkers is miRNA, which includes a group of 22 nucleotide non-coding RNAs involved in post-transcriptional regulation of gene expression^7^. As for their role, miRNAs act as tumor suppressors or oncogenes that modulate various tumor-suppressive/oncogenic pathways^8^. Differentially expression of these RNAs was further observed in cancerous samples than in normal ones or in one stage compared with another in many cancers^9^. The strong relationship between miRNAs and cancers indicates the potential application of miRNAs in the diagnosis and prognosis of cancers. This relationship is clearly proved in the literature^10^. For example, Adam et al. suggest that miR-200-family miRNAs (e.g., *miR-141*, *miR-141a*, *miR-429*, *miR-192*, and the like) are promising as non-invasive, diagnostic, and prognostic markers^11^. Taheri et al. have reported using miR-26b-5p as a prognostic biomarker for the recurrence and progression of BC^12^. Moreover, this miRNA demonstrated the highest association rate with progression and recurrence of BC^13^.

This study constructs the gene co-expression network and identifies modules and gene–gene correlations. The WGCNA R package has been widely used in various studies to reveal the correlation between genes and indicate eigen genes and intra-modular hub genes. In addition, it helps estimate measurement values related to topological properties and module membership^14^. Using miRNA and gene expression data, one study employed WGCNA to find diagnostic biomarkers for colorectal cancer (CRC) using miRNA and gene expression data^3^. To this end, MotieGhader et al. constructed co-expression networks for different stages of CRC and extracted low-preserved modules. Further, MotieGhader et al. found two novel miRNA biomarkers via studying gene-miRNA interactions and constructing bipartite networks^3^. Another study performed network construction by applying WGCNA on differentially expressed genes ^15^. The results indicated that this approach facilitates calculation and helps exclude those genes demonstrating a low expression level while representing a high correlation rate.

Many studies have been carried out on BC and introduced some proposed BC biomarkers. However, BC is a common cancer whose critical biological pathways and the involved functional genes have not been fully discovered. The present study explored interactions between genes and their target miRNAs through bipartite networks and identified genes and miRNA biomarkers in different BC samples. Furthermore, investigation of the related pathways could enhance our understanding of BC development. Finally, three drug-gene interaction networks were reconstructed using the Drug Gene Interaction Database (DGIdb) to discover candidate drugs for inhibiting the target genes of hub miRNAs. The results may be helpful for future research studies on the mechanism and treatment of BC.

## Result

### Module analysis

Three gene co-expression networks were reconstructed through WGCNA on 5,563 DEGs delineating 16, 29, and 16 modules in the NBMSC- PBC, PBC- RNIT, and NBMSC-RNIT networks, respectively. In the NBMSC-PBC, the smallest module (lightcyan) consisted of 72 genes, while the largest module (turquoise) comprised 1421 genes. The PBC-RNIT network contained saddle brown and blue modules which were the smallest and largest modules, respectively. The saddle brown and blue modules consisted of 46 and 596 genes, respectively. Furthermore, the smallest and the largest modules in NBMSC-RNIT were lightcyan (with 72 genes) and turquoise (with 1421 genes), respectively. The grey module in the NBMSC-PBC, PBC-RNIT, and NBMSC-RNIT networks incorporated 4, 96, and 4 genes, respectively, which were excluded from further analysis.

### Comparison of the modules between NBMSC, PBC, and RNIT tissues

The altered modules may affect many signaling cascades and lead to disease between BC tissues. Hence, it seems the modules that did not demonstrate inter-tissue preservation were involved in the progression of BC. The Z_summary_ values were calculated for all modules in the NBMSC co-expression network, compared with the PBC expression data, as well as the RNIT co-expression network relative to the PBC expression data and the NBMSC co-expression network compared to RNIT expression data. Consequently, 5, 4, and 5 were determined as thresholds for NBMSC-PBC, PBC-RNIT, and NBMSC-RNIT module groups, respectively (Supplementary Fig. S1). Modules with a Z_summary_ ranging from 7.20 to 9.10 were selected as significant moderated preservation modules (NBMSC-PBC modules), while they ranged from 2.6 to 4.1 for PBC-RNIT modules and from 2.3 to 5.50 for NBMSC-RNIT modules. More precisely, these modules exhibited low preservation and could be useful in the progression of BC. Finally, the signed-hybrid network was set as the type of network. Supplementary Fig. S2 illustrates the preservation of median rank and Z_summary_ along with the module size.

Three modules of NBMSC were selected in comparison with the PBC expression data (NBMSC-PBC modules). Z_summary_ values of these modules were equal to or less than 9.10. Three modules of the NBMSC, compared with RNIT, were chosen with Z_summary_ values equal to or smaller than 5.50 compared with the RNIT expression data (NBMSC-RNIT modules). Furthermore, three modules of the PBC stage with Z_summary_ values equal to and smaller than 4.1 were selected compared with the RNIT expression data (PBC-RNIT modules). The selected modules are illustrated by their attributes in Table 1.

**Table 1:** Extracted modules and their properties at three stages.

	Number of modules	Value of Z_summary_	Module color	Number of genes in module
**NBMSC-PBC**				
1	7	7.20	Lightcyan	72
2	11	9.10	Purple	187
3	12	7.40	Red	277
**PBC-RNIT**				
1	17	3.1	Midnightblue	112
2	18	2.6	Orange	65
3	20	4.1	purple	187
**NBMSC-RNIT**				
1	7	2.30	Lightcyan	72
2	11	5.30	Purple	187
3	12	5.50	Red	277

As a result, lightcyan, purple, and red modules manifested low preservation between NBMSC and PBC tissues (NBMSC-PBC modules). In contrast, the midnight blue, orange, and purple modules exhibited low preservation between PBC and RNIT tissues (PBC–RNIT modules). Likewise, the lightcyan, purple, and red modules exhibited low preservation between NBMSC and RNIT tissues. Table 1 summarizes all detected modules and their Z_summary_ in detail.

### Enrichment analysis of the gene modules

Functional enrichment analysis was run to investigate the biological functions of genes in three sets of significant low preservation modules (i.e., NBMSC-PBC, RNIT-PBC, and NBMSC-RNIT modules).

Based on GO enrichment analysis, the low preservation modules (generated by WGCNA) were enriched in different GO terms. The GO results for NBMSC- PBC modules indicated that the genes in the lightcyan module were enriched in the *apoptotic process*, *skeletal muscle cell differentiation*, *positive regulation of transcription from RNA polymerase II promoter*, *positive regulation of transcription*, and *DNA template*. All other significant GO terms are reported in Supplementary Table S1. The GO terms with the lowest *p*-value (less than 0.01) related to the purple module included *translation*, *transcription*, *DNA template*, *regulation of transcription*, and *cellular response to UV*. In addition, the genes in the red module were more enriched in the *oxidation–reduction process* and *negative regulation of osteoblast differentiation*. Additionally, other significant GO terms for this module are reported in Supplementary Tables S2 and S3.

The enrichment analysis for PBC-RNIT modules indicated that the midnight blue module was highly enriched in *negative regulation of neuron death, dicarboxylic acid transport, phosphorylation, skeletal muscle tissue development, cell differentiation*, and *somatic cell population maintenance* (Supplementary Table S4). GO enrichment for orange and purple modules revealed no significant terms with a *p*-value of less than 0.05 (Supplementary Tables S5 and S6).

Similarly, GO enrichment analysis for NBMSC-RNIT modules was performed. The results demonstrated that the lightcyan module was enriched in the *apoptotic process, skeletal muscle cell differentiation, positive regulation of transcription, DNA template*, and many significant terms provided in Supplementary Table S7.

GO enrichment results for purple and red modules were similar to purple and red modules of NBMSC-PBC as illustrated in Supplementary Tables S8 and S9.

To investigate significant biological pathways for each module in NBMSC-PBC, pathway enrichment analysis was used based on the Reactome pathway database. The results revealed that the most significant pathways related to the lightcyan module were the *signaling by interleukins*, *cytokine signaling in immune* system, *NGF-stimulated transcription*, *signal transduction*, *nuclear events (kinase and transcription factor activation)*, *Toll like receptor 10 (TLR10) cascade*, *Toll like receptor 5 (TLR5) cascade*, *myD88 cascade initiated on plasma membrane*, *TRAF6 mediated induction of NFkB and MAP kinases upon TLR7/8 or 9 activation*, *MyD88 dependent cascade initiated on endosome*, *Toll like receptor 7/8 (TLR7/8) cascade*, *Toll like receptor 9 (TLR9) cascade*, *Toll like receptor TLR6:TLR2 cascade*, *MyD88:MAL(TIRAP) cascade initiated on plasma membrane*, *interleukin-10 signaling*, *Toll like receptor TLR1:TLR2 cascade*, *Toll like receptor 2 (TLR2) cascade*, *RNA polymerase II transcription*, *interleukin-4 and interleukin-13 signaling*, *generic transcription pathway*, *signaling by NTRK1 (TRKA)*, MAPK *activation*, *signaling by NTRKs*, *Toll like receptor 4 (TLR4) cascade*, *gene expression (transcription)*, *circadian clock*, *interleukin-1 family signaling*, *interleukin-17 signaling*, *RAF-independent MAPK1/3 activation*, *immune system*, and *Toll-like receptor cascade* (Supplementary Table S10). The genes in the purple module were more enriched in *gene expression (transcription)*, *RNA polymerase II transcription*, *generic transcription pathway*, *metabolism of RNA*, *axon guidance*, and *nervous system development* with a *p*-value of less than 0.01 (Supplementary Table S11) while the genes in the red module were more enriched in *metabolic pathways*. More details can be found in Supplementary Table S12. It should be noted that, based on the analyses, the significant pathways were identified for the midnight blue, orange, and purple modules (PBC-RNIT modules) included *diseases of metabolism*, *ligand-receptor interactions*, and *neddylation* with a *p*-value less than 0.01, respectively (Supplementary Tables S13, S14 and S15)*.* Additionally, Reactome pathway analysis for NBMSC-RNIT modules was performed. The results illustrated that the most significant pathways related to the lightcyan and purple modules were similar to the lightcyan and purple modules in NBMSC-RNIT (Supplementary Tables S16 and S17). It should be noted that, based on the analyses, no significant pathways were identified for the red module (NBMSC-RNIT modules).

### Gene-miRNA bipartite network and hub miRNAs

**Figure 1:**
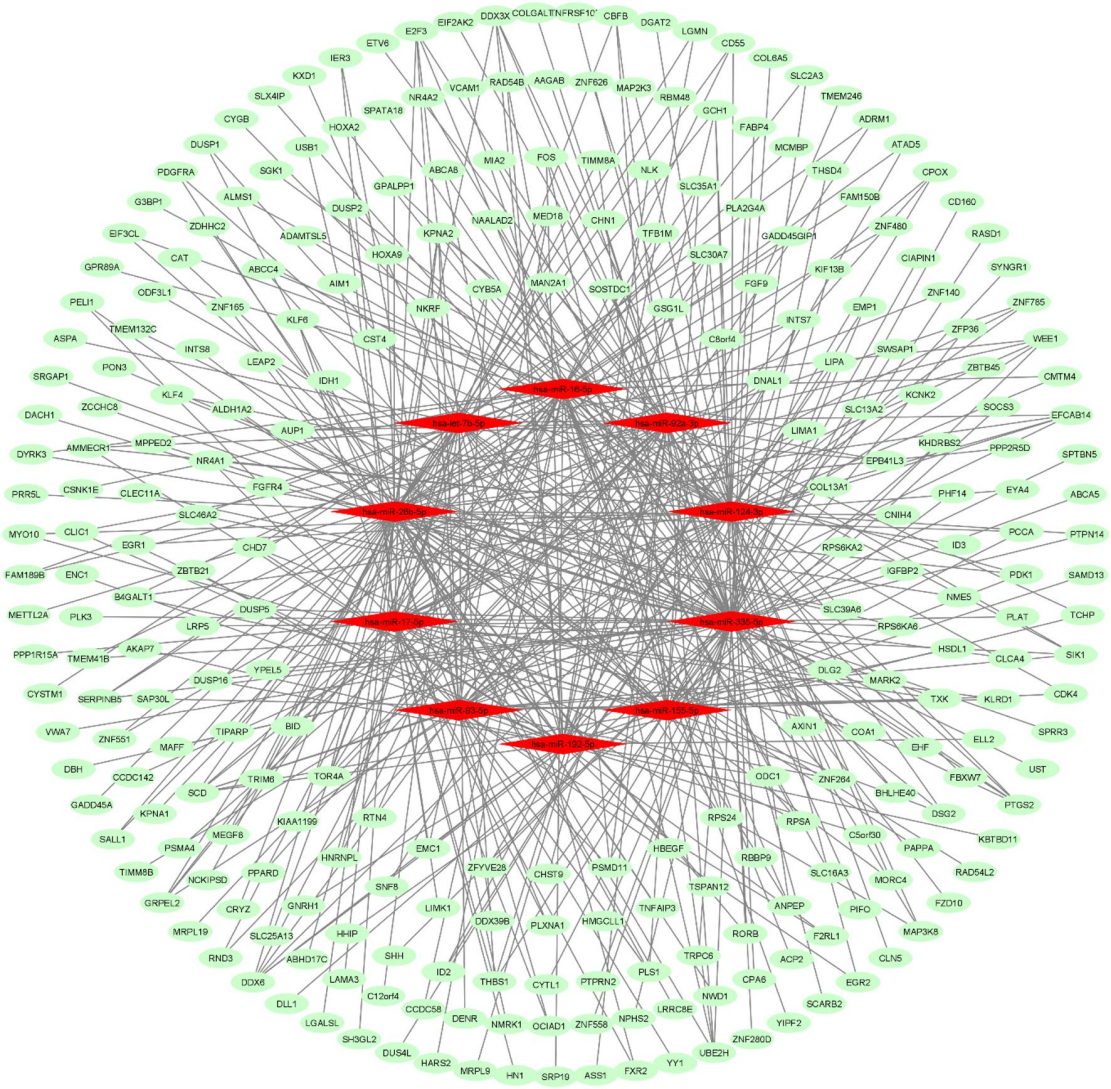
Bipartite mRNA-miRNA subnetwork for NBMSC-PBC. Cytoscape v.3.8.2 was used to visualize the network.

**Figure 2:**
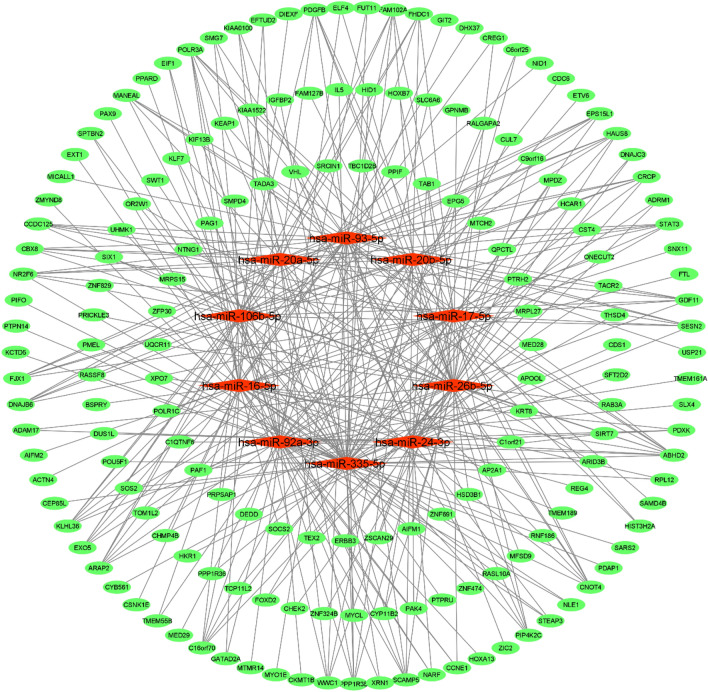
Bipartite mRNA-miRNA subnetwork for PBC-RNIT. Cytoscape v.3.8.2 was used to visualize the network.

**Figure 3:**
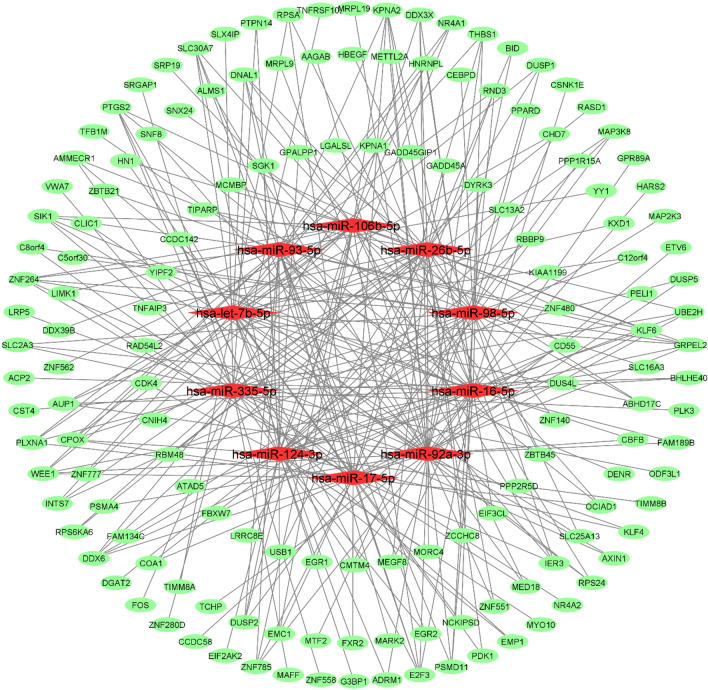
Bipartite mRNA-miRNA subnetwork for NBMSC-RNIT. Cytoscape v.3.8.2 was used to visualize the network.

Three mRNA-miRNA bipartite networks (i.e., NBMSC-PBC, PBC-RNIT, and NBMSC-RNIT) were reconstructed using significant modules and their related miRNAs. High degree miRNAs in these networks modulated a more significant number of genes, exerting a remarkable impact on posttranscriptional regulation. Next, the top 10 miRNAs (hubs) were extracted from each network and used for further analysis along with their target genes. It is important to select only high degree miRNAs to avoid complexity and exclude miRNAs with trivial effects on the networks and pathogenesis. Subnetworks are illustrated in Figs. 1, 2 and 3. The NBMSC-PBC, PBC-RNIT, and NBMSC-RNIT subnetworks contained 273, 171, and 149 genes, respectively.

**Figure 4:**
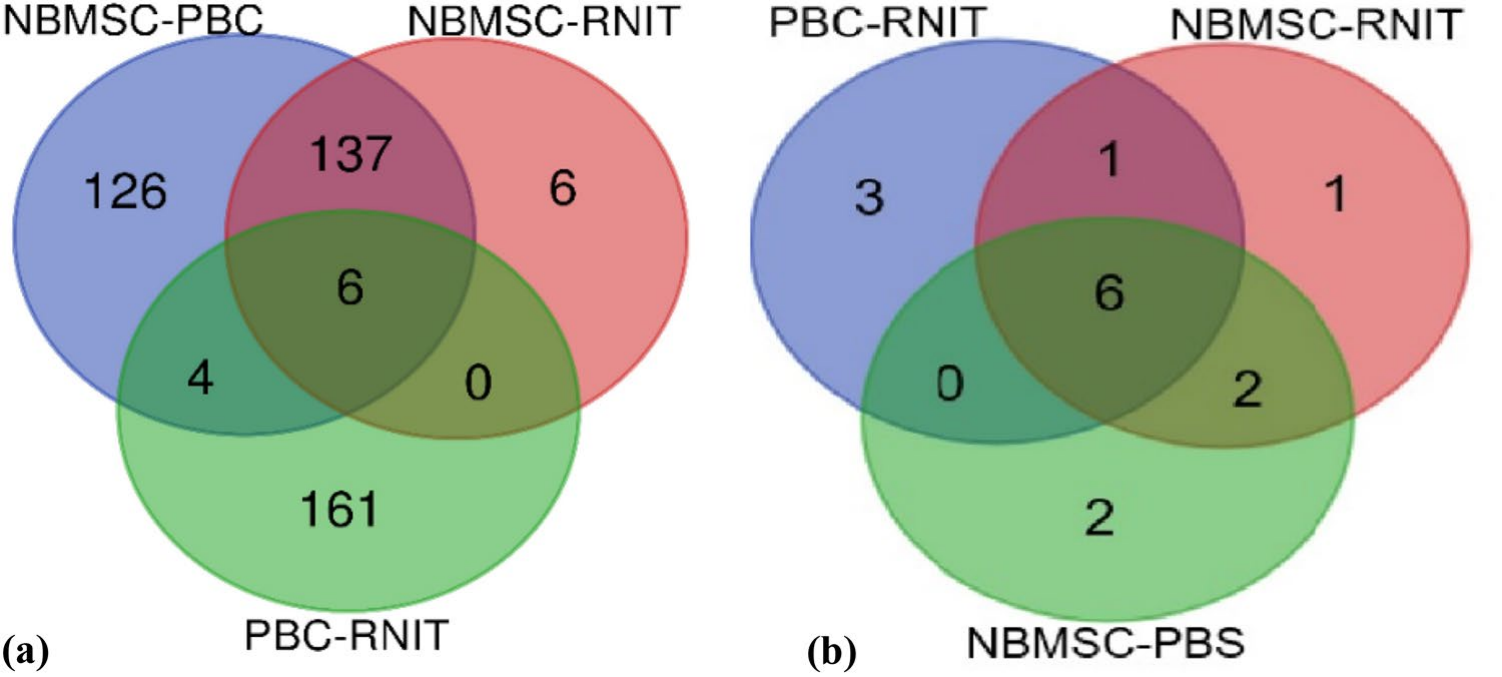
Venn diagram for the obtained genes and miRNAs. (**a**) Venn diagram of genes (**b**) Venn diagram of miRNAs. The webtool (https://bioinformatics.psb.ugent.be/webtools/Venn) was used to construct Venn diagram.

To show the connections between subnetworks, Venn diagrams were used to illustrate the individual subnetworks through genes and previously extracted miRNAs (Fig. 4). The list of genes and miRNAs are shown in Supplementary Tables S18 and S19, respectively.

### Enrichment analysis of mRNAs in the bipartite subnetwork

The GO enrichment analysis results demonstrated that the genes in the NBMSC- PBC subnetwork were more significantly enriched in response to *estradiol*, *negative regulation of osteoblast differentiation*, *peptidyl-tyrosine dephosphorylation*, *regulation of axonogenesis*, *the establishment of cell polarity*, *MAPK cascade*, *inactivation of MAPK activity*, *cytokine production*, and 7 GO terms with a *p*-value of less than 0.01 (Supplementary Table S20). In the same way, the mRNAs in the PBC–RNIT subnetwork were more significantly enriched in *positive regulation of transcription*, *DNA template*, *transcription*, and *cellular response to hormone stimulus* (Supplementary Table S21). Finally, the GO enrichment analysis of the NBMSC–RNIT subnetwork led us to the finding that the genes in this subnetwork were more enriched in such terms as *fat cell differentiation*, *skeletal muscle cell differentiation*, *protein phosphorylation*, *translation*, and other 6 GO terms with *p*-values smaller than 0.01 (Supplementary Table S22). Further, the Reactome pathway database was used for the pathway enrichment analysis of the genes in each bipartite subnetwork. Based on this analysis, the genes in the NBMSC–PBC subnetwork were significantly involved in the *nuclear Events (kinase and transcription factor activation)*, *signal transduction*, *NGF-stimulated transcription*, *signaling by NTRK1 (TRKA)*, *RAF/MAP kinase cascade*, *MAPK1/MAPK3 signaling, signaling by NTRKs*, *MAPK family signaling cascades*, *negative regulation of MAPK pathway*, and *RAF-independent MAPK1/3 activation* (Supplementary Table S23). The NBMSC-RNIT subnetwork was significantly related to the *RNA polymerase II transcription*, *generic transcription pathway*, *gene expression (transcription)*, *nuclear events (kinase and transcription factor activation)*, *signal transduction*, *NGF-stimulated transcription*, *negative regulation of MAPK pathway*, *transcriptional regulation of white adipocyte differentiation*, *MyD88 cascade initiated on plasma membrane*, *Toll like receptor 10 (TLR10) cascade*, *Toll like receptor 5 (TLR5) cascade*, *TRAF6 mediated induction of NFkB and MAP kinases upon TLR7/8 or 9 activation*, *MyD88 dependent cascade initiated on endosome*, *Toll like receptor 7/8 (TLR7/8) cascade*, and *cytokine signaling in immune system* (Supplementary Table S24). It should be noted that based on our analyses, no significant pathways were observed for the PBC-RNIT subnetwork with a *p*-value of less than 0.01.

### Enrichment analysis of miRNAs in the bipartite subnetworks

To annotate hub miRNAs, the TAM tool, which benefits from miRbase and HMDD databases, was applied for enrichment analysis. First, the genes regulated by the miRNAs of interest were selected. The miRNAs found in NBMSC-PBC subnetwork were almost from the miR-124, miR-25, miR-15, and miR-17 family with *p*-values of 3.90e-7 and 1.57e-3, respectively (Supplementary Table S25). The most important functions in this subnetwork were *cell proliferation*, *cell cycle*, *cell division*, *hormone-mediated signaling pathway*, *regulation of stem cell*, *bone regeneration*, *cell differentiation*, *latent virus replication*, *immune system*, along with other functions provided in Supplementary Table S26. The second subnetwork (NBMSC-RNIT) was almost from the miR-124, miR-17, miR-25, miR-15, and miR-7 family, and their *p*-values were 3.90e-7 and 1.57e-3, respectively (Supplementary Table S27). The important functions related to this subnetwork included *cell division*, *cell proliferation*, *cell cycle*, *hormone-mediated signaling pathway*, *tumor suppressor miRNAs*, and some other functions (Supplementary Table S28). Finally, the third subnetwork (PBC-RNIT) was almost from the miR-17, miR- 24, miR-25, and miR-15 family family with *p*-values of 4.85e-10 and 4.88e-4, respectively (Supplementary Table S29). The important functions of this subnetwork were *onco-miRNAs*, *bone regeneration*, *latent virus replication*, *hormone-mediated signaling pathway*, *apoptosis*, and *cell death*. Additionally, other significant GO terms for this subnetwork are reported in Supplementary Table S30.

### Identification of candidate drugs

**Figure 5:**
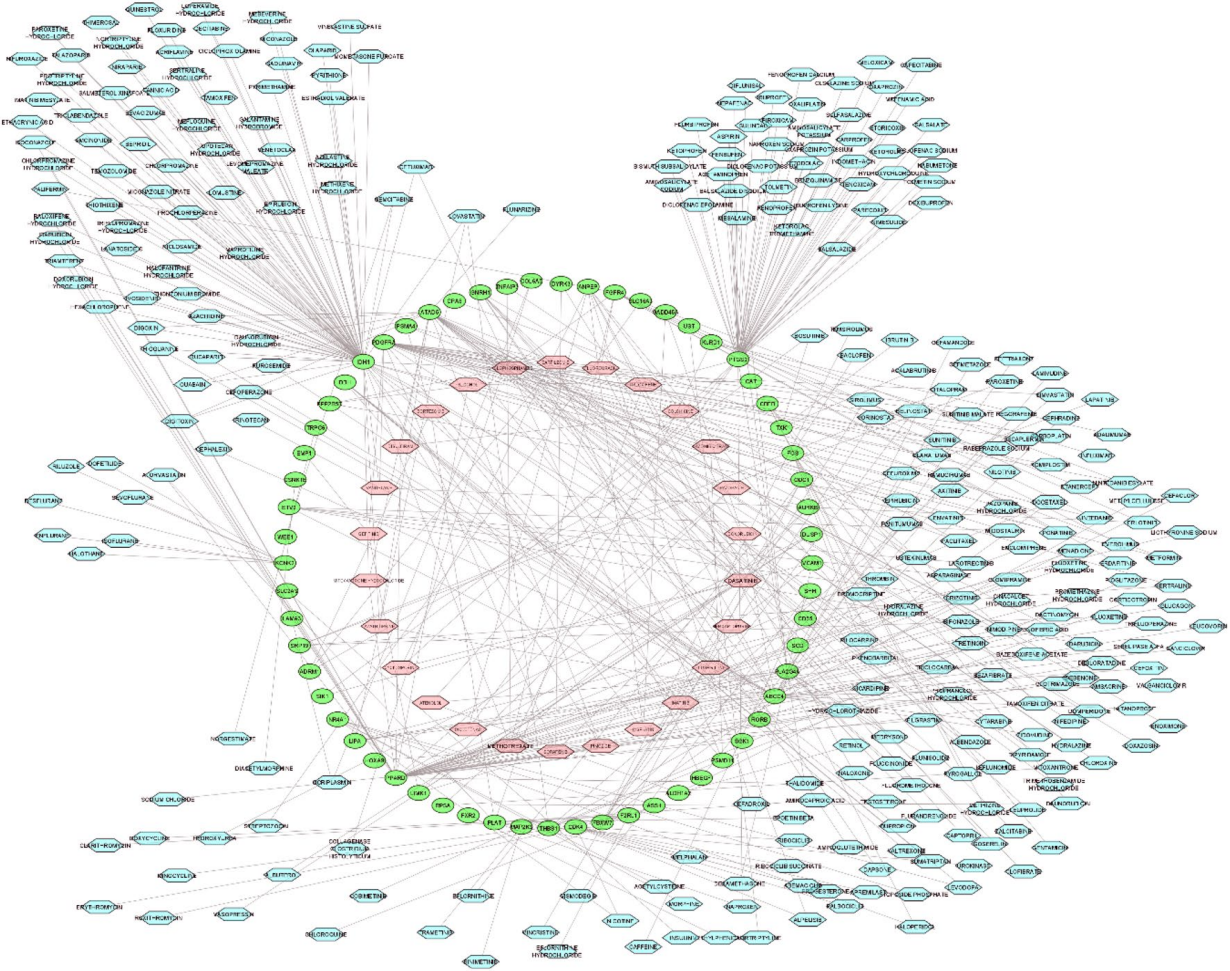
The candidate drug-gene network extracted from DGIdb database. The candidate drugs (lightblue hexagon) identified as regulators of the NBMSC-PBC subnetwork. Among these drugs, some drugs can regulate more than two gene (red hexagon). Cytoscape v.3.8.2 was used to visualize the network.

**Figure 6:**
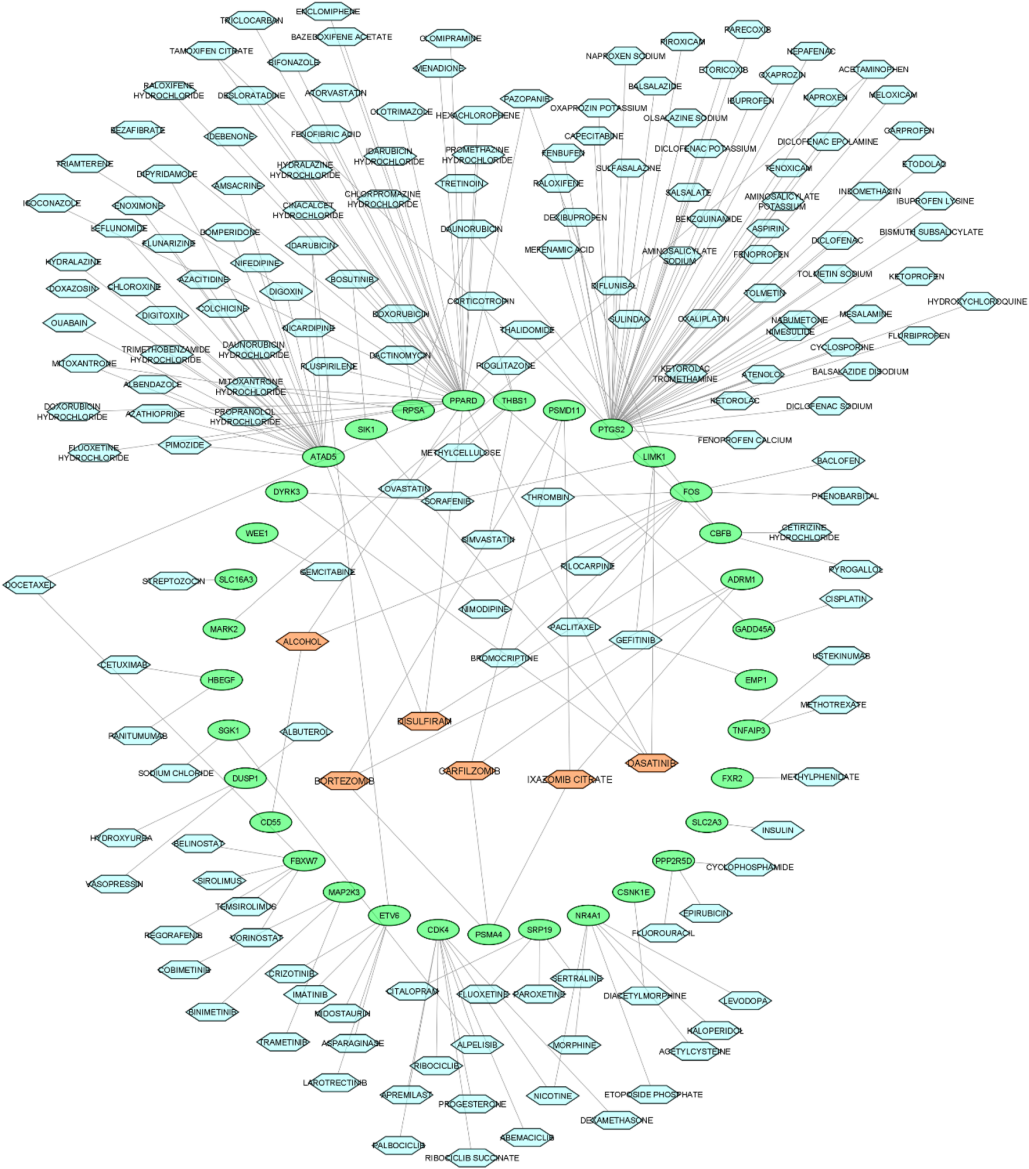
The candidate drug-gene network extracted from DGIdb database. The candidate drugs (lightblue hexagon) identified as regulators of the NBMSC-RNIT subnetwork. Among these drugs, some drugs can regulate more than two gene (red hexagon). Cytoscape v.3.8.2 was used to visualize the network.

**Figure 7:**
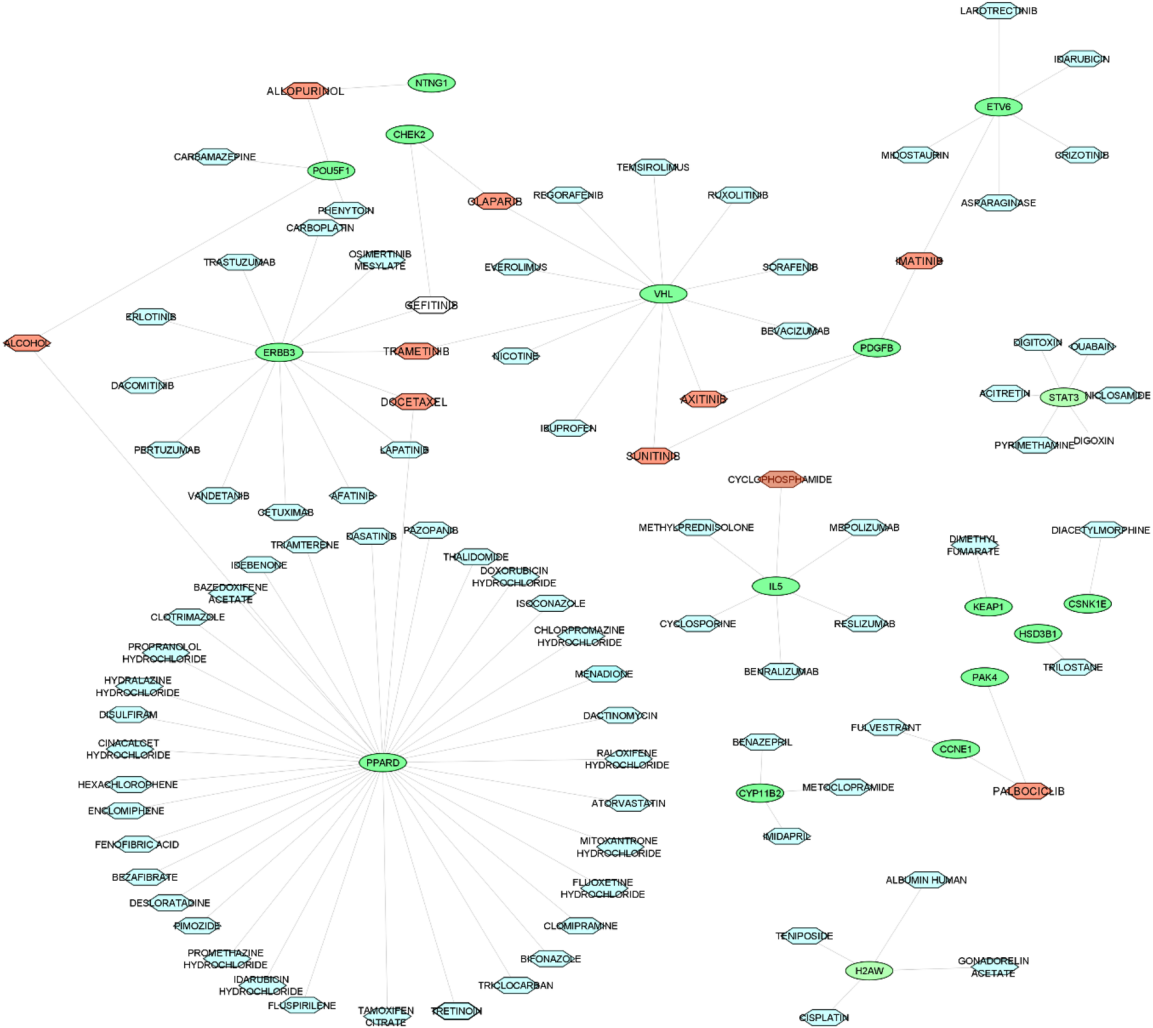
The candidate drug-gene network extracted from DGIdb database. The candidate drugs (lightblue hexagon) identified as regulators of the PBC-RNIT subnetwork. Among these drugs, some drugs can regulate more than two gene (red hexagon). Cytoscape v.3.8.2 was used to visualize the network.

The DGIdb was used to identify drugs that target the genes. In this regard, first, the genes were imported into DGIdb, and then drug-gene interactions were gathered for the genes by limiting drugs to approved drugs. After obtaining drug-gene interactions for all genes in the networks, the drug-gene interactions were gathered and reconstructed for three drug-gene networks. Through the using of this database, many drugs were identified for the genes in three mRNA-miRNA bipartite networks. Then, the Cytoscape (Version 3.8.2) was utilized to visualize these data through the drug-gene networks. In the drug-gene network for NBMSC-PBC, some genes had more than one drug. In addition, some drugs regulated several genes; therefore, these drugs turned out to be more noticeable in the drug-gene network. The drugs such as *dasatinib*, *methotrexate*, *sorafenib*, *pazopanib*, *mercaptopurine*, *disulfiram*, *cisplatin* and *bortezomib* exhibited several interactions with the genes associated with BC (Fig. 5). In addition, in the drug-gene network for NBMSC-RNIT, the important drugs that had more targets were *dasatinib*, *ixazomib citrate*, *carfilzomib*, *bortezomib*, and *disulfiram* (Fig. 6). Finally, the important drugs in the drug-gene network for PBC-RNIT were *sunitinib*, *imatinib*, *axitinib*, *palbociclib*, *allopurinol*, *gefitinib*, *olaparib*, *docetaxel*, and *trametinib* (Fig. 7). All these drugs had two targets, and they can be repurposed for treating BC. The *dastatinib* influences was examined in patients with muscle-invasive urothelial carcinoma of the bladder^16^. The *methotrexate* effects was described in the treatment of patients with advanced urinary BC^17^. Inhibitory actions of *sorafenib* have been confirmed in different BC cell lines^18^. Also, a combination of ritonavir and *ixazomib* inhibited the growth of BC cells^19^. The effect of *sunitinib* was investigated in chemotherapy-resistant BC patients^20^. Activities of these drugs are further elaborated in the discussion section.

## Discussion

In this study, the effect of mRNAs and miRNAs expression in three tissues of BC were investigated to discover biomarkers. Co-expression network analyses were performed based on gene expressions. The experiments were carried out on 165 PBC samples, 58 NBMSC samples, and 24 RNIT samples. First, differentially expressed significant mRNAs (adjusted *p*-value < 0.01) were selected for this purpose. Accordingly, 5563 genes were used to construct the co-expression network. Next, three co-expression networks from NBMSC, PBC, and RNIT samples related to mRNA expression data were reconstructed using WGCNA package.

First, the networks were reconstructed, and then the obtained modules were identified and merged. Some modules are detected by clustering methods whose expression profiles are very identical. More particularly, a module identification method may detect the modules that eigen genes are highly correlated. Since it is difficult to distinct highly correlated modules, it is recommended to merge them^21^. Next, bipartite networks were established using genes and their interacting miRNAs. Hub miRNAs with a fundamental role in the modulation of genes were pinpointed and used to create subnetworks. Afterward, Venn diagrams were constructed for the extracted genes and miRNAs in each subnetwork. The above-mentioned listed genes and miRNAs were the proposed biomarkers of BC and were obtained using our proposed method. We examined the obtained biomarkers meticulously. In the first step, we explored the list of genes that are targeting the miRNAs identified in the previous step. The first group involved six genes (e.g., *PPARD*, *CST4*, *CSNK1E*, *PTPN14*, *ETV6*, and *ADRM1*) that were obtained as a result of the intersection of three module groups. *PPARD* was cloned from epithelial BC cells^22^. Previous studies have reported that cystatins (*CST4*) were involved in tumor invasion and metastasis^23^. *CSNK1E* was overexpressed in several cancer tissue samples and compared to non-tumorigenic normal tissue, suggesting a positive role of *CSNK1E* in neogenesis or maintenance^24^. *PTPN14* (the non-receptor tyrosine phosphatase type 14), also known as *Pez*, *PTP36*, or *PTPD2*, was involved in controlling metastasis^25^. It regulated cell–cell and cell–matrix adhesion, cell migration, and growth^26^. The other gene was *ETV6* (E-Twenty-Six variant gene 6) which is a strong transcriptional repressor associated with the development and progression of tumors^27^. *ADRM1* is consistently overexpressed in BC^28^. Numerous studies suggested that this gene may be a potential target in many tumor types. Recently, knockdown of *ADRM1* has resulted in suppressing proliferation and inhibiting CRC cell migration, survival, and tumorigenicity^29,30^.

The next group consisted of the genes associated with the overlap between NBMSC- PBC module groups and PBC-RNIT module groups (e.g., *KIF13B*, *IGFBP2*, *THSD4*, and *PIFO*). *KIF13B* belongs to the kinesin family (*KIFs*), acting as intracellular transportation or cell division. Some kinesins are involved in mitosis; hence, they have emerged as potential targets for cancer drug development. *KIF13B* is involved in SHh signaling and may be a future target in cancer research^31^. This gene is mainly expressed in endothelial cells and is involved in angiogenesis. The reduction of *KIF13B* had a considerable impact on endothelial cell migration, and its absence exerted a negative effect on the vessels. Re-expression of this gene positively affected angiogenesis in endothelial cells, suggesting the importance of this gene in this process^32^. *IGFBP2* plays a role in cancer progression. Some reports indicated a tumor-suppressive role, and others demonstrated that *IGFBP2* may act as an oncogene^33^. The next gene, *THSD4*, seems to be involved in tumorigenesis and the development of various cancers, including breast cancer, glioblastoma, and esophageal carcinoma^34^. *PIFO* is an important gene involved in ciliary functions. The primary cilium plays a significant role in numerous types of cancer; accordingly, genes and proteins related to the primary cilium's function and structure are considered therapeutic targets. As many of these proteins are related to mitosis, their dysregulation might result in tumorigenesis.

There existed 137 genes that belonged to the overlap between NBMSC-PBC module groups and NBMSC-RNIT module groups. In this step, ten genes with larger degrees were selected from among the 137 genes including *E2F3*, *GRPEL2*, *KPNA2*, *ZNF264*, *CPOX*, *DNAL1*, *PTGS2*, *SIK1*, *SLC30A7*, and *WEE1*. One study revealed that *E2F3* was associated with bladder tumorigenesis. The expression of *E2F3* in BC was demonstrated at the RNA and protein levels^35^, and it was linked with tumorigenesis in this cancer^36^. Since enhanced levels of *E2F3* were associated with a higher tumor stage and grade, this gene was regarded as an oncogene in BC^37^. The lack of the *E2F3* gene led to the inhibition of BC cells, while its overexpression was linked with remarkable growth and proliferation of prostate and BC, which is in line with its oncogenic function^38,39^. High *KPNA2* expression was reported in several cancers such as melanoma^40^, cervical cancer^41^, esophageal cancer^42^, lung cancer^43^, ovarian cancer^44^, prostate cancer^45^, liver cancer^46^, and BC^47^. Upregulation of *PTGS2* was observed in various cancers^48^. Inhibiting efflux pump for chemotherapeutic drugs (e.g., CDDP and docetaxel) occurred by *PTGS2*^49^. Another study indicated that *PTGS2* was involved in BC development and invasion and was associated with angiogenesis^50^. One study for the first time reported that *SIK1* was involved in BC progression^51^. *SIK1* is a tumor suppressor in many cancers^52^. Six genes in this group (i.e., *GRPEL2*, *ZNF264*, *CPOX*, *DANL1*, *SLC30A7*, and *WEE1*) have not been found in clinical research studies.

In the following section, target miRNAs are investigated based on clinical research studies of BC. The first group involves six miRNAs that were obtained based on the intersection of three module groups. These miRNAs included *hsa-miR-93-5p*, *hsa-miR-16-5p*, *hsa-miR-92a-3p*, *hsa-miR-26b-5p*, *hsa-miR-335-5p*, and *hsa-miR-17-5p*.

*hsa-miR-93-5p* was reported to play an important role in the chemosensitivity in BC according to the studies published in 2016 and 2018^53^. In most human cancers (e.g., bladder carcinoma, esophageal carcinoma, lung cancer, and breast cancer), *miR‐93‐5p* was overexpressed. Indeed, *miR‐93‐5p* acted as an oncogene^54^. The functionality of *miR-16-5p* has not been discovered in BC. In one study, the researchers reported that *miR-16-5p* expression was found to be conspicuously attenuated in BC cells^55^. Previous studies evidenced that miR-16-5p is a tumor suppresser in such cancers as chordoma, hepatocellular carcinoma, and colorectal^56–58^. Also *miR-92a-3p* was reported in urinary cells which independently predicted tumor progression in non-muscle invasive BC^59^.

*hsa-miR-26b-5p* was highlighted as a tumor suppressor in different cancers. Downregulating of miR-26b-5p has been reported in BC and its re-expression suppresses invasion and migration of BC cells^60^. *hsa-miR-335-5p* has exerted both oncogenic and tumor-suppressive effects. miR-335 was downregulated in gastric cancer and associated with features such as invasion degree, lymph node metastasis, and a poor prognosis in patients from Amerindian/Hispanic ancestry^61^. *hsa-miR-17-5p* was reported to be upregulated in bladder tumors compared to normal mucosa^62^. Overexpression of miR-17-5p was observed in BC. Further, its overexpression with the cluster miR-17–92 was reported in breast, lung, pancreas, colon, and prostate cancers^63,64^.

The second group of miRNAs consisted of two members: *hsa-miR-124-3p* and *hsa-let-7b-5p*. This group was associated with the overlap between NBMSC- PBC module groups and NBMSC-RNIT module groups. *hsa-miR-124-3p* was downregulated in lymphoblastic leukemia and was suggested to have tumor-suppressive effects in this type of cancer^65^. Functional regulation of hsa-*miR-124-3p* was performed by epigenetic inactivation possibly through methylation in human cancers including acute lymphoblastic leukemia^66,67^. Previous studies demonstrated that miR-124 had some effects such as the inflammatory phenotype, proliferation, and migration of pulmonary vascular fibroblasts^68,69^. Moreover, *miR-124-3p* was engaged in tumor progression via contribution to cell invasion and migration as well as drug resistance. It was also involved in wound healing, adipogenesis, and neural function by targeting different genes. Nevertheless, this miRNA has been recognized as a tumor repressor in a variety of cancers^70^. Downregulation of *miR-124-3p* was reported in BC, while its overexpression was associated with tumor migration and invasion^71^. *hsa-let-7b-5p*, which suppresses invasion, proliferation, and migration of glioma cells, is involved in the regulation of genes that potentially participate in the development of glioma^72^. Let-7b is a cancer suppressor gene that can inhibit the occurrence of cancer^73,74^.

The third group had only one member with *hsa-miR-106b-5p*. It was related to the overlap between NBMSC-RNIT and PBC-RNIT module groups. Upregulation of *miR-106b-5p* was reported in urothelial BC^75^ and in the pre-operative cell-free urine compared with postoperative cells. In addition, *miR-106b-5p* levels were associated with the advanced tumor stage^76^.

The fourth group included 2 miRNAs which were observed exclusively in NBMSC-PBC module groups. *miR-192-5p*, which was reported to be downregulated in BC. Lack of this miRNA promotes tumor growth, and its overexpression remarkably prevents this process. Therefore, miR-192-5p could be potentially used for therapeutic and diagnostic goals^77^. *miR-155-5p*, which was downregulated in BC^78^, nasopharyngeal carcinoma, oral cancer, as well as colon cancer^79,80^.

The fifth group was *miR-98-5p* which was exclusively observed in the NBMSC-RNIT module group. A meta-analysis study on six independent datasets identified miRNAs associated with prostate cancer. A significant upregulation of *miR-98-5p* was observed in recurrent vs. non-recurrent prostate cancer patients after radical prostatectomy surgery^81^. It was also suggested that the upregulation of miR-98-5p in prostate tissue and/or the plasma could be a potential diagnostic and prognostic biomarker^82^. In contrast, the downregulation of miR-98-5p has been observed in lung cancer tissue when compared to adjacent cancer-free tissue^83^.

Likewise, the sixth group had three miRNAs (i.e., *miR-20b-5p*, *miR-20a-5p*, and *miR-24-3p*) which belonged to PBC-RNIT module groups. Previous studies have demonstrated that the regulation of the cell cycle can occur by miR-20b-5p in BC. Also, miR-20b prevents the migration, invasion, and proliferation in cell lines of BC^84^. Yang et al. reported the high expression and overexpression of miR-20a-5p. Exogenous overexpression of miR-20a-5p enhance the migration, invasion, and proliferation of BC cells^85^. *miR-24-3p* is an outstanding miRNA involved in tumorigenesis and tumor progression. Several studies have reported the altered expression of *miR-24-3p* in malignancies such as pancreatic cancer^86^, gastric carcinoma^87^, and acute myelogenous leukemia^88^. It was explored using the BC disease by regulating *DEDD*, a member of the death effector domain-containing protein family. Experiments have indicated that *miR-24-3p* enhanced cell proliferation, invasion, and migration, along with tumor growth in BC^89^.

The proposed biomarkers of BC were mentioned as listed genes, and miRNAs were obtained using our proposed method. After that, we investigated the previous research studies, medical experiments, as well as clinical studies in the field of BC. Based on the results of the clinical and experimental studies and the investigation of the recently published authentic articles, it could be concluded that almost all of our identified genes and miRNAs have been reported in different studies conducted on numerous studies related to cancers. Most of them can be found in the latest articles^22,28,35,37,47^. The obtained genes in the present study include *CST4*, *CSNK1E*, *PTPN14*, *ETV6*, *ADRM1*, *KIF13B*, *IGFBP2*, *THSD4*, *PIFO*, *GRPEL2*, *ZNF264*, *CPOX*, *DANL1*, *SLC30A7*, and *WEE* have not been reported in the recent authentic bladder related studies. A large number of these genes were involved in metastasis or important functions in cancer but were not mentioned in BC. We suggest that these genes, the main results of our study, might be potentially associated with BC and count as significant biomarkers for BC.

Moreover, hsa-mir-16-5p, hsa-mir-335-5p, and hsa-let-7b-5p were introduced as BC-related miRNAs, which could be candidate biomarkers for BC. Eventually, as illustrated in Supplementary Table S16, we suggest that these biomarkers could be evaluated as tools for early diagnosis of BC.

Furthermore, the drug-gene networks were visualized. There was *dastatinib* in two drug-gene networks. *Dastatinib* is an oral SRC-family kinase (SFK) inhibitor. The effect of *dastatinib* was investigated in patients with muscle-invasive urothelial carcinoma of the bladder. The result of this study indicated a significant decrease in pre- and post-treatment tumors. In this study, significant inhibition of phosphorylated SFK (pSFK) has been observed without overall reduction of cellular proliferation or increase of apoptosis by using *dastatinib*^16^. Another therapeutic candidate drug was *methotrexate*, which is an antimetabolic agent affecting folic acid metabolism. This drug which resembles corticosteroid drugs affects the proliferation of connective tissue. *methotrexate* was used in the treatment of psoriasis, psoriatic arthritis, and rheumatoid arthritis^90^. *Methotrexate* with cisplatin and vinblastine chemotherapy was also applied in patients with muscle-invasive urothelial BC treated by cystectomy and/or radiotherapy. The effect of *methotrexate* was reported in the treatment of patients with advanced urinary BC. It is as active as *cisplatin* in the treatment of BC^17^. Likewise, *sorafenib* with anti-proliferative, anti-angiogenic, and pro-apoptotic effects inhibits several intracellular signaling kinases in tumor cells. Inhibitory actions of *sorafenib* have been confirmed in different BC cell lines. Further, the effects of *sorafenib* have been documented on phosphorylation, migration, and proliferation, along with the pro-apoptotic effects of the compound^91^. *Pazopanib* is a tyrosine kinase inhibitor (TKI), and its efficacy has been displayed in a variety of solid tumors^92^. Reduction of phospho-AKT levels by using *Pazopanib* has been observed in docetaxel-resistant BC cells^93^. Chemotherapy is done with the standard gemcitabine-cisplatin (GC) for advanced BC although the therapeutic effect of this type of therapy is limited due to chemo-resistance. *Disulfiram* (anti-alcoholic drug) was identified as a candidate for increasing sensitivity of GC since it has synergistic effects combined with *cisplatin* but not with gemcitabine in multiple cell lines^94^. Several Phase I clinical trials revealed that *disulfiram* is an effective example of a repurposed drug with anticancer effects^95^. Initiation of BC in mice exposed to butyl-N (4-hydroxybuty) nitrosamine was remarkably inhibited by *disulfiram*^96^. However, the operation mechanism of *disulfiram* in this process is unknown. *Cisplatin*, recognized as the most effective single-agent drug, is used for therapy of advanced BC. The use of *Cisplatin* is growing. The application of *Cisplatin* increased the local control rate after an increase in the incomplete transurethral surgery; however, survival improvement has not been observed^97^. *Bortezomib* is a proteasome inhibitor that is used for the treatment of advanced urothelial carcinoma^98^. Regimens containing *Cisplatin* (e.g., methotrexate, vinblastine, doxorubicin and cisplatin (MVAC), chemotherapy with methotrexate, vinblastine, and *Cisplatin*, as well as gemcitabine/cisplatin combinations) are popularly applied treatments^99^. Similarly, *bortezomib* affects several cellular regulatory mechanisms such as the suppression of tumor survival pathways, arresting of tumor growth, and tumor spread and angiogenesis^100^.

There were many drugs in the drug-gen network for NBMSC-RNIT. *dastatinib*, *ixazomib citrate*, *carfilzomib, bortezomib*, and *disulfiram* had more than two targets. In one study, a combination of ritonavir and *ixazomib* decreased intensive apoptosis and finally inhibited the growth of BC cells^19^. *Ixazomib* is currently used for a broad range of human malignancies. Several studies have confirmed the high potency of *ixazomib* against several cancer cell lines^101^. *Ixazomib* inhibits proteasome in both solid tumors and hematologic malignancies^101,102^. Another drug, *carfilzomib* is a selective and proteasome inhibitor investigated in patients with multiple myeloma. It exhibited promising results in clinical trials in multiple myeloma^103^.

There existed 97 drugs in the drug-gene network for PBC-RNIT. Ten drugs, including *sunitinib*, *imatinib*, *axitinib, palbociclib*, *allopurnol*, *gefitinib*, *olaparib*, *docetaxel*, and *trametinib* contained more than a gene target. *Sunitinib* indicated antiproliferative effects in KK47, KK47/DDP20, and KK47/ADR cell lines in vitro due to the suppression of ERK1/2 phosphorylation. Additionally, the antitumor effects of s*unitinib* were reported in a mouse model. Moreover, the effect of *sunitinib* was investigated in chemotherapy-resistant BC patients^20^. *Imatinib* is a selective TKI^104^, and its application is suggested to be concurrent with radiotherapy to treat muscle-invasive BC^105^. *Axitinib* is another drug that inhibits vascular endothelial growth factor receptors selectively and effectively^106^. It is suggested that the antitumor effect of this drug takes place through suppression of angiogenesis and is reversed upon discontinuation of *axitinib*^107^. In a considerable number of urothelial cancers, the genes involved in the retinoblastoma (Rb) pathway were affected, leading to RB loss of function. The function of RB, however, could be restored using a specific drug known as *palbociclib*, which suppresses CDK4/6. Subsequently, the restored activity of RB halts the cell cycle^108^. *Allopurnol* is another drug that was originally designed as a xanthine oxidase inhibitor. However, a line of studies indicated that allopurinol increased the risk of BC in animal models. According to Wang et al., formamide and *allopurnol* could initiate BC in rats via a synergistic mechanic^109^. Moreover, the role of *allopurnol* in BC in rats was reported in two separate studies by Fukushima et al.^110^ and Ito et al.^111^. An in vitro study evidenced that nanomolar concentrations of *gefitinib* inhibited cancer cells growth as this drug acted as an EGFR tyrosine kinase antagonist^112^. Despite remarkable results in preclinical models^113–115^, the effect of *gefitinib* was not as promising in individuals affected with different cancers such as non-small cell lung cancer^116^. According to the extensive and detailed review, some of the drugs extracted by this article were approved for treating of bladder cancer. Some have not been studied in clinical experiments that can be examined experimentally.

## Conclusion

To summarize, our proposed method aimed to conduct a prognostic study and introducing candidate drugs for BC. The gene co-expression network analysis method was administered based on the GEO database. Next, three bipartite networks (mRNA-miRNAs) reconstructed based on the significant module genes were obtained from co-expression networks. Therefore, we used three types of samples that belonged to one of the NBMSC, PBC, and RNIT tissues. Subsequently, the hub genes and miRNAs were identified as well. These genes and miRNAs had the highest connectivity degrees and were regarded as the potential prognostic biomarkers for BC. Based on the aim of study, it identified that the modules' genes target which miRNAs, and there are not any information about the expression of these miRNAs in different types of BC. Thus, the expression of these miRNAs are needed to valid in different types of BC. The novel genes, including (i.e., *PPARD*, *CST4*, *CSNK1E*, *PTPN14*, *ETV6*, and *ADRM1*) as well as novel miRNAs (i.e., *miR-16-5p, miR-335-5p, miR-124-3p,* and *let-7b-5p*) were introduced as BC-related proposed biomarkers which are recommended to be examined in the experimental studies such as clinical ones. A variety of treatments, including the following, are proven strategies for improving patient survival. Immunotherapy and targeted therapy as treatments to prevent advanced and metastatic cancer through targeted manipulation and chemotherapy. Nevertheless, genome instability and signal transduction pathway redundancy continue to pose challenges in the treatment of bladder cancer. Strategies are needed to improve therapeutic efficacy and interaction with other drugs in the preclinical environment. The present study further highlighted the importance of this issue in clinical application and its application as a means of developing strategies to discover and design new drugs.

## Methods

### Dataset and preprocessing

The gene expression data were downloaded from the National Center for Biotechnology Information (NCBI) and Gene Expression Omnibus (GEO) (https://www.ncbi.nlm.nih.gov/geo) using the GSE13507 accession number. The platform of the chip analyzer was GPL6102 (Illumina human-6 V2.0 Expression BeadChip). The data contained an mRNA expression profile for three different tissues. Table 2 depicts the properties of this dataset, representing 165 PBC samples, 58 NBMSC samples, and 23 RNIT samples.

**Table 2:** Description of microarray dataset.

Dataset	Type of samples	PBC	BMSC	RNIT	Expression array
GSE13507	mRNA	165 (Male: 135, Female: 30)	58	23	Illumina human-6 v2.0 expression beadchip

First, the non-gene transcripts were removed from the original file before the preprocessing of the dataset. Then, differentially expressed genes (DEGs) were extracted for all samples based on ANOVA test results in the R environment by using the Limma package from the Bioconductor project. Benjamini and Hochberg’s false discovery rate method^14^ was used to calculate the adjusted *p*-values. The genes were assigned to their related IDs. ID-less genes were subsequently removed after sorting the data. Genes with an adjusted *p*-value less than 0.01 were considered for further analyses. After preprocessing, 5563 genes remained, and the remaining ones were removed. This list allowed us to construct the network and perform further analysis. The list of up-regulated and downregulated genes are provided for each comparison in Supplementary Tables S31–S36. There were 2930, 2574 and 2574 up-regulated genes in PBC-RNIT, NBMSC-PBC, and NBMSC-RNIT, respectively. Also, down-regulated genes were 2693, 2989, and 2993 in PBC-RNIT, NBMSC-PBC, and NBMSC-RNIT, respectively. All DEGs were used to construct the network and perform further analysis.

## Weighted gene co‑expression network analysis (WGCNA)

Initially, 5563 genes were used to construct the co-expression network. Afterwards, three co-expression networks from PBC, NBMSC, and RNIT mRNA expression data were reconstructed using the WGCNA package. To adjust the scale-free property of the networks, the *β* parameter (soft thresholding power beta) was used. It was then set to 5, 4, and 5 for NBMSC-PBC, PBC-RNIT, and NBMSC-RNIT networks, respectively. Scale independency by R^2^ and the mean connectivity along with different values of the soft threshold are presented in Supplementary Fig. S1 for NBMSC-PBC. Supplementary Fig. S1 displays the soft threshold values for the PBC-RNIT and NBMSC-RNIT. Among the powers ranging from 1 to 20, the value of 5 was selected for *β* to gain the scale independence of the network at NBMSC-PBC and NBMSC-RNIT, where the scale free index R^2^ was 0.9. Likewise, the *β* value of 4 was selected for PBC- RNIT. The topological overlap matrix was calculated after generating the adjacency matrix of the expression data. Hierarchical clustering in WGCNA was used to extract modules for each co-expression network. Furthermore, *DeepSplit* and minimal module size parameters were assigned 2 and 30 values, respectively, by examining different parameters. Then, the extracted modules were merged and labeled with colors.

In this study, the Z_summary_ score was utilized to analyze module preservation. Z_summary_ is obtained after calculating Z_density_ and Z_connectivity_, from half of the sum of Z_density_ and Z_connectivity_^117^ that it states the module preservation summary statistics^118^. Z_summary_ is used to evaluate the significance of observed statistics by identifying the preserved from the non-preserved modules^119,120^. The values of Z_summary_ less than 2, between 2 and 10, and equal or more than 10 were regarded as not preserved, moderately preserved, and strongly preserved, respectively. The modules with Z_summary_ values of more than 10 did not provide any information because they were strongly preserved; as a result, these modules were not used. There were no modules with a Z_summary_ value smaller than 2. Supplementary Fig. S2 illustrates the preservation of median rank and preservation of Z_summary_ along with the module size.

### mRNA–miRNA bipartite network reconstruction

First, we extracted miRNAs targeting genes in the NBMSE-PBC modules from the miRWalk 2.0 database (http://zmf.umm.uni-heidelberg.de/mirwalk2)^121^. Given that the mRNA-miRNA interactions were experimentally validated, we reconstructed a bipartite network using miRNAs, and genes obtained from the NBMSE-PBC modules revealed cancer progression from NBMSC to PBC. There were 273 genes in this subnetwork.

Similarly, the second and third bipartite networks were reconstructed from genes in the PBC-RNIT and NBMSC-RNIT modules, and their interacting miRNAs exhibited the progression of BC from PBC to RNIT and from NBMSC to RNIT, respectively. The PBC-RNIT and NBMSC-RNIT contained 171 and 149 genes, respectively. Subsequent visualization of these networks was performed by Cytoscape v.3.8.2 software^122^. After analyzing the relations among genes and their interacting miRNAs, hub miRNAs with the highest level of connectivity were retained to maintain significant connections and avoid complexity.

### Enrichment analysis of genes and mRNAs and miRNAs

We performed the functional enrichment analysis for genes in three sets of significant low-preserved modules (e.g., NBMSC-PBC, PBC-RNIT, and NBMSC-RNIT modules). DAVID bioinformatics tool (https://david.ncifcrf.gov/tools.jsp) was employed for functional enrichment of GO and Reactome pathways analyses. At this stage, the hub miRNAs were evaluated using the TAM tool (http://www.lirmed.com/tam2)^123^ while the *p*-value was less than 0.01. Further, the family and functionality of the miRNAs were determined using the TAM tool by default parameters.

### Drug- gene network construction

After reconstruction of three mRNA-miRNA bipartite networks, DGIdb (www.dgidb.org)^124^ was applied to identify some candidate drugs for repurposing against the genes in bipartite networks as potential therapies. Then, the drug-gene network was established for the extracted gene. After obtaining drug-gene interactions for all genes, the entire drug-gene interactions were gathered and reconstructed as a single drug-gene network. Finally, three drug-gene networks were reconstructed.

## Supplementary Information

Supplementary Information.

## Data Availability

The corresponding author can provide the datasets utilized in this study on a reasonable request. The raw dataset is available on Information Gene expression Omnibus (GEO) with GSE13507 accession number (https://www.ncbi.nlm.nih.gov/geo/query/acc.cgi?acc=gse13507). (https://www.ncbi.nlm.nih.gov/geo/query/acc.cgi?acc=gse13507).
