# Supplementary material for: mRNA–miRNA bipartite networks reconstruction in different tissues of bladder cancer based on gene co-expression network analysis
Source: Sci Rep. 2022 Apr 7;12:5885. doi: 10.1038/s41598-022-09920-4 (PMC8991185; doi:10.1038/s41598-022-09920-4)
Supplement: Supplementary file 1 — Supplementary Information. [file 41598_2022_9920_MOESM1_ESM.docx]

**mRNA-miRNA bipartite networks reconstruction in different tissues of bladder cancer based on gene co-expression network analysis**

Zahra Abedi1, Habib MotieGhader2*, Sahar Sadat Hosseini1, Mohammad Ali Sheikh Beig Goharrizi3, Ali Masoudi-Nejad1*

1. Laboratory of Systems Biology and Bioinformatics (LBB), Institute of Biochemistry and Biophysics, University of Tehran, Tehran, Iran.

2. Department of Biology, Tabriz Branch, Islamic Azad University, Tabriz, Iran.

3. Atherosclerosis Research Center, Baqiyatallah University of Medical Sciences, Tehran, Iran.

* Corresponding author: Habib MotieGhader; contact info: [habib_moti@ut.ac.ir](mailto:habib_moti@ut.ac.ir)

* Corresponding author: Ali Masoudi-Nejad; contact info: E-mail: [amasoudin@ut.ac.ir](mailto:amasoudin@ut.ac.ir%20)


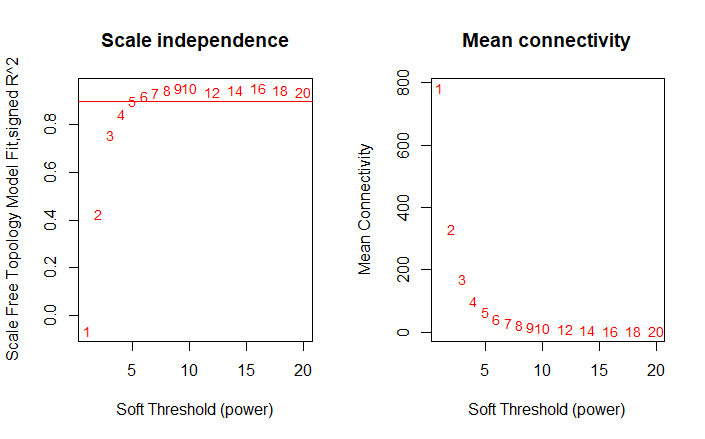


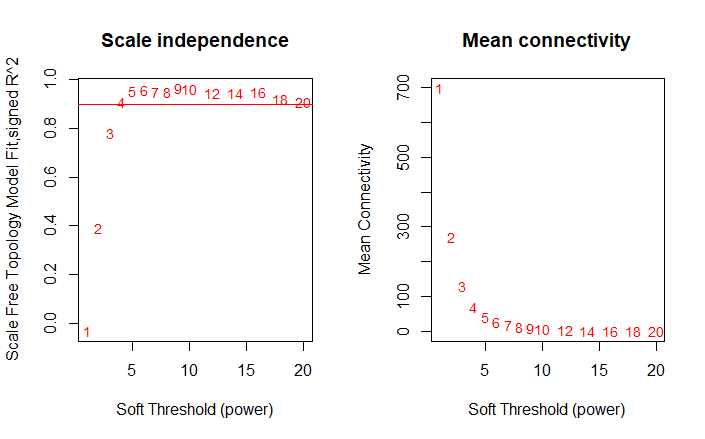


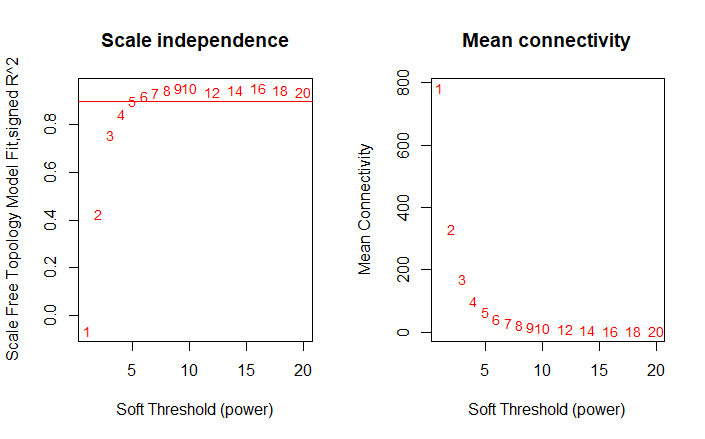


**Figure S1.** Analysis of network topology for several soft-thresholding powers for NBMSC-PBC (a), PBC-RNIT (b) and NBMSC-RNIT (c). The left panel displays scale-free topology index and right panel shows mean connectivity for each power value.

**
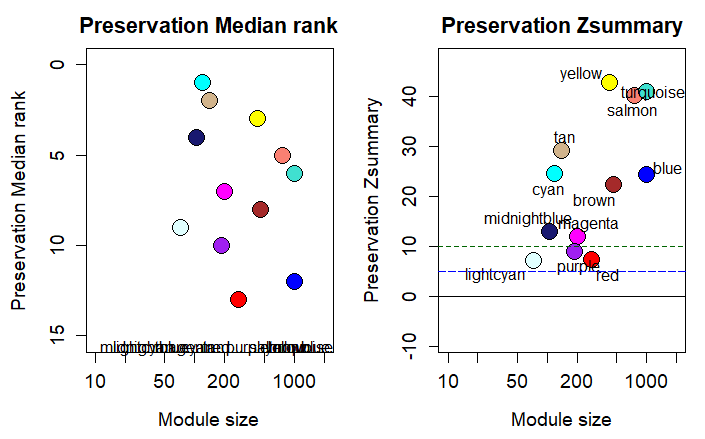
**

**a**

**
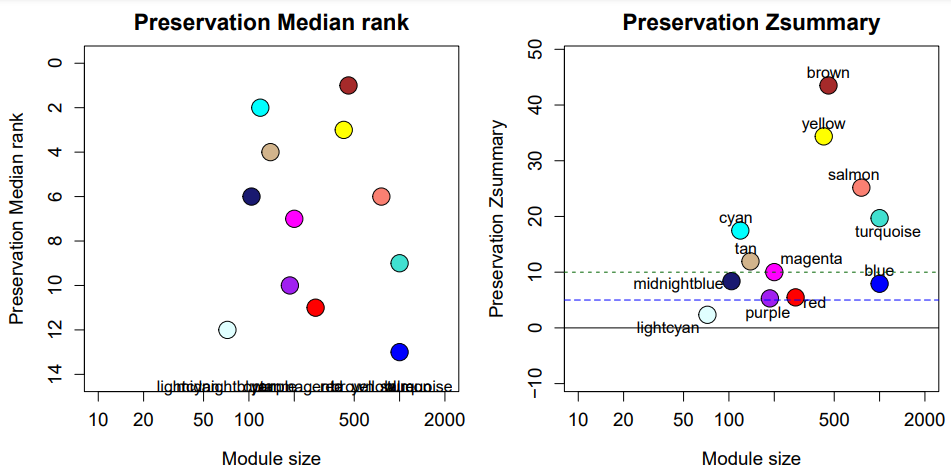
**

**b**

**
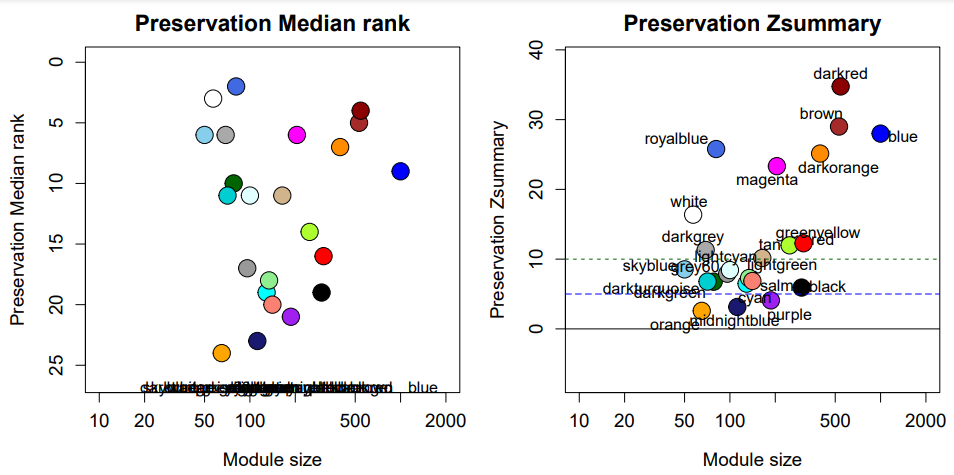
**

**c**

**Figure S2.** Modules of the NBMSC tissue against the PBC expression data according to their Z_summary_ (a); Modules of the NBMSC tissue against the RNIT expression data according to their Zsummary (b); Modules of the PBC tissue against the RNIT expression data according to their Z_summary_ (c).

| **Biological process** | **Count** | **p Value** | **Genes** |
| --- | --- | --- | --- |
| Apoptotic process | 14 | 2.56E-07 | PPP1R15A, CSRNP1, PLK3, GADD45B, GADD45A, AXIN1, C8ORF4, TNFAIP3, NR4A1, IL1B, MAP3K8, SGK1, IER3, PPARD |
| Skeletal muscle cell differentiation | 5 | 4.04E-05 | EGR1, NR4A1, EGR2, MAFF, FOS |
| Positive regulation of transcription from RNA polymerase II promoter | 14 | 9.67E-05 | EGR1, CSRNP1, EGR2, DDX3X, CEBPD, FOS, KLF4, ETS2, NR4A2, NR4A1, IL1B, MAFF, FOSB, NCOA7 |
| Positive regulation of transcription, DNA-templated | 10 | 1.75E-04 | MAP2K3, EGR1, KLF6, EGR2, IL1B, AXIN1, FOS, KLF4, ETS2, PPARD |
| Fat cell differentiation | 5 | 1.93E-04 | NR4A2, NR4A1, EGR2, CEBPD, KLF4 |
| Negative regulation of inflammatory response | 5 | 2.62E-04 | SOCS3, ZFP36, TNFAIP3, KLF4, PPARD |
| Embryo implantation | 4 | 5.97E-04 | IL1B, STC1, PTGS2, PPARD |
| Inflammatory response | 8 | 7.00E-04 | IL1B, CCL4, CCL3, TNFAIP3, FOS, PTGS2, THBS1, TNFRSF10D |
| Cellular response to hormone stimulus | 4 | 7.31E-04 | DUSP1, FOSB, SIK1, FOS |
| Activation of MAPK activity | 5 | 8.29E-04 | MAP2K3, DUSP5, IL1B, TAB3, THBS1 |
| Transcription from RNA polymerase II promoter | 9 | 8.70E-04 | EGR1, CSRNP1, EGR2, CEBPD, NOCT, MAFF, FOSB, FOS, KLF4 |
| Endoderm formation | 3 | 9.79E-04 | DUSP5, DUSP2, DUSP1 |
| Cellular response to organic cyclic compound | 4 | 0.0016112 | TIPARP, IL1B, AXIN1, CCL3 |
| Positive regulation of calcium ion import | 3 | 0.001762 | LGALS3, CCL3, STC1 |
| Negative regulation of apoptotic process | 8 | 0.0020094 | SOCS3, PLK3, DDX3X, DUSP1, THBS1, IER3, TNFRSF10D, PPARD |
| Positive regulation of JNK cascade | 4 | 0.0021288 | GADD45B, GADD45A, IL1B, AXIN1 |
| Neutrophil chemotaxis | 4 | 0.002224 | LGALS3, IL1B, CCL4, CCL3 |
| Negative regulation of cysteine-type endopeptidase activity involved in apoptotic process | 4 | 0.0025251 | NR4A1, DDX3X, KLF4, THBS1 |
| Decidualization | 3 | 0.0027618 | STC1, PTGS2, PPARD |
| Positive regulation of NF-kappaB import into nucleus | 3 | 0.0030448 | IL1B, C8ORF4, PTGS2 |
| MAPK cascade | 6 | 0.0036652 | MAP2K3, DUSP5, ZFP36, IL1B, CCL3, HBEGF |
| Response to lipopolysaccharide | 5 | 0.0039499 | NOCT, PELI1, FOS, PTGS2, TNFRSF10D |
| Inactivation of MAPK activity | 3 | 0.0043059 | DUSP5, DUSP2, DUSP1 |
| Signal transduction | 12 | 0.0052569 | NR4A2, MAP2K3, NR4A1, RASD1, IL1B, CCL4, PPFIA1, AXIN1, PIP5K1A, CSNK1E, TNFRSF10D, HBEGF |
| Negative regulation of smooth muscle cell proliferation | 3 | 0.0057687 | TNFAIP3, KLF4, PPARD |
| Positive regulation of apoptotic process | 6 | 0.0064701 | NR4A1, DDX3X, GADD45B, DUSP1, GADD45A, PTGS2 |
| Peptidyl-tyrosine dephosphorylation | 4 | 0.0069535 | DUSP5, DUSP2, DUSP1, PTPN14 |
| Negative regulation of transcription from RNA polymerase II promoter | 9 | 0.0070261 | NR4A2, EGR1, PLK3, ZFP36, BHLHE40, FOSB, KLF4, ETS2, PPARD |
| Protein kinase B signaling | 3 | 0.0074267 | PLK3, IL1B, CCL3 |
| cellular response to tumor necrosis factor | 4 | 0.00928 | ZFP36, CCL4, CCL3, THBS1 |
| Intracellular receptor signaling pathway | 3 | 0.0097644 | NR4A2, NR4A1, PPARD |
| **Table S1.** The GO results for lightcyan module in NBMSC- PBC | | | |

| **Biological process** | **Count** | **p Value** | **Genes** |
| --- | --- | --- | --- |
| Translation | 10 | 5.26E-04 | COA1, MRPL19, EIF2AK2, ZNF525, RPSA, MRPL9, HARS2, RPS24, GCN1, SLC25A13 |
| Transcription, DNA-templated | 33 | 6.79E-04 | ZNF551, ANP32A, CHD7, ZBTB45, ZNF2, TFB1M, NLK, C14ORF169, ZNF280D, YY1, NKRF, SAP30L, ZNF525, E2F3, ZNF766, ZNF227, ZNF544, ZNF785, ZNF485, ZNF562, ZNF264, ZNF165, ZNF140, ZNF480, EIF2AK2, SNF8, VAX2, MED26, ZNF33B, ZFP64, ZNF613, ZNF558, ZNF777 |
| Regulation of transcription, DNA-templated | 25 | 0.004801 | ZNF551, ANP32A, CHD7, ZBTB45, ZNF2, TFB1M, NLK, SAP30L, ZNF525, ZNF766, ZNF227, ZNF544, ZNF785, ZNF485, ZNF562, ZNF264, ZNF165, ZNF140, ZNF480, ZNF33B, ZFP64, ZNF613, ZNF558, ZNF777, ABCG1 |
| Cellular response to UV | 4 | 0.007474 | YY1, FBXW7, TRIAP1, RHNO1 |

**Table S2.** The GO results for purple module in NBMSC- PBC

| **Biological process** | **Count** | **p Value** | **Genes** |
| --- | --- | --- | --- |
| Oxidation-reduction process | 20 | 5.88E-04 | ABCC4, MOXD1, CYB5A, VCAM1, SRD5A2, HHIP, P3H2, DBH, PTGR1, CRYZ, CYP27A1, ALDH3A1, SCD, ALDH1A2, PHYHD1, ALDH1A1, STEAP1, PIGF, STEAP2, SNCA |
| Negative regulation of osteoblast differentiation | 5 | 0.001998446 | ID2, PTCH1, ID3, HOXA2, RORB |
| Inner ear development | 5 | 0.002408177 | SHH, PLPPR4, EYA4, CYTL1, DLL1 |
| Cellular response to retinoic acid | 6 | 0.002814102 | NTRK3, ALDH1A2, KRT13, HOXA2, RORB, FZD10 |
| Cytokine production | 4 | 0.004783638 | FABP4, TXK, DBH, LIPA |
| Retina morphogenesis in camera-type eye | 3 | 0.005037782 | MAN2A1, PROM1, DLL1 |
| Metanephros development | 4 | 0.005961538 | OSR2, SHH, ID2, ID3 |
| Extracellular matrix organization | 9 | 0.005994214 | POSTN, VCAM1, B4GALT1, COL13A1, LAMC3, LAMA3, ADAMTSL2, NDNF, SERPINB5 |
| Phospholipid metabolic process | 5 | 0.006542132 | PLPPR4, PLPPR5, PLA2G4A, PLPP1, SNCA |
| Pituitary gland development | 4 | 0.006609703 | BMP4, SALL1, ALDH1A2, ISL1 |
| Embryonic skeletal system development | 4 | 0.008027577 | HOXA9, SHH, RBP4, FGF9 |
| Aging | 8 | 0.008097838 | ALDH3A1, VCAM1, TRPC6, IGFBP2, CAT, GNRH1, ASS1, SNCA |
| Response to estradiol | 6 | 0.00856994 | POSTN, ALDH1A2, PTCH1, IGFBP2, CAT, ASS1 |
| Dorsal/ventral pattern formation | 4 | 0.009611094 | SHH, PTCH1, HHIP, HOXA2 |
| Embryonic forelimb morphogenesis | 4 | 0.009611094 | OSR2, HOXA9, SHH, ALDH1A2 |
| Phospholipid dephosphorylation | 3 | 0.009629076 | PLPPR4, PLPPR5, PLPP1 |
| Type B pancreatic cell development | 3 | 0.009629076 | BMP4, DLL1, BMP5 |
| Response to drug | 11 | 0.009860748 | ABCC4, ALDH3A1, SRD5A2, TRPA1, SCN11A, PTCH1, IGFBP2, CAT, ASS1, SLC26A5, SNCA |

**Table S3.** The GO results for red module in NBMSC- PBC

| **Biological process** | **Count** | **p Value** | **Genes** |
| --- | --- | --- | --- |
| Negative regulation of neuron death | 4 | 0.001274421 | GPNMB, CDK5, STAT3, CHMP4B |
| Dicarboxylic acid transport | 2 | 0.015994008 | SLC13A2, SLC1A7 |
| Phosphorylation | 4 | 0.016569162 | CDK5, CKMT1A, STAT3, CKMT1B |
| Skeletal muscle tissue development | 3 | 0.030562767 | CDK5, MYH14, SIX1 |
| Cell differentiation | 7 | 0.037734451 | CATSPER1, PTPRU, ONECUT2, ZIC2, PPDPF, ATRAID, ETS2 |
| Somatic stem cell population maintenance | 3 | 0.047527203 | STAT3, WNT7A, POU5F1 |

**Table S4.** The GO results for midnight blue module in PBC-RNIT

| **Biological process** | **Count** | **p Value** | **Genes** |
| --- | --- | --- | --- |
| Cell-substrate adhesion | 2 | 3.076923077 | TTYH1, PPARD |
| Positive regulation of transcription, DNA-templated | 5 | 7.692307692 | KLF7, IL5, CHEK2, HAND2, PPARD |
| Positive regulation of GTPase activity | 5 | 7.692307692 | GIT2, IL5, ARAP2, FGF1, SOS2 |
| Transcription from RNA polymerase III promoter | 2 | 3.076923077 | CRCP, POLR3A |

**Table S5.** The GO results for orange module in PBC-RNIT

| **Biological process** | **Count** | **p Value** | **Genes** |
| --- | --- | --- | --- |
| Cell-substrate adhesion | 2 | 0.051383785 | TTYH1, PPARD |
| Positive regulation of transcription, DNA-templated | 5 | 0.074774772 | KLF7, IL5, CHEK2, HAND2, PPARD |
| Positive regulation of GTPase activity | 5 | 0.097093157 | GIT2, IL5, ARAP2, FGF1, SOS2 |
| Transcription from RNA polymerase III promoter | 2 | 0.097374804 | CRCP, POLR3A |

**Table S6.** The GO results for purple module in PBC-RNIT

| **Biological process** | **Count** | **p Value** | **Genes** |
| --- | --- | --- | --- |
| Apoptotic process | 14 | 2.56E-07 | PPP1R15A, CSRNP1, PLK3, GADD45B, GADD45A, AXIN1, C8ORF4, TNFAIP3, NR4A1, IL1B, MAP3K8, SGK1, IER3, PPARD |
| Skeletal muscle cell differentiation | 5 | 4.04E-05 | EGR1, NR4A1, EGR2, MAFF, FOS |
| Positive regulation of transcription from RNA polymerase II promoter | 14 | 9.67E-05 | EGR1, CSRNP1, EGR2, DDX3X, CEBPD, FOS, KLF4, ETS2, NR4A2, NR4A1, IL1B, MAFF, FOSB, NCOA7 |
| Positive regulation of transcription, DNA-templated | 10 | 1.75E-04 | MAP2K3, EGR1, KLF6, EGR2, IL1B, AXIN1, FOS, KLF4, ETS2, PPARD |
| Fat cell differentiation | 5 | 1.93E-04 | NR4A2, NR4A1, EGR2, CEBPD, KLF4 |
| Negative regulation of inflammatory response | 5 | 2.62E-04 | SOCS3, ZFP36, TNFAIP3, KLF4, PPARD |
| Embryo implantation | 4 | 5.97E-04 | IL1B, STC1, PTGS2, PPARD |
| Inflammatory response | 8 | 7.00E-04 | IL1B, CCL4, CCL3, TNFAIP3, FOS, PTGS2, THBS1, TNFRSF10D |
| Cellular response to hormone stimulus | 4 | 7.31E-04 | DUSP1, FOSB, SIK1, FOS |
| Activation of MAPK activity | 5 | 8.29E-04 | MAP2K3, DUSP5, IL1B, TAB3, THBS1 |
| Transcription from RNA polymerase II promoter | 9 | 8.70E-04 | EGR1, CSRNP1, EGR2, CEBPD, NOCT, MAFF, FOSB, FOS, KLF4 |
| Endoderm formation | 3 | 9.79E-04 | DUSP5, DUSP2, DUSP1 |
| Cellular response to organic cyclic compound | 4 | 0.00161116 | TIPARP, IL1B, AXIN1, CCL3 |
| Positive regulation of calcium ion import | 3 | 0.00176204 | LGALS3, CCL3, STC1 |
| Negative regulation of apoptotic process | 8 | 0.00200944 | SOCS3, PLK3, DDX3X, DUSP1, THBS1, IER3, TNFRSF10D, PPARD |
| Positive regulation of JNK cascade | 4 | 0.00212881 | GADD45B, GADD45A, IL1B, AXIN1 |
| Neutrophil chemotaxis | 4 | 0.00222396 | LGALS3, IL1B, CCL4, CCL3 |
| Negative regulation of cysteine-type endopeptidase activity involved in apoptotic process | 4 | 0.00252514 | NR4A1, DDX3X, KLF4, THBS1 |
| Decidualization | 3 | 0.00276177 | STC1, PTGS2, PPARD |
| Positive regulation of NF-kappaB import into nucleus | 3 | 0.00304477 | IL1B, C8ORF4, PTGS2 |
| MAPK cascade | 6 | 0.00366522 | MAP2K3, DUSP5, ZFP36, IL1B, CCL3, HBEGF |
| Response to lipopolysaccharide | 5 | 0.00394987 | NOCT, PELI1, FOS, PTGS2, TNFRSF10D |
| Inactivation of MAPK activity | 3 | 0.00430587 | DUSP5, DUSP2, DUSP1 |
| Signal transduction | 12 | 0.0052569 | NR4A2, MAP2K3, NR4A1, RASD1, IL1B, CCL4, PPFIA1, AXIN1, PIP5K1A, CSNK1E, TNFRSF10D, HBEGF |
| Negative regulation of smooth muscle cell proliferation | 3 | 0.00576867 | TNFAIP3, KLF4, PPARD |
| Positive regulation of apoptotic process | 6 | 0.0064701 | NR4A1, DDX3X, GADD45B, DUSP1, GADD45A, PTGS2 |
| Peptidyl-tyrosine dephosphorylation | 4 | 0.00695346 | DUSP5, DUSP2, DUSP1, PTPN14 |
| Negative regulation of transcription from RNA polymerase II promoter | 9 | 0.00702608 | NR4A2, EGR1, PLK3, ZFP36, BHLHE40, FOSB, KLF4, ETS2, PPARD |
| Protein kinase B signaling | 3 | 0.00742674 | PLK3, IL1B, CCL3 |
| Cellular response to tumor necrosis factor | 4 | 0.00928003 | ZFP36, CCL4, CCL3, THBS1 |
| Intracellular receptor signaling pathway | 3 | 0.00976439 | NR4A2, NR4A1, PPARD |

**Table S7.** The GO results for lightcyan module in NBMSC-RNIT

| **Biological process** | **Count** | **p Value** | **Genes** |
| --- | --- | --- | --- |
| Translation | 10 | 5.26E-04 | COA1, MRPL19, EIF2AK2, ZNF525, RPSA, MRPL9, HARS2, RPS24, GCN1, SLC25A13 |
| Transcription, DNA-templated | 33 | 6.79E-04 | ZNF551, ANP32A, CHD7, ZBTB45, ZNF2, TFB1M, NLK, C14ORF169, ZNF280D, YY1, NKRF, SAP30L, ZNF525, E2F3, ZNF766, ZNF227, ZNF544, ZNF785, ZNF485, ZNF562, ZNF264, ZNF165, ZNF140, ZNF480, EIF2AK2, SNF8, VAX2, MED26, ZNF33B, ZFP64, ZNF613, ZNF558, ZNF777 |
| Regulation of transcription, DNA-templated | 25 | 0.00480107 | ZNF551, ANP32A, CHD7, ZBTB45, ZNF2, TFB1M, NLK, SAP30L, ZNF525, ZNF766, ZNF227, ZNF544, ZNF785, ZNF485, ZNF562, ZNF264, ZNF165, ZNF140, ZNF480, ZNF33B, ZFP64, ZNF613, ZNF558, ZNF777, ABCG1 |
| Cellular response to UV | 4 | 0.00747438 | YY1, FBXW7, TRIAP1, RHNO1 |

**Table S8.** The GO results for purple module in NBMSC-RNIT

| **Biological process** | **Count** | **p Value** | **Genes** |
| --- | --- | --- | --- |
| Oxidation-reduction process | 20 | 5.88E-04 | ABCC4, MOXD1, CYB5A, VCAM1, SRD5A2, HHIP, P3H2, DBH, PTGR1, CRYZ, CYP27A1, ALDH3A1, SCD, ALDH1A2, PHYHD1, ALDH1A1, STEAP1, PIGF, STEAP2, SNCA |
| Negative regulation of osteoblast differentiation | 5 | 0.00199845 | ID2, PTCH1, ID3, HOXA2, RORB |
| Inner ear development | 5 | 0.00240818 | SHH, PLPPR4, EYA4, CYTL1, DLL1 |
| Cellular response to retinoic acid | 6 | 0.0028141 | NTRK3, ALDH1A2, KRT13, HOXA2, RORB, FZD10 |
| Cytokine production | 4 | 0.00478364 | FABP4, TXK, DBH, LIPA |
| Retina morphogenesis in camera-type eye | 3 | 0.00503778 | MAN2A1, PROM1, DLL1 |
| Metanephros development | 4 | 0.00596154 | OSR2, SHH, ID2, ID3 |
| Extracellular matrix organization | 9 | 0.00599421 | POSTN, VCAM1, B4GALT1, COL13A1, LAMC3, LAMA3, ADAMTSL2, NDNF, SERPINB5 |
| Phospholipid metabolic process | 5 | 0.00654213 | PLPPR4, PLPPR5, PLA2G4A, PLPP1, SNCA |
| Pituitary gland development | 4 | 0.0066097 | BMP4, SALL1, ALDH1A2, ISL1 |
| Embryonic skeletal system development | 4 | 0.00802758 | HOXA9, SHH, RBP4, FGF9 |
| Aging | 8 | 0.00809784 | ALDH3A1, VCAM1, TRPC6, IGFBP2, CAT, GNRH1, ASS1, SNCA |
| Response to estradiol | 6 | 0.00856994 | POSTN, ALDH1A2, PTCH1, IGFBP2, CAT, ASS1 |
| Dorsal/ventral pattern formation | 4 | 0.00961109 | SHH, PTCH1, HHIP, HOXA2 |
| Embryonic forelimb morphogenesis | 4 | 0.00961109 | OSR2, HOXA9, SHH, ALDH1A2 |
| Phospholipid dephosphorylation | 3 | 0.00962908 | PLPPR4, PLPPR5, PLPP1 |
| Type B pancreatic cell development | 3 | 0.00962908 | BMP4, DLL1, BMP5 |
| Response to drug | 11 | 0.00986075 | ABCC4, ALDH3A1, SRD5A2, TRPA1, SCN11A, PTCH1, IGFBP2, CAT, ASS1, SLC26A5, SNCA |

**Table S9.** The GO results for red module in NBMSC-RNIT

| **Reactome pathway** | **Count** | **pValue** | **Genes** |
| --- | --- | --- | --- |
| Signaling by Interleukins | 12 | 1.21E-05 | MAP2K3, SOCS3, CEBPD, IL1B, CCL4, PELI1, CCL3, TAB3, MAP3K8, FOS, PTGS2, PTPN14 |
| Cytokine Signaling in Immune system | 14 | 3.10E-05 | MAP2K3, EGR1, CEBPD, FOS, PTGS2, PTPN14, SOCS3, IL1B, CCL4, PELI1, CCL3, TAB3, MAP3K8, KPNA1 |
| NGF-stimulated transcription | 5 | 3.82E-05 | EGR1, EGR2, FOSB, FOS, SGK1 |
| Signal Transduction | 26 | 1.68E-04 | PPP1R15A, TNFAIP3, RND3, THBS1, RND1, SOCS3, CCL4, CCL3, PIP5K1A, CD55, IER3, EGR1, DUSP5, EGR2, DUSP2, DUSP1, AXIN1, CSNK1E, FOS, TNFRSF10D, NR4A1, FOSB, TAB3, SGK1, PPARD, HBEGF |
| Nuclear Events (kinase and transcription factor activation) | 5 | 2.24E-04 | EGR1, EGR2, FOSB, FOS, SGK1 |
| Toll Like Receptor 10 (TLR10) Cascade | 5 | 7.95E-04 | MAP2K3, PELI1, TAB3, MAP3K8, FOS |
| Toll Like Receptor 5 (TLR5) Cascade | 5 | 7.95E-04 | MAP2K3, PELI1, TAB3, MAP3K8, FOS |
| MyD88 cascade initiated on plasma membrane | 5 | 7.95E-04 | MAP2K3, PELI1, TAB3, MAP3K8, FOS |
| TRAF6 mediated induction of NFkB and MAP kinases upon TLR7/8 or 9 activation | 5 | 0.001027 | MAP2K3, PELI1, TAB3, MAP3K8, FOS |
| MyD88 dependent cascade initiated on endosome | 5 | 0.001069 | MAP2K3, PELI1, TAB3, MAP3K8, FOS |
| Toll Like Receptor 7/8 (TLR7/8) Cascade | 5 | 0.001113 | MAP2K3, PELI1, TAB3, MAP3K8, FOS |
| Toll Like Receptor 9 (TLR9) Cascade | 5 | 0.001253 | MAP2K3, PELI1, TAB3, MAP3K8, FOS |
| Toll Like Receptor TLR6:TLR2 Cascade | 5 | 0.001512 | MAP2K3, PELI1, TAB3, MAP3K8, FOS |
| MyD88:MAL(TIRAP) cascade initiated on plasma membrane | 5 | 0.001512 | MAP2K3, PELI1, TAB3, MAP3K8, FOS |
| Interleukin-10 signaling | 4 | 0.001577 | IL1B, CCL4, CCL3, PTGS2 |
| Toll Like Receptor TLR1:TLR2 Cascade | 5 | 0.001685 | MAP2K3, PELI1, TAB3, MAP3K8, FOS |
| Toll Like Receptor 2 (TLR2) Cascade | 5 | 0.001685 | MAP2K3, PELI1, TAB3, MAP3K8, FOS |
| RNA Polymerase II Transcription | 16 | 0.001824 | PLK3, ZNF394, GADD45A, AXIN1, SLC2A3, FOS, KLF4, THBS1, ELL2, TNFRSF10D, NR4A2, LGALS3, NR4A1, SOCS3, SGK1, PPARD |
| Interleukin-4 and Interleukin-13 signaling | 5 | 0.001935 | SOCS3, CEBPD, IL1B, FOS, PTGS2 |
| Generic Transcription Pathway | 15 | 0.002122 | PLK3, ZNF394, GADD45A, AXIN1, SLC2A3, FOS, KLF4, THBS1, TNFRSF10D, NR4A2, LGALS3, NR4A1, SOCS3, SGK1, PPARD |
| Signaling by NTRK1 (TRKA) | 5 | 0.002434 | EGR1, EGR2, FOSB, FOS, SGK1 |
| MAP kinase activation | 4 | 0.00382 | MAP2K3, TAB3, MAP3K8, FOS |
| Signaling by NTRKs | 5 | 0.004221 | EGR1, EGR2, FOSB, FOS, SGK1 |
| Toll Like Receptor 4 (TLR4) Cascade | 5 | 0.004334 | MAP2K3, PELI1, TAB3, MAP3K8, FOS |
| Gene expression (Transcription) | 16 | 0.004774 | PLK3, ZNF394, GADD45A, AXIN1, SLC2A3, FOS, KLF4, THBS1, ELL2, TNFRSF10D, NR4A2, LGALS3, NR4A1, SOCS3, SGK1, PPARD |
| Circadian Clock | 4 | 0.004915 | NOCT, BHLHE40, SIK1, CSNK1E |
| Interleukin-1 family signaling | 5 | 0.005057 | IL1B, PELI1, TAB3, MAP3K8, PTPN14 |
| Interleukin-17 signaling | 4 | 0.005319 | MAP2K3, TAB3, MAP3K8, FOS |
| RAF-independent MAPK1/3 activation | 3 | 0.005655 | DUSP5, DUSP2, DUSP1 |
| Immune System | 19 | 0.005924 | MAP2K3, EGR1, DDX3X, CEBPD, TNFAIP3, SLC2A3, FOS, PTGS2, PTPN14, LGALS3, SOCS3, IL1B, CCL4, PELI1, CCL3, TAB3, MAP3K8, CD55, KPNA1 |
| Toll-like Receptor Cascades | 5 | 0.00737 | MAP2K3, PELI1, TAB3, MAP3K8, FOS |

**Table S10.** Reactome pathway database results for lightcyan module in NBMSC- PBC

| Reactome pathway | Count | PValue | Genes |
| --- | --- | --- | --- |
| Gene expression (Transcription) | 31 | 4.55E-05 | ZNF551, PSMD11, CBFB, ICE2, ZNF2, TRIAP1, YY1, SAP30L, DROSHA, ZNF227, ZNF544, BID, ZNF785, ZNF485, ZNF562, ZNF264, ZNF140, ZNF480, FBXW7, MED26, ZNF33B, PSMA4, DDX39B, CDK4, MTF2, ZNF613, ZNF558, ZNF777, INTS7, INTS8, RHNO1 |
| RNA Polymerase II Transcription | 28 | 1.42E-04 | ZNF551, PSMD11, CBFB, ICE2, ZNF2, TRIAP1, YY1, ZNF227, ZNF544, BID, ZNF785, ZNF485, ZNF562, ZNF264, ZNF140, ZNF480, FBXW7, MED26, ZNF33B, PSMA4, DDX39B, CDK4, ZNF613, ZNF558, ZNF777, INTS7, INTS8, RHNO1 |
| Generic Transcription Pathway | 24 | 0.001255689 | ZNF485, ZNF551, ZNF562, ZNF264, PSMD11, CBFB, ZNF140, ZNF480, FBXW7, ZNF2, TRIAP1, MED26, ZNF33B, YY1, PSMA4, CDK4, ZNF613, ZNF558, ZNF777, ZNF227, ZNF544, BID, ZNF785, RHNO1 |
| Metabolism of RNA | 15 | 0.004774349 | NOP56, SF3B4, DDX6, PSMD11, GPKOW, ANP32A, RPP40, RPSA, TFB1M, TRIT1, HNRNPL, PSMA4, DDX39B, TRMT12, RPS24 |
| Axon guidance | 13 | 0.006382222 | ARHGEF11, PSMD11, MYO10, LIMK2, LIMK1, RPSA, ROBO1, TUBB8, RPS6KA6, PSMA4, PLXNA1, SRGAP1, RPS24 |
| Nervous system development | 13 | 0.008955146 | ARHGEF11, PSMD11, MYO10, LIMK2, LIMK1, RPSA, ROBO1, TUBB8, RPS6KA6, PSMA4, PLXNA1, SRGAP1, RPS24 |

**Table S11.** Reactome pathway database results for purple module in NBMSC- PBC

| **Reactome pathway** | **Count** | **P Value** | **Genes** |
| --- | --- | --- | --- |
| Metabolic pathways | 34 | 0.0058 | COLGALT2, B4GALT1, INPP1, HSD17B4, DBH, FUT2, MAN2A1, NMRK1, MGAT5, ANPEP, ENPP2, ENPP6, HMGCLL1, ATP6V1D, HIBCH, CHST9, CERS4, GCH1, SRD5A2, IDH1, PLA2G4A, SGPP2, ASS1, CYP27A1, ALDH3A1, PHOSPHO2, PCCA, SCD, ALDH1A2, CAT, ALDH1A1, PIGF, ASPA, PLPP1 |

**Table S12.** Reactome pathway database results for red module in NBMSC- PBC

| **Reactome pathway** | **Count** | **P Value** | **Genes** |
| --- | --- | --- | --- |
| Diseases of metabolism | 8 | 2.96E-04 | RPIA, BCAN, GALE, G6PC3, MMAA, CYP11A1, THSD4, GNE |

**Table S13.** Reactome pathway database results for midnight blue module in PBC-RNIT

| **Reactome pathway** | **Count** | **PValue** | **Genes** |
| --- | --- | --- | --- |
| Ligand-receptor interactions | 3 | 0.005968 | SHH, PTCH1, HHIP |

**Table S14.** Reactome pathway database results for orange module in PBC-RNIT

| **Reactome pathway** | **Count** | **P Value** | **Genes** |
| --- | --- | --- | --- |
| Neddylation | 8 | 0.008355 | PSMD8, SOCS2, FBXW12, FBXO27, FBXO17, KEAP1, VHL, DDA1 |

**Table S15.** Reactome pathway database results for purple module in PBC-RNIT

| **Reactome pathway** | **Count** | **P Value** | **Genes** |
| --- | --- | --- | --- |
| Signaling by Interleukins | 12 | 1.21E-05 | MAP2K3, SOCS3, CEBPD, IL1B, CCL4, PELI1, CCL3, TAB3, MAP3K8, FOS, PTGS2, PTPN14 |
| Cytokine Signaling in Immune system | 14 | 3.10E-05 | MAP2K3, EGR1, CEBPD, FOS, PTGS2, PTPN14, SOCS3, IL1B, CCL4, PELI1, CCL3, TAB3, MAP3K8, KPNA1 |
| NGF-stimulated transcription | 5 | 3.82E-05 | EGR1, EGR2, FOSB, FOS, SGK1 |
| Signal Transduction | 26 | 1.68E-04 | PPP1R15A, TNFAIP3, RND3, THBS1, RND1, SOCS3, CCL4, CCL3, PIP5K1A, CD55, IER3, EGR1, DUSP5, EGR2, DUSP2, DUSP1, AXIN1, CSNK1E, FOS, TNFRSF10D, NR4A1, FOSB, TAB3, SGK1, PPARD, HBEGF |
| Nuclear Events (kinase and transcription factor activation) | 5 | 2.24E-04 | EGR1, EGR2, FOSB, FOS, SGK1 |
| Toll Like Receptor 5 (TLR5) Cascade | 5 | 7.95E-04 | MAP2K3, PELI1, TAB3, MAP3K8, FOS |
| Toll Like Receptor 10 (TLR10) Cascade | 5 | 7.95E-04 | MAP2K3, PELI1, TAB3, MAP3K8, FOS |
| MyD88 cascade initiated on plasma membrane | 5 | 7.95E-04 | MAP2K3, PELI1, TAB3, MAP3K8, FOS |
| TRAF6 mediated induction of NFkB and MAP kinases upon TLR7/8 or 9 activation | 5 | 0.001027 | MAP2K3, PELI1, TAB3, MAP3K8, FOS |
| MyD88 dependent cascade initiated on endosome | 5 | 0.001069 | MAP2K3, PELI1, TAB3, MAP3K8, FOS |
| Toll Like Receptor 7/8 (TLR7/8) Cascade | 5 | 0.001113 | MAP2K3, PELI1, TAB3, MAP3K8, FOS |
| Toll Like Receptor 9 (TLR9) Cascade | 5 | 0.001253 | MAP2K3, PELI1, TAB3, MAP3K8, FOS |
| MyD88:MAL(TIRAP) cascade initiated on plasma membrane | 5 | 0.001512 | MAP2K3, PELI1, TAB3, MAP3K8, FOS |
| Toll Like Receptor TLR6:TLR2 Cascade | 5 | 0.001512 | MAP2K3, PELI1, TAB3, MAP3K8, FOS |
| Interleukin-10 signaling | 4 | 0.001577 | IL1B, CCL4, CCL3, PTGS2 |
| Toll Like Receptor TLR1:TLR2 Cascade | 5 | 0.001685 | MAP2K3, PELI1, TAB3, MAP3K8, FOS |
| Toll Like Receptor 2 (TLR2) Cascade | 5 | 0.001685 | MAP2K3, PELI1, TAB3, MAP3K8, FOS |
| RNA Polymerase II Transcription | 16 | 0.001824 | PLK3, ZNF394, GADD45A, AXIN1, SLC2A3, FOS, KLF4, THBS1, ELL2, TNFRSF10D, NR4A2, LGALS3, NR4A1, SOCS3, SGK1, PPARD |
| Interleukin-4 and Interleukin-13 signaling | 5 | 0.001935 | SOCS3, CEBPD, IL1B, FOS, PTGS2 |
| Generic Transcription Pathway | 15 | 0.002122 | PLK3, ZNF394, GADD45A, AXIN1, SLC2A3, FOS, KLF4, THBS1, TNFRSF10D, NR4A2, LGALS3, NR4A1, SOCS3, SGK1, PPARD |
| Signaling by NTRK1 (TRKA) | 5 | 0.002434 | EGR1, EGR2, FOSB, FOS, SGK1 |
| MAP kinase activation | 4 | 0.00382 | MAP2K3, TAB3, MAP3K8, FOS |
| Signaling by NTRKs | 5 | 0.004221 | EGR1, EGR2, FOSB, FOS, SGK1 |
| Toll Like Receptor 4 (TLR4) Cascade | 5 | 0.004334 | MAP2K3, PELI1, TAB3, MAP3K8, FOS |
| Gene expression (Transcription) | 16 | 0.004774 | PLK3, ZNF394, GADD45A, AXIN1, SLC2A3, FOS, KLF4, THBS1, ELL2, TNFRSF10D, NR4A2, LGALS3, NR4A1, SOCS3, SGK1, PPARD |
| Circadian Clock | 4 | 0.004915 | NOCT, BHLHE40, SIK1, CSNK1E |
| Interleukin-1 family signaling | 5 | 0.005057 | IL1B, PELI1, TAB3, MAP3K8, PTPN14 |
| Interleukin-17 signaling | 4 | 0.005319 | MAP2K3, TAB3, MAP3K8, FOS |
| RAF-independent MAPK1/3 activation | 3 | 0.005655 | DUSP5, DUSP2, DUSP1 |
| Immune System | 19 | 0.005924 | MAP2K3, EGR1, DDX3X, CEBPD, TNFAIP3, SLC2A3, FOS, PTGS2, PTPN14, LGALS3, SOCS3, IL1B, CCL4, PELI1, CCL3, TAB3, MAP3K8, CD55, KPNA1 |
| Toll-like Receptor Cascades | 5 | 0.00737 | MAP2K3, PELI1, TAB3, MAP3K8, FOS |

**Table S16.** Reactome pathway database results for lightcyan module in NBMSC- RNIT

| **Reactome pathway** | **Count** | **P Value** | **Genes** |
| --- | --- | --- | --- |
| Gene expression (Transcription) | 31 | 4.55E-05 | ZNF551, PSMD11, CBFB, ICE2, ZNF2, TRIAP1, YY1, SAP30L, DROSHA, ZNF227, ZNF544, BID, ZNF785, ZNF485, ZNF562, ZNF264, ZNF140, ZNF480, FBXW7, MED26, ZNF33B, PSMA4, DDX39B, CDK4, MTF2, ZNF613, ZNF558, ZNF777, INTS7, INTS8, RHNO1 |
| RNA Polymerase II Transcription | 28 | 1.42E-04 | ZNF551, PSMD11, CBFB, ICE2, ZNF2, TRIAP1, YY1, ZNF227, ZNF544, BID, ZNF785, ZNF485, ZNF562, ZNF264, ZNF140, ZNF480, FBXW7, MED26, ZNF33B, PSMA4, DDX39B, CDK4, ZNF613, ZNF558, ZNF777, INTS7, INTS8, RHNO1 |
| Generic Transcription Pathway | 24 | 0.001256 | ZNF485, ZNF551, ZNF562, ZNF264, PSMD11, CBFB, ZNF140, ZNF480, FBXW7, ZNF2, TRIAP1, MED26, ZNF33B, YY1, PSMA4, CDK4, ZNF613, ZNF558, ZNF777, ZNF227, ZNF544, BID, ZNF785, RHNO1 |
| Metabolism of RNA | 15 | 0.004774 | NOP56, SF3B4, DDX6, PSMD11, GPKOW, ANP32A, RPP40, RPSA, TFB1M, TRIT1, HNRNPL, PSMA4, DDX39B, TRMT12, RPS24 |
| Axon guidance | 13 | 0.006382 | ARHGEF11, PSMD11, MYO10, LIMK2, LIMK1, RPSA, ROBO1, TUBB8, RPS6KA6, PSMA4, PLXNA1, SRGAP1, RPS24 |
| Nervous system development | 13 | 0.008955 | ARHGEF11, PSMD11, MYO10, LIMK2, LIMK1, RPSA, ROBO1, TUBB8, RPS6KA6, PSMA4, PLXNA1, SRGAP1, RPS24 |

**Table S17.** Reactome pathway database results for purple module in NBMSC- RNIT

| **Names** | **Total** | **Elements** | | | |
| --- | --- | --- | --- | --- | --- |
| NBMSC-PBC, NBMSC-RNIT, PBC-RNIT | 6 | PPARD | CSNK1E | ETV6 |  |
|  |  | CST4 | PTPN14 | ADRM1 |  |
| NBMSC-PBC, PBC-RNIT | 4 | KIF13B | THSD4 |  |  |
|  |  | IGFBP2 | PIFO |  |  |
| NBMSC-PBC, NBMSC-RNIT | 137 | EMP1 | INTS7 | AMMECR1 | CHD7 |
|  |  | RPS6KA6 | EIF2AK2 | ZBTB45 | DENR |
|  |  | MED18 | G3BP1 | CBFB | GADD45A |
|  |  | FAM189B | IER3 | TCHP | KPNA2 |
|  |  | YY1 | PPP1R15A | NR4A2 | CD55 |
|  |  | LGALSL | AXIN1 | SGK1 | KXD1 |
|  |  | PSMA4 | SLC25A13 | KIAA1199 | PLK3 |
|  |  | DUS4L | DYRK3 | CPOX | PTGS2 |
|  |  | BID | MAP3K8 | MORC4 | DUSP1 |
|  |  | ZNF785 | PDK1 | AUP1 | LRP5 |
|  |  | PPP2R5D | SLC13A2 | ZNF264 | MEGF8 |
|  |  | ZNF140 | EGR2 | TFB1M | GRPEL2 |
|  |  | C8orf4 | OCIAD1 | RPS24 | CCDC58 |
|  |  | MARK2 | MCMBP | HNRNPL | USB1 |
|  |  | DUSP5 | THBS1 | ACP2 | MAFF |
|  |  | DDX6 | DGAT2 | MAP2K3 | TNFAIP3 |
|  |  | DUSP2 | PELI1 | MRPL9 | SIK1 |
|  |  | ZNF480 | ATAD5 | VWA7 | C5orf30 |
|  |  | HN1 | SRGAP1 | E2F3 | TIMM8B |
|  |  | PSMD11 | UBE2H | CMTM4 | CLIC1 |
|  |  | LIMK1 | SLC16A3 | COA1 | RASD1 |
|  |  | LRRC8E | DDX3X | C12orf4 | TNFRSF10D |
|  |  | GADD45GIP1 | SRP19 | ABHD17C | KPNA1 |
| NBMSC-PBC | 126 | CPA6 | TMEM41B | ID3 | CAT |
|  |  | GSG1L | DLL1 | ZFP36 | CLCA4 |
|  |  | PCCA | SYNGR1 | TMEM132C | PTPRN2 |
|  |  | ZNF626 | COL13A1 | SERPINB5 | ASPA |
|  |  | ABCA8 | KLRD1 | PON3 | SPRR3 |
|  |  | RTN4 | ANPEP | EFCAB14 | AKAP7 |
|  |  | HMGCLL1 | ID2 | AIM1 | FABP4 |
|  |  | SALL1 | NME5 | NWD1 | SHH |
|  |  | MIA2 | ALDH1A2 | DLG2 | SOSTDC1 |
|  |  | SAP30L | HOXA9 | HHIP | SCD |
|  |  | MPPED2 | TRIM6 | GNRH1 | TOR4A |
|  |  | NAALAD2 | RPS6KA2 | LEAP2 | CIAPIN1 |
|  |  | SLC46A2 | ABCC4 | PLS1 | PDGFRA |
|  |  | KCNK2 | PHF14 | YPEL5 | LGMN |
|  |  | VCAM1 | ODC1 | PLA2G4A | RAD54B |
|  |  | RORB | CYSTM1 | TSPAN12 | HOXA2 |
|  |  | EHF | TMEM246 | ADAMTSL5 | TXK |
|  |  | ZDHHC2 | ZNF165 | COL6A5 | SOCS3 |
|  |  | LIMA1 | SLC39A6 | ZFYVE28 | SCARB2 |
|  |  | SWSAP1 | INTS8 | SLC35A1 | HSDL1 |
|  |  | KHDRBS2 | CRYZ | LAMA3 | TRPC6 |
| PBC-RNIT | 161 | ACTN4 | SAMD4B | ADAM17 | REG4 |
|  |  | HID1 | SESN2 | MED29 | SLX4 |
|  |  | MICALL1 | FUT11 | CYB561 | POU5F1 |
|  |  | MPDZ | IL5 | GPNMB | AIFM2 |
|  |  | SRCIN1 | FJX1 | KIAA1522 | TCP11L2 |
|  |  | FOXD2 | GIT2 | TMEM189 | TEX2 |
|  |  | SOCS2 | CDC6 | SMPD4 | ELF4 |
|  |  | PDAP1 | ZNF829 | HSD3B1 | SIX1 |
|  |  | HIST3H2A | CHEK2 | KRT8 | CNOT4 |
|  |  | AIFM1 | GDF11 | FAM102A | DUS1L |
|  |  | RALGAPA2 | SPTBN2 | KLF7 | CCNE1 |
|  |  | UQCR11 | XPO7 | TACR2 | DIEXF |
|  |  | FHDC1 | CHMP4B | RASSF8 | PTPRU |
|  |  | RNF186 | HCAR1 | CRCP | ERBB3 |
|  |  | AP2A1 | MED28 | PTRH2 | USP21 |
|  |  | POLR1C | VHL | STAT3 | SCAMP5 |
|  |  | SMG7 | KCTD5 | ZFP30 | EIF1 |
|  |  | NID1 | C1orf21 | MYCL | TMEM55B |
|  |  | SIRT7 | DNAJC3 | ZIC2 | KLHL36 |
|  |  | OR2W1 | TMEM161A | C6orf25 | RPL12 |
|  |  | TBC1D2B | TOM1L2 | NARF | CREG1 |
|  |  | QPCTL | PIP4K2C | SARS2 | SFT2D2 |
|  |  | POLR3A | MRPL27 | ZSCAN29 | STEAP3 |
|  |  | EXT1 | NTNG1 | PRICKLE3 | PAF1 |
|  |  | DEDD | XRN1 | ZNF691 | NLE1 |
|  |  | HAUS8 | CYP11B2 | EFTUD2 | SLC6A6 |
|  |  | EXO5 | ABHD2 | SWT1 | CDS1 |
| NBMSC-RNIT | 6 | MTF2 | SNX24 | FAM134C |  |
|  |  | CEBPD | ZNF777 | ZNF562 |  |

**Table S18.** The list of genes

| **Names** | **Total** | **elements** |
| --- | --- | --- |
| BMSC-PBC, BMSC-RNIT, PBC-RNIT | 6 | hsa-miR-93-5p, hsa-miR-16-5p,hsa-miR-92a-3p,hsa-miR-26b-5p,hsa-miR-335-5p,hsa-miR-17-5p |
| BMSC-RNIT, PBC-RNIT | 1 | hsa-miR-106b-5p |
| BMSC-PBC, BMSC-RNIT | 2 | hsa-miR-124-3p, hsa-let-7b-5p |
| PBC-RNIT | 3 | hsa-miR-20b-5p, hsa-miR-20a-5p, hsa-miR-24-3p |
| BMSC-RNIT | 1 | hsa-miR-98-5p |
| BMSC-PBC | 2 | hsa-miR-192-5p, hsa-miR-155-5p |

**Table S19.** The list of miRNAs.

| **Biological process** | **Count** | **p Value** | **Genes** |
| --- | --- | --- | --- |
| Response to estradiol | 7 | 0.00206531 | DUSP1, ALDH1A2, IGFBP2, CAT, BID, PTGS2, ASS1 |
| Negative regulation of osteoblast differentiation | 5 | 0.00236456 | ID2, LRP5, ID3, HOXA2, RORB |
| Peptidyl-tyrosine dephosphorylation | 7 | 0.00315605 | DUSP5, DUSP2, PTPRN2, DUSP1, EYA4, PTPN14, DUSP16 |
| Regulation of axonogenesis | 4 | 0.00427971 | CHN1, UST, KIF13B, MARK2 |
| Establishment of cell polarity | 4 | 0.00483939 | SHH, WEE1, UST, MARK2 |
| MAPK cascade | 11 | 0.00493065 | MAP2K3, PDGFRA, DUSP5, ZFP36, SPTBN5, PSMA4, PSMD11, FGF9, NLK, FGFR4, HBEGF |
| Inactivation of MAPK activity | 4 | 0.0054412 | DUSP5, DUSP2, DUSP1, DUSP16 |
| Cytokine production | 4 | 0.0054412 | FABP4, TXK, DBH, LIPA |
| Skeletal muscle cell differentiation | 5 | 0.0054431 | EGR1, NR4A1, EGR2, MAFF, FOS |
| Retina morphogenesis in camera-type eye | 3 | 0.00551339 | MAN2A1, LRP5, DLL1 |
| Peptidyl-tyrosine phosphorylation | 8 | 0.00693327 | MAP2K3, PDGFRA, WEE1, DYRK3, FGF9, EIF2AK2, FGFR4, HBEGF |
| Canonical Wnt signaling pathway | 6 | 0.00707952 | NR4A2, SHH, AXIN1, LRP5, FZD10, KLF4 |
| Response to testosterone | 4 | 0.0091092 | DUSP1, CDK4, GNRH1, THBS1 |
| Negative regulation of canonical Wnt signaling pathway | 8 | 0.00964628 | EGR1, SHH, PSMA4, PSMD11, G3BP1, IGFBP2, AXIN1, SOSTDC1 |
| Response to lipopolysaccharide | 8 | 0.00995506 | TRIM6, VCAM1, GCH1, PELI1, GNRH1, FOS, PTGS2, TNFRSF10D |

**Table S20.** The GO results for NBMSC- PBC subnetwork

| **Biological process** | **Count** | **p Value** | **Genes** |
| --- | --- | --- | --- |
| Positive regulation of transcription, DNA-templated | 13 | 0.00269 | USP21, STAT3, PDGFB, SIX1, KLF7, IL5, ZIC2, CCNE1, ELF4, CHEK2, TADA3, VHL, PPARD |
| Transcription, DNA-templated | 31 | 0.00281 | ZNF324B, CRCP, HKR1, WWC1, KEAP1, HOXA13, ZNF829, ZFP30, ZIC2, CHEK2, DEDD, CBX8, FOXD2, USP21, SWT1, STAT3, SAMD4B, PTPN14, POU5F1, MED29, NR2F6, GATAD2A, MED28, KLF7, POLR3A, PAX9, TADA3, POLR1C, HOXB7, ZSCAN29, PPARD |
| Cellular response to hormone stimulus | 4 | 0.00796 | SOCS2, CYP11B2, STAT3, IGFBP2 |
| Endocytosis | 6 | 0.00891 | MYO1E, MICALL1, AP2A1, EPS15L1, SNX11, CSNK1E |

**Table S21.** The GO results for PBC-RNIT subnetwork

| **biological process** | **Count** | **p Value** | **Genes** |
| --- | --- | --- | --- |
| Fat cell differentiation | 6 | 2.54E-04 | NR4A2, NR4A1, EGR2, CEBPD, ALMS1, KLF4 |
| Skeletal muscle cell differentiation | 5 | 5.55E-04 | EGR1, NR4A1, EGR2, MAFF, FOS |
| Protein phosphorylation | 12 | 8.39E-04 | PLK3, RPS6KA6, DYRK3, CDK4, LIMK1, EIF2AK2, SIK1, MAP3K8, CSNK1E, SGK1, MARK2, PDK1 |
| Translation | 8 | 0.003565328 | COA1, MRPL19, EIF2AK2, RPSA, MRPL9, HARS2, RPS24, SLC25A13 |
| Endoderm formation | 3 | 0.00373103 | DUSP5, DUSP2, DUSP1 |
| Mitotic cell cycle arrest | 3 | 0.004387122 | GADD45GIP1, DUSP1, GADD45A |
| Apoptotic process | 12 | 0.004586648 | TCHP, PPP1R15A, NR4A1, PLK3, GADD45A, AXIN1, C8ORF4, TNFAIP3, MAP3K8, SGK1, IER3, PPARD |
| Regulation of apoptotic process | 7 | 0.006298496 | EGR1, DUSP1, LRP5, EIF2AK2, BID, SGK1, TNFRSF10D |
| Transcription, DNA-templated | 26 | 0.00776221 | ZNF551, DDX3X, CEBPD, CHD7, ZBTB21, ZBTB45, TFB1M, ZNF280D, YY1, E2F3, ZNF785, ZNF562, ZNF264, EGR2, ZNF140, ZNF480, EIF2AK2, SNF8, PTPN14, NR4A2, NR4A1, KLF6, BHLHE40, ZNF558, ZNF777, PPARD |
| Positive regulation of translation | 4 | 0.008011304 | DDX3X, DDX39B, CDK4, THBS1 |

**Table S22.** The GO results for NBMSC-RNIT subnetwork

| **Reactome pathway** | **Count** | **P Value** | **Genes** |
| --- | --- | --- | --- |
| Nuclear Events (kinase and transcription factor activation) | 8 | 7.83E-05 | EGR1, EGR2, RPS6KA2, ID2, ID3, PPP2R5D, FOS, SGK1 |
| Signal Transduction | 66 | 3.02E-04 | B4GALT1, HHIP, TNFAIP3, PLAT, FZD10, RND3, DUSP16, YY1, SHH, FGF9, RPS6KA2, KPNA2, SH3GL2, IER3, PDK1, PDGFRA, DUSP5, DUSP2, TRPC6, DUSP1, FBXW7, AXIN1, PLA2G4A, PPP2R5D, CSNK1E, FOS, PSMA4, DDX39B, ALDH1A2, DSG2, COL6A5, SGK1, HBEGF, PPARD, PPP1R15A, SPTBN5, KHDRBS2, PSMD11, CBFB, LAMA3, LRP5, ABHD17C, NLK, THBS1, DLL1, RTN4, NCKIPSD, SOCS3, CHN1, PLXNA1, E2F3, SRGAP1, CD55, EGR1, EGR2, LIMK1, TNFRSF10D, NR4A1, DLG2, SCD, CDK4, ID2, ID3, F2RL1, GNRH1, FGFR4 |
| NGF-stimulated transcription | 6 | 5.07E-04 | EGR1, EGR2, ID2, ID3, FOS, SGK1 |
| Signaling by NTRK1 (TRKA) | 9 | 8.04E-04 | EGR1, EGR2, RPS6KA2, ID2, ID3, PPP2R5D, FOS, SGK1, SH3GL2 |
| RAF/MAP kinase cascade | 14 | 0.001074507 | PDGFRA, DUSP5, SPTBN5, DUSP2, PSMD11, DUSP1, PPP2R5D, ABHD17C, DUSP16, DLG2, PSMA4, FGF9, FGFR4, HBEGF |
| MAPK1/MAPK3 signaling | 14 | 0.001302526 | PDGFRA, DUSP5, SPTBN5, DUSP2, PSMD11, DUSP1, PPP2R5D, ABHD17C, DUSP16, DLG2, PSMA4, FGF9, FGFR4, HBEGF |
| Signaling by NTRKs | 9 | 0.002149921 | EGR1, EGR2, RPS6KA2, ID2, ID3, PPP2R5D, FOS, SGK1, SH3GL2 |
| MAPK family signaling cascades | 14 | 0.003977604 | PDGFRA, DUSP5, SPTBN5, DUSP2, PSMD11, DUSP1, PPP2R5D, ABHD17C, DUSP16, DLG2, PSMA4, FGF9, FGFR4, HBEGF |
| Negative regulation of MAPK pathway | 5 | 0.006163317 | DUSP5, DUSP2, DUSP1, PPP2R5D, DUSP16 |
| RAF-independent MAPK1/3 activation | 4 | 0.006853997 | DUSP5, DUSP2, DUSP1, DUSP16 |

**Table S23.** Reactome pathway analysis results for NBMSC- PBC subnetwork

| **Reactome pathway** | **Count** | **P Value** | **Genes** |
| --- | --- | --- | --- |
| RNA Polymerase II Transcription | 29 | 1.00E-05 | ZNF551, PSMD11, CBFB, SLC2A3, THBS1, YY1, BID, ZNF785, ZNF562, ZNF264, PLK3, ZNF140, ZNF480, GADD45A, FBXW7, AXIN1, FOS, KLF4, TNFRSF10D, NR4A2, NR4A1, PSMA4, DDX39B, CDK4, ZNF558, ZNF777, INTS7, SGK1, PPARD |
| Generic Transcription Pathway | 27 | 1.62E-05 | ZNF551, PSMD11, CBFB, SLC2A3, THBS1, YY1, BID, ZNF785, ZNF562, ZNF264, PLK3, ZNF140, ZNF480, GADD45A, FBXW7, AXIN1, FOS, KLF4, TNFRSF10D, NR4A2, NR4A1, PSMA4, CDK4, ZNF558, ZNF777, SGK1, PPARD |
| Gene expression (Transcription) | 30 | 2.22E-05 | ZNF551, PSMD11, CBFB, SLC2A3, THBS1, YY1, BID, ZNF785, ZNF562, ZNF264, PLK3, ZNF140, ZNF480, GADD45A, FBXW7, AXIN1, FOS, KLF4, TNFRSF10D, NR4A2, NR4A1, PSMA4, DDX39B, CDK4, MTF2, ZNF558, ZNF777, INTS7, SGK1, PPARD |
| Nuclear Events (kinase and transcription factor activation) | 5 | 0.00202 | EGR1, EGR2, PPP2R5D, FOS, SGK1 |
| Signal Transduction | 36 | 0.002894 | PPP1R15A, PSMD11, CBFB, LRP5, TNFAIP3, ABHD17C, RND3, THBS1, YY1, NCKIPSD, PLXNA1, E2F3, KPNA2, SRGAP1, CD55, IER3, PDK1, EGR1, DUSP5, EGR2, DUSP2, DUSP1, FBXW7, LIMK1, AXIN1, PPP2R5D, CSNK1E, FOS, TNFRSF10D, NR4A1, PSMA4, DDX39B, CDK4, SGK1, PPARD, HBEGF |
| NGF-stimulated transcription | 4 | 0.004833 | EGR1, EGR2, FOS, SGK1 |
| Negative regulation of MAPK pathway | 4 | 0.006363 | DUSP5, DUSP2, DUSP1, PPP2R5D |
| Transcriptional regulation of white adipocyte differentiation | 5 | 0.006407 | EGR2, CEBPD, CDK4, KLF4, MED18 |
| MyD88 cascade initiated on plasma membrane | 5 | 0.006678 | MAP2K3, PELI1, PPP2R5D, MAP3K8, FOS |
| Toll Like Receptor 10 (TLR10) Cascade | 5 | 0.006678 | MAP2K3, PELI1, PPP2R5D, MAP3K8, FOS |
| Toll Like Receptor 5 (TLR5) Cascade | 5 | 0.006678 | MAP2K3, PELI1, PPP2R5D, MAP3K8, FOS |
| TRAF6 mediated induction of NFkB and MAP kinases upon TLR7/8 or 9 activation | 5 | 0.00847 | MAP2K3, PELI1, PPP2R5D, MAP3K8, FOS |
| MyD88 dependent cascade initiated on endosome | 5 | 0.008797 | MAP2K3, PELI1, PPP2R5D, MAP3K8, FOS |
| Toll Like Receptor 7/8 (TLR7/8) Cascade | 5 | 0.009131 | MAP2K3, PELI1, PPP2R5D, MAP3K8, FOS |
| Cytokine Signaling in Immune system | 14 | 0.009753 | MAP2K3, EGR1, PSMD11, CEBPD, EIF2AK2, PPP2R5D, FOS, PTGS2, PTPN14, PSMA4, PELI1, MAP3K8, KPNA2, KPNA1 |

**Table S24.** Reactome pathway analysis results for NBMSC- RNIT subnetwork

| **Category** | **Term** | **Count** | **P-value** | **miRNA** |
| --- | --- | --- | --- | --- |
| Family | mir-124 family | 3 | 3.90E-07 | hsa-mir-124-1,hsa-mir-124-2,hsa-mir-124-3 |
| Family | mir-25 family | 2 | 3.43E-04 | hsa-mir-92a-1,hsa-mir-92a-2 |
| Family | mir-15 family | 2 | 5.69E-04 | hsa-mir-16-1,hsa-mir-16-2 |
| Family | mir-17 family | 2 | 1.57E-03 | hsa-mir-17,hsa-mir-93 |

**Table S25.** Family of miRNAs in NBMSC-PBC bipartite subnetwork

| **Function** | **Count** | **P-value** | **miRNA** |
| --- | --- | --- | --- |
| Cell Proliferation | 10 | 1.69E-11 | hsa-let-7b,hsa-mir-124-2,hsa-mir-17,hsa-mir-92a-1,hsa-mir-16-1,hsa-mir-124-1,hsa-mir-16-2,hsa-mir-124-3,hsa-mir-93,hsa-mir-92a-2 |
| Cell Cycle | 10 | 2.48E-11 | hsa-let-7b,hsa-mir-124-2,hsa-mir-17,hsa-mir-92a-1,hsa-mir-16-1,hsa-mir-124-1,hsa-mir-16-2,hsa-mir-124-3,hsa-mir-155,hsa-mir-92a-2 |
| Cell Division | 6 | 8.21E-10 | hsa-mir-124-2,hsa-let-7b,hsa-mir-124-1,hsa-mir-16-2,hsa-mir-124-3,hsa-mir-16-1 |
| Hormone-mediated Signaling Pathway | 8 | 2.04E-09 | hsa-mir-124-2,hsa-mir-17,hsa-mir-92a-1,hsa-mir-16-1,hsa-mir-124-1,hsa-mir-16-2,hsa-mir-124-3,hsa-mir-92a-2 |
| Regulation of Stem Cell | 8 | 2.60E-08 | hsa-mir-124-2,hsa-mir-26b,hsa-mir-17,hsa-mir-124-1,hsa-mir-124-3,hsa-mir-93,hsa-mir-155,hsa-mir-192 |
| Bone Regeneration | 6 | 3.74E-08 | hsa-let-7b,hsa-mir-17,hsa-mir-92a-1,hsa-mir-93,hsa-mir-92a-2,hsa-mir-155 |
| Cell Differentiation | 7 | 6.11E-08 | hsa-let-7b,hsa-mir-124-2,hsa-mir-16-1,hsa-mir-124-1,hsa-mir-16-2,hsa-mir-124-3,hsa-mir-155 |
| Latent Virus Replication | 5 | 8.02E-08 | hsa-mir-17,hsa-mir-92a-1,hsa-mir-93,hsa-mir-92a-2,hsa-mir-155 |
| Immune Response | 8 | 8.89E-08 | hsa-mir-92a-1,hsa-mir-16-2,hsa-mir-155,hsa-mir-16-1,hsa-mir-92a-2,hsa-mir-192,hsa-mir-93,hsa-mir-17 |
| Brain Development | 6 | 1.20E-07 | hsa-mir-124-2,hsa-mir-17,hsa-mir-124-1,hsa-mir-124-3,hsa-mir-155,hsa-mir-192 |
| Aging | 7 | 1.42E-07 | hsa-let-7b,hsa-mir-17,hsa-mir-92a-1,hsa-mir-16-1,hsa-mir-16-2,hsa-mir-155,hsa-mir-92a-2 |
| Tumor Suppressor MiRNAs | 7 | 1.78E-07 | hsa-let-7b,hsa-mir-124-2,hsa-mir-26b,hsa-mir-16-1,hsa-mir-124-1,hsa-mir-16-2,hsa-mir-124-3 |
| Angiogenesis | 7 | 1.78E-07 | hsa-let-7b,hsa-mir-17,hsa-mir-92a-1,hsa-mir-16-1,hsa-mir-16-2,hsa-mir-93,hsa-mir-92a-2 |
| Adipocyte Differentiation | 6 | 2.72E-07 | hsa-let-7b,hsa-mir-26b,hsa-mir-17,hsa-mir-92a-1,hsa-mir-92a-2,hsa-mir-192 |
| Inflammation | 8 | 4.26E-07 | hsa-mir-124-2,hsa-mir-124-3,hsa-mir-155,hsa-mir-335,hsa-mir-192,hsa-mir-93,hsa-mir-17,hsa-mir-124-1 |
| Cell Death | 7 | 6.44E-07 | hsa-let-7b,hsa-mir-17,hsa-mir-92a-1,hsa-mir-16-1,hsa-mir-16-2,hsa-mir-92a-2,hsa-mir-155 |
| Epithelial-to-Mesenchymal Transition | 7 | 9.94E-07 | hsa-let-7b,hsa-mir-124-2,hsa-mir-26b,hsa-mir-124-1,hsa-mir-124-3,hsa-mir-155,hsa-mir-192 |
| Osteoclast Differentiation | 4 | 2.31E-06 | hsa-mir-124-2,hsa-mir-124-1,hsa-mir-17,hsa-mir-124-3 |
| T-Cell Differentiation | 4 | 4.17E-06 | hsa-mir-16-1,hsa-let-7b,hsa-mir-16-2,hsa-mir-155 |
| Onco-MiRNAs | 5 | 5.19E-06 | hsa-mir-17,hsa-mir-92a-1,hsa-mir-93,hsa-mir-92a-2,hsa-mir-155 |
| Apoptosis | 7 | 5.37E-06 | hsa-mir-92a-1,hsa-mir-16-2,hsa-mir-155,hsa-mir-16-1,hsa-mir-92a-2,hsa-mir-26b,hsa-mir-17 |
| Embryonic Development | 4 | 5.42E-06 | hsa-mir-124-2,hsa-mir-124-1,hsa-mir-124-3,hsa-mir-93 |
| Neurotoxicity | 4 | 1.09E-05 | hsa-mir-16-2,hsa-mir-92a-1,hsa-mir-92a-2,hsa-mir-16-1 |
| Peritoneal Cavity Homeostasis(26495316) | 4 | 1.96E-05 | hsa-mir-26b,hsa-mir-335,hsa-mir-93,hsa-mir-192 |
| Regulation of Akt Pathway | 4 | 3.27E-05 | hsa-mir-17,hsa-mir-92a-1,hsa-mir-92a-2,hsa-mir-155 |
| Glucose Metabolism | 4 | 4.44E-05 | hsa-let-7b,hsa-mir-124-2,hsa-mir-124-1,hsa-mir-124-3 |
| Hematopoiesis | 5 | 4.57E-05 | hsa-let-7b,hsa-mir-17,hsa-mir-92a-1,hsa-mir-92a-2,hsa-mir-155 |
| Neuron Differentiation | 3 | 1.35E-04 | hsa-mir-124-2,hsa-mir-124-1,hsa-mir-124-3 |
| Neuron Apoptosis | 3 | 1.68E-04 | hsa-mir-124-2,hsa-mir-124-1,hsa-mir-124-3 |
| Smooth Muscle Cell Proliferation | 3 | 2.97E-04 | hsa-mir-124-2,hsa-mir-124-1,hsa-mir-124-3 |
| Lipid Metabolism | 4 | 2.99E-04 | hsa-mir-92a-1,hsa-mir-335,hsa-mir-92a-2,hsa-mir-192 |
| Muscle Development | 3 | 4.77E-04 | hsa-mir-124-2,hsa-mir-124-1,hsa-mir-124-3 |
| Immune System(Xiao's Cell2010) | 3 | 4.77E-04 | hsa-mir-17,hsa-mir-93,hsa-mir-155 |
| Circadian Rhythm | 3 | 5.49E-04 | hsa-mir-16-1,hsa-mir-16-2,hsa-mir-192 |
| Osteogenesis | 4 | 8.56E-04 | hsa-mir-124-2,hsa-mir-17,hsa-mir-124-1,hsa-mir-124-3 |
| Toxicity | 3 | 2.39E-03 | hsa-mir-16-1,hsa-mir-16-2,hsa-mir-192 |
| Granulopoiesis | 2 | 2.50E-03 | hsa-mir-17,hsa-mir-155 |
| Chondrocyte Development | 2 | 5.71E-03 | hsa-mir-92a-1,hsa-mir-92a-2 |
| DNA Damage Response | 2 | 6.50E-03 | hsa-mir-16-2,hsa-mir-16-1 |
| T-helper 17 Cell Differentiation | 2 | 9.13E-03 | hsa-mir-93,hsa-mir-155 |

**Table S26.** Enrichment analysis of miRNAs in NBMSC-PBC bipartite subnetwork

| **Family** | **Count** | **P-value** | **miRNA** |
| --- | --- | --- | --- |
| mir-124 family | 3 | 3.90E-07 | hsa-mir-124-1,hsa-mir-124-2,hsa-mir-124-3 |
| mir-17 family | 3 | 2.13E-05 | hsa-mir-106b,hsa-mir-17,hsa-mir-93 |
| mir-25 family | 2 | 3.43E-04 | hsa-mir-92a-1,hsa-mir-92a-2 |
| mir-15 family | 2 | 5.69E-04 | hsa-mir-16-1,hsa-mir-16-2 |
| let-7 family | 2 | 3.64E-03 | hsa-let-7b,hsa-mir-98 |

**Table S27.** Family of miRNAs in NBMSC-RNIT bipartite subnetwork

| **Term** | **Count** | **P-value** | **miRNA** |
| --- | --- | --- | --- |
| Cell Division | 7 | 5.88E-12 | hsa-mir-124-2,hsa-mir-106b,hsa-let-7b,hsa-mir-124-1,hsa-mir-16-2,hsa-mir-124-3,hsa-mir-16-1 |
| Cell Proliferation | 10 | 1.69E-11 | hsa-let-7b,hsa-mir-124-2,hsa-mir-17,hsa-mir-92a-1,hsa-mir-16-1,hsa-mir-124-1,hsa-mir-16-2,hsa-mir-124-3,hsa-mir-93,hsa-mir-92a-2 |
| Cell Cycle | 10 | 2.48E-11 | hsa-let-7b,hsa-mir-124-2,hsa-mir-17,hsa-mir-92a-1,hsa-mir-98,hsa-mir-16-1,hsa-mir-124-1,hsa-mir-16-2,hsa-mir-124-3,hsa-mir-92a-2 |
| Hormone-mediated Signaling Pathway | 9 | 3.94E-11 | hsa-mir-124-2,hsa-mir-17,hsa-mir-92a-1,hsa-mir-98,hsa-mir-16-1,hsa-mir-124-1,hsa-mir-16-2,hsa-mir-124-3,hsa-mir-92a-2 |
| Tumor Suppressor MiRNAs | 8 | 5.25E-09 | hsa-let-7b,hsa-mir-124-2,hsa-mir-26b,hsa-mir-98,hsa-mir-16-1,hsa-mir-124-1,hsa-mir-16-2,hsa-mir-124-3 |
| Latent Virus Replication | 5 | 8.02E-08 | hsa-mir-106b,hsa-mir-17,hsa-mir-92a-1,hsa-mir-93,hsa-mir-92a-2 |
| Angiogenesis | 7 | 1.78E-07 | hsa-let-7b,hsa-mir-17,hsa-mir-92a-1,hsa-mir-16-1,hsa-mir-16-2,hsa-mir-93,hsa-mir-92a-2 |
| Bile Duct Proliferation | 3 | 3.90E-07 | hsa-mir-124-2,hsa-mir-124-1,hsa-mir-124-3 |
| Cell Death | 7 | 6.44E-07 | hsa-let-7b,hsa-mir-17,hsa-mir-92a-1,hsa-mir-98,hsa-mir-16-1,hsa-mir-16-2,hsa-mir-92a-2 |
| Regulation of Stem Cell | 7 | 7.04E-07 | hsa-mir-124-2,hsa-mir-106b,hsa-mir-26b,hsa-mir-17,hsa-mir-124-1,hsa-mir-124-3,hsa-mir-93 |
| Bone Regeneration | 5 | 1.75E-06 | hsa-let-7b,hsa-mir-17,hsa-mir-92a-1,hsa-mir-93,hsa-mir-92a-2 |
| Cell Differentiation | 6 | 1.85E-06 | hsa-let-7b,hsa-mir-124-2,hsa-mir-16-1,hsa-mir-124-1,hsa-mir-16-2,hsa-mir-124-3 |
| Immune Response | 7 | 2.03E-06 | hsa-mir-92a-1,hsa-mir-16-2,hsa-mir-16-1,hsa-mir-92a-2,hsa-mir-98,hsa-mir-93,hsa-mir-17 |
| Osteoclast Differentiation | 4 | 2.31E-06 | hsa-mir-124-2,hsa-mir-124-1,hsa-mir-17,hsa-mir-124-3 |
| Aging | 6 | 3.76E-06 | hsa-let-7b,hsa-mir-17,hsa-mir-92a-1,hsa-mir-16-1,hsa-mir-16-2,hsa-mir-92a-2 |
| Brain Development | 5 | 4.50E-06 | hsa-mir-124-2,hsa-mir-106b,hsa-mir-17,hsa-mir-124-1,hsa-mir-124-3 |
| Onco-MiRNAs | 5 | 5.19E-06 | hsa-mir-106b,hsa-mir-17,hsa-mir-92a-1,hsa-mir-93,hsa-mir-92a-2 |
| Apoptosis | 7 | 5.37E-06 | hsa-mir-92a-1,hsa-mir-16-2,hsa-mir-16-1,hsa-mir-92a-2,hsa-mir-26b,hsa-mir-98,hsa-mir-17 |
| Embryonic Development | 4 | 5.42E-06 | hsa-mir-124-2,hsa-mir-124-1,hsa-mir-124-3,hsa-mir-93 |
| Regulation of Wnt Signaling Pathway | 3 | 7.69E-06 | hsa-mir-124-2,hsa-mir-124-1,hsa-mir-124-3 |
| Inflammation | 7 | 7.81E-06 | hsa-mir-124-2,hsa-mir-124-3,hsa-mir-335,hsa-mir-98,hsa-mir-93,hsa-mir-17,hsa-mir-124-1 |
| Adipocyte Differentiation | 5 | 8.76E-06 | hsa-let-7b,hsa-mir-26b,hsa-mir-17,hsa-mir-92a-1,hsa-mir-92a-2 |
| Neurotoxicity | 4 | 1.09E-05 | hsa-mir-16-2,hsa-mir-92a-1,hsa-mir-92a-2,hsa-mir-16-1 |
| Cholesterol Metabolism | 3 | 2.13E-05 | hsa-mir-92a-1,hsa-mir-98,hsa-mir-92a-2 |
| Transdifferentiation | 3 | 2.13E-05 | hsa-mir-124-2,hsa-mir-124-1,hsa-mir-124-3 |
| Glucose Metabolism | 4 | 4.44E-05 | hsa-let-7b,hsa-mir-124-2,hsa-mir-124-1,hsa-mir-124-3 |
| Osteogenesis | 5 | 5.42E-05 | hsa-mir-124-2,hsa-mir-17,hsa-mir-98,hsa-mir-124-1,hsa-mir-124-3 |
| Neuron Differentiation | 3 | 1.35E-04 | hsa-mir-124-2,hsa-mir-124-1,hsa-mir-124-3 |
| Neuron Apoptosis | 3 | 1.68E-04 | hsa-mir-124-2,hsa-mir-124-1,hsa-mir-124-3 |
| T-Cell Differentiation | 3 | 2.05E-04 | hsa-mir-16-1,hsa-let-7b,hsa-mir-16-2 |
| Epithelial-to-Mesenchymal Transition | 5 | 2.83E-04 | hsa-let-7b,hsa-mir-124-2,hsa-mir-26b,hsa-mir-124-1,hsa-mir-124-3 |
| Smooth Muscle Cell Proliferation | 3 | 2.97E-04 | hsa-mir-124-2,hsa-mir-124-1,hsa-mir-124-3 |
| Lipid Metabolism | 4 | 2.99E-04 | hsa-mir-92a-1,hsa-mir-98,hsa-mir-335,hsa-mir-92a-2 |
| Immune System(Xiao's Cell2010) | 3 | 4.77E-04 | hsa-mir-106b,hsa-mir-17,hsa-mir-93 |
| Muscle Development | 3 | 4.77E-04 | hsa-mir-124-2,hsa-mir-124-1,hsa-mir-124-3 |
| Peritoneal Cavity Homeostasis(26495316) | 3 | 6.29E-04 | hsa-mir-26b,hsa-mir-335,hsa-mir-93 |
| Hematopoiesis | 4 | 7.50E-04 | hsa-let-7b,hsa-mir-17,hsa-mir-92a-1,hsa-mir-92a-2 |
| Regulation of Akt Pathway | 3 | 9.10E-04 | hsa-mir-17,hsa-mir-92a-1,hsa-mir-92a-2 |
| Anti-Cell Proliferation(Hwang Etal Bjc2007) | 2 | 1.57E-03 | hsa-let-7b,hsa-mir-98 |
| Chemosensitivity Of Tumor Cells | 2 | 1.57E-03 | hsa-mir-16-1,hsa-mir-16-2 |
| Chondrocyte Development | 2 | 5.71E-03 | hsa-mir-92a-1,hsa-mir-92a-2 |
| DNA Damage Response | 2 | 6.50E-03 | hsa-mir-16-2,hsa-mir-16-1 |

**Table S28.** Enrichment analysis of miRNAs in NBMSC-RNIT bipartite subnetwork

| **Family** | **Count** | **P-value** | **miRNA** |
| --- | --- | --- | --- |
| mir-17 family | 5 | 4.85e-10 | hsa-mir-106b,hsa-mir-17,hsa-mir-20a,hsa-mir-20b,hsa-mir-93 |
| mir-24 family | 2 | 4.94e-5 | hsa-mir-24-1,hsa-mir-24-2 |
| mir-25 family | 2 | 2.94e-4 | hsa-mir-92a-1,hsa-mir-92a-2 |
| mir-15 family | 2 | 4.88e-4 | hsa-mir-16-1,hsa-mir-16-2 |
| mir-26 family | 1 | 0.0218 | hsa-mir-26b |

**Table S29.** Family of miRNAs in PBC-RNIT bipartite subnetwork

| **Function** | **Count** | **P-value** | **miRNA** |
| --- | --- | --- | --- |
| Onco-MiRNAs | 9 | 1.76e-13 | hsa-mir-24-1,hsa-mir-20a,hsa-mir-106b,hsa-mir-17,hsa-mir-20b,hsa-mir-92a-1,hsa-mir-24-2,hsa-mir-93,hsa-mir-92a-2 |
| Bone Regeneration | 7 | 2.97e-10 | hsa-mir-24-1,hsa-mir-17,hsa-mir-20b,hsa-mir-92a-1,hsa-mir-24-2,hsa-mir-93,hsa-mir-92a-2 |
| Latent Virus Replication | 6 | 4.72e-10 | hsa-mir-106b,hsa-mir-20a,hsa-mir-17,hsa-mir-92a-1,hsa-mir-93,hsa-mir-92a-2 |
| Hormone-mediated Signaling Pathway | 8 | 8.95e-10 | hsa-mir-24-1,hsa-mir-20a,hsa-mir-17,hsa-mir-92a-1,hsa-mir-16-1,hsa-mir-16-2,hsa-mir-24-2,hsa-mir-92a-2 |
| Apoptosis | 9 | 4.02e-9 | hsa-mir-24-1,hsa-mir-92a-1,hsa-mir-16-2,hsa-mir-16-1,hsa-mir-92a-2,hsa-mir-26b,hsa-mir-20a,hsa-mir-17,hsa-mir-24-2 |
| Cell Death | 8 | 1.04e-8 | hsa-mir-24-1,hsa-mir-20a,hsa-mir-17,hsa-mir-92a-1,hsa-mir-16-1,hsa-mir-16-2,hsa-mir-24-2,hsa-mir-92a-2 |
| Cell Proliferation | 8 | 1.28e-8 | hsa-mir-24-1,hsa-mir-17,hsa-mir-92a-1,hsa-mir-16-1,hsa-mir-16-2,hsa-mir-24-2,hsa-mir-93,hsa-mir-92a-2 |
| Cell Cycle | 8 | 1.73e-8 | hsa-mir-24-1,hsa-mir-20a,hsa-mir-17,hsa-mir-92a-1,hsa-mir-16-1,hsa-mir-16-2,hsa-mir-24-2,hsa-mir-92a-2 |
| Angiogenesis | 7 | 9.15e-8 | hsa-mir-20a,hsa-mir-17,hsa-mir-92a-1,hsa-mir-16-1,hsa-mir-16-2,hsa-mir-93,hsa-mir-92a-2 |
| Immune System(Xiao's Cell2010) | 5 | 1.68e-7 | hsa-mir-106b,hsa-mir-17,hsa-mir-20b,hsa-mir-93,hsa-mir-20a |
| Regulation of Stem Cell | 7 | 3.65e-7 | hsa-mir-24-1,hsa-mir-20a,hsa-mir-106b,hsa-mir-26b,hsa-mir-17,hsa-mir-24-2,hsa-mir-93 |
| Immune Response | 7 | 1.06e-6 | hsa-mir-92a-1,hsa-mir-16-2,hsa-mir-16-1,hsa-mir-92a-2,hsa-mir-93,hsa-mir-20a,hsa-mir-17 |
| Vascular Inflammation | 4 | 2.25e-6 | hsa-mir-24-1,hsa-mir-17,hsa-mir-24-2,hsa-mir-20a |
| Inflammation | 7 | 4.12e-6 | hsa-mir-24-1,hsa-mir-20b,hsa-mir-335,hsa-mir-93,hsa-mir-20a,hsa-mir-17,hsa-mir-24-2 |
| Adipocyte Differentiation | 5 | 5.73e-6 | hsa-mir-20a,hsa-mir-26b,hsa-mir-17,hsa-mir-92a-1,hsa-mir-92a-2 |
| Neurotoxicity | 4 | 7.84e-6 | hsa-mir-16-2,hsa-mir-92a-1,hsa-mir-92a-2,hsa-mir-16-1 |
| Regulation of Akt Pathway | 4 | 2.36e-5 | hsa-mir-20a,hsa-mir-17,hsa-mir-92a-1,hsa-mir-92a-2 |
| Hematopoiesis | 5 | 3.01e-5 | hsa-mir-20a,hsa-mir-17,hsa-mir-20b,hsa-mir-92a-1,hsa-mir-92a-2 |
| Embryonic Stem Cell Differentiation | 4 | 4.87e-5 | hsa-mir-24-1,hsa-mir-92a-1,hsa-mir-24-2,hsa-mir-92a-2 |
| Aging | 5 | 4.94e-5 | hsa-mir-17,hsa-mir-92a-1,hsa-mir-16-1,hsa-mir-16-2,hsa-mir-92a-2 |
| Muscle Regeneration | 2 | 4.94e-5 | hsa-mir-17,hsa-mir-20a |
| Cell Division | 3 | 1.96e-4 | hsa-mir-106b,hsa-mir-16-2,hsa-mir-16-1 |
| T-helper 17 Cell Differentiation | 3 | 2.77e-4 | hsa-mir-20b,hsa-mir-93,hsa-mir-20a |
| Cell Proliferation(Hwang Etal Bjc2007) | 2 | 2.94e-4 | hsa-mir-17,hsa-mir-20a |
| Adipogenesis | 3 | 3.25e-4 | hsa-mir-24-1,hsa-mir-26b,hsa-mir-24-2 |
| Peritoneal Cavity Homeostasis(26495316) | 3 | 4.98e-4 | hsa-mir-26b,hsa-mir-335,hsa-mir-93 |
| Chemosensitivity Of Tumor Cells | 2 | 1.35e-3 | hsa-mir-16-1,hsa-mir-16-2 |
| Cholesterol Metabolism | 2 | 1.35e-3 | hsa-mir-92a-1,hsa-mir-92a-2 |
| Myogensis | 2 | 1.35e-3 | hsa-mir-17,hsa-mir-20a |
| Brain Development | 3 | 1.90e-3 | hsa-mir-20a,hsa-mir-106b,hsa-mir-17 |
| Vascular Homeostasis | 2 | 3.13e-3 | hsa-mir-24-1,hsa-mir-24-2 |
| Lipid Metabolism | 3 | 3.64e-3 | hsa-mir-92a-1,hsa-mir-335,hsa-mir-92a-2 |
| Osteoclast Differentiation | 2 | 4.28e-3 | hsa-mir-17,hsa-mir-20a |
| Chondrocyte Development | 2 | 4.92e-3 | hsa-mir-92a-1,hsa-mir-92a-2 |
| Neuron Apoptosis | 2 | 4.92e-3 | hsa-mir-24-1,hsa-mir-24-2 |
| DNA Damage Response | 2 | 5.60e-3 | hsa-mir-16-2,hsa-mir-16-1 |
| T-Cell Differentiation | 2 | 5.60e-3 | hsa-mir-16-1,hsa-mir-16-2 |
| Embryonic Development | 2 | 6.32e-3 | hsa-mir-20b,hsa-mir-93 |
| DNA Damage Repair | 2 | 7.88e-3 | hsa-mir-24-1,hsa-mir-24-2 |
| Muscle Development | 2 | 9.60e-3 | hsa-mir-24-1,hsa-mir-24-2 |
| Tumor Suppressor MiRNAs | 3 | 0.0103 | hsa-mir-26b,hsa-mir-16-1,hsa-mir-16-2 |
| Circadian Rhythm | 2 | 0.0105 | hsa-mir-16-1,hsa-mir-16-2 |
| Skeletal Muscle Cell Differentiation | 2 | 0.0105 | hsa-mir-20a,hsa-mir-26b |

**Table S30.** Enrichment analysis of miRNAs in PBC-RNIT bipartite subnetwork

| **Gene.symbol** | **logFC** | **Gene.title** |
| --- | --- | --- |
| IGF2 | -1.48602108 | insulin like growth factor 2 |
| CCR7 | -1.18536495 | C-C motif chemokine receptor 7 |
| RN7SK | -1.04929117 | RNA, 7SK small nuclear |
| FBXO27 | -1.03924374 | F-box protein 27 |
| SYNE4 | -0.92741765 | spectrin repeat containing nuclear envelope family member 4 |
| FCRLB | -0.90984453 | Fc receptor like B |
| MYEOV | -0.87857444 | myeloma overexpressed |
| DEFB1 | -0.83615283 | defensin beta 1 |
| MYCL | -0.81464032 | v-myc avian myelocytomatosis viral oncogene lung carcinoma derived homolog |
| ATP6V1B1 | -0.80703821 | ATPase H+ transporting V1 subunit B1 |
| GALR2 | -0.74195784 | galanin receptor 2 |
| CKMT1A | -0.73508037 | creatine kinase, mitochondrial 1A |
| GPNMB | -0.71186561 | glycoprotein nmb |
| CKMT1B | -0.70725692 | creatine kinase, mitochondrial 1B |
| CEBPA | -0.68402899 | CCAAT/enhancer binding protein alpha |
| RGS5 | -0.68168643 | regulator of G-protein signaling 5 |
| KRT7 | -0.66768906 | keratin 7 |
| APLNR | -0.66282213 | apelin receptor |
| PLCXD1 | -0.64842688 | phosphatidylinositol specific phospholipase C X domain containing 1 |
| MYH11 | -0.64566008 | myosin heavy chain 11 |
| BICDL1 | -0.62676943 | BICD family like cargo adaptor 1 |
| CCNB2 | -0.62200264 | cyclin B2 |
| COL3A1 | -0.62060606 | collagen type III alpha 1 chain |
| HCAR1 | -0.61311462 | hydroxycarboxylic acid receptor 1 |
| SLC1A6 | -0.60522266 | solute carrier family 1 member 6 |
| PLAT | -0.60189723 | plasminogen activator, tissue type |
| PRC1 | -0.59662451 | protein regulator of cytokinesis 1 |
| TACC3 | -0.59150725 | transforming acidic coiled-coil containing protein 3 |
| MYLK4 | -0.59065613 | myosin light chain kinase family member 4 |
| HCLS1 | -0.58817655 | hematopoietic cell-specific Lyn substrate 1 |
| CDC25A | -0.58545982 | cell division cycle 25A |
| FASN | -0.58238472 | fatty acid synthase |
| CSTA | -0.57804743 | cystatin A |
| ACTA2 | -0.57402372 | actin, alpha 2, smooth muscle, aorta |
| FAM129A | -0.57225823 | family with sequence similarity 129 member A |
| TOP2A | -0.56360738 | topoisomerase (DNA) II alpha |
| RPSA | -0.56335968 | ribosomal protein SA |
| PDGFRB | -0.55216074 | platelet derived growth factor receptor beta |
| ESPL1 | -0.55195784 | extra spindle pole bodies like 1, separase |
| RNFT2 | -0.55126746 | ring finger protein, transmembrane 2 |
| TROAP | -0.54705138 | trophinin associated protein |
| PARPBP | -0.54474045 | PARP1 binding protein |
| FAM162B | -0.54271673 | family with sequence similarity 162 member B |
| CDT1 | -0.54134387 | chromatin licensing and DNA replication factor 1 |
| CALML3 | -0.5404137 | calmodulin like 3 |
| AMH | -0.53932543 | anti-Mullerian hormone |
| CCL19 | -0.53713834 | C-C motif chemokine ligand 19 |
| TTK | -0.5347668 | TTK protein kinase |
| ASF1B | -0.5320975 | anti-silencing function 1B histone chaperone |
| ACTG2 | -0.5286166 | actin, gamma 2, smooth muscle, enteric |
| SARS2 | -0.52431884 | seryl-tRNA synthetase 2, mitochondrial |
| NECTIN4 | -0.52357049 | nectin cell adhesion molecule 4 |
| PPIH | -0.52089328 | peptidylprolyl isomerase H |
| WASH1 | -0.52052174 | WAS protein family homolog 1 |
| WASH1 | -0.52052174 | WAS protein family homolog 1 |
| CD19 | -0.51395784 | CD19 molecule |
| PTRHD1 | -0.51368906 | peptidyl-tRNA hydrolase domain containing 1 |
| AURKA | -0.51204216 | aurora kinase A |
| CERS3 | -0.50797892 | ceramide synthase 3 |
| CDCA3 | -0.50033992 | cell division cycle associated 3 |
| ZFP30 | -0.49934387 | ZFP30 zinc finger protein |
| FRZB | -0.49764427 | frizzled-related protein |
| SELP | -0.49143874 | selectin P |
| COX4I2 | -0.48999473 | cytochrome c oxidase subunit 4I2 |
| TPX2 | -0.48919368 | TPX2, microtubule nucleation factor |
| MRPS12 | -0.48816601 | mitochondrial ribosomal protein S12 |
| GPX7 | -0.48738867 | glutathione peroxidase 7 |
| CENPF | -0.48638999 | centromere protein F |
| POTEJ | -0.48415283 | POTE ankyrin domain family member J |
| C10orf10 | -0.48386561 | chromosome 10 open reading frame 10 |
| RAMP2 | -0.48322266 | receptor activity modifying protein 2 |
| NCAPD2 | -0.48128063 | non-SMC condensin I complex subunit D2 |
| FCN3 | -0.47856653 | ficolin 3 |
| NOP56 | -0.47820817 | NOP56 ribonucleoprotein |
| NOP56 | -0.47820817 | NOP56 ribonucleoprotein |
| COL1A1 | -0.47634783 | collagen type I alpha 1 chain |
| S100A14 | -0.47527009 | S100 calcium binding protein A14 |
| SLC4A11 | -0.47189987 | solute carrier family 4 member 11 |
| SOX21 | -0.47101713 | SRY-box 21 |
| MAP4K1 | -0.46755204 | mitogen-activated protein kinase kinase kinase kinase 1 |
| RAPGEF3 | -0.4666693 | Rap guanine nucleotide exchange factor 3 |
| FANCI | -0.4665112 | Fanconi anemia complementation group I |
| CENPM | -0.46497233 | centromere protein M |
| SPARC | -0.46254809 | secreted protein acidic and cysteine rich |
| PAK4 | -0.45673781 | p21 (RAC1) activated kinase 4 |
| C22orf29 | -0.45621607 | chromosome 22 open reading frame 29 |
| PAFAH1B3 | -0.45600527 | platelet activating factor acetylhydrolase 1b catalytic subunit 3 |
| MYT1 | -0.4552253 | myelin transcription factor 1 |
| FAM83A | -0.45199209 | family with sequence similarity 83 member A |
| CDK1 | -0.45065086 | cyclin dependent kinase 1 |
| C1orf112 | -0.45060079 | chromosome 1 open reading frame 112 |
| TRIT1 | -0.44852174 | tRNA isopentenyltransferase 1 |
| ADCY4 | -0.44802899 | adenylate cyclase 4 |
| CCNB1 | -0.44498287 | cyclin B1 |
| DTX1 | -0.44347826 | deltex E3 ubiquitin ligase 1 |
| SDC1 | -0.44315415 | syndecan 1 |
| POLQ | -0.44267721 | DNA polymerase theta |
| USF1 | -0.43657708 | upstream transcription factor 1 |
| EPHA1 | -0.43640316 | EPH receptor A1 |
| GINS2 | -0.42684321 | GINS complex subunit 2 |
| CDCA5 | -0.42570751 | cell division cycle associated 5 |
| PLAC9 | -0.42509618 | placenta specific 9 |
| FER1L4 | -0.42269038 | fer-1 like family member 4, pseudogene |
| GPX3 | -0.4226614 | glutathione peroxidase 3 |
| HIGD1B | -0.42153887 | HIG1 hypoxia inducible domain family member 1B |
| CD248 | -0.41936495 | CD248 molecule |
| TIMELESS | -0.41875099 | timeless circadian clock |
| EVPL | -0.41813439 | envoplakin |
| FOXS1 | -0.41709354 | forkhead box S1 |
| CDC45 | -0.41669302 | cell division cycle 45 |
| SAPCD2 | -0.41658235 | suppressor APC domain containing 2 |
| UPK3A | -0.4162029 | uroplakin 3A |
| ERBB3 | -0.41467194 | erb-b2 receptor tyrosine kinase 3 |
| ADM5 | -0.41367325 | adrenomedullin 5 (putative) |
| MCM2 | -0.41314625 | minichromosome maintenance complex component 2 |
| NUSAP1 | -0.41289328 | nucleolar and spindle associated protein 1 |
| CENPO | -0.41285112 | centromere protein O |
| APOD | -0.41254282 | apolipoprotein D |
| TK1 | -0.41191831 | thymidine kinase 1 |
| BLM | -0.41183399 | Bloom syndrome RecQ like helicase |
| TPI1P2 | -0.41164163 | triosephosphate isomerase 1 pseudogene 2 |
| CDK12 | -0.41065876 | cyclin dependent kinase 12 |
| OXCT2 | -0.40902767 | 3-oxoacid CoA-transferase 2 |
| PAF1 | -0.40821344 | PAF1 homolog, Paf1/RNA polymerase II complex component |
| E2F2 | -0.40782872 | E2F transcription factor 2 |
| CDC20 | -0.4057365 | cell division cycle 20 |
| HOXC4 | -0.40527009 | homeobox C4 |
| MSH6 | -0.40453228 | mutS homolog 6 |
| RPS15 | -0.40332016 | ribosomal protein S15 |
| RASL10A | -0.40260079 | RAS like family 10 member A |
| AUNIP | -0.40088011 | aurora kinase A and ninein interacting protein |
| YIF1B | -0.40033729 | Yip1 interacting factor homolog B, membrane trafficking protein |
| ITLN2 | -0.39927536 | intelectin 2 |
| FOXD2 | -0.39853491 | forkhead box D2 |
| NCAPG | -0.39842424 | non-SMC condensin I complex subunit G |
| C1QTNF5 | -0.39801581 | C1q and tumor necrosis factor related protein 5 |
| LINGO1 | -0.39602372 | leucine rich repeat and Ig domain containing 1 |
| PODXL2 | -0.39590514 | podocalyxin like 2 |
| COL6A3 | -0.39545982 | collagen type VI alpha 3 chain |
| CD6 | -0.39526482 | CD6 molecule |
| TCHP | -0.39424769 | trichoplein keratin filament binding |
| MRPL11 | -0.39397101 | mitochondrial ribosomal protein L11 |
| PTGDS | -0.39320422 | prostaglandin D2 synthase |
| TBX1 | -0.39011594 | T-box 1 |
| BCL2L12 | -0.38954941 | BCL2 like 12 |
| SNRPB | -0.38913043 | small nuclear ribonucleoprotein polypeptides B and B1 |
| PFKFB4 | -0.38909354 | 6-phosphofructo-2-kinase/fructose-2,6-biphosphatase 4 |
| GPT2 | -0.3887668 | glutamic--pyruvic transaminase 2 |
| UHRF1 | -0.38826877 | ubiquitin like with PHD and ring finger domains 1 |
| SLC15A1 | -0.38778393 | solute carrier family 15 member 1 |
| MCM10 | -0.38742819 | minichromosome maintenance 10 replication initiation factor |
| KIF20A | -0.38621871 | kinesin family member 20A |
| PARM1 | -0.38498551 | prostate androgen-regulated mucin-like protein 1 |
| FOLR1 | -0.38371278 | folate receptor 1 |
| RPN2 | -0.38331752 | ribophorin II |
| RNF128 | -0.3832859 | ring finger protein 128, E3 ubiquitin protein ligase |
| FBLN1 | -0.38247431 | fibulin 1 |
| CGNL1 | -0.38240843 | cingulin like 1 |
| CEP55 | -0.38175758 | centrosomal protein 55 |
| FAM98C | -0.38143874 | family with sequence similarity 98 member C |
| NT5DC2 | -0.37951779 | 5'-nucleotidase domain containing 2 |
| SLC26A6 | -0.37812648 | solute carrier family 26 member 6 |
| CLEC11A | -0.37796574 | C-type lectin domain family 11 member A |
| MKI67 | -0.3775942 | marker of proliferation Ki-67 |
| CENPW | -0.37418709 | centromere protein W |
| E2F1 | -0.37286693 | E2F transcription factor 1 |
| IMPA2 | -0.37162055 | inositol monophosphatase 2 |
| HOXC9 | -0.37053491 | homeobox C9 |
| DTL | -0.36976548 | denticleless E3 ubiquitin protein ligase homolog |
| NUP210 | -0.36968379 | nucleoporin 210 |
| FANCD2 | -0.36887484 | Fanconi anemia complementation group D2 |
| ALDH1A2 | -0.36663768 | aldehyde dehydrogenase 1 family member A2 |
| HN1 | -0.36606324 | hematological and neurological expressed 1 |
| CDCA8 | -0.36564163 | cell division cycle associated 8 |
| C16orf59 | -0.36419236 | chromosome 16 open reading frame 59 |
| HSD3B1 | -0.36362055 | hydroxy-delta-5-steroid dehydrogenase, 3 beta- and steroid delta-isomerase 1 |
| CDK4 | -0.36236891 | cyclin dependent kinase 4 |
| GCN1 | -0.36186561 | GCN1, eIF2 alpha kinase activator homolog |
| TRIB3 | -0.36161001 | tribbles pseudokinase 3 |
| NES | -0.3602029 | nestin |
| SAMD4B | -0.36005797 | sterile alpha motif domain containing 4B |
| ETV4 | -0.35894598 | ETS variant 4 |
| SLC2A1 | -0.35861133 | solute carrier family 2 member 1 |
| LGALSL | -0.35854809 | galectin like |
| C19orf48 | -0.35844796 | chromosome 19 open reading frame 48 |
| TRIP13 | -0.35806324 | thyroid hormone receptor interactor 13 |
| DHCR7 | -0.35737549 | 7-dehydrocholesterol reductase |
| RNF144A | -0.35696443 | ring finger protein 144A |
| CENPL | -0.35516733 | centromere protein L |
| RASL12 | -0.35424506 | RAS like family 12 |
| HIST1H2BH | -0.35370487 | histone cluster 1, H2bh |
| RAD54L | -0.35328327 | RAD54-like (S. cerevisiae) |
| SCD | -0.35219499 | stearoyl-CoA desaturase |
| HOXA13 | -0.35136759 | homeobox A13 |
| LAMC3 | -0.35019499 | laminin subunit gamma 3 |
| KIF20B | -0.34998946 | kinesin family member 20B |
| CHURC1 | -0.34959947 | churchill domain containing 1 |
| POLD1 | -0.34844005 | DAN polymerase delta 1, catalytic subunit |
| KIF15 | -0.34787879 | kinesin family member 15 |
| OLFML2A | -0.34745455 | olfactomedin like 2A |
| ZNF585A | -0.34714888 | zinc finger protein 585A |
| C14orf2 | -0.34677997 | chromosome 14 open reading frame 2 |
| SNORD4A | -0.34655863 | small nucleolar RNA, C/D box 4A |
| DYNLL2 | -0.34652701 | dynein light chain LC8-type 2 |
| C8orf4 | -0.34639526 | chromosome 8 open reading frame 4 |
| FAM72D | -0.34637154 | family with sequence similarity 72 member D |
| MRPS26 | -0.34594993 | mitochondrial ribosomal protein S26 |
| ABHD17C | -0.34585771 | abhydrolase domain containing 17C |
| RNASEH2A | -0.34580237 | ribonuclease H2 subunit A |
| P3H4 | -0.34563373 | prolyl 3-hydroxylase family member 4 (non-enzymatic) |
| CIB2 | -0.34519104 | calcium and integrin binding family member 2 |
| HJURP | -0.34491963 | Holliday junction recognition protein |
| EDARADD | -0.3448195 | EDAR associated death domain |
| ASPM | -0.34481686 | abnormal spindle microtubule assembly |
| RPP25 | -0.34463505 | ribonuclease P/MRP subunit p25 |
| SYAP1 | -0.34405534 | synapse associated protein 1 |
| AURKB | -0.34394466 | aurora kinase B |
| PCK2 | -0.34393939 | phosphoenolpyruvate carboxykinase 2, mitochondrial |
| WISP3 | -0.34369697 | WNT1 inducible signaling pathway protein 3 |
| TMEM74B | -0.34339921 | transmembrane protein 74B |
| TGIF2 | -0.34276943 | TGFB induced factor homeobox 2 |
| CSK | -0.34250066 | c-src tyrosine kinase |
| PSORS1C2 | -0.34158366 | psoriasis susceptibility 1 candidate 2 |
| SLC38A1 | -0.3408274 | solute carrier family 38 member 1 |
| PPFIA1 | -0.3403083 | PTPRF interacting protein alpha 1 |
| FOXM1 | -0.33604743 | forkhead box M1 |
| PSMD8 | -0.33362055 | proteasome 26S subunit, non-ATPase 8 |
| WASH7P | -0.33355995 | WAS protein family homolog 7 pseudogene |
| WASH7P | -0.33355995 | WAS protein family homolog 7 pseudogene |
| WASH7P | -0.33355995 | WAS protein family homolog 7 pseudogene |
| WASH7P | -0.33355995 | WAS protein family homolog 7 pseudogene |
| NUDT1 | -0.3323083 | nudix hydrolase 1 |
| HIC2 | -0.33172596 | hypermethylated in cancer 2 |
| MORC4 | -0.33172596 | MORC family CW-type zinc finger 4 |
| SGO1 | -0.33126746 | shugoshin 1 |
| RAD51AP1 | -0.33115679 | RAD51 associated protein 1 |
| NDC80 | -0.33087484 | NDC80, kinetochore complex component |
| PIGU | -0.33009486 | phosphatidylinositol glycan anchor biosynthesis class U |
| PDGFRA | -0.32997365 | platelet derived growth factor receptor alpha |
| RNU6ATAC | -0.32864559 | RNA, U6atac small nuclear (U12-dependent splicing) |
| ASIC1 | -0.32845059 | acid sensing ion channel subunit 1 |
| COL1A2 | -0.32842161 | collagen type I alpha 2 chain |
| AXIN2 | -0.32790514 | axin 2 |
| DENND2A | -0.32721739 | DENN domain containing 2A |
| ELF4 | -0.32687484 | E74 like ETS transcription factor 4 |
| SLC9A3R1 | -0.3266087 | SLC9A3 regulator 1 |
| ATP6V0A4 | -0.32648221 | ATPase H+ transporting V0 subunit a4 |
| SPAG4 | -0.32498024 | sperm associated antigen 4 |
| IGFBP7 | -0.32386561 | insulin like growth factor binding protein 7 |
| DPP3 | -0.32368643 | dipeptidyl peptidase 3 |
| EOMES | -0.32332543 | eomesodermin |
| A2M | -0.32315679 | alpha-2-macroglobulin |
| NELL2 | -0.32274572 | neural EGFL like 2 |
| MCM5 | -0.32210277 | minichromosome maintenance complex component 5 |
| LPCAT3 | -0.32203426 | lysophosphatidylcholine acyltransferase 3 |
| ENO3 | -0.32137549 | enolase 3 |
| C2orf68 | -0.32099605 | chromosome 2 open reading frame 68 |
| CYP1B1 | -0.3200448 | cytochrome P450 family 1 subfamily B member 1 |
| PBK | -0.31991041 | PDZ binding kinase |
| DLGAP5 | -0.31980237 | DLG associated protein 5 |
| VCX | -0.31975231 | variable charge, X-linked |
| VCX | -0.31975231 | variable charge, X-linked |
| VCX | -0.31975231 | variable charge, X-linked |
| EFS | -0.31942292 | embryonal Fyn-associated substrate |
| HOXC6 | -0.31930171 | homeobox C6 |
| TCF3 | -0.31911726 | transcription factor 3 |
| PMEL | -0.31728063 | premelanosome protein |
| CCNA2 | -0.31642161 | cyclin A2 |
| RBM12 | -0.31571805 | RNA binding motif protein 12 |
| CHAF1B | -0.3148643 | chromatin assembly factor 1 subunit B |
| AEBP1 | -0.31446904 | AE binding protein 1 |
| NCKIPSD | -0.31385507 | NCK interacting protein with SH3 domain |
| P3H3 | -0.31339394 | prolyl 3-hydroxylase 3 |
| TYMSOS | -0.31257444 | TYMS opposite strand |
| TICRR | -0.31255072 | TOPBP1 interacting checkpoint and replication regulator |
| DSN1 | -0.31232938 | DSN1 homolog, MIS12 kinetochore complex component |
| THBS2 | -0.31226877 | thrombospondin 2 |
| PPP1R14A | -0.31017391 | protein phosphatase 1 regulatory inhibitor subunit 14A |
| DROSHA | -0.30938076 | drosha ribonuclease III |
| CCNG2 | -0.30719631 | cyclin G2 |
| CDH11 | -0.30718841 | cadherin 11 |
| GPI | -0.3071278 | glucose-6-phosphate isomerase |
| EXO5 | -0.30701713 | exonuclease 5 |
| PLPP1 | -0.30637418 | phospholipid phosphatase 1 |
| KNTC1 | -0.30625033 | kinetochore associated 1 |
| PRR11 | -0.30611594 | proline rich 11 |
| KIAA0101 | -0.30576812 | KIAA0101 |
| MGP | -0.30429513 | matrix Gla protein |
| MORF4L1 | -0.30411858 | mortality factor 4 like 1 |
| LIG1 | -0.30398419 | DNA ligase 1 |
| SPRR3 | -0.30320422 | small proline rich protein 3 |
| PRSS8 | -0.30296706 | protease, serine 8 |
| CD79A | -0.30283004 | CD79a molecule |
| KDM2B | -0.30050856 | lysine demethylase 2B |
| ANAPC15 | -0.30013966 | anaphase promoting complex subunit 15 |
| SVBP | -0.29967062 | small vasohibin binding protein |
| LOC101928126 | -0.29901713 | uncharacterized LOC101928126 |
| PRRT3 | -0.29816074 | proline rich transmembrane protein 3 |
| CENPN | -0.2979473 | centromere protein N |
| KIF11 | -0.29703821 | kinesin family member 11 |
| ADAD2 | -0.29642424 | adenosine deaminase domain containing 2 |
| PLAC1 | -0.29638999 | placenta specific 1 |
| MFSD5 | -0.29587879 | major facilitator superfamily domain containing 5 |
| SNX8 | -0.29552833 | sorting nexin 8 |
| SNX8 | -0.29552833 | sorting nexin 8 |
| CD27 | -0.29432411 | CD27 molecule |
| ANKRD35 | -0.29277997 | ankyrin repeat domain 35 |
| CELSR3 | -0.29083531 | cadherin EGF LAG seven-pass G-type receptor 3 |
| TARBP2 | -0.29051383 | TARBP2, RISC loading complex RNA binding subunit |
| KIF4A | -0.29018445 | kinesin family member 4A |
| TAGLN | -0.29012385 | transgelin |
| NME1 | -0.28906192 | NME/NM23 nucleoside diphosphate kinase 1 |
| SKA1 | -0.28880105 | spindle and kinetochore associated complex subunit 1 |
| DHRS13 | -0.28869829 | dehydrogenase/reductase 13 |
| PIM2 | -0.28857971 | Pim-2 proto-oncogene, serine/threonine kinase |
| ECE2 | -0.28794203 | endothelin converting enzyme 2 |
| POLR2J | -0.28788406 | RNA polymerase II subunit J |
| CALR | -0.28748353 | calreticulin |
| PPP1R16B | -0.28551252 | protein phosphatase 1 regulatory subunit 16B |
| EZH2 | -0.28518314 | enhancer of zeste 2 polycomb repressive complex 2 subunit |
| IRAK1 | -0.28516733 | interleukin 1 receptor associated kinase 1 |
| HIGD1A | -0.28506983 | HIG1 hypoxia inducible domain family member 1A |
| NLK | -0.28489592 | nemo like kinase |
| STOM | -0.28437154 | stomatin |
| RPS23 | -0.28421344 | ribosomal protein S23 |
| LRTM2 | -0.28419763 | leucine rich repeats and transmembrane domains 2 |
| UNC119 | -0.2837971 | unc-119 lipid binding chaperone |
| WDR92 | -0.28375494 | WD repeat domain 92 |
| CBLC | -0.28363373 | Cbl proto-oncogene C |
| SLC25A3 | -0.28330962 | solute carrier family 25 member 3 |
| HOXD9 | -0.28323057 | homeobox D9 |
| IQGAP3 | -0.28285903 | IQ motif containing GTPase activating protein 3 |
| ZNF35 | -0.28282213 | zinc finger protein 35 |
| KIF2C | -0.28276943 | kinesin family member 2C |
| SLC38A10 | -0.28229249 | solute carrier family 38 member 10 |
| ANLN | -0.28220553 | anillin actin binding protein |
| FAM110A | -0.28196574 | family with sequence similarity 110 member A |
| ORAOV1 | -0.28195257 | oral cancer overexpressed 1 |
| RECQL4 | -0.28182609 | RecQ like helicase 4 |
| TRIM28 | -0.28152042 | tripartite motif containing 28 |
| DMRTA2 | -0.2811805 | DMRT like family A2 |
| GAREM1 | -0.28108827 | GRB2 associated regulator of MAPK1 subtype 1 |
| IGSF9 | -0.28027931 | immunoglobulin superfamily member 9 |
| CST2 | -0.27989987 | cystatin SA |
| CORO1A | -0.27935441 | coronin 1A |
| POLR3F | -0.27910672 | RNA polymerase III subunit F |
| FURIN | -0.27817655 | furin, paired basic amino acid cleaving enzyme |
| CCNF | -0.27802635 | cyclin F |
| B4GALT3 | -0.27784717 | beta-1,4-galactosyltransferase 3 |
| ITGA8 | -0.2776469 | integrin subunit alpha 8 |
| RPL13AP6 | -0.27762582 | ribosomal protein L13a pseudogene 6 |
| C17orf53 | -0.27752569 | chromosome 17 open reading frame 53 |
| LOC390705 | -0.27739394 | protein phosphatase 2 regulatory subunit B'', beta pseudogene |
| PKP1 | -0.27737286 | plakophilin 1 |
| LRFN2 | -0.27719895 | leucine rich repeat and fibronectin type III domain containing 2 |
| DCN | -0.27690646 | decorin |
| SHMT2 | -0.27647431 | serine hydroxymethyltransferase 2 |
| PAG1 | -0.275639 | phosphoprotein membrane anchor with glycosphingolipid microdomains 1 |
| C19orf53 | -0.27550988 | chromosome 19 open reading frame 53 |
| CST3 | -0.27550725 | cystatin C |
| REL | -0.27544928 | REL proto-oncogene, NF-kB subunit |
| KPNA2 | -0.27512253 | karyopherin subunit alpha 2 |
| KPNA2 | -0.27512253 | karyopherin subunit alpha 2 |
| NUDT5 | -0.27503821 | nudix hydrolase 5 |
| FIRRE | -0.27485375 | firre intergenic repeating RNA element |
| P3H1 | -0.27378129 | prolyl 3-hydroxylase 1 |
| HSPA12B | -0.27367062 | heat shock protein family A (Hsp70) member 12B |
| CNOT3 | -0.27326219 | CCR4-NOT transcription complex subunit 3 |
| CSNK2A1 | -0.27153623 | casein kinase 2 alpha 1 |
| PYCR1 | -0.27063241 | pyrroline-5-carboxylate reductase 1 |
| CDC7 | -0.27040316 | cell division cycle 7 |
| LMNB2 | -0.27028195 | lamin B2 |
| SLC5A6 | -0.26993939 | solute carrier family 5 member 6 |
| WDR72 | -0.26982082 | WD repeat domain 72 |
| PAMR1 | -0.26908564 | peptidase domain containing associated with muscle regeneration 1 |
| BRI3BP | -0.26894598 | BRI3 binding protein |
| PDXDC2P | -0.26893017 | pyridoxal dependent decarboxylase domain containing 2, pseudogene |
| SPAG5 | -0.26798946 | sperm associated antigen 5 |
| CPSF3 | -0.26778129 | cleavage and polyadenylation specific factor 3 |
| SRRM1 | -0.26764427 | serine and arginine repetitive matrix 1 |
| ZDHHC24 | -0.26693544 | zinc finger DHHC-type containing 24 |
| BUB1 | -0.26686693 | BUB1 mitotic checkpoint serine/threonine kinase |
| LPCAT1 | -0.26637418 | lysophosphatidylcholine acyltransferase 1 |
| CYB5A | -0.26498287 | cytochrome b5 type A |
| RIMKLA | -0.26490382 | ribosomal modification protein rimK like family member A |
| TFPT | -0.26422134 | TCF3 fusion partner |
| ZNF551 | -0.26393676 | zinc finger protein 551 |
| DEDD | -0.26354414 | death effector domain containing |
| GUSB | -0.26344928 | glucuronidase beta |
| ISG15 | -0.26332279 | ISG15 ubiquitin-like modifier |
| OLFML1 | -0.26279578 | olfactomedin like 1 |
| HNRNPL | -0.26275099 | heterogeneous nuclear ribonucleoprotein L |
| SLC1A7 | -0.26252701 | solute carrier family 1 member 7 |
| SEMA3F | -0.26240316 | semaphorin 3F |
| ALG3 | -0.26203426 | ALG3, alpha-1,3- mannosyltransferase |
| NUF2 | -0.26076416 | NUF2, NDC80 kinetochore complex component |
| CSE1L | -0.26072464 | chromosome segregation 1 like |
| RFC5 | -0.2603531 | replication factor C subunit 5 |
| PPIF | -0.25980501 | peptidylprolyl isomerase F |
| STRA13 | -0.25941502 | stimulated by retinoic acid 13 |
| PKMYT1 | -0.25884058 | protein kinase, membrane associated tyrosine/threonine 1 |
| SALL4 | -0.25875889 | spalt like transcription factor 4 |
| ZNF570 | -0.25864559 | zinc finger protein 570 |
| RBPMS2 | -0.25861397 | RNA binding protein with multiple splicing 2 |
| CCDC142 | -0.2580975 | coiled-coil domain containing 142 |
| COL5A1 | -0.25786034 | collagen type V alpha 1 chain |
| KRTAP10-11 | -0.25779183 | keratin associated protein 10-11 |
| ALDH16A1 | -0.25772859 | aldehyde dehydrogenase 16 family member A1 |
| CIT | -0.25732016 | citron rho-interacting serine/threonine kinase |
| EXOSC5 | -0.25722266 | exosome component 5 |
| CALD1 | -0.25687747 | caldesmon 1 |
| TMC6 | -0.25674045 | transmembrane channel like 6 |
| CSN1S1 | -0.25662978 | casein alpha s1 |
| MRPL21 | -0.25618709 | mitochondrial ribosomal protein L21 |
| ATAD3A | -0.25579447 | ATPase family, AAA domain containing 3A |
| TYMS | -0.25524374 | thymidylate synthetase |
| ERGIC3 | -0.25512253 | ERGIC and golgi 3 |
| DCXR | -0.2549697 | dicarbonyl and L-xylulose reductase |
| PSMD6 | -0.2549249 | proteasome 26S subunit, non-ATPase 6 |
| RRP9 | -0.25439526 | ribosomal RNA processing 9, small subunit (SSU) processome component, homolog (yeast) |
| ACP7 | -0.25432938 | acid phosphatase 7, tartrate resistant (putative) |
| CD247 | -0.25419499 | CD247 molecule |
| POSTN | -0.25396311 | periostin |
| ETV6 | -0.25290382 | ETS variant 6 |
| CENPU | -0.25275626 | centromere protein U |
| DDX55 | -0.25176812 | DEAD-box helicase 55 |
| ADAMTSL2 | -0.25146245 | ADAMTS like 2 |
| ACY1 | -0.25043478 | aminoacylase 1 |
| FUT3 | -0.24983926 | fucosyltransferase 3 (Lewis blood group) |
| TBC1D10C | -0.24939921 | TBC1 domain family member 10C |
| NDUFS2 | -0.24937022 | NADH:ubiquinone oxidoreductase core subunit S2 |
| ANKRD16 | -0.24894071 | ankyrin repeat domain 16 |
| RASAL3 | -0.24868511 | RAS protein activator like 3 |
| ORC6 | -0.2486166 | origin recognition complex subunit 6 |
| GIMAP8 | -0.24860079 | GTPase, IMAP family member 8 |
| EFHD1 | -0.24827668 | EF-hand domain family member D1 |
| POLE | -0.24803953 | DNA polymerase epsilon, catalytic subunit |
| BSN | -0.24697497 | bassoon presynaptic cytomatrix protein |
| SEPN1 | -0.24581555 | selenoprotein N, 1 |
| ZNF556 | -0.24511726 | zinc finger protein 556 |
| TUBA1C | -0.24489855 | tubulin alpha 1c |
| CAMK2N1 | -0.24427668 | calcium/calmodulin dependent protein kinase II inhibitor 1 |
| EDNRA | -0.2440527 | endothelin receptor type A |
| IP6K1 | -0.24402635 | inositol hexakisphosphate kinase 1 |
| P4HA1 | -0.24388142 | prolyl 4-hydroxylase subunit alpha 1 |
| IQGAP1 | -0.24304875 | IQ motif containing GTPase activating protein 1 |
| CHPT1 | -0.2428643 | choline phosphotransferase 1 |
| DUOX2 | -0.24264295 | dual oxidase 2 |
| ACKR1 | -0.24249012 | atypical chemokine receptor 1 (Duffy blood group) |
| MS4A7 | -0.24235837 | membrane spanning 4-domains A7 |
| EBF1 | -0.24215547 | early B-cell factor 1 |
| EBF1 | -0.24215547 | early B-cell factor 1 |
| TOP3B | -0.24201054 | topoisomerase (DNA) III beta |
| FBL | -0.24198419 | fibrillarin |
| SMARCB1 | -0.24196311 | SWI/SNF related, matrix associated, actin dependent regulator of chromatin, subfamily b, member 1 |
| CNIH4 | -0.24168643 | cornichon family AMPA receptor auxiliary protein 4 |
| MED25 | -0.24156785 | mediator complex subunit 25 |
| MMP15 | -0.2412859 | matrix metallopeptidase 15 |
| CDCA4 | -0.24105665 | cell division cycle associated 4 |
| COX7A1 | -0.24101713 | cytochrome c oxidase subunit 7A1 |
| LPAR2 | -0.24075362 | lysophosphatidic acid receptor 2 |
| GPR89A | -0.24060606 | G protein-coupled receptor 89A |
| LRP8 | -0.24057971 | LDL receptor related protein 8 |
| GIMAP4 | -0.23930962 | GTPase, IMAP family member 4 |
| CDKN3 | -0.23925428 | cyclin dependent kinase inhibitor 3 |
| SERPINF1 | -0.2391726 | serpin family F member 1 |
| APEX2 | -0.238722 | apurinic/apyrimidinic endodeoxyribonuclease 2 |
| DARS2 | -0.23856917 | aspartyl-tRNA synthetase 2, mitochondrial |
| ITGAL | -0.23856653 | integrin subunit alpha L |
| DACT3 | -0.23854545 | dishevelled binding antagonist of beta catenin 3 |
| RPL13A | -0.23818972 | ribosomal protein L13a |
| COL8A1 | -0.23815547 | collagen type VIII alpha 1 chain |
| TPM3P9 | -0.23782345 | tropomyosin 3 pseudogene 9 |
| VIM | -0.2377444 | vimentin |
| BUB1B | -0.23761001 | BUB1 mitotic checkpoint serine/threonine kinase B |
| CHRNA5 | -0.23739921 | cholinergic receptor nicotinic alpha 5 subunit |
| SYDE1 | -0.23706192 | synapse defective Rho GTPase homolog 1 |
| LAD1 | -0.23701186 | ladinin 1 |
| CDCA2 | -0.23677734 | cell division cycle associated 2 |
| MIS18A | -0.23655072 | MIS18 kinetochore protein A |
| HES6 | -0.23632938 | hes family bHLH transcription factor 6 |
| RBM15B | -0.2358946 | RNA binding motif protein 15B |
| PREX1 | -0.23551779 | phosphatidylinositol-3,4,5-trisphosphate dependent Rac exchange factor 1 |
| CD4 | -0.23541502 | CD4 molecule |
| STK35 | -0.23525692 | serine/threonine kinase 35 |
| LINC01451 | -0.23507246 | long intergenic non-protein coding RNA 1451 |
| FCRL5 | -0.23491963 | Fc receptor like 5 |
| SDF2L1 | -0.23468775 | stromal cell derived factor 2 like 1 |
| SRC | -0.2344585 | SRC proto-oncogene, non-receptor tyrosine kinase |
| C12orf57 | -0.2344585 | chromosome 12 open reading frame 57 |
| PRNP | -0.23382082 | prion protein |
| PRKDC | -0.23349934 | protein kinase, DNA-activated, catalytic polypeptide |
| TTYH3 | -0.23317787 | tweety family member 3 |
| PLXNB1 | -0.23250066 | plexin B1 |
| FNDC5 | -0.23248748 | fibronectin type III domain containing 5 |
| SLC13A2 | -0.23238208 | solute carrier family 13 member 2 |
| WFDC1 | -0.23187615 | WAP four-disulfide core domain 1 |
| RAD54L2 | -0.23161792 | RAD54-like 2 (S. cerevisiae) |
| PTH1R | -0.23158893 | parathyroid hormone 1 receptor |
| RGS10 | -0.23142556 | regulator of G-protein signaling 10 |
| FAAP24 | -0.23114888 | Fanconi anemia core complex associated protein 24 |
| SFMBT1 | -0.2309697 | Scm-like with four mbt domains 1 |
| XRCC1 | -0.23074308 | X-ray repair cross complementing 1 |
| C3 | -0.23069302 | complement component 3 |
| C3 | -0.23069302 | complement component 3 |
| CEP152 | -0.23031094 | centrosomal protein 152 |
| C7 | -0.23001845 | complement component 7 |
| NIT1 | -0.22972069 | nitrilase 1 |
| LOC105376064 | -0.22962319 | uncharacterized LOC105376064 |
| SRM | -0.22852437 | spermidine synthase |
| HIST2H3D | -0.22779974 | histone cluster 2, H3d |
| HIST2H3D | -0.22779974 | histone cluster 2, H3d |
| UBE2Q1 | -0.22765481 | ubiquitin conjugating enzyme E2 Q1 |
| GET4 | -0.22743083 | golgi to ER traffic protein 4 |
| P2RY8 | -0.22735968 | purinergic receptor P2Y8 |
| ENC1 | -0.227278 | ectodermal-neural cortex 1 |
| FSTL1 | -0.22673254 | follistatin like 1 |
| TRAIP | -0.22650593 | TRAF interacting protein |
| RHBDD3 | -0.22637154 | rhomboid domain containing 3 |
| VASH2 | -0.22635573 | vasohibin 2 |
| NSMCE4A | -0.22632148 | NSE4 homolog A, SMC5-SMC6 complex component |
| FOXF2 | -0.22607642 | forkhead box F2 |
| TMEM114 | -0.22525692 | transmembrane protein 114 |
| ERO1A | -0.2248722 | endoplasmic reticulum oxidoreductase 1 alpha |
| RUNX3 | -0.22453755 | runt related transcription factor 3 |
| TEX264 | -0.22440843 | testis expressed 264 |
| CREG1 | -0.22353096 | cellular repressor of E1A stimulated genes 1 |
| MCRIP1 | -0.22339921 | MAPK regulated corepressor interacting protein 1 |
| HMGB3 | -0.22315942 | high mobility group box 3 |
| SP1 | -0.22294598 | Sp1 transcription factor |
| NOP2 | -0.22275362 | NOP2 nucleolar protein |
| GPC4 | -0.22262451 | glypican 4 |
| PLXNA1 | -0.22249275 | plexin A1 |
| MTG1 | -0.22219763 | mitochondrial ribosome associated GTPase 1 |
| MED29 | -0.2219473 | mediator complex subunit 29 |
| ACTN4 | -0.22183399 | actinin alpha 4 |
| SNRPA | -0.22138867 | small nuclear ribonucleoprotein polypeptide A |
| CTNNBL1 | -0.22105929 | catenin beta like 1 |
| MARVELD3 | -0.22082477 | MARVEL domain containing 3 |
| SCAND1 | -0.22067194 | SCAN domain containing 1 |
| ADGRA2 | -0.22044005 | adhesion G protein-coupled receptor A2 |
| RFWD3 | -0.22023715 | ring finger and WD repeat domain 3 |
| CARHSP1 | -0.21953096 | calcium regulated heat stable protein 1 |
| PTPRCAP | -0.21949407 | protein tyrosine phosphatase, receptor type C associated protein |
| FAM46C | -0.21935441 | family with sequence similarity 46 member C |
| PDIA4 | -0.2193386 | protein disulfide isomerase family A member 4 |
| TNFRSF13B | -0.21884321 | TNF receptor superfamily member 13B |
| HRC | -0.21880369 | histidine rich calcium binding protein |
| SMIM24 | -0.21834256 | small integral membrane protein 24 |
| NKRF | -0.21730435 | NFKB repressing factor |
| STMN1 | -0.21699868 | stathmin 1 |
| FANCE | -0.21691963 | Fanconi anemia complementation group E |
| HILPDA | -0.21689328 | hypoxia inducible lipid droplet associated |
| RFC4 | -0.21654018 | replication factor C subunit 4 |
| CEP250 | -0.21600264 | centrosomal protein 250 |
| TMEM214 | -0.2158498 | transmembrane protein 214 |
| TRAPPC6A | -0.21580501 | trafficking protein particle complex 6A |
| UBE2T | -0.21553887 | ubiquitin conjugating enzyme E2 T |
| HM13 | -0.21548353 | histocompatibility minor 13 |
| TSFM | -0.21506719 | Ts translation elongation factor, mitochondrial |
| VHL | -0.21456126 | von Hippel-Lindau tumor suppressor |
| SPRY1 | -0.21418182 | sprouty RTK signaling antagonist 1 |
| SLC19A1 | -0.21407905 | solute carrier family 19 member 1 |
| KRT13 | -0.21405534 | keratin 13 |
| CLDN11 | -0.21393939 | claudin 11 |
| CMPK1 | -0.21359157 | cytidine/uridine monophosphate kinase 1 |
| KMT2B | -0.21343083 | lysine methyltransferase 2B |
| PHKA2 | -0.21330435 | phosphorylase kinase regulatory subunit alpha 2 |
| P4HB | -0.2132253 | prolyl 4-hydroxylase subunit beta |
| RASGRP1 | -0.21310935 | RAS guanyl releasing protein 1 |
| RTN4R | -0.21299078 | reticulon 4 receptor |
| UQCRC2 | -0.21298814 | ubiquinol-cytochrome c reductase core protein II |
| WNT5A | -0.21199736 | Wnt family member 5A |
| CENPA | -0.21196838 | centromere protein A |
| LDB2 | -0.21189987 | LIM domain binding 2 |
| ERVW-1 | -0.21179183 | endogenous retrovirus group W member 1 |
| XRCC3 | -0.21176812 | X-ray repair cross complementing 3 |
| SS18L2 | -0.21132806 | SS18 like 2 |
| CNPY2 | -0.21085375 | canopy FGF signaling regulator 2 |
| KIAA1522 | -0.21059552 | KIAA1522 |
| USP21 | -0.21052964 | ubiquitin specific peptidase 21 |
| RPS26P11 | -0.21048748 | ribosomal protein S26 pseudogene 11 |
| SDK1 | -0.21022925 | sidekick cell adhesion molecule 1 |
| BID | -0.21014493 | BH3 interacting domain death agonist |
| ORC1 | -0.20987088 | origin recognition complex subunit 1 |
| ZNF526 | -0.20970224 | zinc finger protein 526 |
| CEMIP | -0.20908827 | cell migration inducing hyaluronan binding protein |
| MCMBP | -0.20848221 | minichromosome maintenance complex binding protein |
| TRIM65 | -0.20802372 | tripartite motif containing 65 |
| RNF186 | -0.20746772 | ring finger protein 186 |
| MIS18BP1 | -0.20739394 | MIS18 binding protein 1 |
| TLCD1 | -0.20737813 | TLC domain containing 1 |
| AIFM1 | -0.20683004 | apoptosis inducing factor, mitochondria associated 1 |
| NR2F1 | -0.20660343 | nuclear receptor subfamily 2 group F member 1 |
| LMNB1 | -0.20644269 | lamin B1 |
| VAX2 | -0.2062029 | ventral anterior homeobox 2 |
| TBC1D10B | -0.20611067 | TBC1 domain family member 10B |
| CHD7 | -0.20596311 | chromodomain helicase DNA binding protein 7 |
| C19orf54 | -0.20584453 | chromosome 19 open reading frame 54 |
| DSCC1 | -0.20574704 | DNA replication and sister chromatid cohesion 1 |
| EIF2S2 | -0.20573913 | eukaryotic translation initiation factor 2 subunit beta |
| HTRA1 | -0.20563636 | HtrA serine peptidase 1 |
| GDF11 | -0.20559157 | growth differentiation factor 11 |
| ISLR | -0.20545455 | immunoglobulin superfamily containing leucine rich repeat |
| THOP1 | -0.20532279 | thimet oligopeptidase 1 |
| KNOP1 | -0.20523847 | lysine rich nucleolar protein 1 |
| CCL21 | -0.20514625 | C-C motif chemokine ligand 21 |
| HSD11B2 | -0.20494862 | hydroxysteroid 11-beta dehydrogenase 2 |
| ENTPD1 | -0.2048274 | ectonucleoside triphosphate diphosphohydrolase 1 |
| CAMK1G | -0.20451647 | calcium/calmodulin dependent protein kinase IG |
| L2HGDH | -0.2044058 | L-2-hydroxyglutarate dehydrogenase |
| DLGAP1 | -0.20393939 | DLG associated protein 1 |
| OXTR | -0.20360474 | oxytocin receptor |
| XYLB | -0.20354677 | xylulokinase |
| NFKBIB | -0.20346509 | NFKB inhibitor beta |
| TRMT2B | -0.20330435 | tRNA methyltransferase 2 homolog B |
| ATRIP | -0.20293808 | ATR interacting protein |
| FOXN2 | -0.20291173 | forkhead box N2 |
| TNNT3 | -0.20288801 | troponin T3, fast skeletal type |
| EPB41L3 | -0.20287747 | erythrocyte membrane protein band 4.1 like 3 |
| FBXO17 | -0.20282477 | F-box protein 17 |
| CEP89 | -0.20274835 | centrosomal protein 89 |
| ETFA | -0.20265349 | electron transfer flavoprotein alpha subunit |
| ESM1 | -0.20228986 | endothelial cell specific molecule 1 |
| ZNF691 | -0.20217918 | zinc finger protein 691 |
| CSTF2 | -0.20197892 | cleavage stimulation factor subunit 2 |
| RPS5 | -0.20188669 | ribosomal protein S5 |
| PCIF1 | -0.20120685 | PDX1 C-terminal inhibiting factor 1 |
| PEX11B | -0.20118314 | peroxisomal biogenesis factor 11 beta |
| UQCRHL | -0.20114361 | ubiquinol-cytochrome c reductase hinge protein like |
| B4GALNT4 | -0.20097497 | beta-1,4-N-acetyl-galactosaminyltransferase 4 |
| C21orf59 | -0.20085375 | chromosome 21 open reading frame 59 |
| GRPEL2 | -0.20033729 | GrpE like 2, mitochondrial |
| PRSS27 | -0.20019499 | protease, serine 27 |
| STIL | -0.20012648 | SCL/TAL1 interrupting locus |
| B3GALT6 | -0.20004216 | beta-1,3-galactosyltransferase 6 |
| PYM1 | -0.20000527 | PYM homolog 1, exon junction complex associated factor |
| RPP14 | -0.19982872 | ribonuclease P/MRP subunit p14 |
| POP5 | -0.1996469 | POP5 homolog, ribonuclease P/MRP subunit |
| NPRL2 | -0.19957049 | NPR2-like, GATOR1 complex subunit |
| CNOT10 | -0.19949144 | CCR4-NOT transcription complex subunit 10 |
| PRICKLE3 | -0.19909618 | prickle planar cell polarity protein 3 |
| VCX2 | -0.19894862 | variable charge, X-linked 2 |
| TCF7L1 | -0.19852437 | transcription factor 7 like 1 |
| RHNO1 | -0.19844269 | RAD9-HUS1-RAD1 interacting nuclear orphan 1 |
| SCAP | -0.1983531 | SREBF chaperone |
| ST13P4 | -0.19801054 | suppression of tumorigenicity 13 (colon carcinoma) (Hsp70 interacting protein) pseudogene 4 |
| DDR2 | -0.19787088 | discoidin domain receptor tyrosine kinase 2 |
| TMEM94 | -0.19786825 | transmembrane protein 94 |
| PPDPF | -0.19771542 | pancreatic progenitor cell differentiation and proliferation factor |
| MANEAL | -0.19740711 | mannosidase endo-alpha like |
| FLNC | -0.19737022 | filamin C |
| CYGB | -0.1968195 | cytoglobin |
| MOGS | -0.19674572 | mannosyl-oligosaccharide glucosidase |
| YES1 | -0.19668511 | YES proto-oncogene 1, Src family tyrosine kinase |
| ARF4 | -0.19650329 | ADP ribosylation factor 4 |
| CSNK1D | -0.19599209 | casein kinase 1 delta |
| OTX1 | -0.19597892 | orthodenticle homeobox 1 |
| CC2D1A | -0.1957892 | coiled-coil and C2 domain containing 1A |
| DENR | -0.19506192 | density regulated re-initiation and release factor |
| BLK | -0.19452437 | BLK proto-oncogene, Src family tyrosine kinase |
| RALY | -0.19438208 | RALY heterogeneous nuclear ribonucleoprotein |
| U2AF2 | -0.19432148 | U2 small nuclear RNA auxiliary factor 2 |
| SNRPG | -0.19422925 | small nuclear ribonucleoprotein polypeptide G |
| AKTIP | -0.19402635 | AKT interacting protein |
| GIMAP5 | -0.19393412 | GTPase, IMAP family member 5 |
| ACOX2 | -0.19378129 | acyl-CoA oxidase 2 |
| DDIAS | -0.19355468 | DNA damage induced apoptosis suppressor |
| EMCN | -0.19340711 | endomucin |
| CENPK | -0.19335968 | centromere protein K |
| PSMD3 | -0.19334387 | proteasome 26S subunit, non-ATPase 3 |
| PRELID1 | -0.19332279 | PRELI domain containing 1 |
| RELL2 | -0.19311462 | RELT like 2 |
| CALM1 | -0.19302503 | calmodulin 1 |
| NUDT8 | -0.1925639 | nudix hydrolase 8 |
| TRIM24 | -0.19246113 | tripartite motif containing 24 |
| GTF2I | -0.19242161 | general transcription factor IIi |
| SEP6 | -0.19207905 | septin 6 |
| SNHG11 | -0.19158893 | small nucleolar RNA host gene 11 |
| CTSA | -0.19157839 | cathepsin A |
| ADRA2A | -0.19129117 | adrenoceptor alpha 2A |
| ANGPTL2 | -0.19121476 | angiopoietin like 2 |
| WDR62 | -0.19110408 | WD repeat domain 62 |
| PPIB | -0.19090382 | peptidylprolyl isomerase B |
| PLK4 | -0.19085112 | polo like kinase 4 |
| LETM1 | -0.19080896 | leucine zipper and EF-hand containing transmembrane protein 1 |
| HMMR | -0.19052437 | hyaluronan mediated motility receptor |
| PTTG1 | -0.19031357 | pituitary tumor-transforming 1 |
| DUXA | -0.1902556 | double homeobox A |
| LYL1 | -0.18992885 | LYL1, basic helix-loop-helix family member |
| COL15A1 | -0.18984717 | collagen type XV alpha 1 chain |
| TANGO2 | -0.18966535 | transport and golgi organization 2 homolog |
| GART | -0.18966271 | phosphoribosylglycinamide formyltransferase, phosphoribosylglycinamide synthetase, phosphoribosylaminoimidazole synthetase |
| C12orf10 | -0.18947563 | chromosome 12 open reading frame 10 |
| SUFU | -0.18922266 | SUFU negative regulator of hedgehog signaling |
| ZBTB45 | -0.18914097 | zinc finger and BTB domain containing 45 |
| LINC00563 | -0.18900395 | long intergenic non-protein coding RNA 563 |
| TMEM145 | -0.18896179 | transmembrane protein 145 |
| GJA1 | -0.18888801 | gap junction protein alpha 1 |
| CYP11A1 | -0.18867984 | cytochrome P450 family 11 subfamily A member 1 |
| RDH16 | -0.18865086 | retinol dehydrogenase 16 (all-trans) |
| RANGAP1 | -0.18860343 | Ran GTPase activating protein 1 |
| FZD10 | -0.18842161 | frizzled class receptor 10 |
| FAM189B | -0.18841107 | family with sequence similarity 189 member B |
| TEK | -0.18832938 | TEK receptor tyrosine kinase |
| CTAGE6 | -0.18808696 | CTAGE family member 6 |
| E2F6 | -0.18807642 | E2F transcription factor 6 |
| MTFP1 | -0.18794466 | mitochondrial fission process 1 |
| CCDC60 | -0.18788406 | coiled-coil domain containing 60 |
| ZIC2 | -0.18744664 | Zic family member 2 |
| POU2F1 | -0.18743083 | POU class 2 homeobox 1 |
| RHBG | -0.18674835 | Rh family B glycoprotein (gene/pseudogene) |
| ATP5J2 | -0.18661397 | ATP synthase, H+ transporting, mitochondrial Fo complex subunit F2 |
| GPR180 | -0.18657971 | G protein-coupled receptor 180 |
| CDK5RAP1 | -0.18627141 | CDK5 regulatory subunit associated protein 1 |
| DDX28 | -0.18619763 | DEAD-box helicase 28 |
| SYT11 | -0.18616337 | synaptotagmin 11 |
| VKORC1L1 | -0.18603426 | vitamin K epoxide reductase complex subunit 1 like 1 |
| TMEM119 | -0.18597101 | transmembrane protein 119 |
| PREB | -0.18554941 | prolactin regulatory element binding |
| SKP2 | -0.18516996 | S-phase kinase-associated protein 2, E3 ubiquitin protein ligase |
| MARS | -0.18484848 | methionyl-tRNA synthetase |
| C17orf62 | -0.18471146 | chromosome 17 open reading frame 62 |
| EIF2AK1 | -0.18442951 | eukaryotic translation initiation factor 2 alpha kinase 1 |
| VPS16 | -0.18428986 | VPS16, CORVET/HOPS core subunit |
| PRICKLE1 | -0.18411067 | prickle planar cell polarity protein 1 |
| ZNF28 | -0.18408432 | zinc finger protein 28 |
| EIF1B | -0.18403426 | eukaryotic translation initiation factor 1B |
| GZMH | -0.184 | granzyme H |
| TET1 | -0.1839473 | tet methylcytosine dioxygenase 1 |
| SWT1 | -0.18390777 | SWT1, RNA endoribonuclease homolog |
| PIGF | -0.18384453 | phosphatidylinositol glycan anchor biosynthesis class F |
| CERK | -0.18352306 | ceramide kinase |
| NAA10 | -0.18350988 | N(alpha)-acetyltransferase 10, NatA catalytic subunit |
| TAS2R10 | -0.18302767 | taste 2 receptor member 10 |
| KHSRP | -0.18301976 | KH-type splicing regulatory protein |
| RPIA | -0.18206588 | ribose 5-phosphate isomerase A |
| PDRG1 | -0.18187088 | p53 and DNA damage regulated 1 |
| TFAP2A | -0.18182345 | transcription factor AP-2 alpha |
| REXO1 | -0.18130962 | RNA exonuclease 1 homolog |
| PCSK5 | -0.18122793 | proprotein convertase subtilisin/kexin type 5 |
| ZNF280D | -0.18119104 | zinc finger protein 280D |
| SLC39A11 | -0.1811805 | solute carrier family 39 member 11 |
| ACAP1 | -0.18083267 | ArfGAP with coiled-coil, ankyrin repeat and PH domains 1 |
| RBP1 | -0.18067194 | retinol binding protein 1 |
| PSMA4 | -0.18030303 | proteasome subunit alpha 4 |
| DAZAP1 | -0.17971542 | DAZ associated protein 1 |
| SFT2D2 | -0.17962582 | SFT2 domain containing 2 |
| GMIP | -0.17936759 | GEM interacting protein |
| PHLDB3 | -0.17914097 | pleckstrin homology like domain family B member 3 |
| DUT | -0.17889855 | deoxyuridine triphosphatase |
| TYRO3 | -0.1786693 | TYRO3 protein tyrosine kinase |
| SMG7 | -0.17841634 | SMG7, nonsense mediated mRNA decay factor |
| TEX261 | -0.17825823 | testis expressed 261 |
| AUP1 | -0.17819236 | ancient ubiquitous protein 1 |
| LUM | -0.17812385 | lumican |
| SF3A2 | -0.17804216 | splicing factor 3a subunit 2 |
| CHTF18 | -0.17764163 | chromosome transmission fidelity factor 18 |
| TMEM115 | -0.17753623 | transmembrane protein 115 |
| PDCD2L | -0.17751515 | programmed cell death 2 like |
| PPOX | -0.17740711 | protoporphyrinogen oxidase |
| MICALL1 | -0.17727273 | MICAL like 1 |
| FCHO1 | -0.17720685 | FCH domain only 1 |
| DNASE1L2 | -0.17616337 | deoxyribonuclease 1 like 2 |
| HKR1 | -0.17613966 | HKR1, GLI-Kruppel zinc finger family member |
| ACVR2B | -0.17604743 | activin A receptor type 2B |
| RNF123 | -0.17593149 | ring finger protein 123 |
| FAM25A | -0.17561265 | family with sequence similarity 25 member A |
| FAM25A | -0.17561265 | family with sequence similarity 25 member A |
| USP4 | -0.17528327 | ubiquitin specific peptidase 4 |
| PMS2P2 | -0.1749249 | PMS1 homolog 2, mismatch repair system component pseudogene 2 |
| ROCK2 | -0.17474045 | Rho associated coiled-coil containing protein kinase 2 |
| ZNF366 | -0.17462187 | zinc finger protein 366 |
| KIF22 | -0.17461397 | kinesin family member 22 |
| GATC | -0.17428458 | glutamyl-tRNA amidotransferase subunit C |
| CNN3 | -0.17417391 | calponin 3 |
| CD1D | -0.17411067 | CD1d molecule |
| SLC39A3 | -0.17329908 | solute carrier family 39 member 3 |
| GLRX3 | -0.17321476 | glutaredoxin 3 |
| IL7R | -0.17306983 | interleukin 7 receptor |
| SRRD | -0.17245586 | SRR1 domain containing |
| CD37 | -0.17240316 | CD37 molecule |
| UHMK1 | -0.17227668 | U2AF homology motif (UHM) kinase 1 |
| CKAP2L | -0.17218182 | cytoskeleton associated protein 2 like |
| GARS | -0.17217655 | glycyl-tRNA synthetase |
| DNAJC30 | -0.17197892 | DnaJ heat shock protein family (Hsp40) member C30 |
| BCL7C | -0.1717444 | BCL tumor suppressor 7C |
| CTDSPL | -0.17160211 | CTD small phosphatase like |
| FDCSP | -0.17133597 | follicular dendritic cell secreted protein |
| ZFP64 | -0.17132543 | ZFP64 zinc finger protein |
| PCBP1 | -0.1712859 | poly(rC) binding protein 1 |
| SMARCC2 | -0.17112253 | SWI/SNF related, matrix associated, actin dependent regulator of chromatin subfamily c member 2 |
| KIF14 | -0.17102767 | kinesin family member 14 |
| ANAPC11 | -0.17094598 | anaphase promoting complex subunit 11 |
| COMTD1 | -0.17074835 | catechol-O-methyltransferase domain containing 1 |
| CNOT11 | -0.17055072 | CCR4-NOT transcription complex subunit 11 |
| TMED3 | -0.17052437 | transmembrane p24 trafficking protein 3 |
| SHISA5 | -0.17030567 | shisa family member 5 |
| MYL9 | -0.17028986 | myosin light chain 9 |
| CEBPG | -0.17028722 | CCAAT/enhancer binding protein gamma |
| HNRNPD | -0.17026877 | heterogeneous nuclear ribonucleoprotein D |
| C19orf24 | -0.17005007 | chromosome 19 open reading frame 24 |
| EXO1 | -0.17003426 | exonuclease 1 |
| CHMP4B | -0.16999473 | charged multivesicular body protein 4B |
| WNT4 | -0.16988406 | Wnt family member 4 |
| HNRNPA1 | -0.16980501 | heterogeneous nuclear ribonucleoprotein A1 |
| SSBP2 | -0.16966008 | single stranded DNA binding protein 2 |
| OSBPL10 | -0.16947826 | oxysterol binding protein like 10 |
| CLPTM1 | -0.16942292 | CLPTM1, transmembrane protein |
| KCNJ8 | -0.16924638 | potassium voltage-gated channel subfamily J member 8 |
| STIM2 | -0.16914625 | stromal interaction molecule 2 |
| UBL4A | -0.16901186 | ubiquitin like 4A |
| COIL | -0.16894862 | coilin |
| LEF1 | -0.16859289 | lymphoid enhancer binding factor 1 |
| CHAF1A | -0.16855863 | chromatin assembly factor 1 subunit A |
| RBM14 | -0.16854809 | RNA binding motif protein 14 |
| ZNF695 | -0.16846377 | zinc finger protein 695 |
| ANXA1 | -0.16797101 | annexin A1 |
| CPSF1 | -0.1678419 | cleavage and polyadenylation specific factor 1 |
| RPS27 | -0.16766798 | ribosomal protein S27 |
| DDOST | -0.16765481 | dolichyl-diphosphooligosaccharide--protein glycosyltransferase non-catalytic subunit |
| HAPLN1 | -0.16762582 | hyaluronan and proteoglycan link protein 1 |
| PRR12 | -0.16715152 | proline rich 12 |
| GRB7 | -0.16714097 | growth factor receptor bound protein 7 |
| CD79B | -0.16713834 | CD79b molecule |
| SCAMP5 | -0.16696443 | secretory carrier membrane protein 5 |
| DUSP16 | -0.16688274 | dual specificity phosphatase 16 |
| HCAR2 | -0.16687747 | hydroxycarboxylic acid receptor 2 |
| ZSCAN2 | -0.16656126 | zinc finger and SCAN domain containing 2 |
| CTSF | -0.16631884 | cathepsin F |
| GIMAP1 | -0.16629513 | GTPase, IMAP family member 1 |
| ITM2A | -0.16627931 | integral membrane protein 2A |
| FJX1 | -0.16606851 | four jointed box 1 |
| ANKRD13D | -0.1658498 | ankyrin repeat domain 13D |
| MFSD13A | -0.16578129 | major facilitator superfamily domain containing 13A |
| FRMD6 | -0.16510145 | FERM domain containing 6 |
| MND1 | -0.16489592 | meiotic nuclear divisions 1 |
| EMILIN1 | -0.16485112 | elastin microfibril interfacer 1 |
| KDELR1 | -0.16428986 | KDEL endoplasmic reticulum protein retention receptor 1 |
| CDC34 | -0.16427141 | cell division cycle 34 |
| RERGL | -0.16422661 | RERG like |
| UBALD2 | -0.16402108 | UBA like domain containing 2 |
| CPVL | -0.16394993 | carboxypeptidase, vitellogenic like |
| GATM | -0.16390777 | glycine amidinotransferase |
| GPKOW | -0.16343347 | G-patch domain and KOW motifs |
| SCUBE2 | -0.16332279 | signal peptide, CUB domain and EGF like domain containing 2 |
| NOTCH3 | -0.16328327 | notch 3 |
| PRICKLE2 | -0.16317787 | prickle planar cell polarity protein 2 |
| HTR2C | -0.16316206 | 5-hydroxytryptamine receptor 2C |
| CLDN5 | -0.16291436 | claudin 5 |
| MOXD1 | -0.16291173 | monooxygenase DBH like 1 |
| LAMA4 | -0.16278261 | laminin subunit alpha 4 |
| CCDC174 | -0.16264295 | coiled-coil domain containing 174 |
| CEP85L | -0.16262451 | centrosomal protein 85 like |
| CEP85L | -0.16262451 | centrosomal protein 85 like |
| NIPAL1 | -0.16241897 | NIPA like domain containing 1 |
| NAA20 | -0.16227404 | N(alpha)-acetyltransferase 20, NatB catalytic subunit |
| CLN6 | -0.16210013 | ceroid-lipofuscinosis, neuronal 6, late infantile, variant |
| SUZ12 | -0.16202899 | SUZ12 polycomb repressive complex 2 subunit |
| AMER1 | -0.16161792 | APC membrane recruitment protein 1 |
| SUV39H1 | -0.16125955 | suppressor of variegation 3-9 homolog 1 |
| SPNS1 | -0.16124901 | sphingolipid transporter 1 (putative) |
| ZNF829 | -0.16093808 | zinc finger protein 829 |
| SDHAF4 | -0.16087747 | succinate dehydrogenase complex assembly factor 4 |
| ZNF581 | -0.16065349 | zinc finger protein 581 |
| ERAL1 | -0.16042951 | Era like 12S mitochondrial rRNA chaperone 1 |
| ADRM1 | -0.16027404 | adhesion regulating molecule 1 |
| IPO9 | -0.15962846 | importin 9 |
| JAM2 | -0.15944137 | junctional adhesion molecule 2 |
| MFGE8 | -0.15940448 | milk fat globule-EGF factor 8 protein |
| LHFP | -0.15920949 | lipoma HMGIC fusion partner |
| KMT5A | -0.15889328 | lysine methyltransferase 5A |
| CCNT1 | -0.15875362 | cyclin T1 |
| MCM7 | -0.15874045 | minichromosome maintenance complex component 7 |
| ARL2BP | -0.1586614 | ADP ribosylation factor like GTPase 2 binding protein |
| HSPB6 | -0.15849539 | heat shock protein family B (small) member 6 |
| UNG | -0.1584664 | uracil DNA glycosylase |
| EDC3 | -0.15828458 | enhancer of mRNA decapping 3 |
| BSG | -0.15745718 | basigin (Ok blood group) |
| MRPL12 | -0.15723847 | mitochondrial ribosomal protein L12 |
| GMCL1 | -0.15722266 | germ cell-less, spermatogenesis associated 1 |
| NR2C2AP | -0.15719631 | nuclear receptor 2C2 associated protein |
| CBX2 | -0.15694335 | chromobox 2 |
| TATDN2 | -0.15683794 | TatD DNase domain containing 2 |
| WDCP | -0.15680105 | WD repeat and coiled coil containing |
| STRN4 | -0.15662187 | striatin 4 |
| AMMECR1 | -0.15659289 | Alport syndrome, mental retardation, midface hypoplasia and elliptocytosis chromosomal region gene 1 |
| ARMC6 | -0.15616864 | armadillo repeat containing 6 |
| SWSAP1 | -0.15591831 | SWIM-type zinc finger 7 associated protein 1 |
| GFRA2 | -0.15589196 | GDNF family receptor alpha 2 |
| UBE2J1 | -0.15577866 | ubiquitin conjugating enzyme E2 J1 |
| PLK1 | -0.15570751 | polo like kinase 1 |
| NR2F6 | -0.15532543 | nuclear receptor subfamily 2 group F member 6 |
| PLD3 | -0.15530698 | phospholipase D family member 3 |
| DDX49 | -0.1551357 | DEAD-box helicase 49 |
| URB2 | -0.15495389 | URB2 ribosome biogenesis 2 homolog (S. cerevisiae) |
| VAMP1 | -0.15471937 | vesicle associated membrane protein 1 |
| COL6A2 | -0.15462451 | collagen type VI alpha 2 chain |
| SLC25A39 | -0.15452437 | solute carrier family 25 member 39 |
| SLC39A6 | -0.15450856 | solute carrier family 39 member 6 |
| FAM127B | -0.15426877 | family with sequence similarity 127 member B |
| ZNF766 | -0.15420553 | zinc finger protein 766 |
| MRPL37 | -0.15400527 | mitochondrial ribosomal protein L37 |
| NAPSA | -0.15377075 | napsin A aspartic peptidase |
| DPT | -0.15338076 | dermatopontin |
| PUS7 | -0.15326219 | pseudouridylate synthase 7 (putative) |
| NARF | -0.15313043 | nuclear prelamin A recognition factor |
| CSPG4 | -0.15308037 | chondroitin sulfate proteoglycan 4 |
| FAM136A | -0.15300132 | family with sequence similarity 136 member A |
| TRIL | -0.15291436 | TLR4 interactor with leucine rich repeats |
| NDUFA4 | -0.15280632 | NDUFA4, mitochondrial complex associated |
| CDS1 | -0.15277734 | CDP-diacylglycerol synthase 1 |
| SLC6A6 | -0.15263241 | solute carrier family 6 member 6 |
| KRT8 | -0.15260079 | keratin 8 |
| TMEM106C | -0.15259289 | transmembrane protein 106C |
| MTF2 | -0.15258235 | metal response element binding transcription factor 2 |
| RBMX2 | -0.1525639 | RNA binding motif protein, X-linked 2 |
| TRIAP1 | -0.15244532 | TP53 regulated inhibitor of apoptosis 1 |
| KCNMB1 | -0.15244005 | potassium calcium-activated channel subfamily M regulatory beta subunit 1 |
| H2AFY2 | -0.1524058 | H2A histone family member Y2 |
| RAB3IP | -0.15225033 | RAB3A interacting protein |
| SNRK | -0.1521054 | SNF related kinase |
| OCIAD2 | -0.15184717 | OCIA domain containing 2 |
| TSEN54 | -0.15184453 | tRNA splicing endonuclease subunit 54 |
| PALD1 | -0.15178656 | phosphatase domain containing, paladin 1 |
| RNF34 | -0.15162319 | ring finger protein 34 |
| PFDN2 | -0.15134914 | prefoldin subunit 2 |
| GYPC | -0.15104875 | glycophorin C (Gerbich blood group) |
| POC1A | -0.15095389 | POC1 centriolar protein A |
| PPP4C | -0.15091436 | protein phosphatase 4 catalytic subunit |
| NCSTN | -0.15088274 | nicastrin |
| PDGFD | -0.15085375 | platelet derived growth factor D |
| JCHAIN | -0.15080105 | joining chain of multimeric IgA and IgM |
| LPIN1 | -0.15075099 | lipin 1 |
| CD2 | -0.15066403 | CD2 molecule |
| LSG1 | -0.15018709 | large 60S subunit nuclear export GTPase 1 |
| EBP | -0.15012912 | emopamil binding protein (sterol isomerase) |
| SHKBP1 | -0.14986561 | SH3KBP1 binding protein 1 |
| PLA2G2F | -0.14966535 | phospholipase A2 group IIF |
| SLC25A10 | -0.1495336 | solute carrier family 25 member 10 |
| G6PC3 | -0.1492332 | glucose-6-phosphatase catalytic subunit 3 |
| LRFN3 | -0.14922793 | leucine rich repeat and fibronectin type III domain containing 3 |
| B9D2 | -0.14893017 | B9 protein domain 2 |
| ATP2A2 | -0.14878524 | ATPase sarcoplasmic/endoplasmic reticulum Ca2+ transporting 2 |
| CCDC3 | -0.14842161 | coiled-coil domain containing 3 |
| KDM1A | -0.14833729 | lysine demethylase 1A |
| TMEM160 | -0.14833465 | transmembrane protein 160 |
| SSPN | -0.1482029 | sarcospan |
| WWC1 | -0.14810804 | WW and C2 domain containing 1 |
| DLGAP4 | -0.14795257 | DLG associated protein 4 |
| ZDHHC23 | -0.14773386 | zinc finger DHHC-type containing 23 |
| DKFZP586I1420 | -0.14770224 | uncharacterized protein DKFZp586I1420 |
| CCDC69 | -0.14749407 | coiled-coil domain containing 69 |
| DGUOK-AS1 | -0.14738603 | DGUOK antisense RNA 1 |
| ANKDD1A | -0.14737549 | ankyrin repeat and death domain containing 1A |
| TPSB2 | -0.14721212 | tryptase beta 2 (gene/pseudogene) |
| PTRF | -0.14689855 | polymerase I and transcript release factor |
| SLC25A13 | -0.14683531 | solute carrier family 25 member 13 |
| PUSL1 | -0.14679315 | pseudouridylate synthase-like 1 |
| SPIB | -0.14668775 | Spi-B transcription factor |
| UFD1L | -0.14665086 | ubiquitin fusion degradation 1 like (yeast) |
| SCML1 | -0.14662451 | sex comb on midleg-like 1 (Drosophila) |
| KAZN | -0.14657971 | kazrin, periplakin interacting protein |
| KIAA0100 | -0.14642424 | KIAA0100 |
| TAOK1 | -0.14635046 | TAO kinase 1 |
| GTSE1 | -0.14633202 | G2 and S-phase expressed 1 |
| PM20D2 | -0.14586561 | peptidase M20 domain containing 2 |
| ANO6 | -0.14547826 | anoctamin 6 |
| SPTBN2 | -0.14529117 | spectrin beta, non-erythrocytic 2 |
| PA2G4 | -0.14506192 | proliferation-associated 2G4 |
| ZFP42 | -0.14501186 | ZFP42 zinc finger protein |
| GIT2 | -0.14501186 | GIT ArfGAP 2 |
| GIT2 | -0.14501186 | GIT ArfGAP 2 |
| RBBP8NL | -0.14499341 | RBBP8 N-terminal like |
| FKBP4 | -0.14487484 | FK506 binding protein 4 |
| ZNF785 | -0.14484058 | zinc finger protein 785 |
| SNORD34 | -0.14459552 | small nucleolar RNA, C/D box 34 |
| NDUFA4L2 | -0.14452701 | NDUFA4, mitochondrial complex associated like 2 |
| OXER1 | -0.14435046 | oxoeicosanoid receptor 1 |
| SGTA | -0.14417128 | small glutamine rich tetratricopeptide repeat containing alpha |
| THOC5 | -0.14403953 | THO complex 5 |
| TRIM46 | -0.14403162 | tripartite motif containing 46 |
| BTG1 | -0.14390777 | BTG anti-proliferation factor 1 |
| MSI1 | -0.14388142 | musashi RNA binding protein 1 |
| NPTN | -0.14372332 | neuroplastin |
| PCGF1 | -0.14358366 | polycomb group ring finger 1 |
| DDX27 | -0.14330698 | DEAD-box helicase 27 |
| PMP22 | -0.1432859 | peripheral myelin protein 22 |
| ZNF74 | -0.14307246 | zinc finger protein 74 |
| SEC13 | -0.1428643 | SEC13 homolog, nuclear pore and COPII coat complex component |
| RBP5 | -0.14275362 | retinol binding protein 5 |
| MTA2 | -0.14255072 | metastasis associated 1 family member 2 |
| ZNF777 | -0.14249802 | zinc finger protein 777 |
| RUSC1 | -0.14233729 | RUN and SH3 domain containing 1 |
| HECW2 | -0.14222661 | HECT, C2 and WW domain containing E3 ubiquitin protein ligase 2 |
| EFTUD2 | -0.14220817 | elongation factor Tu GTP binding domain containing 2 |
| AQP1 | -0.14197892 | aquaporin 1 (Colton blood group) |
| DDX23 | -0.14187879 | DEAD-box helicase 23 |
| FAM43A | -0.14180237 | family with sequence similarity 43 member A |
| DDIT4L | -0.14171542 | DNA damage inducible transcript 4 like |
| MAPK13 | -0.14136759 | mitogen-activated protein kinase 13 |
| FLAD1 | -0.14095125 | flavin adenine dinucleotide synthetase 1 |
| RDH10 | -0.14071673 | retinol dehydrogenase 10 (all-trans) |
| COPG2 | -0.14067984 | coatomer protein complex subunit gamma 2 |
| ARID3B | -0.14048485 | AT-rich interaction domain 3B |
| ZNF593 | -0.14042424 | zinc finger protein 593 |
| ALPK2 | -0.14042424 | alpha kinase 2 |
| GZMK | -0.14022661 | granzyme K |
| FCER2 | -0.14016337 | Fc fragment of IgE receptor II |
| DNAJB11 | -0.14014229 | DnaJ heat shock protein family (Hsp40) member B11 |
| RITA1 | -0.14013439 | RBPJ interacting and tubulin associated 1 |
| VPS72 | -0.14003162 | vacuolar protein sorting 72 homolog |
| SLC30A3 | -0.14000527 | solute carrier family 30 member 3 |
| MBOAT7 | -0.13993412 | membrane bound O-acyltransferase domain containing 7 |
| POLR3B | -0.13990777 | RNA polymerase III subunit B |
| SEC61A2 | -0.13984453 | Sec61 translocon alpha 2 subunit |
| SEMA4F | -0.1396469 | ssemaphorin 4F |
| RUNDC3A | -0.13961265 | RUN domain containing 3A |
| JOSD2 | -0.13950725 | Josephin domain containing 2 |
| MRPS15 | -0.13937022 | mitochondrial ribosomal protein S15 |
| C3orf62 | -0.13925428 | chromosome 3 open reading frame 62 |
| EIF2AK3 | -0.13925428 | eukaryotic translation initiation factor 2 alpha kinase 3 |
| ZNF629 | -0.13908827 | zinc finger protein 629 |
| RAB11FIP4 | -0.13906719 | RAB11 family interacting protein 4 |
| ADH1B | -0.13898287 | alcohol dehydrogenase 1B (class I), beta polypeptide |
| UFSP1 | -0.13893017 | UFM1 specific peptidase 1 (inactive) |
| NBPF11 | -0.13890382 | neuroblastoma breakpoint family member 11 |
| CCDC33 | -0.13885639 | coiled-coil domain containing 33 |
| PIGR | -0.13835573 | polymeric immunoglobulin receptor |
| TMEM41A | -0.13832938 | transmembrane protein 41A |
| SLC39A7 | -0.13831094 | solute carrier family 39 member 7 |
| MAP3K9 | -0.13826877 | mitogen-activated protein kinase kinase kinase 9 |
| CXCR4 | -0.138 | C-X-C motif chemokine receptor 4 |
| WDR54 | -0.13775494 | WD repeat domain 54 |
| INTS7 | -0.13767852 | integrator complex subunit 7 |
| ANO10 | -0.13759157 | anoctamin 10 |
| SLC39A4 | -0.13738867 | solute carrier family 39 member 4 |
| SYPL1 | -0.13732543 | synaptophysin like 1 |
| GNB1L | -0.13731489 | G protein subunit beta 1 like |
| H3F3B | -0.13713834 | H3 histone, family 3B |
| ADAM15 | -0.13713043 | ADAM metallopeptidase domain 15 |
| RFXANK | -0.13699868 | regulatory factor X associated ankyrin containing protein |
| TOR3A | -0.13698024 | torsin family 3 member A |
| PELO | -0.13691436 | pelota homolog (Drosophila) |
| R3HDM2 | -0.13657708 | R3H domain containing 2 |
| ISLR2 | -0.13650329 | immunoglobulin superfamily containing leucine rich repeat 2 |
| C9orf3 | -0.1363531 | chromosome 9 open reading frame 3 |
| IER5L | -0.13601054 | immediate early response 5 like |
| PRADC1 | -0.13597892 | protease associated domain containing 1 |
| RAE1 | -0.13587352 | ribonucleic acid export 1 |
| TIMM17B | -0.13579183 | translocase of inner mitochondrial membrane 17 homolog B (yeast) |
| MRPL46 | -0.13577602 | mitochondrial ribosomal protein L46 |
| ZFP14 | -0.13567852 | ZFP14 zinc finger protein |
| PDK1 | -0.13562582 | pyruvate dehydrogenase kinase 1 |
| HGH1 | -0.13552306 | HGH1 homolog |
| MTMR14 | -0.13531489 | myotubularin related protein 14 |
| TLR10 | -0.13528327 | toll like receptor 10 |
| C7orf49 | -0.13518577 | chromosome 7 open reading frame 49 |
| GEMIN6 | -0.13514625 | gem nuclear organelle associated protein 6 |
| ROMO1 | -0.13503821 | reactive oxygen species modulator 1 |
| SEC22C | -0.13503294 | SEC22 homolog C, vesicle trafficking protein |
| B3GNT4 | -0.13490382 | UDP-GlcNAc:betaGal beta-1,3-N-acetylglucosaminyltransferase 4 |
| RNF113A | -0.13485639 | ring finger protein 113A |
| LSM3 | -0.13464822 | LSM3 homolog, U6 small nuclear RNA and mRNA degradation associated |
| CNRIP1 | -0.13450856 | cannabinoid receptor interacting protein 1 |
| EIF6 | -0.13432938 | eukaryotic translation initiation factor 6 |
| CXCR5 | -0.13429249 | C-X-C motif chemokine receptor 5 |
| TGFBR3 | -0.13409223 | transforming growth factor beta receptor 3 |
| ICA1L | -0.13405534 | islet cell autoantigen 1 like |
| ITGA10 | -0.13395784 | integrin subunit alpha 10 |
| NKG7 | -0.13378129 | natural killer cell granule protein 7 |
| ZNF264 | -0.13373123 | zinc finger protein 264 |
| ISCU | -0.13368116 | iron-sulfur cluster assembly enzyme |
| PRKCSH | -0.13311462 | protein kinase C substrate 80K-H |
| DIAPH3 | -0.13299868 | diaphanous related formin 3 |
| DIAPH3 | -0.13299868 | diaphanous related formin 3 |
| CYTH2 | -0.13287484 | cytohesin 2 |
| CENPE | -0.13265349 | centromere protein E |
| GAPDH | -0.13242688 | glyceraldehyde-3-phosphate dehydrogenase |
| NDUFA11 | -0.13241897 | NADH:ubiquinone oxidoreductase subunit A11 |
| TBC1D7 | -0.13238999 | TBC1 domain family member 7 |
| USF2 | -0.13237154 | upstream transcription factor 2, c-fos interacting |
| STXBP2 | -0.13228195 | syntaxin binding protein 2 |
| CD164L2 | -0.13220817 | CD164 molecule like 2 |
| BOP1 | -0.13208432 | block of proliferation 1 |
| S100A11 | -0.13208432 | S100 calcium binding protein A11 |
| BOP1 | -0.13208432 | block of proliferation 1 |
| ADAM17 | -0.132 | ADAM metallopeptidase domain 17 |
| CDK5R1 | -0.13199736 | cyclin dependent kinase 5 regulatory subunit 1 |
| NGDN | -0.1317971 | neuroguidin |
| PSRC1 | -0.13159684 | proline and serine rich coiled-coil 1 |
| ITGB2-AS1 | -0.13150988 | ITGB2 antisense RNA 1 |
| NUFIP2 | -0.13149144 | NUFIP2, FMR1 interacting protein 2 |
| TOMM40 | -0.13098287 | translocase of outer mitochondrial membrane 40 |
| ATAD5 | -0.13088011 | ATPase family, AAA domain containing 5 |
| ZDHHC16 | -0.13083531 | zinc finger DHHC-type containing 16 |
| SPDL1 | -0.13059289 | spindle apparatus coiled-coil protein 1 |
| INMT | -0.13025033 | indolethylamine N-methyltransferase |
| NEK2 | -0.13022398 | NIMA related kinase 2 |
| NDE1 | -0.13019763 | nudE neurodevelopment protein 1 |
| RTKN | -0.13001318 | rhotekin |
| C19orf25 | -0.13000264 | chromosome 19 open reading frame 25 |
| NOX4 | -0.1297444 | NADPH oxidase 4 |
| FAM64A | -0.12974177 | family with sequence similarity 64 member A |
| SMG9 | -0.12940975 | SMG9, nonsense mediated mRNA decay factor |
| LONP2 | -0.12940448 | lon peptidase 2, peroxisomal |
| ZNF234 | -0.12925428 | zinc finger protein 234 |
| CBFB | -0.12924901 | core-binding factor beta subunit |
| DAD1 | -0.12918577 | defender against cell death 1 |
| HOXA9 | -0.1291726 | homeobox A9 |
| YARS | -0.12914625 | tyrosyl-tRNA synthetase |
| REM1 | -0.1291357 | RRAD and GEM like GTPase 1 |
| APBA3 | -0.12910935 | amyloid beta precursor protein binding family A member 3 |
| MRGPRF | -0.12904875 | MAS related GPR family member F |
| CCNE1 | -0.12901976 | cyclin E1 |
| PAIP2B | -0.12899605 | poly(A) binding protein interacting protein 2B |
| RHOJ | -0.12872464 | ras homolog family member J |
| ANKEF1 | -0.12867194 | ankyrin repeat and EF-hand domain containing 1 |
| VCY1B | -0.12864295 | variable charge, Y-linked 1B |
| VCY1B | -0.12864295 | variable charge, Y-linked 1B |
| OXSM | -0.1284664 | 3-oxoacyl-ACP synthase, mitochondrial |
| RIPPLY3 | -0.12821871 | ripply transcriptional repressor 3 |
| SASH3 | -0.12813702 | SAM and SH3 domain containing 3 |
| PSIP1 | -0.12812912 | PC4 and SFRS1 interacting protein 1 |
| BAG6 | -0.12811594 | BCL2 associated athanogene 6 |
| KLHDC4 | -0.12811331 | kelch domain containing 4 |
| DENND5B | -0.12805534 | DENN domain containing 5B |
| SOX4 | -0.1280448 | SRY-box 4 |
| PCMT1 | -0.12800527 | protein-L-isoaspartate (D-aspartate) O-methyltransferase |
| SPARCL1 | -0.12784717 | SPARC like 1 |
| ATG101 | -0.12780501 | autophagy related 101 |
| LSR | -0.12762846 | lipolysis stimulated lipoprotein receptor |
| SMAD5 | -0.12750461 | SMAD family member 5 |
| RP9 | -0.12719368 | retinitis pigmentosa 9 (autosomal dominant) |
| PDCD4 | -0.12698287 | programmed cell death 4 (neoplastic transformation inhibitor) |
| PUS1 | -0.12671673 | pseudouridylate synthase 1 |
| SNORD30 | -0.12664822 | small nucleolar RNA, C/D box 30 |
| LIN9 | -0.12664559 | lin-9 DREAM MuvB core complex component |
| ZBTB39 | -0.12663768 | zinc finger and BTB domain containing 39 |
| ST13 | -0.12652174 | suppression of tumorigenicity 13 (colon carcinoma) (Hsp70 interacting protein) |
| ST14 | -0.12650329 | suppression of tumorigenicity 14 |
| FAM129C | -0.12647694 | family with sequence similarity 129 member C |
| SCAMP4 | -0.12619499 | secretory carrier membrane protein 4 |
| RPS24 | -0.12618445 | ribosomal protein S24 |
| ZNF398 | -0.1261581 | zinc finger protein 398 |
| NOS1AP | -0.12603689 | nitric oxide synthase 1 adaptor protein |
| ARHGEF19 | -0.12597628 | Rho guanine nucleotide exchange factor 19 |
| ZNF525 | -0.12592095 | zinc finger protein 525 |
| ALKBH2 | -0.12591568 | alkB homolog 2, alpha-ketoglutarate dependent dioxygenase |
| PDGFB | -0.12564954 | platelet derived growth factor subunit B |
| COPG1 | -0.12524901 | coatomer protein complex subunit gamma 1 |
| PPM1G | -0.12513043 | protein phosphatase, Mg2+/Mn2+ dependent 1G |
| ZNF282 | -0.12511726 | zinc finger protein 282 |
| ATF5 | -0.12504611 | activating transcription factor 5 |
| NOP14 | -0.12495125 | NOP14 nucleolar protein |
| C12orf49 | -0.12482213 | chromosome 12 open reading frame 49 |
| NRXN2 | -0.12473781 | neurexin 2 |
| MRPL19 | -0.1246614 | mitochondrial ribosomal protein L19 |
| ARHGAP27 | -0.12450856 | Rho GTPase activating protein 27 |
| TCL1A | -0.12442161 | T-cell leukemia/lymphoma 1A |
| GNL3L | -0.12438735 | G protein nucleolar 3 like |
| MTF1 | -0.1243531 | metal regulatory transcription factor 1 |
| CHRM3 | -0.12431621 | cholinergic receptor muscarinic 3 |
| CHRM3 | -0.12431621 | cholinergic receptor muscarinic 3 |
| LTBR | -0.12428986 | lymphotoxin beta receptor |
| KRT3 | -0.12427668 | keratin 3 |
| EFEMP1 | -0.12404216 | EGF containing fibulin like extracellular matrix protein 1 |
| CLEC3B | -0.12360211 | C-type lectin domain family 3 member B |
| TXNL4A | -0.12347563 | thioredoxin like 4A |
| PHF14 | -0.12347299 | PHD finger protein 14 |
| CCNG1 | -0.12338867 | cyclin G1 |
| VCAM1 | -0.12336759 | vascular cell adhesion molecule 1 |
| SUGCT | -0.12336232 | succinyl-CoA:glutarate-CoA transferase |
| SIX4 | -0.12335968 | SIX homeobox 4 |
| ELMOD3 | -0.12335705 | ELMO domain containing 3 |
| SFXN1 | -0.1228722 | sideroflexin 1 |
| NEDD8 | -0.12286957 | neural precursor cell expressed, developmentally down-regulated 8 |
| KIF23 | -0.12265876 | kinesin family member 23 |
| GNG11 | -0.12222398 | G protein subunit gamma 11 |
| RPL22 | -0.12214756 | ribosomal protein L22 |
| SAC3D1 | -0.12196838 | SAC3 domain containing 1 |
| MRPL9 | -0.12186561 | mitochondrial ribosomal protein L9 |
| CR2 | -0.12157576 | complement component 3d receptor 2 |
| ZAP70 | -0.12128327 | zeta chain of T cell receptor associated protein kinase 70 |
| SHCBP1 | -0.12110935 | SHC binding and spindle associated 1 |
| FAM135A | -0.12107773 | family with sequence similarity 135 member A |
| NOL12 | -0.12089592 | nucleolar protein 12 |
| PCDH18 | -0.12074835 | protocadherin 18 |
| BCL9 | -0.12073254 | B-cell CLL/lymphoma 9 |
| PYROXD1 | -0.12060343 | pyridine nucleotide-disulphide oxidoreductase domain 1 |
| LONP1 | -0.12056653 | lon peptidase 1, mitochondrial |
| ACAA2 | -0.12046904 | acetyl-CoA acyltransferase 2 |
| ZBTB46 | -0.1204137 | zinc finger and BTB domain containing 46 |
| ISOC2 | -0.12031884 | isochorismatase domain containing 2 |
| TP53I3 | -0.12027931 | tumor protein p53 inducible protein 3 |
| MDK | -0.12024242 | midkine (neurite growth-promoting factor 2) |
| ROCK1 | -0.12023452 | Rho associated coiled-coil containing protein kinase 1 |
| MRPS17 | -0.12017391 | mitochondrial ribosomal protein S17 |
| RALGAPA2 | -0.12012385 | Ral GTPase activating protein catalytic alpha subunit 2 |
| EIF2AK2 | -0.12002372 | eukaryotic translation initiation factor 2 alpha kinase 2 |
| ATP13A2 | -0.11981818 | ATPase 13A2 |
| LYRM2 | -0.11906192 | LYR motif containing 2 |
| GRWD1 | -0.11899868 | glutamate rich WD repeat containing 1 |
| MRTO4 | -0.11878524 | MRT4 homolog, ribosome maturation factor |
| RRP7A | -0.11845586 | ribosomal RNA processing 7 homolog A |
| NUDT21 | -0.11833202 | nudix hydrolase 21 |
| ASPSCR1 | -0.11831357 | ASPSCR1, UBX domain containing tether for SLC2A4 |
| CFL1 | -0.11821344 | cofilin 1 |
| FITM1 | -0.11820026 | fat storage inducing transmembrane protein 1 |
| SMOC2 | -0.11817391 | SPARC related modular calcium binding 2 |
| SLC9B2 | -0.11816337 | solute carrier family 9 member B2 |
| SLC9B2 | -0.11816337 | solute carrier family 9 member B2 |
| ZMYND8 | -0.1178419 | zinc finger MYND-type containing 8 |
| SLC6A8 | -0.11781818 | solute carrier family 6 member 8 |
| OIP5 | -0.11774967 | Opa interacting protein 5 |
| TNFRSF1B | -0.11758893 | TNF receptor superfamily member 1B |
| METTL7B | -0.11754941 | methyltransferase like 7B |
| IL17RB | -0.11751252 | interleukin 17 receptor B |
| FOXRED2 | -0.11741502 | FAD dependent oxidoreductase domain containing 2 |
| NUP93 | -0.11741238 | nucleoporin 93 |
| UBE2O | -0.11713834 | ubiquitin conjugating enzyme E2 O |
| MTA3 | -0.11712253 | metastasis associated 1 family member 3 |
| ME2 | -0.11708564 | malic enzyme 2 |
| FYN | -0.11702767 | FYN proto-oncogene, Src family tyrosine kinase |
| SOX7 | -0.11682477 | SRY-box 7 |
| FTL | -0.11680369 | ferritin light chain |
| TMEM65 | -0.11675889 | transmembrane protein 65 |
| MCAM | -0.11653755 | melanoma cell adhesion molecule |
| TUBG1 | -0.11632148 | tubulin gamma 1 |
| MYO1G | -0.11631884 | myosin IG |
| DGCR14 | -0.11616601 | DiGeorge syndrome critical region gene 14 |
| PLPP3 | -0.11608169 | phospholipid phosphatase 3 |
| TMEM109 | -0.11603162 | transmembrane protein 109 |
| PAQR4 | -0.11597892 | progestin and adipoQ receptor family member 4 |
| VAMP5 | -0.1158498 | vesicle associated membrane protein 5 |
| MTMR4 | -0.11561265 | myotubularin related protein 4 |
| IL16 | -0.11548353 | interleukin 16 |
| ZNF181 | -0.11526746 | zinc finger protein 181 |
| TMEM200A | -0.11496179 | transmembrane protein 200A |
| MED26 | -0.11490909 | mediator complex subunit 26 |
| RUVBL2 | -0.11473781 | RuvB like AAA ATPase 2 |
| FIS1 | -0.11456653 | fission, mitochondrial 1 |
| ANKRD13B | -0.11434519 | ankyrin repeat domain 13B |
| TMEM168 | -0.11424242 | transmembrane protein 168 |
| SLC46A3 | -0.11417391 | solute carrier family 46 member 3 |
| ENO1 | -0.11391568 | enolase 1 |
| DGCR8 | -0.11383136 | DGCR8 microprocessor complex subunit |
| POLA2 | -0.11362319 | DNA polymerase alpha 2, accessory subunit |
| MRPL38 | -0.11351252 | mitochondrial ribosomal protein L38 |
| NABP2 | -0.11344928 | nucleic acid binding protein 2 |
| SRRT | -0.11330962 | serrate, RNA effector molecule |
| CPSF4 | -0.11330435 | cleavage and polyadenylation specific factor 4 |
| MCOLN2 | -0.11308827 | mucolipin 2 |
| MICA | -0.11293808 | MHC class I polypeptide-related sequence A |
| METTL2A | -0.11276416 | methyltransferase like 2A |
| PLS1 | -0.11228458 | plastin 1 |
| RSL1D1 | -0.11166798 | ribosomal L1 domain containing 1 |
| FCN1 | -0.11144664 | ficolin 1 |
| TOMM22 | -0.11115415 | translocase of outer mitochondrial membrane 22 |
| FAM58A | -0.11111462 | family with sequence similarity 58 member A |
| SH3GL2 | -0.11105665 | SH3 domain containing GRB2 like 2, endophilin A1 |
| PYGO2 | -0.11102503 | pygopus family PHD finger 2 |
| RBM42 | -0.11089328 | RNA binding motif protein 42 |
| RPS27L | -0.11086957 | ribosomal protein S27 like |
| TTI1 | -0.1107668 | TELO2 interacting protein 1 |
| TIMM8B | -0.11072991 | translocase of inner mitochondrial membrane 8 homolog B |
| SMUG1 | -0.11071673 | single-strand-selective monofunctional uracil-DNA glycosylase 1 |
| PKM | -0.11050329 | pyruvate kinase, muscle |
| CHN1 | -0.1102556 | chimerin 1 |
| SLC37A4 | -0.11022661 | solute carrier family 37 member 4 |
| LEMD2 | -0.11013439 | LEM domain containing 2 |
| PPFIA3 | -0.11008959 | PTPRF interacting protein alpha 3 |
| DPYSL3 | -0.11002108 | dihydropyrimidinase like 3 |
| USB1 | -0.11 | U6 snRNA biogenesis phosphodiesterase 1 |
| TONSL | -0.10998682 | tonsoku-like, DNA repair protein |
| PDCD11 | -0.10996311 | programmed cell death 11 |
| LAX1 | -0.10973913 | lymphocyte transmembrane adaptor 1 |
| GALNT14 | -0.10962582 | polypeptide N-acetylgalactosaminyltransferase 14 |
| SHANK2 | -0.10960211 | SH3 and multiple ankyrin repeat domains 2 |
| CADM4 | -0.10959157 | cell adhesion molecule 4 |
| MIAT | -0.10949144 | myocardial infarction associated transcript (non-protein coding) |
| CDC25C | -0.10948353 | cell division cycle 25C |
| CHMP3 | -0.10945982 | charged multivesicular body protein 3 |
| CLEC4F | -0.10939921 | C-type lectin domain family 4 member F |
| RPL15 | -0.10936759 | ribosomal protein L15 |
| RASL11A | -0.10899341 | RAS like family 11 member A |
| HDAC11 | -0.10893281 | histone deacetylase 11 |
| TMEM79 | -0.10887747 | transmembrane protein 79 |
| C10orf2 | -0.10885903 | chromosome 10 open reading frame 2 |
| ATN1 | -0.10883794 | atrophin 1 |
| SEP1 | -0.10875889 | septin 1 |
| DNMT3B | -0.10849539 | DNA methyltransferase 3 beta |
| NHSL1 | -0.10842424 | NHS like 1 |
| PPP4R3B | -0.10841897 | protein phosphatase 4 regulatory subunit 3B |
| RASGRP2 | -0.10835573 | RAS guanyl releasing protein 2 |
| GIMAP7 | -0.10824769 | GTPase, IMAP family member 7 |
| TSPAN12 | -0.10780237 | tetraspanin 12 |
| ITK | -0.1077971 | IL2 inducible T-cell kinase |
| CD22 | -0.10772069 | CD22 molecule |
| CLCN5 | -0.10770751 | chloride voltage-gated channel 5 |
| FAM101B | -0.10769697 | family with sequence similarity 101 member B |
| ARL1 | -0.10732279 | ADP ribosylation factor like GTPase 1 |
| LAS1L | -0.10701976 | LAS1 like, ribosome biogenesis factor |
| CD9 | -0.1069776 | CD9 molecule |
| APH1B | -0.10693544 | aph-1 homolog B, gamma-secretase subunit |
| ANO8 | -0.10654809 | anoctamin 8 |
| CLSTN3 | -0.10653755 | calsyntenin 3 |
| ARAF | -0.10644269 | A-Raf proto-oncogene, serine/threonine kinase |
| RPS19BP1 | -0.10639789 | ribosomal protein S19 binding protein 1 |
| FCMR | -0.10637681 | Fc fragment of IgM receptor |
| PTCD1 | -0.1062029 | pentatricopeptide repeat domain 1 |
| DDX21 | -0.10600527 | DEAD-box helicase 21 |
| ZNF419 | -0.10597892 | zinc finger protein 419 |
| TRIM11 | -0.10590514 | tripartite motif containing 11 |
| PSMD11 | -0.1057971 | proteasome 26S subunit, non-ATPase 11 |
| MNX1 | -0.10571278 | motor neuron and pancreas homeobox 1 |
| EMC9 | -0.10570487 | ER membrane protein complex subunit 9 |
| POFUT1 | -0.10568906 | protein O-fucosyltransferase 1 |
| PFN1 | -0.1055415 | profilin 1 |
| POLE2 | -0.10545718 | DNA polymerase epsilon 2, accessory subunit |
| RALA | -0.10541765 | RALA Ras like proto-oncogene A |
| PCNT | -0.10537549 | pericentrin |
| RPUSD1 | -0.10498814 | RNA pseudouridylate synthase domain containing 1 |
| VPS4A | -0.10496706 | vacuolar protein sorting 4 homolog A |
| RPUSD3 | -0.10492227 | RNA pseudouridylate synthase domain containing 3 |
| EIF2S3 | -0.10491963 | eukaryotic translation initiation factor 2 subunit gamma |
| MRPL27 | -0.10490382 | mitochondrial ribosomal protein L27 |
| PTGS1 | -0.10469565 | prostaglandin-endoperoxide synthase 1 |
| TTF2 | -0.1042556 | transcription termination factor 2 |
| FBXW12 | -0.10425033 | F-box and WD repeat domain containing 12 |
| SOX12 | -0.10410277 | SRY-box 12 |
| ATRAID | -0.10406851 | all-trans retinoic acid induced differentiation factor |
| TYSND1 | -0.10399473 | trypsin domain containing 1 |
| DPY19L3 | -0.10393149 | dpy-19 like 3 (C. elegans) |
| MTPN | -0.10367062 | myotrophin |
| DPM3 | -0.1035336 | dolichyl-phosphate mannosyltransferase subunit 3 |
| PELP1 | -0.10347826 | proline, glutamate and leucine rich protein 1 |
| FARSA | -0.10331752 | phenylalanyl-tRNA synthetase alpha subunit |
| ZNF335 | -0.10329117 | zinc finger protein 335 |
| LTBP3 | -0.10327536 | latent transforming growth factor beta binding protein 3 |
| INTS1 | -0.10325165 | integrator complex subunit 1 |
| RTF1 | -0.10296706 | RTF1 homolog, Paf1/RNA polymerase II complex component |
| CARM1 | -0.10285112 | coactivator associated arginine methyltransferase 1 |
| RPS15A | -0.10281159 | ribosomal protein S15a |
| COL6A1 | -0.1024664 | collagen type VI alpha 1 chain |
| TFAP4 | -0.10218182 | transcription factor AP-4 |
| VAMP8 | -0.10212912 | vesicle associated membrane protein 8 |
| PSENEN | -0.10211594 | presenilin enhancer gamma-secretase subunit |
| AAAS | -0.10168116 | aladin WD repeat nucleoporin |
| SOWAHA | -0.10154941 | sosondowah ankyrin repeat domain family member A |
| CERS2 | -0.10145191 | ceramide synthase 2 |
| S1PR1 | -0.10141238 | sphingosine-1-phosphate receptor 1 |
| FXYD6 | -0.10138603 | FXYD domain containing ion transport regulator 6 |
| PPAN | -0.1013386 | peter pan homolog (Drosophila) |
| EME1 | -0.10122266 | essential meiotic structure-specific endonuclease 1 |
| ATMIN | -0.10120158 | ATM interactor |
| LRP5 | -0.10116996 | LDL receptor related protein 5 |
| SAP30L | -0.10116206 | SAP30 like |
| SAP30L | -0.10116206 | SAP30 like |
| RAD51 | -0.10099605 | RAD51 recombinase |
| HINT1 | -0.10084321 | histidine triad nucleotide binding protein 1 |
| FDXR | -0.10076943 | ferredoxin reductase |
| RPS27A | -0.10075626 | ribosomal protein S27a |
| CUL7 | -0.10060606 | cullin 7 |
| CRYAB | -0.10045059 | crystallin alpha B |
| FAM71E1 | -0.10044269 | family with sequence similarity 71 member E1 |
| CHEK1 | -0.10035573 | checkpoint kinase 1 |
| SNORD35A | -0.10012385 | small nucleolar RNA, C/D box 35A |
| MEG3 | -0.10004216 | maternally expressed 3 (non-protein coding) |
| MAPKAPK3 | -0.10000527 | mitogen-activated protein kinase-activated protein kinase 3 |
| SMTN | -0.09991304 | smoothelin |
| NDUFS6 | -0.09972069 | NADH:ubiquinone oxidoreductase subunit S6 |
| SF3B4 | -0.0995336 | splicing factor 3b subunit 4 |
| GCDH | -0.0994809 | glutaryl-CoA dehydrogenase |
| CCNY | -0.09931752 | cyclin Y |
| CRTC2 | -0.09925165 | CREB regulated transcription coactivator 2 |
| LRRC1 | -0.09911989 | leucine rich repeat containing 1 |
| SZRD1 | -0.09905665 | SUZ RNA binding domain containing 1 |
| BRK1 | -0.09890909 | BRICK1, SCAR/WAVE actin nucleating complex subunit |
| UVSSA | -0.09867984 | UV stimulated scaffold protein A |
| NIPSNAP1 | -0.09863505 | nipsnap homolog 1 (C. elegans) |
| RGS11 | -0.09830303 | regulator of G-protein signaling 11 |
| MTIF2 | -0.09828722 | mitochondrial translational initiation factor 2 |
| HOXA10 | -0.09827668 | homeobox A10 |
| EIF5A | -0.09822925 | eukaryotic translation initiation factor 5A |
| RPP21 | -0.09818972 | ribonuclease P/MRP subunit p21 |
| NCAPH2 | -0.09791041 | non-SMC condensin II complex subunit H2 |
| AGO2 | -0.09785244 | argonaute 2, RISC catalytic component |
| MPST | -0.0977892 | mercaptopyruvate sulfurtransferase |
| C10orf142 | -0.09741765 | chromosome 10 open reading frame 142 |
| ZNF792 | -0.0973834 | zinc finger protein 792 |
| FGF12 | -0.09735705 | fibroblast growth factor 12 |
| H3F3C | -0.09731752 | H3 histone, family 3C |
| TMEM176A | -0.09684585 | transmembrane protein 176A |
| RAB11FIP5 | -0.09662978 | RAB11 family interacting protein 5 |
| PLCL2 | -0.09636627 | phospholipase C like 2 |
| CRB3 | -0.0963083 | crumbs 3, cell polarity complex component |
| SRPRB | -0.09610013 | SRP receptor beta subunit |
| CLIP3 | -0.09609486 | CAP-Gly domain containing linker protein 3 |
| MFAP4 | -0.09587352 | microfibrillar associated protein 4 |
| RTP5 | -0.09585507 | receptor transporter protein 5 (putative) |
| ACTN1 | -0.09571014 | actinin alpha 1 |
| RCC1 | -0.09561528 | regulator of chromosome condensation 1 |
| IL1B | -0.09561528 | interleukin 1 beta |
| PLA2G4C | -0.09555731 | phospholipase A2 group IVC |
| UQCR10 | -0.09525692 | ubiquinol-cytochrome c reductase, complex III subunit X |
| ABRACL | -0.09521476 | ABRA C-terminal like |
| SESN2 | -0.09515415 | sestrin 2 |
| NCF4 | -0.09498024 | neutrophil cytosolic factor 4 |
| C6orf120 | -0.09495916 | chromosome 6 open reading frame 120 |
| GIT1 | -0.09493017 | GIT ArfGAP 1 |
| WIPF1 | -0.09456653 | WAS/WASL interacting protein family member 1 |
| CCDC77 | -0.09438735 | coiled-coil domain containing 77 |
| SPC25 | -0.09436364 | SPC25, NDC80 kinetochore complex component |
| OTOF | -0.09432938 | otoferlin |
| CCDC18 | -0.09407642 | coiled-coil domain containing 18 |
| EEF1B2 | -0.09406061 | eukaryotic translation elongation factor 1 beta 2 |
| TNIP2 | -0.09401054 | TNFAIP3 interacting protein 2 |
| SNORD32A | -0.09398682 | small nucleolar RNA, C/D box 32A |
| CXCL12 | -0.09396574 | C-X-C motif chemokine ligand 12 |
| CCDC71 | -0.0937892 | coiled-coil domain containing 71 |
| IFT20 | -0.09367062 | intraflagellar transport 20 |
| SNF8 | -0.09363636 | SNF8, ESCRT-II complex subunit |
| CD40LG | -0.09361528 | CD40 ligand |
| EIF4EBP2 | -0.09339921 | eukaryotic translation initiation factor 4E binding protein 2 |
| TSSC1 | -0.09333333 | tumor suppressing subtransferable candidate 1 |
| STAMBPL1 | -0.09319368 | STAM binding protein like 1 |
| KRT31 | -0.09296179 | keratin 31 |
| ATP13A1 | -0.09277207 | ATPase 13A1 |
| WDR73 | -0.09276416 | WD repeat domain 73 |
| TAOK2 | -0.09276153 | TAO kinase 2 |
| DCANP1 | -0.09274572 | dendritic cell associated nuclear protein |
| AGMAT | -0.09271937 | agmatinase |
| PYCRL | -0.0927141 | pyrroline-5-carboxylate reductase-like |
| CHRD | -0.09264822 | chordin |
| F10 | -0.09262714 | coagulation factor X |
| PP7080 | -0.09246113 | uncharacterized LOC25845 |
| DUS4L | -0.09228722 | dihydrouridine synthase 4 like |
| LINC01109 | -0.0921502 | long intergenic non-protein coding RNA 1109 |
| SEMA4D | -0.09210804 | semaphorin 4D |
| ELK1 | -0.09205007 | ELK1, ETS transcription factor |
| LAMP2 | -0.09192358 | lysosomal associated membrane protein 2 |
| LAMP2 | -0.09192358 | lysosomal associated membrane protein 2 |
| SGSM1 | -0.09190777 | small G protein signaling modulator 1 |
| LOC100288893 | -0.09169433 | uncharacterized LOC100288893 |
| LINC00341 | -0.0915863 | long intergenic non-protein coding RNA 341 |
| CCR6 | -0.09147299 | C-C motif chemokine receptor 6 |
| MMP11 | -0.09141765 | matrix metallopeptidase 11 |
| SLC5A3 | -0.0913834 | solute carrier family 5 member 3 |
| LLPH | -0.09114361 | LLP homolog, long-term synaptic facilitation |
| RNF130 | -0.09099078 | ring finger protein 130 |
| TXNDC12 | -0.09096443 | thioredoxin domain containing 12 |
| AKR1E2 | -0.0908274 | aldo-keto reductase family 1 member E2 |
| LOC102724002 | -0.09081159 | uncharacterized LOC102724002 |
| SLC25A38 | -0.09080896 | solute carrier family 25 member 38 |
| DNASE1 | -0.09078524 | deoxyribonuclease 1 |
| BLMH | -0.09063768 | bleomycin hydrolase |
| BMP4 | -0.09057971 | bone morphogenetic protein 4 |
| FBXL20 | -0.09055072 | F-box and leucine rich repeat protein 20 |
| CELSR2 | -0.09042951 | cadherin EGF LAG seven-pass G-type receptor 2 |
| STAP2 | -0.09034783 | signal transducing adaptor family member 2 |
| RFNG | -0.09026087 | RFNG O-fucosylpeptide 3-beta-N-acetylglucosaminyltransferase |
| HIST1H2BC | -0.09018182 | histone cluster 1, H2bc |
| RRM1 | -0.08992885 | ribonucleotide reductase catalytic subunit M1 |
| DUS3L | -0.08991831 | dihydrouridine synthase 3 like |
| SYNM | -0.08983136 | synemin |
| CD3EAP | -0.08958366 | CD3e molecule associated protein |
| GFM2 | -0.08949934 | G elongation factor mitochondrial 2 |
| EIF3L | -0.08945718 | eukaryotic translation initiation factor 3 subunit L |
| RBM23 | -0.08924111 | RNA binding motif protein 23 |
| ZNF530 | -0.08885903 | zinc finger protein 530 |
| RPL7 | -0.08875626 | ribosomal protein L7 |
| DHX8 | -0.08869565 | DEAH-box helicase 8 |
| KISS1R | -0.08854018 | KISS1 receptor |
| ADGRG5 | -0.08848485 | adhesion G protein-coupled receptor G5 |
| TRMT2A | -0.08844269 | tRNA methyltransferase 2 homolog A |
| SIX1 | -0.08838735 | SIX homeobox 1 |
| TGFB3 | -0.08834256 | transforming growth factor beta 3 |
| ABI3 | -0.08822398 | ABI family member 3 |
| FAM65C | -0.08818445 | family with sequence similarity 65 member C |
| FOXA3 | -0.08817655 | forkhead box A3 |
| XIAP | -0.08797892 | X-linked inhibitor of apoptosis |
| HCN3 | -0.08781291 | hyperpolarization activated cyclic nucleotide gated potassium channel 3 |
| SNAI2 | -0.08749671 | snail family transcriptional repressor 2 |
| TGFB1I1 | -0.08740448 | transforming growth factor beta 1 induced transcript 1 |
| ROR1 | -0.08718841 | receptor tyrosine kinase like orphan receptor 1 |
| ROR1 | -0.08718841 | receptor tyrosine kinase like orphan receptor 1 |
| TMEM123 | -0.08707773 | transmembrane protein 123 |
| MBD2 | -0.08705665 | methyl-CpG binding domain protein 2 |
| CLEC1A | -0.08700395 | C-type lectin domain family 1 member A |
| CD3E | -0.08693281 | CD3e molecule |
| SH2B3 | -0.08693281 | SH2B adaptor protein 3 |
| NCOA5 | -0.08678788 | nuclear receptor coactivator 5 |
| ZNF524 | -0.08670619 | zinc finger protein 524 |
| DUS2 | -0.08662978 | dihydrouridine synthase 2 |
| TCF4 | -0.08655336 | transcription factor 4 |
| MAN2A1 | -0.08639262 | mannosidase alpha class 2A member 1 |
| TBRG4 | -0.08622925 | transforming growth factor beta regulator 4 |
| ACVRL1 | -0.08587879 | activin A receptor like type 1 |
| DYRK1A | -0.08582082 | dual specificity tyrosine phosphorylation regulated kinase 1A |
| SCAF1 | -0.08537549 | SR-related CTD associated factor 1 |
| GLA | -0.08528327 | galactosidase alpha |
| VSIG10L | -0.08514097 | V-set and immunoglobulin domain containing 10 like |
| ZNF485 | -0.08510672 | zinc finger protein 485 |
| B3GNT2 | -0.08486957 | UDP-GlcNAc:betaGal beta-1,3-N-acetylglucosaminyltransferase 2 |
| DHRS11 | -0.0847747 | dehydrogenase/reductase 11 |
| CHTOP | -0.08475099 | chromatin target of PRMT1 |
| TAF1L | -0.08470356 | TATA-box binding protein associated factor 1 like |
| FIZ1 | -0.08467984 | FLT3 interacting zinc finger 1 |
| KLHL25 | -0.08463241 | kelch like family member 25 |
| ZSCAN29 | -0.0846166 | zinc finger and SCAN domain containing 29 |
| MIEN1 | -0.0844664 | migration and invasion enhancer 1 |
| MAGEH1 | -0.08446377 | MAGE family member H1 |
| SLC12A7 | -0.08446113 | solute carrier family 12 member 7 |
| DEPDC1 | -0.08442951 | DEP domain containing 1 |
| TMEM106A | -0.08428986 | transmembrane protein 106A |
| CRISPLD2 | -0.08427931 | cysteine rich secretory protein LCCL domain containing 2 |
| SLIT2 | -0.08413702 | slit guidance ligand 2 |
| PRPSAP1 | -0.08412385 | phosphoribosyl pyrophosphate synthetase associated protein 1 |
| GIPC1 | -0.08405534 | GIPC PDZ domain containing family member 1 |
| TRAF3IP3 | -0.084 | TRAF3 interacting protein 3 |
| DDI2 | -0.08388142 | DNA damage inducible 1 homolog 2 |
| EHD2 | -0.0834361 | EH domain containing 2 |
| LCMT1 | -0.08331489 | leucine carboxyl methyltransferase 1 |
| RACGAP1 | -0.0832859 | Rac GTPase activating protein 1 |
| CLIC4 | -0.08324901 | chloride intracellular channel 4 |
| SOCS2 | -0.08314097 | suppressor of cytokine signaling 2 |
| PRRT2 | -0.08280369 | proline rich transmembrane protein 2 |
| CYTIP | -0.08274835 | cytohesin 1 interacting protein |
| TXN2 | -0.08250329 | thioredoxin 2 |
| AIFM3 | -0.08238999 | apoptosis inducing factor, mitochondria associated 3 |
| ACAT1 | -0.08237681 | acetyl-CoA acetyltransferase 1 |
| TMUB1 | -0.08222925 | transmembrane and ubiquitin like domain containing 1 |
| NAXE | -0.08212121 | NAD(P)HX epimerase |
| CSGALNACT2 | -0.08206851 | chondroitin sulfate N-acetylgalactosaminyltransferase 2 |
| PPHLN1 | -0.08198419 | periphilin 1 |
| ABHD2 | -0.08183926 | abhydrolase domain containing 2 |
| BORA | -0.08176285 | bora, aurora kinase A activator |
| SNRNP35 | -0.08172859 | small nuclear ribonucleoprotein U11/U12 subunit 35 |
| BRWD3 | -0.08171805 | bromodomain and WD repeat domain containing 3 |
| BTBD1 | -0.08170224 | BTB domain containing 1 |
| ZNF668 | -0.08164427 | zinc finger protein 668 |
| ANKMY2 | -0.08134387 | ankyrin repeat and MYND domain containing 2 |
| ZFAND2A | -0.08129644 | zinc finger AN1-type containing 2A |
| LOC374443 | -0.08122266 | C-type lectin domain family 2 member D pseudogene |
| MRPL33 | -0.08116996 | mitochondrial ribosomal protein L33 |
| PRPS1 | -0.08113043 | phosphoribosyl pyrophosphate synthetase 1 |
| KIF2A | -0.08110672 | kinesin family member 2A |
| AKAP12 | -0.08094598 | A-kinase anchoring protein 12 |
| BCORL1 | -0.08094071 | BCL6 corepressor-like 1 |
| ARPP19 | -0.08084321 | cAMP regulated phosphoprotein 19 |
| NUDT19 | -0.08073781 | nudix hydrolase 19 |
| SF3B3 | -0.08073254 | splicing factor 3b subunit 3 |
| TMEM208 | -0.08065086 | transmembrane protein 208 |
| MARK2 | -0.08054809 | microtubule affinity regulating kinase 2 |
| ARHGAP9 | -0.0804585 | Rho GTPase activating protein 9 |
| UCK2 | -0.08027404 | uridine-cytidine kinase 2 |
| FANCB | -0.08010804 | Fanconi anemia complementation group B |
| GPS1 | -0.0799473 | G protein pathway suppressor 1 |
| MS4A1 | -0.07986561 | membrane spanning 4-domains A1 |
| LGALS3 | -0.07985507 | lectin, galactoside binding soluble 3 |
| LGALS3 | -0.07985507 | lectin, galactoside binding soluble 3 |
| SNORA7B | -0.07958366 | small nucleolar RNA, H/ACA box 7B |
| MAP1A | -0.07955995 | microtubule associated protein 1A |
| ITFG1 | -0.07955731 | integrin alpha FG-GAP repeat containing 1 |
| FLVCR1 | -0.07926746 | feline leukemia virus subgroup C cellular receptor 1 |
| MAPK6 | -0.07916996 | mitogen-activated protein kinase 6 |
| LAMB2 | -0.07902767 | laminin subunit beta 2 |
| ASB18 | -0.07884321 | ankyrin repeat and SOCS box containing 18 |
| MAR5 | -0.07859816 | membrane associated ring-CH-type finger 5 |
| VPS26A | -0.07856653 | VPS26, retromer complex component A |
| C12orf66 | -0.07847167 | chromosome 12 open reading frame 66 |
| UQCC2 | -0.07845586 | ubiquinol-cytochrome c reductase complex assembly factor 2 |
| P2RY10 | -0.07842951 | purinergic receptor P2Y10 |
| P2RY10 | -0.07842951 | purinergic receptor P2Y10 |
| C6orf136 | -0.07824769 | chromosome 6 open reading frame 136 |
| HAAO | -0.07811594 | 3-hydroxyanthranilate 3,4-dioxygenase |
| ZNF793 | -0.07808169 | zinc finger protein 793 |
| TXNDC15 | -0.07802899 | thioredoxin domain containing 15 |
| BBC3 | -0.07787352 | BCL2 binding component 3 |
| ATP5J | -0.07783399 | ATP synthase, H+ transporting, mitochondrial Fo complex subunit F6 |
| SUGP1 | -0.07777866 | SURP and G-patch domain containing 1 |
| NAT9 | -0.07772596 | N-acetyltransferase 9 (putative) |
| PTBP1 | -0.07766535 | polypyrimidine tract binding protein 1 |
| RPS17 | -0.07754677 | ribosomal protein S17 |
| MAGIX | -0.07750988 | MAGI family member, X-linked |
| CNN1 | -0.07744401 | calponin 1 |
| PLEKHO2 | -0.07730962 | pleckstrin homology domain containing O2 |
| LMBRD1 | -0.07719631 | LMBR1 domain containing 1 |
| TRPC6 | -0.07709881 | transient receptor potential cation channel subfamily C member 6 |
| FBLN2 | -0.07701713 | fibulin 2 |
| SOCS1 | -0.07696706 | suppressor of cytokine signaling 1 |
| MDH1 | -0.07696443 | malate dehydrogenase 1 |
| DHX57 | -0.07680369 | DEAH-box helicase 57 |
| MAP1B | -0.07679315 | microtubule associated protein 1B |
| ANKS1A | -0.07666667 | ankyrin repeat and sterile alpha motif domain containing 1A |
| ZNF341 | -0.07663768 | zinc finger protein 341 |
| CSTL1 | -0.07653228 | cystatin like 1 |
| PQBP1 | -0.07631094 | polyglutamine binding protein 1 |
| POLR2C | -0.07627141 | RNA polymerase II subunit C |
| ATP5G1 | -0.07626087 | ATP synthase, H+ transporting, mitochondrial Fo complex subunit C1 (subunit 9) |
| CEP83 | -0.07620553 | centrosomal protein 83 |
| TMEM44 | -0.07614493 | transmembrane protein 44 |
| GAS5 | -0.07594203 | growth arrest specific 5 (non-protein coding) |
| OLFML3 | -0.07593412 | olfactomedin like 3 |
| DUS1L | -0.07570751 | dihydrouridine synthase 1 like |
| SH3GL1 | -0.07569433 | SH3 domain containing GRB2 like 1, endophilin A2 |
| TXK | -0.07568379 | TXK tyrosine kinase |
| MEGF8 | -0.07566271 | multiple EGF like domains 8 |
| ATPIF1 | -0.07546509 | ATPase inhibitory factor 1 |
| NCKAP1L | -0.07529381 | NCK associated protein 1 like |
| GPC2 | -0.07524638 | glypican 2 |
| MATN3 | -0.07514361 | matrilin 3 |
| MELK | -0.07493808 | maternal embryonic leucine zipper kinase |
| PPP1R12A | -0.07492754 | protein phosphatase 1 regulatory subunit 12A |
| LEAP2 | -0.07486166 | liver enriched antimicrobial peptide 2 |
| GINS3 | -0.074722 | GINS complex subunit 3 |
| SCAF8 | -0.07467984 | SR-related CTD associated factor 8 |
| VASN | -0.07423452 | vasorin |
| EPT1 | -0.07421607 | ethanolaminephosphotransferase 1 |
| STX7 | -0.0741502 | syntaxin 7 |
| ABCC4 | -0.07407378 | ATP binding cassette subfamily C member 4 |
| HSDL1 | -0.07406061 | hydroxysteroid dehydrogenase like 1 |
| NRP1 | -0.07396838 | neuropilin 1 |
| POLRMT | -0.07396047 | RNA polymerase mitochondrial |
| TRPV2 | -0.07381555 | transient receptor potential cation channel subfamily V member 2 |
| EDNRB | -0.07373386 | endothelin receptor type B |
| HIST1H3F | -0.07373123 | histone cluster 1, H3f |
| CBX3 | -0.0736996 | chromobox 3 |
| GK5 | -0.0736917 | glycerol kinase 5 (putative) |
| ABCB9 | -0.07357839 | ATP binding cassette subfamily B member 9 |
| GAPT | -0.07355731 | GRB2-binding adaptor protein, transmembrane |
| CORO1B | -0.07355204 | coronin 1B |
| YTHDF1 | -0.07354677 | YTH N6-methyladenosine RNA binding protein 1 |
| HVCN1 | -0.07345191 | hydrogen voltage gated channel 1 |
| TMEM55B | -0.07332279 | transmembrane protein 55B |
| RAB3IL1 | -0.07330962 | RAB3A interacting protein like 1 |
| RTN4 | -0.07323584 | reticulon 4 |
| PSMB3 | -0.07315942 | proteasome subunit beta 3 |
| PPP1CB | -0.07315679 | protein phosphatase 1 catalytic subunit beta |
| SETD1A | -0.07303557 | SET domain containing 1A |
| XPO6 | -0.07303557 | exportin 6 |
| PMM2 | -0.07270619 | phosphomannomutase 2 |
| TLX2 | -0.07267721 | T-cell leukemia homeobox 2 |
| ESCO2 | -0.07261924 | establishment of sister chromatid cohesion N-acetyltransferase 2 |
| TADA3 | -0.07261397 | transcriptional adaptor 3 |
| UBAC2 | -0.07257971 | UBA domain containing 2 |
| WNT3 | -0.07254282 | Wnt family member 3 |
| DDX54 | -0.07252701 | DEAD-box helicase 54 |
| GTPBP3 | -0.07241634 | GTP binding protein 3 (mitochondrial) |
| FNDC1 | -0.07237945 | fibronectin type III domain containing 1 |
| UBE2M | -0.07226614 | ubiquitin conjugating enzyme E2 M |
| SLC7A3 | -0.07224506 | solute carrier family 7 member 3 |
| HDAC4 | -0.07223979 | histone deacetylase 4 |
| ZNF687 | -0.07221607 | zinc finger protein 687 |
| SFRP2 | -0.07216337 | secreted frizzled related protein 2 |
| SMARCA4 | -0.07211594 | SWI/SNF related, matrix associated, actin dependent regulator of chromatin, subfamily a, member 4 |
| PRR19 | -0.0721054 | proline rich 19 |
| ICOS | -0.07208696 | inducible T-cell costimulator |
| NRG2 | -0.07175231 | neuregulin 2 |
| WBSCR22 | -0.07173123 | Williams-Beuren syndrome chromosome region 22 |
| SLC35C2 | -0.07165744 | solute carrier family 35 member C2 |
| EVA1C | -0.07158893 | eva-1 homolog C |
| DNALI1 | -0.07151779 | dynein axonemal light intermediate chain 1 |
| HSPB8 | -0.07149407 | heat shock protein family B (small) member 8 |
| TAF9B | -0.07145191 | TATA-box binding protein associated factor 9b |
| HIST1H4K | -0.07121739 | histone cluster 1, H4k |
| SAMD1 | -0.07109618 | sterile alpha motif domain containing 1 |
| TMEM201 | -0.07105665 | transmembrane protein 201 |
| JAM3 | -0.07091436 | junctional adhesion molecule 3 |
| ALDOC | -0.07064822 | aldolase, fructose-bisphosphate C |
| MID2 | -0.07064822 | midline 2 |
| FAM92A1 | -0.07060606 | family with sequence similarity 92 member A1 |
| SCRIB | -0.07057971 | scribbled planar cell polarity protein |
| DTYMK | -0.070361 | deoxythymidylate kinase |
| MTX1 | -0.07035046 | metaxin 1 |
| C19orf73 | -0.07035046 | chromosome 19 open reading frame 73 |
| WDR1 | -0.0701581 | WD repeat domain 1 |
| QPCTL | -0.0701054 | glutaminyl-peptide cyclotransferase like |
| CIC | -0.07005797 | capicua transcriptional repressor |
| REG4 | -0.0698946 | regenerating family member 4 |
| MEST | -0.06977602 | mesoderm specific transcript |
| ADAMDEC1 | -0.06976812 | ADAM like decysin 1 |
| TBC1D3F | -0.06967589 | TBC1 domain family member 3F |
| CDCP1 | -0.06951515 | CUB domain containing protein 1 |
| TIMM8A | -0.06935968 | translocase of inner mitochondrial membrane 8 homolog A (yeast) |
| SREBF1 | -0.06932279 | sterol regulatory element binding transcription factor 1 |
| YWHAEP1 | -0.06929117 | tyrosine 3-monooxygenase/tryptophan 5-monooxygenase activation protein epsilon pseudogene 1 |
| CD180 | -0.06906192 | CD180 molecule |
| IPO13 | -0.06901449 | importin 13 |
| AP2S1 | -0.06898024 | adaptor related protein complex 2 sigma 1 subunit |
| ATP1A4 | -0.06874835 | ATPase Na+/K+ transporting subunit alpha 4 |
| STAP1 | -0.06864822 | signal transducing adaptor family member 1 |
| REEP2 | -0.06855072 | receptor accessory protein 2 |
| SLC16A3 | -0.06850593 | solute carrier family 16 member 3 |
| FKBP5 | -0.06845586 | FK506 binding protein 5 |
| LOC101927362 | -0.06826877 | uncharacterized LOC101927362 |
| WHAMMP3 | -0.06813439 | WAS protein homolog associated with actin, golgi membranes and microtubules pseudogene 3 |
| TGOLN2 | -0.06801054 | trans-golgi network protein 2 |
| CENPI | -0.06799209 | centromere protein I |
| E2F3 | -0.06794993 | E2F transcription factor 3 |
| LIN37 | -0.06790514 | lin-37 DREAM MuvB core complex component |
| HYAL3 | -0.06781028 | hyaluronoglucosaminidase 3 |
| MT2A | -0.06774177 | metallothionein 2A |
| ENDOD1 | -0.067639 | endonuclease domain containing 1 |
| FOXD2-AS1 | -0.0671726 | FOXD2 antisense RNA 1 (head to head) |
| RARRES2 | -0.06695916 | retinoic acid receptor responder 2 |
| STAB1 | -0.06673518 | stabilin 1 |
| SOCS7 | -0.06665349 | suppressor of cytokine signaling 7 |
| ZNF787 | -0.06647958 | zinc finger protein 787 |
| CIAPIN1 | -0.06646377 | cytokine induced apoptosis inhibitor 1 |
| DES | -0.06642424 | desmin |
| SRSF7 | -0.06642424 | serine and arginine rich splicing factor 7 |
| GABRD | -0.06630567 | gamma-aminobutyric acid type A receptor delta subunit |
| MRPL20 | -0.06628986 | mitochondrial ribosomal protein L20 |
| SLX4IP | -0.06626087 | SLX4 interacting protein |
| ECD | -0.06625033 | ecdysoneless cell cycle regulator |
| TMEM135 | -0.06591831 | transmembrane protein 135 |
| BCL2L1 | -0.06586561 | BCL2 like 1 |
| SFT2D1 | -0.06579974 | SFT2 domain containing 1 |
| EPB41L2 | -0.06575494 | erythrocyte membrane protein band 4.1 like 2 |
| EVX1-AS | -0.0655863 | EVX1 antisense RNA |
| FAM155B | -0.06544664 | family with sequence similarity 155 member B |
| CCDC94 | -0.06517787 | coiled-coil domain containing 94 |
| MCRS1 | -0.0651278 | microspherule protein 1 |
| KLHL6 | -0.06503557 | kelch like family member 6 |
| SAMD10 | -0.06479315 | sterile alpha motif domain containing 10 |
| ZNF521 | -0.0645112 | zinc finger protein 521 |
| FCGRT | -0.06449802 | Fc fragment of IgG receptor and transporter |
| ELOF1 | -0.06447167 | elongation factor 1 homolog |
| PPP2R5D | -0.06435837 | protein phosphatase 2 regulatory subunit B'delta |
| RORA | -0.06421871 | RAR related orphan receptor A |
| GADD45GIP1 | -0.06418182 | GADD45G interacting protein 1 |
| TSPAN17 | -0.06407642 | tetraspanin 17 |
| STK32C | -0.06394466 | serine/threonine kinase 32C |
| ADAT3 | -0.06391568 | adenosine deaminase, tRNA specific 3 |
| TMEM205 | -0.06373386 | transmembrane protein 205 |
| C1orf54 | -0.06369433 | chromosome 1 open reading frame 54 |
| MTMR3 | -0.06353096 | myotubularin related protein 3 |
| LRWD1 | -0.06340975 | leucine rich repeats and WD repeat domain containing 1 |
| TRMU | -0.06325955 | tRNA 5-methylaminomethyl-2-thiouridylate methyltransferase |
| LMBR1 | -0.06317523 | limb development membrane protein 1 |
| TRIM25 | -0.0631357 | tripartite motif containing 25 |
| SIM2 | -0.06306456 | single-minded family bHLH transcription factor 2 |
| LGALS2 | -0.06306456 | galectin 2 |
| CASC4 | -0.06306192 | cancer susceptibility candidate 4 |
| COL4A6 | -0.06296443 | collagen type IV alpha 6 chain |
| HSPB2 | -0.06290119 | heat shock protein family B (small) member 2 |
| VSIG10 | -0.06283267 | V-set and immunoglobulin domain containing 10 |
| POLR3A | -0.06281686 | RNA polymerase III subunit A |
| MFAP5 | -0.0627747 | microfibrillar associated protein 5 |
| ADORA1 | -0.06256917 | adenosine A1 receptor |
| AMPD1 | -0.06250329 | adenosine monophosphate deaminase 1 |
| BET1 | -0.06249539 | Bet1 golgi vesicular membrane trafficking protein |
| ZNF431 | -0.06240843 | zinc finger protein 431 |
| ZNF428 | -0.06207905 | zinc finger protein 428 |
| BIRC3 | -0.06195257 | baculoviral IAP repeat containing 3 |
| SIRT7 | -0.0618946 | sirtuin 7 |
| NACC1 | -0.06187352 | nucleus accumbens associated 1 |
| HIST3H3 | -0.06181291 | histone cluster 3, H3 |
| MED28 | -0.06176548 | mediator complex subunit 28 |
| SS18L1 | -0.06158103 | SS18L1, nBAF chromatin remodeling complex subunit |
| PGRMC1 | -0.06127009 | progesterone receptor membrane component 1 |
| CNKSR1 | -0.06125165 | connector enhancer of kinase suppressor of Ras 1 |
| SDF4 | -0.06123847 | stromal cell derived factor 4 |
| NELFE | -0.06122793 | negative elongation factor complex member E |
| ING1 | -0.06105138 | inhibitor of growth family member 1 |
| KDELC2 | -0.06098814 | KDEL motif containing 2 |
| NBPF10 | -0.06098551 | neuroblastoma breakpoint family member 10 |
| CST4 | -0.06095916 | cystatin S |
| PCDHB4 | -0.0608722 | protocadherin beta 4 |
| ZNF831 | -0.06081686 | zinc finger protein 831 |
| PMPCB | -0.06077997 | peptidase, mitochondrial processing beta subunit |
| PRKCDBP | -0.06057444 | protein kinase C delta binding protein |
| GPBAR1 | -0.06039526 | G protein-coupled bile acid receptor 1 |
| ZNF646 | -0.06020817 | zinc finger protein 646 |
| GPR18 | -0.0601502 | G protein-coupled receptor 18 |
| PSMD6-AS2 | -0.06006588 | PSMD6 antisense RNA 2 |
| LSM4 | -0.05998682 | LSM4 homolog, U6 small nuclear RNA and mRNA degradation associated |
| ZNF271P | -0.05978393 | zinc finger protein 271, pseudogene |
| RPL27 | -0.05970487 | ribosomal protein L27 |
| SPATC1L | -0.05968379 | spermatogenesis and centriole associated 1-like |
| NOL9 | -0.05957576 | nucleolar protein 9 |
| IGDCC4 | -0.05949144 | immunoglobulin superfamily DCC subclass member 4 |
| KCNK17 | -0.05935705 | potassium two pore domain channel subfamily K member 17 |
| CYTH4 | -0.05926219 | cytohesin 4 |
| EPHB4 | -0.05919104 | EPH receptor B4 |
| COL4A4 | -0.05911726 | collagen type IV alpha 4 chain |
| ST8SIA1 | -0.05911199 | ST8 alpha-N-acetyl-neuraminide alpha-2,8-sialyltransferase 1 |
| ST8SIA1 | -0.05911199 | ST8 alpha-N-acetyl-neuraminide alpha-2,8-sialyltransferase 1 |
| ZNF536 | -0.05909618 | zinc finger protein 536 |
| AADAT | -0.05904348 | aminoadipate aminotransferase |
| EXOSC4 | -0.05888538 | exosome component 4 |
| KPNA1 | -0.05881423 | karyopherin subunit alpha 1 |
| TUBB | -0.05880896 | tubulin beta class I |
| GVINP1 | -0.05874308 | GTPase, very large interferon inducible pseudogene 1 |
| COL16A1 | -0.05870092 | collagen type XVI alpha 1 chain |
| DPYSL2 | -0.05867457 | dihydropyrimidinase like 2 |
| PLA2G15 | -0.05865613 | phospholipase A2 group XV |
| ANGPT2 | -0.05861924 | angiopoietin 2 |
| FBLN7 | -0.05858498 | fibulin 7 |
| KIF3C | -0.0585191 | kinesin family member 3C |
| HLA-DOB | -0.05849012 | major histocompatibility complex, class II, DO beta |
| CACNA1C | -0.05845059 | calcium voltage-gated channel subunit alpha1 C |
| CACNA1C | -0.05845059 | calcium voltage-gated channel subunit alpha1 C |
| NUFIP1 | -0.05841634 | NUFIP1, FMR1 interacting protein 1 |
| EPN1 | -0.05837681 | epsin 1 |
| GIMAP1-GIMAP5///GIMAP1///GIMAP5 | -0.05837418 | GIMAP1-GIMAP5 readthrough///GTPase, IMAP family member 1///GTPase, IMAP family member 5 |
| KNL1 | -0.05835046 | kinetochore scaffold 1 |
| FOXRED1 | -0.05817391 | FAD dependent oxidoreductase domain containing 1 |
| SLC25A15 | -0.05817128 | solute carrier family 25 member 15 |
| RBL2 | -0.05811067 | RB transcriptional corepressor like 2 |
| MGME1 | -0.05788142 | mitochondrial genome maintenance exonuclease 1 |
| SLC12A5 | -0.05782082 | solute carrier family 12 member 5 |
| MTERF4 | -0.05774704 | mitochondrial transcription termination factor 4 |
| EHMT2 | -0.0576469 | euchromatic histone lysine methyltransferase 2 |
| RPLP0 | -0.05740711 | ribosomal protein lateral stalk subunit P0 |
| PROM1 | -0.05740184 | prominin 1 |
| IGHMBP2 | -0.05735968 | immunoglobulin mu binding protein 2 |
| THAP11 | -0.05734914 | THAP domain containing 11 |
| SRP9 | -0.05731489 | signal recognition particle 9 |
| FOXO3B | -0.05731489 | forkhead box O3B pseudogene |
| GLB1L2 | -0.05728854 | galactosidase beta 1 like 2 |
| CLDN9 | -0.05726219 | claudin 9 |
| HACE1 | -0.05711726 | HECT domain and ankyrin repeat containing E3 ubiquitin protein ligase 1 |
| PIAS4 | -0.05687484 | protein inhibitor of activated STAT 4 |
| METTL26 | -0.05679051 | methyltransferase like 26 |
| PTGIR | -0.05648748 | prostaglandin I2 (prostacyclin) receptor (IP) |
| RPS12 | -0.05646113 | ribosomal protein S12 |
| PDXK | -0.05628986 | pyridoxal (pyridoxine, vitamin B6) kinase |
| OTOGL | -0.05620553 | otogelin like |
| MORF4L2-AS1 | -0.05618182 | MORF4L2 antisense RNA 1 |
| KAT2B | -0.05611067 | lysine acetyltransferase 2B |
| GIGYF1 | -0.05606851 | GRB10 interacting GYF protein 1 |
| ZNF155 | -0.05601318 | zinc finger protein 155 |
| GLI1 | -0.05601318 | GLI family zinc finger 1 |
| XRCC2 | -0.05581028 | X-ray repair cross complementing 2 |
| SH2D1A | -0.05579183 | SH2 domain containing 1A |
| IL2RG | -0.05569697 | interleukin 2 receptor subunit gamma |
| HS3ST6 | -0.05558893 | heparan sulfate-glucosamine 3-sulfotransferase 6 |
| COX7A2L | -0.05547299 | cytochrome c oxidase subunit 7A2 like |
| CPSF4L | -0.05545191 | cleavage and polyadenylation specific factor 4 like |
| SPDYE3 | -0.05544401 | speedy/RINGO cell cycle regulator family member E3 |
| RIMBP3 | -0.05540711 | RIMS binding protein 3 |
| MFNG | -0.05532016 | MFNG O-fucosylpeptide 3-beta-N-acetylglucosaminyltransferase |
| PPP6R1 | -0.05523584 | protein phosphatase 6 regulatory subunit 1 |
| KEAP1 | -0.05522266 | kelch like ECH associated protein 1 |
| TBC1D16 | -0.05521212 | TBC1 domain family member 16 |
| ZNF212 | -0.05514097 | zinc finger protein 212 |
| MAP6D1 | -0.05499868 | MAP6 domain containing 1 |
| MRPL58 | -0.05497497 | mitochondrial ribosomal protein L58 |
| POLD2 | -0.05495916 | DAN polymerase delta 2, accessory subunit |
| SIRPG | -0.05484848 | signal regulatory protein gamma |
| ZSWIM1 | -0.05479842 | zinc finger SWIM-type containing 1 |
| MCEE | -0.05477997 | methylmalonyl-CoA epimerase |
| ANKRD44 | -0.05474308 | ankyrin repeat domain 44 |
| ITPKB | -0.05473254 | inositol-trisphosphate 3-kinase B |
| EXOC7 | -0.05472464 | exocyst complex component 7 |
| MAP3K10 | -0.05467194 | mitogen-activated protein kinase kinase kinase 10 |
| MT1E | -0.05464032 | metallothionein 1E |
| C4orf32 | -0.05423715 | chromosome 4 open reading frame 32 |
| UBFD1 | -0.05418709 | ubiquitin family domain containing 1 |
| MAT2A | -0.05417655 | methionine adenosyltransferase 2A |
| C1orf53 | -0.05407642 | chromosome 1 open reading frame 53 |
| TMED1 | -0.05392622 | transmembrane p24 trafficking protein 1 |
| PARD6A | -0.05373123 | par-6 family cell polarity regulator alpha |
| RAN | -0.05352833 | RAN, member RAS oncogene family |
| TNFRSF17 | -0.05340711 | TNF receptor superfamily member 17 |
| DNAJC17 | -0.05339921 | DnaJ heat shock protein family (Hsp40) member C17 |
| TRAPPC11 | -0.05332543 | trafficking protein particle complex 11 |
| C11orf24 | -0.05329381 | chromosome 11 open reading frame 24 |
| NEIL2 | -0.05316733 | nei like DNA glycosylase 2 |
| ARHGAP11A | -0.05290909 | Rho GTPase activating protein 11A |
| ZCCHC24 | -0.05277207 | zinc finger CCHC-type containing 24 |
| UBOX5 | -0.05277207 | U-box domain containing 5 |
| RCC2 | -0.05254282 | regulator of chromosome condensation 2 |
| RASAL1 | -0.0525112 | RAS protein activator like 1 |
| RPS14 | -0.05244532 | ribosomal protein S14 |
| LIX1L | -0.05233202 | limb and CNS expressed 1 like |
| S1PR3 | -0.05230567 | sphingosine-1-phosphate receptor 3 |
| CYB561 | -0.05229249 | cytochrome b561 |
| ZNF705A | -0.05214756 | zinc finger protein 705A |
| TSC22D3 | -0.05213966 | TSC22 domain family member 3 |
| CYYR1 | -0.05207115 | cysteine and tyrosine rich 1 |
| SEC14L2 | -0.05202635 | SEC14 like lipid binding 2 |
| GNA14 | -0.0519552 | G protein subunit alpha 14 |
| TAF5L | -0.05186561 | TATA-box binding protein associated factor 5 like |
| ZNF200 | -0.05184453 | zinc finger protein 200 |
| MBLAC2 | -0.05171805 | metallo-beta-lactamase domain containing 2 |
| IMPDH1 | -0.05169433 | inosine monophosphate dehydrogenase 1 |
| KIAA1147 | -0.05158366 | KIAA1147 |
| SGMS1 | -0.05158103 | sphingomyelin synthase 1 |
| PCDH17 | -0.05124111 | protocadherin 17 |
| SNX6 | -0.0510224 | sorting nexin 6 |
| BATF3 | -0.05100395 | basic leucine zipper ATF-like transcription factor 3 |
| TRPC4AP | -0.05089592 | transient receptor potential cation channel subfamily C member 4 associated protein |
| MPDZ | -0.05074308 | multiple PDZ domain crumbs cell polarity complex component |
| FBXO5 | -0.05074308 | F-box protein 5 |
| PARP2 | -0.05072727 | poly(ADP-ribose) polymerase 2 |
| ANKRD54 | -0.05063505 | ankyrin repeat domain 54 |
| CNTNAP1 | -0.05061397 | contactin associated protein 1 |
| PVRIG | -0.05055336 | poliovirus receptor related immunoglobulin domain containing |
| LOC105369266 | -0.05037154 | uncharacterized LOC105369266 |
| FAM159A | -0.05024242 | family with sequence similarity 159 member A |
| TBC1D22A | -0.05014229 | TBC1 domain family member 22A |
| ARHGAP11B | -0.05012121 | Rho GTPase activating protein 11B |
| TPM3 | -0.04997628 | tropomyosin 3 |
| COL13A1 | -0.04986298 | collagen type XIII alpha 1 chain |
| SH3BP5L | -0.04935178 | SH3 binding domain protein 5 like |
| PROCA1 | -0.04923584 | protein interacting with cyclin A1 |
| CDR2L | -0.04913043 | cerebellar degeneration related protein 2 like |
| PEX10 | -0.049083 | peroxisomal biogenesis factor 10 |
| CD1C | -0.04896443 | CD1c molecule |
| PNKP | -0.04882477 | polynucleotide kinase 3'-phosphatase |
| PTOV1 | -0.04874308 | prostate tumor overexpressed 1 |
| HSPA8 | -0.04864295 | heat shock protein family A (Hsp70) member 8 |
| MGC57346 | -0.04848221 | ADP-ribosylation factor pseudogene |
| CDAN1 | -0.04840843 | codanin 1 |
| PDCD1 | -0.04833202 | programmed cell death 1 |
| ZNF579 | -0.04831621 | zinc finger protein 579 |
| DLD | -0.04831357 | dihydrolipoamide dehydrogenase |
| PRR5L | -0.04827668 | proline rich 5 like |
| FICD | -0.04805797 | FIC domain containing |
| SMG5 | -0.04797101 | SMG5, nonsense mediated mRNA decay factor |
| RBM28 | -0.0479552 | RNA binding motif protein 28 |
| PARS2 | -0.04775494 | prolyl-tRNA synthetase 2, mitochondrial (putative) |
| FAM98B | -0.0477444 | family with sequence similarity 98 member B |
| NDRG4 | -0.04773123 | NDRG family member 4 |
| VPS4B | -0.04770487 | vacuolar protein sorting 4 homolog B |
| GATAD2A | -0.0476917 | GATA zinc finger domain containing 2A |
| RAB40C | -0.04761528 | RAB40C, member RAS oncogene family |
| CDC6 | -0.04752042 | cell division cycle 6 |
| KXD1 | -0.04745718 | KxDL motif containing 1 |
| TAB1 | -0.04738603 | TGF-beta activated kinase 1 (MAP3K7) binding protein 1 |
| NOC2LP2 | -0.04734914 | NOC2 like nucleolar associated transcriptional repressor pseudogene 2 |
| MIF | -0.0472332 | macrophage migration inhibitory factor (glycosylation-inhibiting factor) |
| STAC3 | -0.0471278 | SH3 and cysteine rich domain 3 |
| PRAMEF8 | -0.04705138 | PRAME family member 8 |
| PRAMEF8 | -0.04705138 | PRAME family member 8 |
| SNORA10 | -0.04703294 | small nucleolar RNA, H/ACA box 10 |
| ZNF317 | -0.04695652 | zinc finger protein 317 |
| SLC30A7 | -0.04668511 | solute carrier family 30 member 7 |
| DAZAP2 | -0.04656126 | DAZ associated protein 2 |
| TACO1 | -0.04653491 | translational activator of cytochrome c oxidase I |
| TGM2 | -0.04647694 | transglutaminase 2 |
| TUSC1 | -0.04621871 | tumor suppressor candidate 1 |
| LMCD1 | -0.04613966 | LIM and cysteine rich domains 1 |
| COX7A2 | -0.0460975 | cytochrome c oxidase subunit 7A2 |
| NOCT | -0.0459473 | nocturnin |
| MYCT1 | -0.04591304 | myc target 1 |
| KRTCAP2 | -0.04583136 | keratinocyte associated protein 2 |
| YIPF2 | -0.04582345 | Yip1 domain family member 2 |
| PXK | -0.04577866 | PX domain containing serine/threonine kinase like |
| S1PR4 | -0.04575758 | sphingosine-1-phosphate receptor 4 |
| EIF4B | -0.04574177 | eukaryotic translation initiation factor 4B |
| ARHGEF3 | -0.04573386 | Rho guanine nucleotide exchange factor 3 |
| SIRT1 | -0.045639 | sirtuin 1 |
| IRF2BP1 | -0.04557839 | interferon regulatory factor 2 binding protein 1 |
| SETDB1 | -0.0454361 | SET domain bifurcated 1 |
| LIMS2 | -0.0453834 | LIM zinc finger domain containing 2 |
| TSPAN33 | -0.04529117 | tetraspanin 33 |
| GPR162 | -0.04520949 | G protein-coupled receptor 162 |
| IRX1 | -0.04518314 | iroquois homeobox 1 |
| PFDN6 | -0.04503294 | prefoldin subunit 6 |
| PPA1 | -0.04501976 | pyrophosphatase (inorganic) 1 |
| TNRC6C | -0.04500395 | trinucleotide repeat containing 6C |
| TNRC6C | -0.04500395 | trinucleotide repeat containing 6C |
| SCARF2 | -0.04493281 | scavenger receptor class F member 2 |
| MYOZ3 | -0.04491173 | myozenin 3 |
| APOBEC3C | -0.04478788 | apolipoprotein B mRNA editing enzyme catalytic subunit 3C |
| NOP16 | -0.04476153 | NOP16 nucleolar protein |
| ZNF2 | -0.04464822 | zinc finger protein 2 |
| FAM171A1 | -0.04462451 | family with sequence similarity 171 member A1 |
| LINC00877 | -0.04458762 | long intergenic non-protein coding RNA 877 |
| EPS15L1 | -0.04454282 | epidermal growth factor receptor pathway substrate 15 like 1 |
| SETBP1 | -0.04453491 | SET binding protein 1 |
| IPO8 | -0.04449539 | importin 8 |
| ZC3H18 | -0.04443742 | zinc finger CCCH-type containing 18 |
| TMEM176B | -0.04413966 | transmembrane protein 176B |
| MYOM1 | -0.04395257 | myomesin 1 |
| LGI4 | -0.04391831 | leucine rich repeat LGI family member 4 |
| COL6A4P2 | -0.04387615 | collagen type VI alpha 4 pseudogene 2 |
| COL6A4P2 | -0.04387615 | collagen type VI alpha 4 pseudogene 2 |
| POLR2H | -0.04376548 | RNA polymerase II subunit H |
| TBC1D2B | -0.04367852 | TBC1 domain family member 2B |
| CTSZ | -0.04367325 | cathepsin Z |
| CHMP4A | -0.04362319 | charged multivesicular body protein 4A |
| PKDCC | -0.04356522 | protein kinase domain containing, cytoplasmic |
| LRRC26 | -0.04356258 | leucine rich repeat containing 26 |
| PTEN | -0.04354414 | phosphatase and tensin homolog |
| DET1 | -0.04352042 | de-etiolated homolog 1 (Arabidopsis) |
| CST7 | -0.0434809 | cystatin F |
| RNF125 | -0.04345455 | ring finger protein 125 |
| RNF125 | -0.04345455 | ring finger protein 125 |
| NAPSB | -0.04337022 | napsin B aspartic peptidase, pseudogene |
| DTX2 | -0.04314888 | deltex E3 ubiquitin ligase 2 |
| CCDC43 | -0.04311989 | coiled-coil domain containing 43 |
| SCG5 | -0.0428643 | secretogranin V |
| FZD8 | -0.0428643 | frizzled class receptor 8 |
| C20orf96 | -0.04279051 | chromosome 20 open reading frame 96 |
| KIAA1524 | -0.04274572 | KIAA1524 |
| NSD1 | -0.04269302 | nuclear receptor binding SET domain protein 1 |
| HYMAI | -0.0426693 | hydatidiform mole associated and imprinted (non-protein coding) |
| RPL11 | -0.0426693 | ribosomal protein L11 |
| BCAP31 | -0.04262451 | B-cell receptor-associated protein 31 |
| SMARCD1 | -0.04256653 | SWI/SNF related, matrix associated, actin dependent regulator of chromatin, subfamily d, member 1 |
| CRIM1 | -0.04247694 | cysteine rich transmembrane BMP regulator 1 |
| COA3 | -0.04237154 | cytochrome c oxidase assembly factor 3 |
| JTB | -0.04233202 | jumping translocation breakpoint |
| CABP1 | -0.04223188 | calcium binding protein 1 |
| NTRK3 | -0.04221607 | neurotrophic receptor tyrosine kinase 3 |
| NTRK3 | -0.04221607 | neurotrophic receptor tyrosine kinase 3 |
| RPL32 | -0.04212648 | ribosomal protein L32 |
| METTL1 | -0.04211067 | methyltransferase like 1 |
| SPNS3 | -0.04198682 | sphingolipid transporter 3 (putative) |
| LOC153577 | -0.04186825 | uncharacterized LOC153577 |
| PSMG3 | -0.04178393 | proteasome assembly chaperone 3 |
| PDX1 | -0.04173386 | pancreatic and duodenal homeobox 1 |
| CAMSAP3 | -0.04172069 | calmodulin regulated spectrin associated protein family member 3 |
| G3BP1 | -0.04170487 | G3BP stress granule assembly factor 1 |
| CCNE2 | -0.04167589 | cyclin E2 |
| C1orf35 | -0.04149934 | chromosome 1 open reading frame 35 |
| LOC105377443 | -0.0414361 | uncharacterized LOC105377443 |
| LINC01318 | -0.04128854 | long intergenic non-protein coding RNA 1318 |
| ZBTB16 | -0.04118577 | zinc finger and BTB domain containing 16 |
| BRCA1 | -0.04108037 | BRCA1, DNA repair associated |
| APH1A | -0.04106456 | aph-1 homolog A, gamma-secretase subunit |
| IL10RA | -0.04105402 | interleukin 10 receptor subunit alpha |
| IKZF3 | -0.04089855 | IKAROS family zinc finger 3 |
| PIP4K2C | -0.0408643 | phosphatidylinositol-5-phosphate 4-kinase type 2 gamma |
| CHID1 | -0.04069038 | chitinase domain containing 1 |
| BMS1 | -0.04054282 | BMS1, ribosome biogenesis factor |
| CD34 | -0.04052964 | CD34 molecule |
| DACH1 | -0.04038999 | dachshund family transcription factor 1 |
| KATNB1 | -0.04038735 | katanin regulatory subunit B1 |
| CCDC58 | -0.04033729 | coiled-coil domain containing 58 |
| CADM3 | -0.04028458 | cell adhesion molecule 3 |
| SHBG | -0.04024769 | sex hormone binding globulin |
| SMC4 | -0.04022398 | structural maintenance of chromosomes 4 |
| ABHD14B | -0.0402108 | abhydrolase domain containing 14B |
| SASH1 | -0.04010804 | SAM and SH3 domain containing 1 |
| SLC35A2 | -0.04005007 | solute carrier family 35 member A2 |
| GCAT | -0.03986825 | glycine C-acetyltransferase |
| SLX4 | -0.03971014 | SLX4 structure-specific endonuclease subunit |
| C2orf88 | -0.03968906 | chromosome 2 open reading frame 88 |
| XK | -0.03941765 | X-linked Kx blood group |
| ZNF296 | -0.03931225 | zinc finger protein 296 |
| REEP3 | -0.03930962 | receptor accessory protein 3 |
| DBF4B | -0.03919895 | DBF4 zinc finger B |
| CD5 | -0.03916206 | CD5 molecule |
| YEATS2 | -0.03909881 | YEATS domain containing 2 |
| KCNA3 | -0.03906719 | potassium voltage-gated channel subfamily A member 3 |
| GPR143 | -0.0388722 | G protein-coupled receptor 143 |
| KIFC1 | -0.03882477 | kinesin family member C1 |
| AMDHD2 | -0.03877734 | amidohydrolase domain containing 2 |
| TCEAL4 | -0.03856126 | transcription elongation factor A like 4 |
| HMGA1 | -0.03852701 | high mobility group AT-hook 1 |
| UST | -0.0385112 | uronyl 2-sulfotransferase |
| RPS6KA2 | -0.03817391 | ribosomal protein S6 kinase A2 |
| KMT5C | -0.03801845 | lysine methyltransferase 5C |
| RCVRN | -0.03801054 | recoverin |
| ATAD1 | -0.03795784 | ATPase family, AAA domain containing 1 |
| TEX11 | -0.0378498 | testis expressed 11 |
| FZD7 | -0.03769433 | frizzled class receptor 7 |
| PEMT | -0.03767062 | phosphatidylethanolamine N-methyltransferase |
| SLAMF6 | -0.03752569 | SLAM family member 6 |
| PDE1A | -0.03748617 | phosphodiesterase 1A |
| CD7 | -0.03740975 | CD7 molecule |
| ZNF260 | -0.03714888 | zinc finger protein 260 |
| GNG7 | -0.03701186 | G protein subunit gamma 7 |
| TBCA | -0.03693808 | tubulin folding cofactor A |
| RCSD1 | -0.03688801 | RCSD domain containing 1 |
| EPO | -0.03684848 | erythropoietin |
| SMPD2 | -0.03678524 | sphingomyelin phosphodiesterase 2 |
| LOC646938 | -0.03675362 | TBC1 domain family member 2B pseudogene |
| SOX8 | -0.03674572 | SRY-box 8 |
| CTPS2 | -0.03668248 | CTP synthase 2 |
| SLC52A2 | -0.0365639 | solute carrier family 52 member 2 |
| HMGCR | -0.03654018 | 3-hydroxy-3-methylglutaryl-CoA reductase |
| PARVG | -0.03643478 | parvin gamma |
| CDK5 | -0.03637154 | cyclin dependent kinase 5 |
| LOC101928475 | -0.03627668 | uncharacterized LOC101928475 |
| VAC14 | -0.03619236 | Vac14, PIKFYVE complex component |
| SLC12A2 | -0.03608959 | solute carrier family 12 member 2 |
| SMYD5 | -0.03599209 | SMYD family member 5 |
| KDM5C | -0.03591304 | lysine demethylase 5C |
| MRGBP | -0.03589723 | MRG/MORF4L binding protein |
| GSTCD | -0.03586825 | glutathione S-transferase C-terminal domain containing |
| PTRH2 | -0.03586034 | peptidyl-tRNA hydrolase 2 |
| CPZ | -0.03583136 | carboxypeptidase Z |
| MAP1S | -0.03576812 | microtubule associated protein 1S |
| SOX17 | -0.03566008 | SRY-box 17 |
| ORC5 | -0.03547036 | origin recognition complex subunit 5 |
| LIMK1 | -0.03547036 | LIM domain kinase 1 |
| WRAP73 | -0.03546509 | WD repeat containing, antisense to TP73 |
| ARHGAP15 | -0.03536495 | Rho GTPase activating protein 15 |
| PRSS45 | -0.03528854 | protease, serine 45 |
| ATG9B | -0.03527273 | autophagy related 9B |
| GPATCH1 | -0.03525428 | G-patch domain containing 1 |
| PGF | -0.03519368 | placental growth factor |
| DHX33 | -0.03515152 | DEAH-box helicase 33 |
| TDP1 | -0.03510935 | tyrosyl-DNA phosphodiesterase 1 |
| RPL36AL | -0.03504084 | ribosomal protein L36a like |
| TSHZ1 | -0.03501713 | teashirt zinc finger homeobox 1 |
| PAPOLG | -0.03493808 | poly(A) polymerase gamma |
| EIF3G | -0.03484848 | eukaryotic translation initiation factor 3 subunit G |
| RXRG | -0.03478788 | retinoid X receptor gamma |
| LOXL3 | -0.03465613 | lysyl oxidase like 3 |
| SFRP1 | -0.03464822 | secreted frizzled related protein 1 |
| LY96 | -0.03452701 | lymphocyte antigen 96 |
| NDNF | -0.03446377 | neuron derived neurotrophic factor |
| UPF1 | -0.03434519 | UPF1, RNA helicase and ATPase |
| RAB4A | -0.03429776 | RAB4A, member RAS oncogene family |
| HDC | -0.03422925 | histidine decarboxylase |
| CD28 | -0.0342029 | CD28 molecule |
| MYL6B | -0.03411331 | myosin light chain 6B |
| LOC105374297 | -0.03401845 | uncharacterized LOC105374297 |
| AMER3 | -0.03385771 | APC membrane recruitment protein 3 |
| HNRNPK | -0.03368906 | heterogeneous nuclear ribonucleoprotein K |
| GINM1 | -0.03367852 | glycoprotein integral membrane 1 |
| TMEM104 | -0.03350461 | transmembrane protein 104 |
| RER1 | -0.03344401 | retention in endoplasmic reticulum sorting receptor 1 |
| NFATC4 | -0.03337022 | nuclear factor of activated T-cells 4 |
| ATP5F1 | -0.03330698 | ATP synthase, H+ transporting, mitochondrial Fo complex subunit B1 |
| ZNF446 | -0.0332332 | zinc finger protein 446 |
| MIR133A1HG | -0.0332253 | MIR133A1 host gene |
| CCDC116 | -0.03318841 | coiled-coil domain containing 116 |
| RGS12 | -0.03315152 | regulator of G-protein signaling 12 |
| CCL5 | -0.03313834 | C-C motif chemokine ligand 5 |
| AATF | -0.03309354 | apoptosis antagonizing transcription factor |
| GRK6 | -0.03308564 | G protein-coupled receptor kinase 6 |
| ASIC4 | -0.03303294 | acid sensing ion channel subunit family member 4 |
| CPEB4 | -0.0329697 | cytoplasmic polyadenylation element binding protein 4 |
| ZNF696 | -0.03289065 | zinc finger protein 696 |
| HIST1H3C | -0.03287747 | histone cluster 1, H3c |
| GMEB1 | -0.03282213 | glucocorticoid modulatory element binding protein 1 |
| FSCN1 | -0.03276943 | fascin actin-bundling protein 1 |
| TXLNG | -0.03262714 | taxilin gamma |
| FAM83H | -0.03257971 | family with sequence similarity 83 member H |
| FDPS | -0.03242951 | farnesyl diphosphate synthase |
| TNFAIP8L2 | -0.03233992 | TNF alpha induced protein 8 like 2 |
| MAP6 | -0.03216074 | microtubule associated protein 6 |
| NKIRAS1 | -0.0321581 | NFKB inhibitor interacting Ras like 1 |
| PAK1IP1 | -0.03213966 | PAK1 interacting protein 1 |
| FAM26F | -0.0320975 | family with sequence similarity 26 member F |
| MMGT1 | -0.03205534 | membrane magnesium transporter 1 |
| UQCR11 | -0.03173386 | ubiquinol-cytochrome c reductase, complex III subunit XI |
| DOK2 | -0.0313307 | docking protein 2 |
| SLAMF1 | -0.03131752 | signaling lymphocytic activation molecule family member 1 |
| LINC01152 | -0.03127273 | long intergenic non-protein coding RNA 1152 |
| LONRF1 | -0.03126482 | LON peptidase N-terminal domain and ring finger 1 |
| USP9X | -0.03118841 | ubiquitin specific peptidase 9, X-linked |
| DSG2 | -0.03113307 | desmoglein 2 |
| ADAM28 | -0.03106192 | ADAM metallopeptidase domain 28 |
| ADAM28 | -0.03106192 | ADAM metallopeptidase domain 28 |
| GMPPB | -0.03104875 | GDP-mannose pyrophosphorylase B |
| RPS9 | -0.03098814 | ribosomal protein S9 |
| BCAN | -0.03090382 | brevican |
| CATSPER1 | -0.03086166 | cation channel sperm associated 1 |
| FSCB | -0.03084321 | fibrous sheath CABYR binding protein |
| HIST1H2AH | -0.03064559 | histone cluster 1, H2ah |
| PPM1A | -0.0306087 | protein phosphatase, Mg2+/Mn2+ dependent 1A |
| PPM1A | -0.0306087 | protein phosphatase, Mg2+/Mn2+ dependent 1A |
| MFSD12 | -0.03059025 | major facilitator superfamily domain containing 12 |
| UTRN | -0.03036891 | utrophin |
| FTX | -0.03033465 | FTX transcript, XIST regulator (non-protein coding) |
| ABCG1 | -0.0302556 | ATP binding cassette subfamily G member 1 |
| HIST1H1B | -0.03017391 | histone cluster 1, H1b |
| CCDC89 | -0.03015547 | coiled-coil domain containing 89 |
| TMEM161A | -0.03015547 | transmembrane protein 161A |
| COL14A1 | -0.03001845 | collagen type XIV alpha 1 chain |
| LY9 | -0.02997628 | lymphocyte antigen 9 |
| UBALD1 | -0.02990514 | UBA like domain containing 1 |
| PYHIN1 | -0.02989196 | pyrin and HIN domain family member 1 |
| P2RX7 | -0.02986034 | purinergic receptor P2X 7 |
| DDX39B | -0.02984717 | DEAD-box helicase 39B |
| DNAJB6 | -0.0297444 | DnaJ heat shock protein family (Hsp40) member B6 |
| GRAP2 | -0.02972332 | GRB2-related adaptor protein 2 |
| RABEP2 | -0.0296996 | rabaptin, RAB GTPase binding effector protein 2 |
| ANP32A | -0.02967062 | acidic nuclear phosphoprotein 32 family member A |
| LINC01476 | -0.02942029 | long intergenic non-protein coding RNA 1476 |
| ADAMTS5 | -0.0293386 | ADAM metallopeptidase with thrombospondin type 1 motif 5 |
| RDM1 | -0.02931489 | RAD52 motif containing 1 |
| SERPINA3 | -0.02920422 | serpin family A member 3 |
| EFCAB3 | -0.02915942 | EF-hand calcium binding domain 3 |
| CRY1 | -0.02909354 | cryptochrome circadian clock 1 |
| SCAMP3 | -0.02908827 | secretory carrier membrane protein 3 |
| NAALADL1 | -0.02899868 | N-acetylated alpha-linked acidic dipeptidase-like 1 |
| CH25H | -0.02883794 | cholesterol 25-hydroxylase |
| ZNF616 | -0.02883531 | zinc finger protein 616 |
| FASTK | -0.02883004 | Fas activated serine/threonine kinase |
| TBX4 | -0.02878261 | T-box 4 |
| PPM1L | -0.02876416 | protein phosphatase, Mg2+/Mn2+ dependent 1L |
| AKIRIN2 | -0.02875626 | akirin 2 |
| SREK1IP1 | -0.02870883 | SREK1 interacting protein 1 |
| ROR2 | -0.02868511 | receptor tyrosine kinase like orphan receptor 2 |
| GSG2 | -0.02859289 | germ cell associated 2, haspin |
| TRMT1 | -0.02856126 | tRNA methyltransferase 1 |
| WNT2B | -0.02842951 | Wnt family member 2B |
| ELANE | -0.02837681 | elastase, neutrophil expressed |
| IPO5 | -0.02832411 | importin 5 |
| SAMD5 | -0.02831621 | sterile alpha motif domain containing 5 |
| PLEKHF2 | -0.02830303 | pleckstrin homology and FYVE domain containing 2 |
| AP5B1 | -0.02818445 | adaptor related protein complex 5 beta 1 subunit |
| PTPRU | -0.02805534 | protein tyrosine phosphatase, receptor type U |
| CHDH | -0.02802899 | choline dehydrogenase |
| COL6A5 | -0.02799209 | collagen type VI alpha 5 chain |
| TUBB8 | -0.02798682 | tubulin beta 8 class VIII |
| RC3H1 | -0.02794466 | ring finger and CCCH-type domains 1 |
| TIFAB | -0.0277444 | TIFA inhibitor |
| SH2B2 | -0.02773123 | SH2B adaptor protein 2 |
| TIMP2 | -0.02771014 | TIMP metallopeptidase inhibitor 2 |
| ZNF546 | -0.02763109 | zinc finger protein 546 |
| MTA1 | -0.02758893 | metastasis associated 1 |
| ASF1A | -0.02741765 | anti-silencing function 1A histone chaperone |
| IRF4 | -0.02729644 | interferon regulatory factor 4 |
| LOC105371763 | -0.02728063 | uncharacterized LOC105371763 |
| PTGDR2 | -0.02721739 | prostaglandin D2 receptor 2 |
| PYDC2 | -0.02713834 | pyrin domain containing 2 |
| HLA-DPB1 | -0.02709881 | major histocompatibility complex, class II, DP beta 1 |
| GTF2H5 | -0.02707773 | general transcription factor IIH subunit 5 |
| LAMA2 | -0.0270751 | laminin subunit alpha 2 |
| PLXNA3 | -0.02690119 | plexin A3 |
| ZDHHC9 | -0.02690119 | zinc finger DHHC-type containing 9 |
| FAM83A-AS1 | -0.02689065 | FAM83A antisense RNA 1 |
| SCIMP | -0.02687484 | SLP adaptor and CSK interacting membrane protein |
| HNRNPR | -0.02683267 | heterogeneous nuclear ribonucleoprotein R |
| AP2A1 | -0.02682213 | adaptor related protein complex 2 alpha 1 subunit |
| WDR18 | -0.02652964 | WD repeat domain 18 |
| MVK | -0.02649275 | mevalonate kinase |
| TMEM150C | -0.02629776 | transmembrane protein 150C |
| MFSD3 | -0.02629513 | major facilitator superfamily domain containing 3 |
| NKIRAS2 | -0.0262029 | NFKB inhibitor interacting Ras like 2 |
| RAVER1 | -0.02608959 | ribonucleoprotein, PTB binding 1 |
| NKAPL | -0.02604216 | NFKB activating protein like |
| AMZ1 | -0.02591304 | archaelysin family metallopeptidase 1 |
| SFXN5 | -0.0258419 | sideroflexin 5 |
| CTU2 | -0.02572332 | cytosolic thiouridylase subunit 2 |
| RPS19 | -0.02567852 | ribosomal protein S19 |
| ZNF106 | -0.02559947 | zinc finger protein 106 |
| CDKN1C | -0.02545455 | cyclin dependent kinase inhibitor 1C |
| DEFB128 | -0.02543083 | defensin beta 128 |
| PPARD | -0.02542556 | peroxisome proliferator activated receptor delta |
| GAB3 | -0.02535178 | GRB2 associated binding protein 3 |
| TMSB10 | -0.02530962 | thymosin beta 10 |
| NTF3 | -0.02527536 | neurotrophin 3 |
| SYMPK | -0.0252332 | symplekin |
| B3GALNT1 | -0.0251805 | beta-1,3-N-acetylgalactosaminyltransferase 1 (globoside blood group) |
| VTI1A | -0.02516206 | vesicle transport through interaction with t-SNAREs 1A |
| AXL | -0.02500922 | AXL receptor tyrosine kinase |
| RAC3 | -0.02486166 | ras-related C3 botulinum toxin substrate 3 (rho family, small GTP binding protein Rac3) |
| MEOX1 | -0.02478261 | mesenchyme homeobox 1 |
| HDAC7 | -0.024722 | histone deacetylase 7 |
| SSC4D | -0.02463241 | scavenger receptor cysteine rich family member with 4 domains |
| MT1A | -0.02455072 | metallothionein 1A |
| C1QL1 | -0.02447694 | complement C1q like 1 |
| MLH3 | -0.02438735 | mutL homolog 3 |
| TFR2 | -0.02432938 | transferrin receptor 2 |
| WNT7A | -0.02419499 | Wnt family member 7A |
| C1orf132 | -0.02417128 | chromosome 1 open reading frame 132 |
| PDAP1 | -0.02399736 | PDGFA associated protein 1 |
| C17orf82 | -0.02398419 | chromosome 17 open reading frame 82 |
| OR10D1P | -0.02391831 | olfactory receptor family 10 subfamily D member 1 pseudogene |
| ARHGEF17 | -0.02386298 | Rho guanine nucleotide exchange factor 17 |
| CHI3L2 | -0.02376021 | chitinase 3 like 2 |
| FCRL1 | -0.02375758 | Fc receptor like 1 |
| PEX26 | -0.02369433 | peroxisomal biogenesis factor 26 |
| LOC286437 | -0.02350988 | uncharacterized LOC286437 |
| FBXL6 | -0.02328327 | F-box and leucine rich repeat protein 6 |
| RDH14 | -0.02323584 | retinol dehydrogenase 14 (all-trans/9-cis/11-cis) |
| HSP90AA1 | -0.02322793 | heat shock protein 90 alpha family class A member 1 |
| TRUB1 | -0.02320685 | TruB pseudouridine synthase family member 1 |
| CEP72 | -0.02316469 | centrosomal protein 72 |
| EIF4E3 | -0.02313043 | eukaryotic translation initiation factor 4E family member 3 |
| ANKRD29 | -0.02308564 | ankyrin repeat domain 29 |
| DCAF1 | -0.02293281 | DDB1 and CUL4 associated factor 1 |
| ZBTB32 | -0.02291963 | zinc finger and BTB domain containing 32 |
| NAT14 | -0.0228195 | N-acetyltransferase 14 (putative) |
| AKT1S1 | -0.0227668 | AKT1 substrate 1 |
| ITGA1 | -0.02275099 | integrin subunit alpha 1 |
| RAD9A | -0.02273518 | RAD9 checkpoint clamp component A |
| S100Z | -0.02263768 | S100 calcium binding protein Z |
| HMGCLL1 | -0.02260343 | 3-hydroxymethyl-3-methylglutaryl-CoA lyase like 1 |
| C12orf4 | -0.02250593 | chromosome 12 open reading frame 4 |
| MMS22L | -0.02244269 | MMS22 like, DNA repair protein |
| NELFA | -0.02242688 | negative elongation factor complex member A |
| MPHOSPH10 | -0.02231621 | M-phase phosphoprotein 10 |
| CTSG | -0.02224506 | cathepsin G |
| CIRBP | -0.02218182 | cold inducible RNA binding protein |
| ZNF580 | -0.02217918 | zinc finger protein 580 |
| TRPA1 | -0.02198419 | transient receptor potential cation channel subfamily A member 1 |
| SCRN1 | -0.02176548 | secernin 1 |
| TMEM18 | -0.02170487 | transmembrane protein 18 |
| SPPL2C | -0.02164954 | signal peptide peptidase like 2C |
| FAM198B | -0.02157839 | family with sequence similarity 198 member B |
| ADAMTS2 | -0.02132279 | ADAM metallopeptidase with thrombospondin type 1 motif 2 |
| ITGA5 | -0.02130435 | integrin subunit alpha 5 |
| PDE2A | -0.02126746 | phosphodiesterase 2A |
| GLTSCR1 | -0.0212253 | glioma tumor suppressor candidate region gene 1 |
| C3orf70 | -0.02115152 | chromosome 3 open reading frame 70 |
| HCST | -0.02110408 | hematopoietic cell signal transducer |
| PGM5P2 | -0.02103294 | phosphoglucomutase 5 pseudogene 2 |
| BRD4 | -0.02101186 | bromodomain containing 4 |
| SNORD38A | -0.02094335 | small nucleolar RNA, C/D box 38A |
| FAM78A | -0.02091173 | family with sequence similarity 78 member A |
| GPR137 | -0.02079842 | G protein-coupled receptor 137 |
| LAG3 | -0.0207141 | lymphocyte activating 3 |
| LOC102723604 | -0.02056917 | uncharacterized LOC102723604 |
| CMTM4 | -0.02052174 | CKLF like MARVEL transmembrane domain containing 4 |
| CMTM4 | -0.02052174 | CKLF like MARVEL transmembrane domain containing 4 |
| PIAS1 | -0.0204585 | protein inhibitor of activated STAT 1 |
| SCARA3 | -0.02041897 | scavenger receptor class A member 3 |
| LOC105374546 | -0.02032411 | uncharacterized LOC105374546 |
| TTYH1 | -0.02032148 | tweety family member 1 |
| EBI3 | -0.02017391 | Epstein-Barr virus induced 3 |
| UPF3B | -0.0200527 | UPF3 regulator of nonsense transcripts homolog B (yeast) |
| AKT1 | -0.01986825 | AKT serine/threonine kinase 1 |
| MCOLN1 | -0.0197971 | mucolipin 1 |
| LMOD1 | -0.01969433 | leiomodin 1 |
| HPS1 | -0.01961001 | HPS1, biogenesis of lysosomal organelles complex 3 subunit 1 |
| ARPC5L | -0.01954941 | actin related protein 2/3 complex subunit 5 like |
| TUB | -0.01954414 | tubby bipartite transcription factor |
| CLEC10A | -0.01951252 | C-type lectin domain family 10 member A |
| BOC | -0.01949407 | BOC cell adhesion associated, oncogene regulated |
| ABCB4 | -0.01934914 | ATP binding cassette subfamily B member 4 |
| BFSP2 | -0.01931752 | beaded filament structural protein 2 |
| DYRK1B | -0.01927273 | dual specificity tyrosine phosphorylation regulated kinase 1B |
| ZC3HAV1L | -0.01926219 | zinc finger CCCH-type containing, antiviral 1 like |
| CTGF | -0.01923057 | connective tissue growth factor |
| KIFAP3 | -0.01911726 | kinesin associated protein 3 |
| ZMYND19 | -0.01905665 | zinc finger MYND-type containing 19 |
| PSMC6 | -0.01895125 | proteasome 26S subunit, ATPase 6 |
| TLE6 | -0.01890909 | transducin like enhancer of split 6 |
| STOML3 | -0.01881423 | stomatin like 3 |
| NUTF2 | -0.01869829 | nuclear transport factor 2 |
| CMBL | -0.01863768 | carboxymethylenebutenolidase homolog |
| ATP5L | -0.01862978 | ATP synthase, H+ transporting, mitochondrial Fo complex subunit G |
| TMEM151A | -0.01859025 | transmembrane protein 151A |
| KCTD5 | -0.01850593 | potassium channel tetramerization domain containing 5 |
| C1orf159 | -0.01849802 | chromosome 1 open reading frame 159 |
| FAM189A1 | -0.01848748 | family with sequence similarity 189 member A1 |
| KRTAP12-3 | -0.01842161 | keratin associated protein 12-3 |
| GINS4 | -0.01835837 | GINS complex subunit 4 |
| GDPD1 | -0.01828722 | glycerophosphodiester phosphodiesterase domain containing 1 |
| TMEM189 | -0.01824506 | transmembrane protein 189 |
| ELMO1 | -0.01818182 | engulfment and cell motility 1 |
| CNOT4 | -0.01814493 | CCR4-NOT transcription complex subunit 4 |
| SOD3 | -0.01803426 | superoxide dismutase 3, extracellular |
| PMPCA | -0.01802108 | peptidase, mitochondrial processing alpha subunit |
| HOXC13 | -0.01792358 | homeobox C13 |
| PIGY | -0.01775494 | phosphatidylinositol glycan anchor biosynthesis class Y |
| ARHGEF25 | -0.01769697 | Rho guanine nucleotide exchange factor 25 |
| GALNT15 | -0.01761528 | polypeptide N-acetylgalactosaminyltransferase 15 |
| SPINK2 | -0.01760211 | serine peptidase inhibitor, Kazal type 2 |
| CCT5 | -0.01759947 | chaperonin containing TCP1 subunit 5 |
| MINOS1 | -0.01758366 | mitochondrial inner membrane organizing system 1 |
| ANKRD39 | -0.01748353 | ankyrin repeat domain 39 |
| RAB33A | -0.01740975 | RAB33A, member RAS oncogene family |
| LY86 | -0.01739657 | lymphocyte antigen 86 |
| ZNF558 | -0.01723847 | zinc finger protein 558 |
| COX18 | -0.01722003 | COX18, cytochrome c oxidase assembly factor |
| FGF11 | -0.01710145 | fibroblast growth factor 11 |
| ALKBH6 | -0.01707773 | alkB homolog 6 |
| CYP2D6 | -0.01705929 | cytochrome P450 family 2 subfamily D member 6 |
| LDOC1L | -0.01699078 | leucine zipper down-regulated in cancer 1 like |
| PEX1 | -0.01696179 | peroxisomal biogenesis factor 1 |
| SCUBE1 | -0.01694335 | signal peptide, CUB domain and EGF like domain containing 1 |
| BHLHE22 | -0.01693017 | basic helix-loop-helix family member e22 |
| DSTN | -0.01691436 | destrin, actin depolymerizing factor |
| ODF3L1 | -0.01690909 | outer dense fiber of sperm tails 3 like 1 |
| CYTL1 | -0.01688011 | cytokine like 1 |
| TNFSF4 | -0.01685112 | tumor necrosis factor superfamily member 4 |
| PDE6A | -0.01677734 | phosphodiesterase 6A |
| SNORD42B | -0.01670356 | small nucleolar RNA, C/D box 42B |
| ATP1A2 | -0.01668775 | ATPase Na+/K+ transporting subunit alpha 2 |
| GSG1L | -0.01665086 | GSG1 like |
| ZNF324B | -0.01662451 | zinc finger protein 324B |
| PIGH | -0.01659025 | phosphatidylinositol glycan anchor biosynthesis class H |
| TSNAX | -0.01646904 | translin associated factor X |
| TMX4 | -0.0162635 | thioredoxin related transmembrane protein 4 |
| HEATR6 | -0.01625296 | HEAT repeat containing 6 |
| PAM16 | -0.01623452 | presequence translocase associated motor 16 homolog |
| B3GAT3 | -0.01613175 | beta-1,3-glucuronyltransferase 3 |
| EPHA3 | -0.0161054 | EPH receptor A3 |
| C3orf67 | -0.01606324 | chromosome 3 open reading frame 67 |
| DDX56 | -0.01601054 | DEAD-box helicase 56 |
| FREM1 | -0.01595257 | FRAS1 related extracellular matrix 1 |
| CDNF | -0.01592095 | cerebral dopamine neurotrophic factor |
| ABI3BP | -0.01566798 | ABI family member 3 binding protein |
| RNF2 | -0.01561265 | ring finger protein 2 |
| HAUS8 | -0.01548617 | HAUS augmin like complex subunit 8 |
| DOHH | -0.0154361 | deoxyhypusine hydroxylase/monooxygenase |
| COA7 | -0.01518577 | cytochrome c oxidase assembly factor 7 (putative) |
| RPS4X | -0.01505138 | ribosomal protein S4, X-linked |
| TOB2P1 | -0.01503294 | transducer of ERBB2, 2 pseudogene 1 |
| APC | -0.01501976 | APC, WNT signaling pathway regulator |
| ZNF620 | -0.0149249 | zinc finger protein 620 |
| C1QTNF6 | -0.0148195 | C1q and tumor necrosis factor related protein 6 |
| ITGB4 | -0.01456653 | integrin subunit beta 4 |
| MAP2K7 | -0.01450329 | mitogen-activated protein kinase kinase 7 |
| AARS2 | -0.01443742 | alanyl-tRNA synthetase 2, mitochondrial |
| FAM109A | -0.01442424 | family with sequence similarity 109 member A |
| NIT2 | -0.0143531 | nitrilase family member 2 |
| ATP1B2 | -0.01425033 | ATPase Na+/K+ transporting subunit beta 2 |
| FXYD1 | -0.01420026 | FXYD domain containing ion transport regulator 1 |
| IQGAP2 | -0.01417918 | IQ motif containing GTPase activating protein 2 |
| PTPA | -0.01412648 | protein phosphatase 2 phosphatase activator |
| RAB4B | -0.01399473 | RAB4B, member RAS oncogene family |
| RPUSD2 | -0.01398946 | RNA pseudouridylate synthase domain containing 2 |
| NALCN | -0.01395257 | sodium leak channel, non-selective |
| ZMYND11 | -0.01388142 | zinc finger MYND-type containing 11 |
| DNAH1 | -0.01386825 | dynein axonemal heavy chain 1 |
| APOB | -0.01386561 | apolipoprotein B |
| DSCR3 | -0.01367589 | DSCR3 arrestin fold containing |
| MRPL55 | -0.01359157 | mitochondrial ribosomal protein L55 |
| TPT1 | -0.01353623 | tumor protein, translationally-controlled 1 |
| SIRPA | -0.01350725 | signal regulatory protein alpha |
| CRTAP | -0.01315415 | cartilage associated protein |
| GMNC | -0.01307773 | geminin coiled-coil domain containing |
| SDAD1 | -0.012917 | SDA1 domain containing 1 |
| CCDC184 | -0.01264822 | coiled-coil domain containing 184 |
| BIRC5 | -0.01243215 | baculoviral IAP repeat containing 5 |
| ZNF121 | -0.01231357 | zinc finger protein 121 |
| LOC283713 | -0.01217655 | uncharacterized LOC283713 |
| CDC42SE2 | -0.01206588 | CDC42 small effector 2 |
| AOAH | -0.01201318 | acyloxyacyl hydrolase |
| THSD4 | -0.01197101 | thrombospondin type 1 domain containing 4 |
| NCF1 | -0.0119025 | neutrophil cytosolic factor 1 |
| NRROS | -0.01187088 | negative regulator of reactive oxygen species |
| ENTPD2 | -0.01185507 | ectonucleoside triphosphate diphosphohydrolase 2 |
| FABP6 | -0.01178129 | fatty acid binding protein 6 |
| ZNF3 | -0.01176021 | zinc finger protein 3 |
| G6PD | -0.01173386 | glucose-6-phosphate dehydrogenase |
| C1QTNF8 | -0.01165744 | C1q and tumor necrosis factor related protein 8 |
| MAR1 | -0.01157576 | membrane associated ring-CH-type finger 1 |
| ITPA | -0.01156785 | inosine triphosphatase |
| PCYT1A | -0.01154941 | phosphate cytidylyltransferase 1, choline, alpha |
| SLC29A2 | -0.01149671 | solute carrier family 29 member 2 |
| ULK1 | -0.01140448 | unc-51 like autophagy activating kinase 1 |
| JUP | -0.01129381 | junction plakoglobin |
| GRASP | -0.01127009 | general receptor for phosphoinositides 1 associated scaffold protein |
| THRA | -0.01100132 | thyroid hormone receptor, alpha |
| ZNF544 | -0.01098287 | zinc finger protein 544 |
| CEP85 | -0.01083794 | centrosomal protein 85 |
| CHMP2A | -0.01064295 | charged multivesicular body protein 2A |
| CECR3 | -0.01061924 | cat eye syndrome chromosome region, candidate 3 (non-protein coding) |
| SEP2 | -0.01060079 | septin 2 |
| RCBTB2 | -0.01056653 | RCC1 and BTB domain containing protein 2 |
| VPS33A | -0.01054282 | VPS33A, CORVET/HOPS core subunit |
| FBXW9 | -0.01052964 | F-box and WD repeat domain containing 9 |
| GUCA1C | -0.01038472 | guanylate cyclase activator 1C |
| RGS13 | -0.01037418 | regulator of G-protein signaling 13 |
| GUCY2D | -0.01031621 | guanylate cyclase 2D, retinal |
| ATP2B4 | -0.01028458 | ATPase plasma membrane Ca2+ transporting 4 |
| REV3L | -0.01026877 | REV3 like, DNA directed polymerase zeta catalytic subunit |
| UTP14C | -0.0102556 | UTP14, small subunit processome component homolog C (S. cerevisiae) |
| DOC2A | -0.0102029 | double C2 domain alpha |
| DAB2 | -0.0101581 | DAB2, clathrin adaptor protein |
| AP3M1 | -0.01011858 | adaptor related protein complex 3 mu 1 subunit |
| NDRG1 | -0.00993676 | N-myc downstream regulated 1 |
| BRCA2 | -0.00987879 | BRCA2, DNA repair associated |
| ZNF473 | -0.00987879 | zinc finger protein 473 |
| CXCR3 | -0.00982345 | C-X-C motif chemokine receptor 3 |
| ACAP2 | -0.00972332 | ArfGAP with coiled-coil, ankyrin repeat and PH domains 2 |
| PRKAG1 | -0.00953623 | protein kinase AMP-activated non-catalytic subunit gamma 1 |
| SFI1 | -0.00939921 | SFI1 centrin binding protein |
| RPL36A | -0.00938076 | ribosomal protein L36a |
| RBBP9 | -0.00935441 | RB binding protein 9, serine hydrolase |
| TNFSF14 | -0.00929381 | tumor necrosis factor superfamily member 14 |
| CHRM2 | -0.00915415 | cholinergic receptor muscarinic 2 |
| ST6GAL1 | -0.00901449 | ST6 beta-galactoside alpha-2,6-sialyltransferase 1 |
| FZD1 | -0.00896443 | frizzled class receptor 1 |
| LRRC37A2 | -0.00884058 | leucine rich repeat containing 37 member A2 |
| MAVS | -0.00881159 | mitochondrial antiviral signaling protein |
| EVI2B | -0.00880896 | ecotropic viral integration site 2B |
| NDUFS4 | -0.00871673 | NADH:ubiquinone oxidoreductase subunit S4 |
| MROH1 | -0.00870092 | maestro heat like repeat family member 1 |
| TMEM186 | -0.00845323 | transmembrane protein 186 |
| NCLN | -0.00844269 | nicalin |
| LST1 | -0.00840843 | leukocyte specific transcript 1 |
| HOMER3 | -0.00838999 | homer scaffolding protein 3 |
| COL21A1 | -0.00837945 | collagen type XXI alpha 1 chain |
| CCDC189 | -0.00834519 | coiled-coil domain containing 189 |
| TIMP3 | -0.00829776 | TIMP metallopeptidase inhibitor 3 |
| GSTM5 | -0.00823979 | glutathione S-transferase mu 5 |
| HNRNPH2 | -0.00822398 | heterogeneous nuclear ribonucleoprotein H2 (H') |
| DCHS1 | -0.00821344 | dachsous cadherin-related 1 |
| MEF2C | -0.00819236 | myocyte enhancer factor 2C |
| MEIS1 | -0.00818709 | Meis homeobox 1 |
| FDX1L | -0.00813702 | ferredoxin 1 like |
| ITM2C | -0.00809223 | integral membrane protein 2C |
| CPOX | -0.00806851 | coproporphyrinogen oxidase |
| PRF1 | -0.00800791 | perforin 1 |
| TNFSF12-TNFSF13 | -0.00791041 | TNFSF12-TNFSF13 readthrough |
| NID1 | -0.00789723 | nidogen 1 |
| AMOT | -0.00783663 | angiomotin |
| MYH13 | -0.00777602 | myosin heavy chain 13 |
| GALE | -0.00776021 | UDP-galactose-4-epimerase |
| FUT11 | -0.00773913 | fucosyltransferase 11 |
| MFN1 | -0.00772069 | mitofusin 1 |
| CBLN1 | -0.00770487 | cerebellin 1 precursor |
| NOTUM | -0.00769433 | NOTUM, palmitoleoyl-protein carboxylesterase |
| EIF5A2 | -0.00768379 | eukaryotic translation initiation factor 5A2 |
| TAF15 | -0.00767589 | TATA-box binding protein associated factor 15 |
| LRRC8E | -0.00762846 | leucine rich repeat containing 8 family member E |
| LIMD1 | -0.00762319 | LIM domains containing 1 |
| TTTY6 | -0.00760474 | testis-specific transcript, Y-linked 6 (non-protein coding) |
| ITGA4 | -0.00758893 | integrin subunit alpha 4 |
| OAZ3 | -0.00756785 | ornithine decarboxylase antizyme 3 |
| JAZF1-AS1 | -0.00742819 | JAZF1 antisense RNA 1 |
| IFNAR1 | -0.00735441 | interferon alpha and beta receptor subunit 1 |
| INPP5F | -0.00732543 | inositol polyphosphate-5-phosphatase F |
| GLIPR2 | -0.00727273 | GLI pathogenesis related 2 |
| SLC22A15 | -0.00717523 | solute carrier family 22 member 15 |
| ATAT1 | -0.00698287 | alpha tubulin acetyltransferase 1 |
| TEX101 | -0.00692227 | testis expressed 101 |
| ANAPC13 | -0.00681423 | anaphase promoting complex subunit 13 |
| NMT1 | -0.00679578 | N-myristoyltransferase 1 |
| LAP3 | -0.00673781 | leucine aminopeptidase 3 |
| LGALS1 | -0.00670356 | galectin 1 |
| TMTC2 | -0.00657971 | transmembrane and tetratricopeptide repeat containing 2 |
| INTS8 | -0.00656653 | integrator complex subunit 8 |
| DZIP1 | -0.00647958 | DAZ interacting zinc finger protein 1 |
| LIN54 | -0.00643478 | lin-54 DREAM MuvB core complex component |
| ECHDC1 | -0.00605534 | ethylmalonyl-CoA decarboxylase 1 |
| JAK1 | -0.0060448 | Janus kinase 1 |
| TMEM57 | -0.00595784 | transmembrane protein 57 |
| CD320 | -0.00579447 | CD320 molecule |
| GPR65 | -0.00579183 | G protein-coupled receptor 65 |
| CDC27 | -0.00568643 | cell division cycle 27 |
| HGS | -0.00562846 | hepatocyte growth factor-regulated tyrosine kinase substrate |
| OR5M3 | -0.00552306 | olfactory receptor family 5 subfamily M member 3 |
| TNS2 | -0.00534914 | tensin 2 |
| DNAJB9 | -0.00532279 | DnaJ heat shock protein family (Hsp40) member B9 |
| ZNF613 | -0.00529117 | zinc finger protein 613 |
| GZMM | -0.00524111 | granzyme M |
| TMEM88 | -0.00524111 | transmembrane protein 88 |
| LCN6 | -0.00515415 | lipocalin 6 |
| TOMM7 | -0.00511199 | translocase of outer mitochondrial membrane 7 |
| FOXF1 | -0.00498551 | forkhead box F1 |
| ZBTB49 | -0.004917 | zinc finger and BTB domain containing 49 |
| DAAM2 | -0.00488274 | dishevelled associated activator of morphogenesis 2 |
| CAMK4 | -0.00487747 | calcium/calmodulin dependent protein kinase IV |
| G3BP2 | -0.00482477 | G3BP stress granule assembly factor 2 |
| DPH5 | -0.00475626 | diphthamide biosynthesis 5 |
| PPP1R3B | -0.00473781 | protein phosphatase 1 regulatory subunit 3B |
| TMEM173 | -0.00472464 | transmembrane protein 173 |
| VSTM4 | -0.00465086 | V-set and transmembrane domain containing 4 |
| MRPL2 | -0.00463241 | mitochondrial ribosomal protein L2 |
| SNHG4 | -0.00452437 | small nucleolar RNA host gene 4 |
| FIBP | -0.00441107 | FGF1 intracellular binding protein |
| PRKACB | -0.00437154 | protein kinase cAMP-activated catalytic subunit beta |
| KLRG1 | -0.00435046 | killer cell lectin like receptor G1 |
| LINC01081 | -0.00426614 | long intergenic non-protein coding RNA 1081 |
| RABGGTA | -0.00421871 | Rab geranylgeranyltransferase alpha subunit |
| SYNPO2 | -0.00413702 | synaptopodin 2 |
| BMT2 | -0.00413175 | base methyltransferase of 25S rRNA 2 homolog |
| BBX | -0.00408169 | BBX, HMG-box containing |
| LOC105373185 | -0.00403953 | uncharacterized LOC105373185 |
| NUDT10 | -0.00399209 | nudix hydrolase 10 |
| NFIX | -0.00390777 | nuclear factor I X |
| MOSPD3 | -0.0039025 | motile sperm domain containing 3 |
| DPH2 | -0.00381291 | DPH2 homolog |
| KRTAP10-6 | -0.00375494 | keratin associated protein 10-6 |
| JAK3 | -0.00356522 | Janus kinase 3 |
| SOX6 | -0.00350461 | SRY-box 6 |
| CHEK2 | -0.00345982 | checkpoint kinase 2 |
| GTF2IRD1 | -0.00336759 | GTF2I repeat domain containing 1 |
| ATP6V0E1 | -0.00333333 | ATPase H+ transporting V0 subunit e1 |
| ATP11B | -0.00329117 | ATPase phospholipid transporting 11B (putative) |
| SP5 | -0.00322266 | Sp5 transcription factor |
| LONRF2 | -0.00322003 | LON peptidase N-terminal domain and ring finger 2 |
| ANGPTL6 | -0.0031805 | angiopoietin like 6 |
| SVIL | -0.00316996 | supervillin |
| ADPRHL2 | -0.00308037 | ADP-ribosylhydrolase like 2 |
| TOP3A | -0.00306456 | topoisomerase (DNA) III alpha |
| KIAA0922 | -0.00302503 | KIAA0922 |
| NCBP1 | -0.00301976 | nuclear cap binding protein subunit 1 |
| PISD | -0.00292227 | phosphatidylserine decarboxylase |
| FANCA | -0.00291173 | Fanconi anemia complementation group A |
| ACTR5 | -0.0028274 | ARP5 actin-related protein 5 homolog |
| NLE1 | -0.00282477 | notchless homolog 1 |
| EVI2A | -0.00280896 | ecotropic viral integration site 2A |
| TSC22D2 | -0.00278524 | TSC22 domain family member 2 |
| USH1C | -0.00265086 | USH1 protein network component harmonin |
| C4B | -0.00253491 | complement component 4B (Chido blood group) |
| BYSL | -0.0024664 | bystin like |
| RETNLB | -0.00245586 | resistin like beta |
| MIER2 | -0.00240843 | MIER family member 2 |
| LOC100996263 | -0.0023531 | uncharacterized LOC100996263 |
| MAP3K3 | -0.00233202 | mitogen-activated protein kinase kinase kinase 3 |
| TDG | -0.00227404 | thymine DNA glycosylase |
| KHDRBS3 | -0.00215547 | KH RNA binding domain containing, signal transduction associated 3 |
| CD48 | -0.00213175 | CD48 molecule |
| TBX10 | -0.0020975 | T-box 10 |
| GHRL | -0.00207905 | ghrelin/obestatin prepropeptide |
| WDR34 | -0.00206851 | WD repeat domain 34 |
| A4GNT | -0.00204743 | alpha-1,4-N-acetylglucosaminyltransferase |
| ZNF768 | -0.00193149 | zinc finger protein 768 |
| ZNF653 | -0.00188406 | zinc finger protein 653 |
| CBLN3 | -0.00186561 | cerebellin 3 precursor |
| FYCO1 | -0.00184717 | FYVE and coiled-coil domain containing 1 |
| MAMDC2 | -0.0017892 | MAM domain containing 2 |
| GPR78 | -0.00162846 | G protein-coupled receptor 78 |
| GFI1 | -0.00158366 | growth factor independent 1 transcriptional repressor |
| PCSK2 | -0.00137813 | proprotein convertase subtilisin/kexin type 2 |
| FANCG | -0.00135705 | Fanconi anemia complementation group G |
| PPP2R1A | -0.00130698 | protein phosphatase 2 scaffold subunit Aalpha |
| NFATC2IP | -0.00129908 | nuclear factor of activated T-cells 2 interacting protein |
| PROP1 | -0.00124638 | PROP paired-like homeobox 1 |
| HBZ | -0.0008643 | hemoglobin subunit zeta |
| SPON2 | -0.00084585 | spondin 2 |
| NT5E | -0.0008195 | 5'-nucleotidase ecto |
| IQCC | -0.00079315 | IQ motif containing C |
| TIMM21 | -0.00077734 | translocase of inner mitochondrial membrane 21 |
| RAB3A | -0.0007747 | RAB3A, member RAS oncogene family |
| ST6GALNAC3 | -0.00071673 | ST6 N-acetylgalactosaminide alpha-2,6-sialyltransferase 3 |
| ISL2 | -0.00070356 | ISL LIM homeobox 2 |
| CCDC65 | -0.00065349 | coiled-coil domain containing 65 |
| TRMT12 | -0.00062451 | tRNA methyltransferase 12 homolog |
| FAM110D | -0.00060079 | family with sequence similarity 110 member D |
| CCDC183 | -0.00059816 | coiled-coil domain containing 183 |
| KREMEN2 | -0.00059289 | kringle containing transmembrane protein 2 |
| C14orf1 | -0.00049275 | chromosome 14 open reading frame 1 |
| ADTRP | -0.00047958 | androgen dependent TFPI regulating protein |
| LLGL2 | -0.00042688 | LLGL2, scribble cell polarity complex component |
| LOC729040 | -0.00042424 | uncharacterized LOC729040 |
| MMP7 | -0.00035573 | matrix metallopeptidase 7 |
| TRDMT1 | -0.00024506 | tRNA aspartic acid methyltransferase 1 |
| KRIT1 | -0.00012648 | KRIT1, ankyrin repeat containing |
| HIST3H2BB | -8.17E-05 | histone cluster 3, H2bb |
| SMPD4 | -7.12E-05 | sphingomyelin phosphodiesterase 4 |
| FABP1 | -2.64E-05 | fatty acid binding protein 1 |

**Table S31.** The list of down-regulated genes in PBC-RNIT

| Gene.symbol | logFC | Gene.title |
| --- | --- | --- |
| CLPSL2 | 3.16E-05 | colipase like 2 |
| IKZF1 | 3.69E-05 | IKAROS family zinc finger 1 |
| RECQL5 | 0.00012121 | RecQ like helicase 5 |
| SGCE | 0.00022925 | sarcoglycan epsilon |
| MED24 | 0.00025823 | mediator complex subunit 24 |
| HIC1 | 0.00029776 | hypermethylated in cancer 1 |
| VSTM2A-OT1 | 0.00031621 | VSTM2A overlapping transcript 1 |
| HSPG2 | 0.00041897 | heparan sulfate proteoglycan 2 |
| TMEM39B | 0.00045323 | transmembrane protein 39B |
| EIF3CL | 0.00061397 | eukaryotic translation initiation factor 3 subunit C-like |
| AFAP1L2 | 0.0006166 | actin filament associated protein 1 like 2 |
| LINC01534 | 0.00062187 | long intergenic non-protein coding RNA 1534 |
| FGFR4 | 0.00063768 | fibroblast growth factor receptor 4 |
| LARS | 0.0007141 | leucyl-tRNA synthetase |
| ABCA12 | 0.00083794 | ATP binding cassette subfamily A member 12 |
| GPR55 | 0.00091173 | G protein-coupled receptor 55 |
| CDV3 | 0.00108827 | CDV3 homolog |
| EEF2K | 0.00124638 | eukaryotic elongation factor 2 kinase |
| KLHL5 | 0.0012859 | kelch like family member 5 |
| SALL1 | 0.00132016 | spalt like transcription factor 1 |
| XAB2 | 0.00134387 | XPA binding protein 2 |
| SORBS1 | 0.00135178 | sorbin and SH3 domain containing 1 |
| TAS2R20 | 0.00140975 | taste 2 receptor member 20 |
| GNRH1 | 0.00145455 | gonadotropin releasing hormone 1 |
| ZNF444 | 0.00149934 | zinc finger protein 444 |
| SP4 | 0.00162319 | Sp4 transcription factor |
| SELM | 0.00163373 | selenoprotein M |
| MYLK2 | 0.00165217 | myosin light chain kinase 2 |
| PGK2 | 0.00173913 | phosphoglycerate kinase 2 |
| FAM111B | 0.00177602 | family with sequence similarity 111 member B |
| TIGD5 | 0.00181291 | tigger transposable element derived 5 |
| COPZ2 | 0.00186034 | coatomer protein complex subunit zeta 2 |
| ALG14 | 0.00198419 | ALG14, UDP-N-acetylglucosaminyltransferase subunit |
| MACROD2 | 0.00205534 | MACRO domain containing 2 |
| PLPP7 | 0.00206061 | phospholipid phosphatase 7 (inactive) |
| TTLL12 | 0.00211594 | tubulin tyrosine ligase like 12 |
| MLST8 | 0.00218972 | MTOR associated protein, LST8 homolog |
| TNFRSF6B | 0.00225823 | TNF receptor superfamily member 6b |
| JAML | 0.00226614 | junction adhesion molecule like |
| ZNHIT1 | 0.00233465 | zinc finger HIT-type containing 1 |
| HIST1H4E | 0.00237945 | histone cluster 1, H4e |
| TAS2R39 | 0.00247167 | taste 2 receptor member 39 |
| MYO1F | 0.00247431 | myosin IF |
| BACE2 | 0.00250066 | beta-site APP-cleaving enzyme 2 |
| MN1 | 0.0026166 | MN1 proto-oncogene, transcriptional regulator |
| TSTD2 | 0.00262187 | thiosulfate sulfurtransferase like domain containing 2 |
| EPHA5 | 0.00262451 | EPH receptor A5 |
| CD276 | 0.00264822 | CD276 molecule |
| PRDM4 | 0.00268775 | PR/SET domain 4 |
| ATXN7L2 | 0.00275099 | ataxin 7 like 2 |
| KCNQ4 | 0.00288011 | potassium voltage-gated channel subfamily Q member 4 |
| KLHL42 | 0.00299868 | kelch like family member 42 |
| MEF2D | 0.00301713 | myocyte enhancer factor 2D |
| EPHX1 | 0.00305402 | epoxide hydrolase 1 |
| PCBP4 | 0.00316996 | poly(rC) binding protein 4 |
| ACO2 | 0.0031726 | aconitase 2 |
| MOB4 | 0.00324901 | MOB family member 4, phocein |
| DHX37 | 0.00332016 | DEAH-box helicase 37 |
| AMIGO2 | 0.0033386 | adhesion molecule with Ig like domain 2 |
| TEAD4 | 0.00335441 | TEA domain transcription factor 4 |
| HSPA1L | 0.00336759 | heat shock protein family A (Hsp70) member 1 like |
| RBFOX3 | 0.00348617 | RNA binding protein, fox-1 homolog 3 |
| NXPH3 | 0.00348617 | neurexophilin 3 |
| RPL35A | 0.00360474 | ribosomal protein L35a |
| CORIN | 0.00365481 | corin, serine peptidase |
| ITGA11 | 0.00393149 | integrin subunit alpha 11 |
| ZNF781 | 0.00393676 | zinc finger protein 781 |
| SNX11 | 0.00396047 | sorting nexin 11 |
| NOC2L | 0.00398946 | NOC2 like nucleolar associated transcriptional repressor |
| ALKBH4 | 0.004 | alkB homolog 4, lysine demethylase |
| CXorf65 | 0.00400791 | chromosome X open reading frame 65 |
| TFIP11 | 0.00402108 | tuftelin interacting protein 11 |
| DSCAML1 | 0.00411067 | DS cell adhesion molecule like 1 |
| KRTAP19-2 | 0.00418182 | keratin associated protein 19-2 |
| TMEM150A | 0.0042029 | transmembrane protein 150A |
| NSDHL | 0.00422134 | NAD(P) dependent steroid dehydrogenase-like |
| RAB37 | 0.00422925 | RAB37, member RAS oncogene family |
| MYL6 | 0.00431357 | myosin light chain 6 |
| DR1 | 0.00446113 | down-regulator of transcription 1 |
| PLEKHO1 | 0.00448221 | pleckstrin homology domain containing O1 |
| DNA2 | 0.00449802 | DNA replication helicase/nuclease 2 |
| UPB1 | 0.00456653 | beta-ureidopropionase 1 |
| MAP2K3 | 0.00462451 | mitogen-activated protein kinase kinase 3 |
| MRVI1 | 0.00473781 | murine retrovirus integration site 1 homolog |
| CDH3 | 0.00480632 | cadherin 3 |
| TRAF3 | 0.00489592 | TNF receptor associated factor 3 |
| AHCYL2 | 0.00493544 | adenosylhomocysteinase like 2 |
| ME1 | 0.00494862 | malic enzyme 1 |
| SPTBN4 | 0.00497497 | spectrin beta, non-erythrocytic 4 |
| INSR | 0.00498287 | insulin receptor |
| B3GALT2 | 0.00500659 | beta-1,3-galactosyltransferase 2 |
| AP1AR | 0.00500922 | adaptor related protein complex 1 associated regulatory protein |
| STX8 | 0.00516206 | syntaxin 8 |
| NOP9 | 0.00521212 | NOP9 nucleolar protein |
| CALCOCO2 | 0.00539921 | calcium binding and coiled-coil domain 2 |
| CORO1C | 0.00540184 | coronin 1C |
| OTUB1 | 0.0055415 | OTU deubiquitinase, ubiquitin aldehyde binding 1 |
| CKMT2 | 0.00572596 | creatine kinase, mitochondrial 2 |
| OR2J2 | 0.00575494 | olfactory receptor family 2 subfamily J member 2 |
| SNORD43 | 0.00575494 | small nucleolar RNA, C/D box 43 |
| MTBP | 0.00577866 | MDM2 binding protein |
| CD1B | 0.0057971 | CD1b molecule |
| RAB9B | 0.00581555 | RAB9B, member RAS oncogene family |
| DUSP18 | 0.00592622 | dual specificity phosphatase 18 |
| ERO1B | 0.00593412 | endoplasmic reticulum oxidoreductase 1 beta |
| TDRD10 | 0.00603953 | tudor domain containing 10 |
| NPLOC4 | 0.00608696 | NPL4 homolog, ubiquitin recognition factor |
| POLR1C | 0.00630567 | RNA polymerase I subunit C |
| HEPH | 0.00640316 | hephaestin |
| FBLN5 | 0.0064137 | fibulin 5 |
| MAZ | 0.00644532 | MYC associated zinc finger protein |
| YKT6 | 0.00647958 | YKT6 v-SNARE homolog (S. cerevisiae) |
| HS6ST2-AS1 | 0.00649802 | HS6ST2 antisense RNA 1 |
| MAD2L2 | 0.00652701 | MAD2 mitotic arrest deficient-like 2 (yeast) |
| NBL1 | 0.00660606 | neuroblastoma 1, DAN family BMP antagonist |
| EFNA4 | 0.00667984 | ephrin A4 |
| TMEM204 | 0.00670356 | transmembrane protein 204 |
| NPFFR2 | 0.00673254 | neuropeptide FF receptor 2 |
| PTGDR | 0.00675889 | prostaglandin D2 receptor |
| DIAPH1 | 0.00676416 | diaphanous related formin 1 |
| BARHL2 | 0.00676943 | BarH like homeobox 2 |
| GPR15 | 0.00685375 | G protein-coupled receptor 15 |
| PLOD3 | 0.00686957 | procollagen-lysine,2-oxoglutarate 5-dioxygenase 3 |
| C16orf89 | 0.00692227 | chromosome 16 open reading frame 89 |
| FAM101A | 0.0069776 | family with sequence similarity 101 member A |
| NCAPH | 0.00700922 | non-SMC condensin I complex subunit H |
| PPP2R5A | 0.00701976 | protein phosphatase 2 regulatory subunit B'alpha |
| ARHGAP8 | 0.00728063 | Rho GTPase activating protein 8 |
| TECR | 0.00734651 | trans-2,3-enoyl-CoA reductase |
| HIST2H3C | 0.00734914 | histone cluster 2, H3c |
| FAM102B | 0.00736495 | family with sequence similarity 102 member B |
| RSU1 | 0.00737813 | Ras suppressor protein 1 |
| CCDC121 | 0.00743874 | coiled-coil domain containing 121 |
| C16orf70 | 0.00745718 | chromosome 16 open reading frame 70 |
| FAM19A5 | 0.00760474 | family with sequence similarity 19 member A5, C-C motif chemokine like |
| ORMDL2 | 0.00762055 | ORMDL sphingolipid biosynthesis regulator 2 |
| SMIM11A | 0.00763109 | small integral membrane protein 11A |
| ZFPL1 | 0.00763373 | zinc finger protein like 1 |
| SKA3 | 0.00764954 | spindle and kinetochore associated complex subunit 3 |
| MEN1 | 0.00770224 | menin 1 |
| MRFAP1 | 0.00772859 | Morf4 family associated protein 1 |
| PSMD7 | 0.0077365 | proteasome 26S subunit, non-ATPase 7 |
| OR2T2 | 0.0077444 | olfactory receptor family 2 subfamily T member 2 |
| COL11A2 | 0.00774704 | collagen type XI alpha 2 chain |
| CDHR3 | 0.00774704 | cadherin related family member 3 |
| DKKL1 | 0.00777866 | dickkopf like acrosomal protein 1 |
| TNXA | 0.00789987 | tenascin XA (pseudogene) |
| PQLC3 | 0.00792095 | PQ loop repeat containing 3 |
| INTS5 | 0.00796311 | integrator complex subunit 5 |
| DTNBP1 | 0.00799473 | dystrobrevin binding protein 1 |
| DEPDC5 | 0.00804743 | DEP domain containing 5 |
| FYB | 0.00805534 | FYN binding protein |
| MPP5 | 0.00806324 | membrane palmitoylated protein 5 |
| ARHGAP31 | 0.00812648 | Rho GTPase activating protein 31 |
| LOXL2 | 0.00816601 | lysyl oxidase like 2 |
| GNL2 | 0.00825823 | G protein nucleolar 2 |
| PIK3R6 | 0.00836364 | phosphoinositide-3-kinase regulatory subunit 6 |
| CA3 | 0.00838208 | carbonic anhydrase 3 |
| KPNA6 | 0.00855599 | karyopherin subunit alpha 6 |
| HAND1 | 0.00858762 | heart and neural crest derivatives expressed 1 |
| KANSL1L | 0.00860079 | KAT8 regulatory NSL complex subunit 1 like |
| WDR4 | 0.00861397 | WD repeat domain 4 |
| PTGER3 | 0.0086614 | prostaglandin E receptor 3 |
| ARHGAP24 | 0.0088274 | Rho GTPase activating protein 24 |
| NTNG1 | 0.00898551 | netrin G1 |
| NTNG1 | 0.00898551 | netrin G1 |
| RYR3 | 0.00899078 | ryanodine receptor 3 |
| PSMC1 | 0.00929117 | proteasome 26S subunit, ATPase 1 |
| PSMC1 | 0.00929117 | proteasome 26S subunit, ATPase 1 |
| CCDC80 | 0.00939657 | coiled-coil domain containing 80 |
| RSPH9 | 0.00945191 | radial spoke head 9 homolog |
| CD3G | 0.00949407 | CD3g molecule |
| FAT4 | 0.00952569 | FAT atypical cadherin 4 |
| ZFAND6 | 0.00955468 | zinc finger AN1-type containing 6 |
| ARMCX2 | 0.00957049 | armadillo repeat containing, X-linked 2 |
| CABP4 | 0.00959947 | calcium binding protein 4 |
| CACNB1 | 0.00962846 | calcium voltage-gated channel auxiliary subunit beta 1 |
| RNF103 | 0.00968116 | ring finger protein 103 |
| GFPT2 | 0.00972332 | glutamine-fructose-6-phosphate transaminase 2 |
| PARK2 | 0.00993939 | parkin RBR E3 ubiquitin protein ligase |
| SCN4B | 0.0099552 | sodium voltage-gated channel beta subunit 4 |
| TEX12 | 0.00995784 | testis expressed 12 |
| NCALD | 0.00997628 | neurocalcin delta |
| STXBP6 | 0.00998155 | syntaxin binding protein 6 |
| EHF | 0.01008696 | ETS homologous factor |
| TMED7 | 0.01011331 | transmembrane p24 trafficking protein 7 |
| EMSY | 0.0101581 | EMSY, BRCA2 interacting transcriptional repressor |
| PPP1R12B | 0.01016337 | protein phosphatase 1 regulatory subunit 12B |
| IL22RA1 | 0.01020026 | interleukin 22 receptor subunit alpha 1 |
| ARSB | 0.01023188 | arylsulfatase B |
| LRRTM4 | 0.01032411 | leucine rich repeat transmembrane neuronal 4 |
| KLB | 0.0104664 | klotho beta |
| ZXDA | 0.01052701 | zinc finger, X-linked, duplicated A |
| PRDM6 | 0.01056126 | PR/SET domain 6 |
| KCNE4 | 0.0105639 | potassium voltage-gated channel subfamily E regulatory subunit 4 |
| GPR146 | 0.01078261 | G protein-coupled receptor 146 |
| WTAP | 0.01083267 | Wilms tumor 1 associated protein |
| WDTC1 | 0.0108643 | WD and tetratricopeptide repeats 1 |
| SYNGR2 | 0.01093017 | synaptogyrin 2 |
| FAM161B | 0.01095652 | family with sequence similarity 161 member B |
| LOC101928760 | 0.0110224 | uncharacterized LOC101928760 |
| CEP112 | 0.0110303 | centrosomal protein 112 |
| IGF2BP1 | 0.01113043 | insulin like growth factor 2 mRNA binding protein 1 |
| FOXK2 | 0.01119104 | forkhead box K2 |
| KIAA0226L | 0.01123057 | KIAA0226 like |
| RADIL | 0.01124638 | Rap associating with DIL domain |
| GEMIN8P4 | 0.01139657 | gem nuclear organelle associated protein 8 pseudogene 4 |
| DNAJC5 | 0.0115863 | DnaJ heat shock protein family (Hsp40) member C5 |
| FBXO44 | 0.01159947 | F-box protein 44 |
| OR2Z1 | 0.01159947 | olfactory receptor family 2 subfamily Z member 1 |
| DNMBP | 0.01162319 | dynamin binding protein |
| TMEM81 | 0.01164954 | transmembrane protein 81 |
| LRRC38 | 0.01167589 | leucine rich repeat containing 38 |
| ODF2 | 0.01171805 | outer dense fiber of sperm tails 2 |
| FAM65B | 0.0117971 | family with sequence similarity 65 member B |
| FGFR1 | 0.01180501 | fibroblast growth factor receptor 1 |
| NARFL | 0.01181028 | nuclear prelamin A recognition factor like |
| TNF | 0.01186298 | tumor necrosis factor |
| TCEB3 | 0.01199209 | transcription elongation factor B subunit 3 |
| FADS2 | 0.01199209 | fatty acid desaturase 2 |
| AOC3 | 0.01202899 | amine oxidase, copper containing 3 |
| WDR90 | 0.01203426 | WD repeat domain 90 |
| FUT6 | 0.01204216 | fucosyltransferase 6 |
| MGAT5 | 0.01207378 | mannosyl (alpha-1,6-)-glycoprotein beta-1,6-N-acetyl-glucosaminyltransferase |
| INO80B | 0.01211067 | INO80 complex subunit B |
| ARID5B | 0.01212385 | AT-rich interaction domain 5B |
| DHTKD1 | 0.01219236 | dehydrogenase E1 and transketolase domain containing 1 |
| HERPUD1 | 0.01224506 | homocysteine inducible ER protein with ubiquitin like domain 1 |
| POU2F2 | 0.01229776 | POU class 2 homeobox 2 |
| RRAGA | 0.01238999 | Ras related GTP binding A |
| GALNT9 | 0.0124585 | polypeptide N-acetylgalactosaminyltransferase 9 |
| TPM2 | 0.01255599 | tropomyosin 2 (beta) |
| BRSK1 | 0.01259025 | BR serine/threonine kinase 1 |
| INPP5A | 0.01262187 | inositol polyphosphate-5-phosphatase A |
| GAS2L3 | 0.01271146 | growth arrest specific 2 like 3 |
| NXPE3 | 0.01274835 | neurexophilin and PC-esterase domain family member 3 |
| LDB3 | 0.01281423 | LIM domain binding 3 |
| AKAP14 | 0.01281686 | A-kinase anchoring protein 14 |
| KIR3DL2 | 0.01284848 | killer cell immunoglobulin like receptor, three Ig domains and long cytoplasmic tail 2 |
| PDLIM2 | 0.01296443 | PDZ and LIM domain 2 |
| FAM177A1 | 0.01296706 | family with sequence similarity 177 member A1 |
| GPR45 | 0.01298287 | G protein-coupled receptor 45 |
| FASLG | 0.01308037 | Fas ligand |
| C11orf84 | 0.01309354 | chromosome 11 open reading frame 84 |
| PHKB | 0.01310672 | phosphorylase kinase regulatory subunit beta |
| RB1 | 0.01315679 | RB transcriptional corepressor 1 |
| HEMGN | 0.01322793 | hemogen |
| FMOD | 0.01325692 | fibromodulin |
| RPL17 | 0.01333333 | ribosomal protein L17 |
| PQLC2 | 0.01334124 | PQ loop repeat containing 2 |
| CRHBP | 0.01334124 | corticotropin releasing hormone binding protein |
| ARMCX3 | 0.01334914 | armadillo repeat containing, X-linked 3 |
| ZAN | 0.01344664 | zonadhesin (gene/pseudogene) |
| WNT2 | 0.01345455 | Wnt family member 2 |
| KY | 0.01347563 | kyphoscoliosis peptidase |
| TOM1L2 | 0.01350198 | target of myb1 like 2 membrane trafficking protein |
| MLLT11 | 0.01353623 | myeloid/lymphoid or mixed-lineage leukemia; translocated to, 11 |
| DDB1 | 0.01359684 | damage specific DNA binding protein 1 |
| DIP2A | 0.01363636 | disco interacting protein 2 homolog A |
| MYO10 | 0.01363636 | myosin X |
| COLGALT2 | 0.01365217 | collagen beta(1-O)galactosyltransferase 2 |
| CORO2B | 0.01377866 | coronin 2B |
| LINC01015 | 0.01377866 | long intergenic non-protein coding RNA 1015 |
| KCNK2 | 0.01379447 | potassium two pore domain channel subfamily K member 2 |
| RGL4 | 0.01381555 | ral guanine nucleotide dissociation stimulator like 4 |
| LINC00844 | 0.01384717 | long intergenic non-protein coding RNA 844 |
| ZFYVE16 | 0.01386034 | zinc finger FYVE-type containing 16 |
| NHLRC1 | 0.01388142 | NHL repeat containing E3 ubiquitin protein ligase 1 |
| GPR21 | 0.01391304 | G protein-coupled receptor 21 |
| TUBA4B | 0.0139473 | tubulin alpha 4b |
| LIFR | 0.01398419 | leukemia inhibitory factor receptor alpha |
| MIIP | 0.01406588 | migration and invasion inhibitory protein |
| SF3B6 | 0.01417128 | splicing factor 3b subunit 6 |
| GTF2H2 | 0.01417391 | general transcription factor IIH subunit 2 |
| LILRA4 | 0.01422134 | leukocyte immunoglobulin like receptor A4 |
| ARHGEF39 | 0.01427931 | Rho guanine nucleotide exchange factor 39 |
| CLU | 0.0143004 | clusterin |
| STEAP2-AS1 | 0.01434256 | STEAP2 antisense RNA 1 |
| SSTR3 | 0.01444269 | somatostatin receptor 3 |
| ETV2 | 0.01455336 | ETS variant 2 |
| LOC102723757 | 0.01455863 | uncharacterized LOC102723757 |
| LEPR | 0.01463241 | leptin receptor |
| B3GNT8 | 0.01478788 | UDP-GlcNAc:betaGal beta-1,3-N-acetylglucosaminyltransferase 8 |
| ATP5A1 | 0.01485112 | ATP synthase, H+ transporting, mitochondrial F1 complex, alpha subunit 1, cardiac muscle |
| HNRNPU | 0.01493544 | heterogeneous nuclear ribonucleoprotein U |
| HNRNPA0 | 0.01502503 | heterogeneous nuclear ribonucleoprotein A0 |
| MRPL57 | 0.01502767 | mitochondrial ribosomal protein L57 |
| CELA1 | 0.01505138 | chymotrypsin like elastase family member 1 |
| B3GNT6 | 0.01508564 | UDP-GlcNAc:betaGal beta-1,3-N-acetylglucosaminyltransferase 6 |
| RANBP3 | 0.01510935 | RAN binding protein 3 |
| KIR2DS4 | 0.0152253 | killer cell immunoglobulin like receptor, two Ig domains and short cytoplasmic tail 4 |
| CPED1 | 0.01524901 | cadherin like and PC-esterase domain containing 1 |
| RPTOR | 0.01527273 | regulatory associated protein of MTOR complex 1 |
| FKBPL | 0.01537286 | FK506 binding protein like |
| OR2W5 | 0.01553623 | olfactory receptor family 2 subfamily W member 5 (gene/pseudogene) |
| DLL1 | 0.01555204 | delta like canonical Notch ligand 1 |
| PTCH2 | 0.01558893 | patched 2 |
| FBXL21 | 0.01560738 | F-box and leucine rich repeat protein 21 (gene/pseudogene) |
| TMEM9 | 0.01571014 | transmembrane protein 9 |
| RUNX1T1 | 0.01571805 | RUNX1 translocation partner 1 |
| MRGPRF-AS1 | 0.0157444 | MRGPRF antisense RNA 1 |
| CNGB3 | 0.01575758 | cyclic nucleotide gated channel beta 3 |
| EFCAB14 | 0.01582082 | EF-hand calcium binding domain 14 |
| LCMT2 | 0.01586825 | leucine carboxyl methyltransferase 2 |
| SET | 0.01587879 | SET nuclear proto-oncogene |
| COG4 | 0.01588933 | component of oligomeric golgi complex 4 |
| SNPH | 0.0159025 | syntaphilin |
| PID1 | 0.01593676 | phosphotyrosine interaction domain containing 1 |
| ECM2 | 0.01598155 | extracellular matrix protein 2 |
| DMRTC1B | 0.01611594 | DMRT like family C1B |
| HYAL1 | 0.01619499 | hyaluronoglucosaminidase 1 |
| ZW10 | 0.01629776 | zw10 kinetochore protein |
| SSFA2 | 0.01633992 | sperm specific antigen 2 |
| ZBTB7A | 0.01634256 | zinc finger and BTB domain containing 7A |
| MCFD2 | 0.01638999 | multiple coagulation factor deficiency 2 |
| GIPR | 0.01643742 | gastric inhibitory polypeptide receptor |
| SOX5 | 0.01645059 | SRY-box 5 |
| UBIAD1 | 0.01647694 | UbiA prenyltransferase domain containing 1 |
| C19orf68 | 0.01653755 | chromosome 19 open reading frame 68 |
| CEP120 | 0.01657444 | centrosomal protein 120 |
| NAIF1 | 0.01659025 | nuclear apoptosis inducing factor 1 |
| SSTR1 | 0.01666667 | somatostatin receptor 1 |
| KLHL10 | 0.01677734 | kelch like family member 10 |
| GPR132 | 0.01679051 | G protein-coupled receptor 132 |
| HPSE2 | 0.01682213 | heparanase 2 (inactive) |
| FGF1 | 0.01688801 | fibroblast growth factor 1 |
| EPAS1 | 0.01689065 | endothelial PAS domain protein 1 |
| LRRC3B | 0.01695916 | leucine rich repeat containing 3B |
| ZFYVE28 | 0.01703821 | zinc finger FYVE-type containing 28 |
| SLC8A1 | 0.01705665 | solute carrier family 8 member A1 |
| PCGF2 | 0.01720158 | polycomb group ring finger 2 |
| VPS13C | 0.01750988 | vacuolar protein sorting 13 homolog C |
| SGCZ | 0.01756522 | sarcoglycan zeta |
| NRIP3 | 0.01759684 | nuclear receptor interacting protein 3 |
| CD160 | 0.01760474 | CD160 molecule |
| SPCS2 | 0.01767062 | signal peptidase complex subunit 2 |
| ACTC1 | 0.01773123 | actin, alpha, cardiac muscle 1 |
| FBXO42 | 0.01775494 | F-box protein 42 |
| AVPR1A | 0.01782872 | arginine vasopressin receptor 1A |
| ALMS1 | 0.01793939 | ALMS1, centrosome and basal body associated protein |
| ARCN1 | 0.01811594 | archain 1 |
| NUDT16 | 0.01817655 | nudix hydrolase 16 |
| DRAP1 | 0.01824506 | DR1 associated protein 1 |
| HOXC13-AS | 0.01832148 | HOXC13 antisense RNA |
| SLA2 | 0.01838472 | Src like adaptor 2 |
| SNORD15A | 0.01840316 | small nucleolar RNA, C/D box 15A |
| VCL | 0.01842161 | vinculin |
| TXLNB | 0.01846113 | taxilin beta |
| ICE2 | 0.01853228 | interactor of little elongation complex ELL subunit 2 |
| BDKRB2 | 0.01859816 | bradykinin receptor B2 |
| DDX24 | 0.01860606 | DEAD-box helicase 24 |
| NME7 | 0.01862714 | NME/NM23 family member 7 |
| TLR7 | 0.01869038 | toll like receptor 7 |
| DENND2C | 0.01871146 | DENN domain containing 2C |
| FGF10 | 0.01876416 | fibroblast growth factor 10 |
| SRSF3 | 0.01884848 | serine and arginine rich splicing factor 3 |
| RABGEF1 | 0.01901713 | RAB guanine nucleotide exchange factor 1 |
| KCNH6 | 0.01903294 | potassium voltage-gated channel subfamily H member 6 |
| ZNF800 | 0.01920685 | zinc finger protein 800 |
| C1orf21 | 0.01922003 | chromosome 1 open reading frame 21 |
| CASKIN2 | 0.01957049 | CASK interacting protein 2 |
| CDC42EP3 | 0.0195942 | CDC42 effector protein 3 |
| RCCD1 | 0.01960211 | RCC1 domain containing 1 |
| MRM1 | 0.01965217 | mitochondrial rRNA methyltransferase 1 |
| GAS7 | 0.01966535 | growth arrest specific 7 |
| OTOP3 | 0.01972859 | otopetrin 3 |
| TMEM200B | 0.01979447 | transmembrane protein 200B |
| SPDEF | 0.01979974 | SAM pointed domain containing ETS transcription factor |
| GNGT2 | 0.01981555 | G protein subunit gamma transducin 2 |
| KDM5B | 0.01985244 | lysine demethylase 5B |
| KCNJ16 | 0.01991568 | potassium voltage-gated channel subfamily J member 16 |
| ADCK5 | 0.01992095 | aarF domain containing kinase 5 |
| FGFBP2 | 0.01993149 | fibroblast growth factor binding protein 2 |
| C7orf61 | 0.01993939 | chromosome 7 open reading frame 61 |
| PCP4L1 | 0.01995784 | Purkinje cell protein 4 like 1 |
| FKBP2 | 0.01998946 | FK506 binding protein 2 |
| KIAA1614 | 0.02011067 | KIAA1614 |
| SLC24A4 | 0.02018445 | solute carrier family 24 member 4 |
| EFEMP2 | 0.02018972 | EGF containing fibulin like extracellular matrix protein 2 |
| ARHGAP39 | 0.02019763 | Rho GTPase activating protein 39 |
| ZNF84 | 0.02022134 | zinc finger protein 84 |
| WWC2 | 0.02031621 | WW and C2 domain containing 2 |
| LOC101926959 | 0.02031884 | uncharacterized LOC101926959 |
| CILP | 0.02042161 | cartilage intermediate layer protein |
| ARVCF | 0.02050856 | armadillo repeat gene deleted in velocardiofacial syndrome |
| CCNI | 0.02055072 | cyclin I |
| SPTLC1 | 0.02063241 | serine palmitoyltransferase long chain base subunit 1 |
| B4GALT2 | 0.02072464 | beta-1,4-galactosyltransferase 2 |
| EIF4H | 0.02076153 | eukaryotic translation initiation factor 4H |
| MAR11 | 0.02078788 | membrane associated ring-CH-type finger 11 |
| C2orf66 | 0.02084058 | chromosome 2 open reading frame 66 |
| EID1 | 0.02096706 | EP300 interacting inhibitor of differentiation 1 |
| LCMT1-AS1 | 0.02099341 | LCMT1 antisense RNA 1 |
| LOC100507599 | 0.02105929 | uncharacterized LOC100507599 |
| CBX8 | 0.02116206 | chromobox 8 |
| GPRC5D | 0.02125955 | G protein-coupled receptor class C group 5 member D |
| C6orf223 | 0.02126746 | chromosome 6 open reading frame 223 |
| CX3CL1 | 0.02128063 | C-X3-C motif chemokine ligand 1 |
| FIGNL2 | 0.02132806 | fidgetin like 2 |
| KIAA1958 | 0.02134651 | KIAA1958 |
| PELI1 | 0.02135441 | pellino E3 ubiquitin protein ligase 1 |
| PTPRD-AS1 | 0.02139657 | PTPRD antisense RNA 1 |
| RASGRF2 | 0.02149671 | Ras protein specific guanine nucleotide releasing factor 2 |
| ZNRF1 | 0.02155468 | zinc and ring finger 1, E3 ubiquitin protein ligase |
| CHL1 | 0.02156258 | cell adhesion molecule L1 like |
| CHL1 | 0.02156258 | cell adhesion molecule L1 like |
| IGSF10 | 0.02158103 | immunoglobulin superfamily member 10 |
| KCNE5 | 0.02167325 | potassium voltage-gated channel subfamily E regulatory subunit 5 |
| LOC101927531 | 0.02185507 | uncharacterized LOC101927531 |
| PNPLA4 | 0.02198419 | patatin like phospholipase domain containing 4 |
| TVP23B | 0.022 | trans-golgi network vesicle protein 23 homolog B (S. cerevisiae) |
| PTGES3L-AARSD1 | 0.0220448 | PTGES3L-AARSD1 readthrough |
| ARMC4 | 0.02214493 | armadillo repeat containing 4 |
| CCR2 | 0.02223715 | C-C motif chemokine receptor 2 |
| CCR2 | 0.02223715 | C-C motif chemokine receptor 2 |
| FBXO7 | 0.0222556 | F-box protein 7 |
| PPP2R3C | 0.02227668 | protein phosphatase 2 regulatory subunit B''gamma |
| CD84 | 0.02242951 | CD84 molecule |
| FAM19A2 | 0.02255599 | family with sequence similarity 19 member A2, C-C motif chemokine like |
| LOC554174 | 0.0225639 | uncharacterized LOC554174 |
| SOX10 | 0.02259816 | SRY-box 10 |
| SERP1 | 0.02265349 | stress-associated endoplasmic reticulum protein 1 |
| IL5 | 0.0227141 | interleukin 5 |
| MIA2 | 0.02271937 | melanoma inhibitory activity 2 |
| ALG8 | 0.02273518 | ALG8, alpha-1,3-glucosyltransferase |
| PRSS35 | 0.02275626 | protease, serine 35 |
| CSDC2 | 0.02279315 | cold shock domain containing C2 |
| MIR4435-2HG | 0.02283267 | MIR4435-2 host gene |
| DTX4 | 0.02284848 | deltex E3 ubiquitin ligase 4 |
| C6orf25 | 0.02286166 | chromosome 6 open reading frame 25 |
| LACE1 | 0.02289065 | lactation elevated 1 |
| BNIP2 | 0.02293281 | BCL2 interacting protein 2 |
| LTA | 0.02298024 | lymphotoxin alpha |
| ESR1 | 0.0230303 | estrogen receptor 1 |
| EMC1 | 0.02312253 | ER membrane protein complex subunit 1 |
| FGF18 | 0.02314625 | fibroblast growth factor 18 |
| ATG4C | 0.02330962 | autophagy related 4C cysteine peptidase |
| MROH8 | 0.02333597 | maestro heat like repeat family member 8 |
| SLC27A4 | 0.02335441 | solute carrier family 27 member 4 |
| FGF6 | 0.02353887 | fibroblast growth factor 6 |
| FAM13B | 0.0235415 | family with sequence similarity 13 member B |
| RPS3 | 0.0235415 | ribosomal protein S3 |
| FXN | 0.02354941 | frataxin |
| PTPN22 | 0.02365481 | protein tyrosine phosphatase, non-receptor type 22 |
| FGF13 | 0.02380237 | fibroblast growth factor 13 |
| AIFM2 | 0.02380237 | apoptosis inducing factor, mitochondria associated 2 |
| TPM4 | 0.02393939 | tropomyosin 4 |
| PRSS3P2 | 0.02402108 | protease, serine 3 pseudogene 2 |
| SEMA6D | 0.0240448 | semaphorin 6D |
| HHIP | 0.02410013 | hedgehog interacting protein |
| OR9A4 | 0.02412385 | olfactory receptor family 9 subfamily A member 4 |
| LRP1 | 0.02414229 | LDL receptor related protein 1 |
| MICAL2 | 0.02420553 | microtubule associated monooxygenase, calponin and LIM domain containing 2 |
| APPBP2 | 0.02421344 | amyloid beta precursor protein binding protein 2 |
| STYXL1 | 0.02433465 | serine/threonine/tyrosine interacting-like 1 |
| NTNG2 | 0.02433992 | netrin G2 |
| TNNI3K | 0.02439789 | TNNI3 interacting kinase |
| LOC729950 | 0.02440053 | uncharacterized LOC729950 |
| SH3RF3 | 0.02448485 | SH3 domain containing ring finger 3 |
| TTYH2 | 0.02452964 | tweety family member 2 |
| SMAD4 | 0.02453228 | SMAD family member 4 |
| CSRP2 | 0.02475362 | cysteine and glycine rich protein 2 |
| PLEKHM2 | 0.0247668 | pleckstrin homology and RUN domain containing M2 |
| FAIM2 | 0.02478788 | Fas apoptotic inhibitory molecule 2 |
| ZSWIM5 | 0.02479315 | zinc finger SWIM-type containing 5 |
| SPRY2 | 0.02480632 | sprouty RTK signaling antagonist 2 |
| C6orf47 | 0.02481686 | chromosome 6 open reading frame 47 |
| NDUFA6 | 0.02484058 | NADH:ubiquinone oxidoreductase subunit A6 |
| RANBP6 | 0.02484848 | RAN binding protein 6 |
| LOC285147 | 0.02485375 | uncharacterized LOC285147 |
| SEC16B | 0.02497233 | SEC16 homolog B, endoplasmic reticulum export factor |
| AICDA | 0.0249776 | activation-induced cytidine deaminase |
| ATP7A | 0.02511726 | ATPase copper transporting alpha |
| MASP1 | 0.02512516 | mannan binding lectin serine peptidase 1 |
| PLAGL1 | 0.02514097 | PLAG1 like zinc finger 1 |
| SNORA64 | 0.02515152 | small nucleolar RNA, H/ACA box 64 |
| ARHGAP25 | 0.02518314 | Rho GTPase activating protein 25 |
| GPR4 | 0.02519895 | G protein-coupled receptor 4 |
| ZNF140 | 0.02521212 | zinc finger protein 140 |
| MROH9 | 0.02537813 | maestro heat like repeat family member 9 |
| CASR | 0.02539657 | calcium sensing receptor |
| PCDHA3 | 0.02540711 | protocadherin alpha 3 |
| TCTEX1D1 | 0.02543347 | Tctex1 domain containing 1 |
| TGFBI | 0.02546245 | transforming growth factor beta induced |
| ABCG2 | 0.02547826 | ATP binding cassette subfamily G member 2 (Junior blood group) |
| TPM1 | 0.02549407 | tropomyosin 1 (alpha) |
| PTPN5 | 0.02555995 | protein tyrosine phosphatase, non-receptor type 5 |
| PPME1 | 0.02562055 | protein phosphatase methylesterase 1 |
| SMIM10L1 | 0.02567589 | small integral membrane protein 10 like 1 |
| IL12RB1 | 0.02568379 | interleukin 12 receptor subunit beta 1 |
| NCAPD3 | 0.02577866 | non-SMC condensin II complex subunit D3 |
| TEX2 | 0.02582082 | testis expressed 2 |
| SNAPC5 | 0.02595784 | small nuclear RNA activating complex polypeptide 5 |
| SPIN4 | 0.02598419 | spindlin family member 4 |
| PAX1 | 0.02607905 | paired box 1 |
| LOC100505795 | 0.02624242 | uncharacterized LOC100505795 |
| GINS1 | 0.0262556 | GINS complex subunit 1 |
| SLA | 0.02627931 | Src-like-adaptor |
| SLFN11 | 0.02627931 | schlafen family member 11 |
| RAB35 | 0.0264058 | RAB35, member RAS oncogene family |
| VPS26B | 0.02643215 | VPS26, retromer complex component B |
| ABCC9 | 0.02645059 | ATP binding cassette subfamily C member 9 |
| LOC100131174 | 0.02646113 | uncharacterized LOC100131174 |
| MIR1-1HG | 0.02647431 | MIR1-1 host gene |
| GIMAP6 | 0.02650066 | GTPase, IMAP family member 6 |
| AKAP6 | 0.02657444 | A-kinase anchoring protein 6 |
| TTC7A | 0.02658762 | tetratricopeptide repeat domain 7A |
| PGAM2 | 0.02663241 | phosphoglycerate mutase 2 |
| ANKRD40 | 0.02668248 | ankyrin repeat domain 40 |
| PRDM1 | 0.02673254 | PR/SET domain 1 |
| TMEM156 | 0.02675362 | transmembrane protein 156 |
| PCDHGA9 | 0.02675626 | protocadherin gamma subfamily A, 9 |
| SPATA31E1 | 0.02678261 | SPATA31 subfamily E member 1 |
| TLL1 | 0.0268274 | tolloid like 1 |
| SPTB | 0.02688801 | spectrin beta, erythrocytic |
| GPRIN1 | 0.02689328 | G protein regulated inducer of neurite outgrowth 1 |
| PDE1C | 0.02690909 | phosphodiesterase 1C |
| PDE1C | 0.02690909 | phosphodiesterase 1C |
| PDE1C | 0.02690909 | phosphodiesterase 1C |
| TMEM203 | 0.02693017 | transmembrane protein 203 |
| ERC1 | 0.02709618 | ELKS/RAB6-interacting/CAST family member 1 |
| ARMC2 | 0.02715415 | armadillo repeat containing 2 |
| PCOLCE2 | 0.02731489 | procollagen C-endopeptidase enhancer 2 |
| RDH8 | 0.02734651 | retinol dehydrogenase 8 (all-trans) |
| TMPRSS7 | 0.02740711 | transmembrane protease, serine 7 |
| ABCB1 | 0.02743874 | ATP binding cassette subfamily B member 1 |
| HIST2H2AB | 0.02744928 | histone cluster 2, H2ab |
| SHISA3 | 0.02745191 | shisa family member 3 |
| HIRA | 0.02746772 | histone cell cycle regulator |
| CDADC1 | 0.02748353 | cytidine and dCMP deaminase domain containing 1 |
| CDR2 | 0.0274888 | cerebellar degeneration related protein 2 |
| BTBD2 | 0.02750198 | BTB domain containing 2 |
| EGR3 | 0.02750988 | early growth response 3 |
| OR2T33 | 0.02754941 | olfactory receptor family 2 subfamily T member 33 |
| TMCC2 | 0.02756785 | transmembrane and coiled-coil domain family 2 |
| CMKLR1 | 0.02765744 | chemerin chemokine-like receptor 1 |
| SYT3 | 0.0277444 | synaptotagmin 3 |
| SLC39A9 | 0.02795784 | solute carrier family 39 member 9 |
| PRTG | 0.02800264 | protogenin |
| CPM | 0.02807115 | carboxypeptidase M |
| CLEC9A | 0.02808959 | C-type lectin domain family 9 member A |
| CRBN | 0.0281502 | cereblon |
| FUT10 | 0.02824242 | fucosyltransferase 10 |
| FERMT3 | 0.02831884 | fermitin family member 3 |
| MPL | 0.0283531 | MPL proto-oncogene, thrombopoietin receptor |
| IGBP1 | 0.02839526 | immunoglobulin (CD79A) binding protein 1 |
| MYH14 | 0.02865086 | myosin, heavy chain 14, non-muscle |
| MBTPS2 | 0.02869829 | membrane bound transcription factor peptidase, site 2 |
| MAP1LC3B | 0.02871937 | microtubule associated protein 1 light chain 3 beta |
| LHFPL1 | 0.02873781 | lipoma HMGIC fusion partner-like 1 |
| CCIN | 0.02880369 | calicin |
| PRKCZ | 0.02880896 | protein kinase C zeta |
| TAGAP | 0.0290303 | T-cell activation RhoGTPase activating protein |
| ARNTL2 | 0.02914097 | aryl hydrocarbon receptor nuclear translocator like 2 |
| SNX7 | 0.02919368 | sorting nexin 7 |
| LINC00945 | 0.02920949 | long intergenic non-protein coding RNA 945 |
| ZC3H6 | 0.02928327 | zinc finger CCCH-type containing 6 |
| LURAP1L | 0.02932016 | leucine rich adaptor protein 1 like |
| C14orf180 | 0.02937022 | chromosome 14 open reading frame 180 |
| HIST3H2A | 0.02952306 | histone cluster 3, H2a |
| KLRD1 | 0.02957049 | killer cell lectin like receptor D1 |
| TXNL4B | 0.02972332 | thioredoxin like 4B |
| TRAT1 | 0.0298419 | T cell receptor associated transmembrane adaptor 1 |
| CD53 | 0.02989987 | CD53 molecule |
| PC | 0.02993412 | pyruvate carboxylase |
| FBXO34 | 0.03006851 | F-box protein 34 |
| NMRAL1 | 0.03011331 | NmrA like redox sensor 1 |
| AGL | 0.03037418 | amylo-alpha-1, 6-glucosidase, 4-alpha-glucanotransferase |
| ENTPD8 | 0.03042424 | ectonucleoside triphosphate diphosphohydrolase 8 |
| TTC5 | 0.03044796 | tetratricopeptide repeat domain 5 |
| JMJD4 | 0.03051383 | jumonji domain containing 4 |
| LILRA2 | 0.03057444 | leukocyte immunoglobulin like receptor A2 |
| LIME1 | 0.03057971 | Lck interacting transmembrane adaptor 1 |
| MYOCD | 0.03061397 | myocardin |
| MYOCD | 0.03061397 | myocardin |
| SRL | 0.0306693 | sarcalumenin |
| HIF3A | 0.03080369 | hypoxia inducible factor 3 alpha subunit |
| XYLT1 | 0.03083004 | xylosyltransferase 1 |
| LENG1 | 0.03087747 | leukocyte receptor cluster member 1 |
| ANK2 | 0.030917 | ankyrin 2, neuronal |
| RELN | 0.03092227 | reelin |
| UBQLN4 | 0.03097497 | ubiquilin 4 |
| PTPRJ | 0.0309776 | protein tyrosine phosphatase, receptor type J |
| OCM2 | 0.03102767 | oncomodulin 2 |
| SEC22B | 0.03104084 | SEC22 homolog B, vesicle trafficking protein (gene/pseudogene) |
| SCN1B | 0.03104875 | sodium voltage-gated channel beta subunit 1 |
| LINC00472 | 0.03108564 | long intergenic non-protein coding RNA 472 |
| PTGFRN | 0.03108564 | prostaglandin F2 receptor inhibitor |
| LILRB1 | 0.03116733 | leukocyte immunoglobulin like receptor B1 |
| SPATA32 | 0.03121739 | spermatogenesis associated 32 |
| C15orf54 | 0.03132806 | chromosome 15 open reading frame 54 |
| ETS1 | 0.03134124 | ETS proto-oncogene 1, transcription factor |
| MEX3A | 0.03137813 | mex-3 RNA binding family member A |
| KCNQ5 | 0.03146245 | potassium voltage-gated channel subfamily Q member 5 |
| SLC22A20 | 0.03147563 | solute carrier family 22 member 20 |
| APBB2 | 0.0314809 | amyloid beta precursor protein binding family B member 2 |
| C10orf35 | 0.03156258 | chromosome 10 open reading frame 35 |
| NAGS | 0.03174967 | N-acetylglutamate synthase |
| RFESD | 0.03178393 | Rieske Fe-S domain containing |
| TSPYL1 | 0.0317892 | TSPY like 1 |
| UBE2U | 0.03202635 | ubiquitin conjugating enzyme E2 U (putative) |
| MACF1 | 0.03211858 | microtubule-actin crosslinking factor 1 |
| TMEM100 | 0.0322108 | transmembrane protein 100 |
| SP3 | 0.03221871 | Sp3 transcription factor |
| UBE2H | 0.03225033 | ubiquitin conjugating enzyme E2 H |
| CPXM2 | 0.03225296 | carboxypeptidase X, M14 family member 2 |
| MIR99AHG | 0.0323531 | mir-99a-let-7c cluster host gene |
| FBXW2 | 0.03247431 | F-box and WD repeat domain containing 2 |
| APLN | 0.03255863 | apelin |
| RFX1 | 0.03262978 | regulatory factor X1 |
| MYL10 | 0.03279578 | myosin light chain 10 |
| NIPSNAP3B | 0.03282477 | nipsnap homolog 3B |
| UBE2J2 | 0.03283531 | ubiquitin conjugating enzyme E2 J2 |
| RPL13P5 | 0.03283531 | ribosomal protein L13 pseudogene 5 |
| MDH1B | 0.03288011 | malate dehydrogenase 1B |
| ZDHHC17 | 0.03289328 | zinc finger DHHC-type containing 17 |
| PIGL | 0.03295652 | phosphatidylinositol glycan anchor biosynthesis class L |
| CEP126 | 0.03304348 | centrosomal protein 126 |
| SGK1 | 0.03305138 | serum/glucocorticoid regulated kinase 1 |
| PRR34 | 0.03307246 | proline rich 34 |
| VNN2 | 0.03312516 | vanin 2 |
| RCC1L | 0.03313834 | RCC1 like |
| FIBCD1 | 0.03315415 | fibrinogen C domain containing 1 |
| GNG2 | 0.03319368 | G protein subunit gamma 2 |
| SNORD46 | 0.03324901 | small nucleolar RNA, C/D box 46 |
| RSPO2 | 0.03331489 | R-spondin 2 |
| BIN2 | 0.03339394 | bridging integrator 2 |
| LOC100506207 | 0.03347036 | uncharacterized LOC100506207 |
| KIF26B | 0.03359157 | kinesin family member 26B |
| RPF2 | 0.03366008 | ribosome production factor 2 homolog |
| VSTM2A | 0.03368906 | V-set and transmembrane domain containing 2A |
| CCDC170 | 0.03371014 | coiled-coil domain containing 170 |
| CHPF2 | 0.03374967 | chondroitin polymerizing factor 2 |
| GSK3B | 0.03391568 | glycogen synthase kinase 3 beta |
| PRAF2 | 0.03393412 | PRA1 domain family member 2 |
| FHDC1 | 0.03407115 | FH2 domain containing 1 |
| NKD1 | 0.03416074 | naked cuticle homolog 1 |
| DICER1 | 0.03428195 | dicer 1, ribonuclease III |
| FAM19A3 | 0.03428722 | family with sequence similarity 19 member A3, C-C motif chemokine like |
| LRRK2 | 0.03431884 | leucine rich repeat kinase 2 |
| MSC | 0.03436891 | musculin |
| ZBTB40 | 0.03443478 | zinc finger and BTB domain containing 40 |
| OR2Y1 | 0.03447694 | olfactory receptor family 2 subfamily Y member 1 |
| LRRC14 | 0.03450066 | leucine rich repeat containing 14 |
| PEX2 | 0.03450856 | peroxisomal biogenesis factor 2 |
| RMDN3 | 0.03452437 | regulator of microtubule dynamics 3 |
| ZNF70 | 0.03457708 | zinc finger protein 70 |
| KDF1 | 0.03469829 | keratinocyte differentiation factor 1 |
| DNASE1L3 | 0.03476416 | deoxyribonuclease 1 like 3 |
| ZNF322 | 0.03481423 | zinc finger protein 322 |
| LOC100128993 | 0.03481423 | uncharacterized LOC100128993 |
| CCDC120 | 0.0348274 | coiled-coil domain containing 120 |
| DDA1 | 0.03492227 | DET1 and DDB1 associated 1 |
| IQUB | 0.03506456 | IQ motif and ubiquitin domain containing |
| CEP68 | 0.03514097 | centrosomal protein 68 |
| CSF3R | 0.03514361 | colony stimulating factor 3 receptor |
| TRIM41 | 0.03520158 | tripartite motif containing 41 |
| EBF2 | 0.0352332 | early B-cell factor 2 |
| SHPK | 0.0353307 | sedoheptulokinase |
| RGS9 | 0.03535441 | regulator of G-protein signaling 9 |
| ECSCR | 0.03538603 | endothelial cell surface expressed chemotaxis and apoptosis regulator |
| C17orf77 | 0.03544401 | chromosome 17 open reading frame 77 |
| ZCCHC16 | 0.03545982 | zinc finger CCHC-type containing 16 |
| POU2AF1 | 0.03547036 | POU class 2 associating factor 1 |
| ZNF606 | 0.03550988 | zinc finger protein 606 |
| RWDD1 | 0.03554677 | RWD domain containing 1 |
| SLC38A7 | 0.03574967 | solute carrier family 38 member 7 |
| VAX1 | 0.03576812 | ventral anterior homeobox 1 |
| ZC3H3 | 0.0357892 | zinc finger CCCH-type containing 3 |
| DOK5 | 0.03579183 | docking protein 5 |
| IRF8 | 0.03588669 | interferon regulatory factor 8 |
| CLTA | 0.0359552 | clathrin light chain A |
| TMEM222 | 0.03600264 | transmembrane protein 222 |
| MED31 | 0.03601581 | mediator complex subunit 31 |
| CT45A3 | 0.03606324 | cancer/testis antigen family 45, member A3 |
| RERG | 0.03609486 | RAS like estrogen regulated growth inhibitor |
| MGARP | 0.03613702 | mitochondria localized glutamic acid rich protein |
| PRCC | 0.03616601 | papillary renal cell carcinoma (translocation-associated) |
| PCM1 | 0.03617918 | pericentriolar material 1 |
| UBQLN1 | 0.03617918 | ubiquilin 1 |
| FXYD2 | 0.03624769 | FXYD domain containing ion transport regulator 2 |
| WDR74 | 0.03625296 | WD repeat domain 74 |
| CCDC91 | 0.03637418 | coiled-coil domain containing 91 |
| COA1 | 0.03638735 | cytochrome c oxidase assembly factor 1 homolog |
| GLYATL1 | 0.03640843 | glycine-N-acyltransferase like 1 |
| SGCA | 0.03647167 | sarcoglycan alpha |
| NRG1 | 0.03652174 | neuregulin 1 |
| NRG1 | 0.03652174 | neuregulin 1 |
| NKX2-8 | 0.03662451 | NK2 homeobox 8 |
| SYPL2 | 0.03664295 | synaptophysin like 2 |
| TJP2 | 0.03688538 | tight junction protein 2 |
| ZKSCAN5 | 0.03688538 | zinc finger with KRAB and SCAN domains 5 |
| C1QTNF7 | 0.03689328 | C1q and tumor necrosis factor related protein 7 |
| LOC401554 | 0.03694071 | uncharacterized LOC401554 |
| MEG9 | 0.03705665 | maternally expressed 9 (non-protein coding) |
| THOC6 | 0.03706192 | THO complex 6 |
| IL2RB | 0.03710672 | interleukin 2 receptor subunit beta |
| SARS | 0.03720949 | seryl-tRNA synthetase |
| PIK3CD | 0.03727009 | phosphatidylinositol-4,5-bisphosphate 3-kinase catalytic subunit delta |
| TRPC5 | 0.03733333 | transient receptor potential cation channel subfamily C member 5 |
| PTGIS | 0.03734914 | prostaglandin I2 (prostacyclin) synthase |
| AP1S2 | 0.03737813 | adaptor related protein complex 1 sigma 2 subunit |
| TFAM | 0.03739657 | transcription factor A, mitochondrial |
| GPALPP1 | 0.03744137 | GPALPP motifs containing 1 |
| CD81 | 0.03751515 | CD81 molecule |
| TAB3 | 0.03753096 | TGF-beta activated kinase 1/MAP3K7 binding protein 3 |
| TRANK1 | 0.03754677 | tetratricopeptide repeat and ankyrin repeat containing 1 |
| DAGLB | 0.03760211 | diacylglycerol lipase beta |
| CREBBP | 0.03761265 | CREB binding protein |
| RNF215 | 0.03766008 | ring finger protein 215 |
| CHIT1 | 0.0377444 | chitinase 1 |
| LRRC15 | 0.03776812 | leucine rich repeat containing 15 |
| EIF3J | 0.0378498 | eukaryotic translation initiation factor 3 subunit J |
| DIEXF | 0.03797101 | digestive organ expansion factor homolog (zebrafish) |
| ACTN1-AS1 | 0.03807642 | ACTN1 antisense RNA 1 |
| MKX | 0.03809486 | mohawk homeobox |
| SLC25A19 | 0.03815547 | solute carrier family 25 member 19 |
| TMEM229A | 0.03835046 | transmembrane protein 229A |
| KLRB1 | 0.03838472 | killer cell lectin like receptor B1 |
| KDM4B | 0.03840316 | lysine demethylase 4B |
| MSRB3 | 0.03841634 | methionine sulfoxide reductase B3 |
| NANOS1 | 0.03844532 | nanos C2HC-type zinc finger 1 |
| DBH | 0.03845323 | dopamine beta-hydroxylase |
| NEK7 | 0.03852174 | NIMA related kinase 7 |
| NEK7 | 0.03852174 | NIMA related kinase 7 |
| CCDC148 | 0.03861133 | coiled-coil domain containing 148 |
| PRCP | 0.03861924 | prolylcarboxypeptidase |
| MEOX2 | 0.03862451 | mesenchyme homeobox 2 |
| FAM107A | 0.0386614 | family with sequence similarity 107 member A |
| FAM107A | 0.0386614 | family with sequence similarity 107 member A |
| PCGF5 | 0.03867194 | polycomb group ring finger 5 |
| SLC45A2 | 0.03877997 | solute carrier family 45 member 2 |
| COX11 | 0.03882213 | COX11, cytochrome c oxidase copper chaperone |
| ABCF1 | 0.03885112 | ATP binding cassette subfamily F member 1 |
| MED18 | 0.03890909 | mediator complex subunit 18 |
| ADAM21 | 0.03899341 | ADAM metallopeptidase domain 21 |
| MRPS27 | 0.03899868 | mitochondrial ribosomal protein S27 |
| PLD5 | 0.03901976 | phospholipase D family member 5 |
| MSL2 | 0.03918314 | male-specific lethal 2 homolog (Drosophila) |
| SLC25A27 | 0.03930171 | solute carrier family 25 member 27 |
| DNAJB8 | 0.03930962 | DnaJ heat shock protein family (Hsp40) member B8 |
| RGAG4 | 0.03931752 | retrotransposon gag domain containing 4 |
| E2F7 | 0.0394361 | E2F transcription factor 7 |
| HIST1H2BF | 0.03948617 | histone cluster 1, H2bf |
| FLJ12120 | 0.03951779 | uncharacterized LOC388439 |
| TSHZ3 | 0.03957312 | teashirt zinc finger homeobox 3 |
| UBB | 0.03957576 | ubiquitin B |
| TINAG | 0.03962319 | tubulointerstitial nephritis antigen |
| TNKS2-AS1 | 0.03963636 | TNKS2 antisense RNA 1 (head to head) |
| C1R | 0.03965744 | complement C1r subcomponent |
| UBR3 | 0.03966535 | ubiquitin protein ligase E3 component n-recognin 3 (putative) |
| ADARB2 | 0.03971805 | adenosine deaminase, RNA specific B2 (inactive) |
| RSPO3 | 0.0397444 | R-spondin 3 |
| PLEKHA8 | 0.03977075 | pleckstrin homology domain containing A8 |
| APBB1 | 0.03983399 | amyloid beta precursor protein binding family B member 1 |
| CIDEC | 0.03994993 | cell death inducing DFFA like effector c |
| SLC35E2 | 0.03996047 | solute carrier family 35 member E2 |
| IMP4 | 0.03998946 | IMP4 homolog, U3 small nucleolar ribonucleoprotein |
| MIR194-2HG | 0.0400527 | MIR194-2 host gene |
| TNS3 | 0.04009223 | tensin 3 |
| VSIG1 | 0.04013702 | V-set and immunoglobulin domain containing 1 |
| HERC3 | 0.04022134 | HECT and RLD domain containing E3 ubiquitin protein ligase 3 |
| SIPA1L3 | 0.04034783 | signal induced proliferation associated 1 like 3 |
| ZNF688 | 0.04035046 | zinc finger protein 688 |
| DNAJC19 | 0.04039789 | DnaJ heat shock protein family (Hsp40) member C19 |
| NOVA1 | 0.04041634 | NOVA alternative splicing regulator 1 |
| CLIP1 | 0.04051647 | CAP-Gly domain containing linker protein 1 |
| TNK1 | 0.04056653 | tyrosine kinase non receptor 1 |
| MGAT1 | 0.04056917 | mannosyl (alpha-1,3-)-glycoprotein beta-1,2-N-acetylglucosaminyltransferase |
| MAR6 | 0.04059025 | membrane associated ring-CH-type finger 6 |
| SEP10 | 0.0406166 | septin 10 |
| SCN2B | 0.04077997 | sodium voltage-gated channel beta subunit 2 |
| ADCY5 | 0.04083267 | adenylate cyclase 5 |
| EZH1 | 0.04091436 | enhancer of zeste 1 polycomb repressive complex 2 subunit |
| TSPAN11 | 0.0409776 | tetraspanin 11 |
| TSPAN11 | 0.0409776 | tetraspanin 11 |
| ERBB2 | 0.04100132 | erb-b2 receptor tyrosine kinase 2 |
| UFSP2 | 0.04100395 | UFM1 specific peptidase 2 |
| PIK3R2 | 0.04100659 | phosphoinositide-3-kinase regulatory subunit 2 |
| KCNQ3 | 0.0410303 | potassium voltage-gated channel subfamily Q member 3 |
| CCDC30 | 0.04112516 | coiled-coil domain containing 30 |
| SPTSSA | 0.04116206 | serine palmitoyltransferase small subunit A |
| ADAM33 | 0.04121739 | ADAM metallopeptidase domain 33 |
| EAF2 | 0.04126746 | ELL associated factor 2 |
| STMN3 | 0.04131225 | stathmin 3 |
| BACH2 | 0.04138603 | BTB domain and CNC homolog 2 |
| FOXD4L4 | 0.04148617 | forkhead box D4-like 4 |
| GKAP1 | 0.04153623 | G kinase anchoring protein 1 |
| CCDC8 | 0.04170751 | coiled-coil domain containing 8 |
| RHEBL1 | 0.04177866 | Ras homolog enriched in brain like 1 |
| TSSK6 | 0.04188406 | testis specific serine kinase 6 |
| HSF2BP | 0.04193149 | heat shock transcription factor 2 binding protein |
| FGF2 | 0.04196838 | fibroblast growth factor 2 |
| GSG1 | 0.04203953 | germ cell associated 1 |
| XKR4 | 0.0420527 | XK related 4 |
| CHST11 | 0.04221344 | carbohydrate (chondroitin 4) sulfotransferase 11 |
| ARHGEF11 | 0.04232675 | Rho guanine nucleotide exchange factor 11 |
| OR51T1 | 0.04237154 | olfactory receptor family 51 subfamily T member 1 |
| CSNK1E | 0.04240053 | casein kinase 1 epsilon |
| FAM122A | 0.04243215 | family with sequence similarity 122A |
| HERC1 | 0.04247431 | HECT and RLD domain containing E3 ubiquitin protein ligase family member 1 |
| PQLC1 | 0.0425191 | PQ loop repeat containing 1 |
| CTSK | 0.04252437 | cathepsin K |
| LY6K | 0.04257708 | lymphocyte antigen 6 complex, locus K |
| F3 | 0.04269829 | coagulation factor III, tissue factor |
| CEP131 | 0.04280896 | centrosomal protein 131 |
| XAGE5 | 0.04286693 | X antigen family member 5 |
| APOL2 | 0.04287484 | apolipoprotein L2 |
| BTF3 | 0.04296443 | basic transcription factor 3 |
| PPP1R12C | 0.04303557 | protein phosphatase 1 regulatory subunit 12C |
| CCDC96 | 0.04319631 | coiled-coil domain containing 96 |
| ZNF683 | 0.04321476 | zinc finger protein 683 |
| SOX2 | 0.04331225 | SRY-box 2 |
| USP53 | 0.04344401 | ubiquitin specific peptidase 53 |
| TRAF2 | 0.04358103 | TNF receptor associated factor 2 |
| ZNF182 | 0.04363636 | zinc finger protein 182 |
| STAT5B | 0.04370487 | signal transducer and activator of transcription 5B |
| XPO7 | 0.04372069 | exportin 7 |
| PRPF19 | 0.04378129 | pre-mRNA processing factor 19 |
| PPP1R21 | 0.04379447 | protein phosphatase 1 regulatory subunit 21 |
| BCL2L2 | 0.0437971 | BCL2 like 2 |
| TMEM116 | 0.04381291 | transmembrane protein 116 |
| DPY19L2 | 0.04385507 | dpy-19 like 2 |
| BOD1 | 0.04386825 | biorientation of chromosomes in cell division 1 |
| IK | 0.04391831 | IK cytokine, down-regulator of HLA II |
| TBC1D31 | 0.04392885 | TBC1 domain family member 31 |
| KHDRBS2 | 0.04395784 | KH RNA binding domain containing, signal transduction associated 2 |
| FKBP8 | 0.04399473 | FK506 binding protein 8 |
| CADM2 | 0.04410804 | cell adhesion molecule 2 |
| FAM180B | 0.04434519 | family with sequence similarity 180 member B |
| ZNF512B | 0.04436627 | zinc finger protein 512B |
| MAT2B | 0.04437418 | methionine adenosyltransferase 2B |
| NR1H2 | 0.04444532 | nuclear receptor subfamily 1 group H member 2 |
| ARL4C | 0.04447694 | ADP ribosylation factor like GTPase 4C |
| S100B | 0.04453491 | S100 calcium binding protein B |
| GNAO1 | 0.04455336 | G protein subunit alpha o1 |
| CPN1 | 0.04459552 | carboxypeptidase N subunit 1 |
| SGCG | 0.04464032 | sarcoglycan gamma |
| GPAT4 | 0.0447747 | glycerol-3-phosphate acyltransferase 4 |
| RPL9 | 0.04477997 | ribosomal protein L9 |
| C3orf18 | 0.04485375 | chromosome 3 open reading frame 18 |
| CSF1 | 0.04488538 | colony stimulating factor 1 |
| DNAJC3 | 0.04489592 | DnaJ heat shock protein family (Hsp40) member C3 |
| NDUFS8 | 0.04505665 | NADH:ubiquinone oxidoreductase core subunit S8 |
| SUSD3 | 0.0450751 | sushi domain containing 3 |
| SRGAP3 | 0.0452253 | SLIT-ROBO Rho GTPase activating protein 3 |
| CA9 | 0.04526219 | carbonic anhydrase 9 |
| IL26 | 0.04530171 | interleukin 26 |
| IFT57 | 0.04538076 | intraflagellar transport 57 |
| P2RY14 | 0.04539657 | purinergic receptor P2Y14 |
| ZNF25 | 0.04542819 | zinc finger protein 25 |
| EOGT | 0.04545982 | EGF domain specific O-linked N-acetylglucosamine transferase |
| LSAMP | 0.04553623 | limbic system-associated membrane protein |
| RGL1 | 0.04562846 | ral guanine nucleotide dissociation stimulator like 1 |
| PON3 | 0.045639 | paraoxonase 3 |
| MAPK9 | 0.04568906 | mitogen-activated protein kinase 9 |
| ST8SIA4 | 0.04571278 | ST8 alpha-N-acetyl-neuraminide alpha-2,8-sialyltransferase 4 |
| ELSPBP1 | 0.04575758 | epididymal sperm binding protein 1 |
| ZNF213 | 0.04578129 | zinc finger protein 213 |
| PPP1R2 | 0.04582345 | protein phosphatase 1 regulatory inhibitor subunit 2 |
| BEST1 | 0.04583399 | bestrophin 1 |
| GMEB2 | 0.04601845 | glucocorticoid modulatory element binding protein 2 |
| CCDC53 | 0.04601845 | coiled-coil domain containing 53 |
| ANGPTL1 | 0.04607378 | angiopoietin like 1 |
| NDUFB3 | 0.04607378 | NADH:ubiquinone oxidoreductase subunit B3 |
| GYPE | 0.0460975 | glycophorin E (MNS blood group) |
| SMAD2 | 0.04610804 | SMAD family member 2 |
| RBM48 | 0.04611331 | RNA binding motif protein 48 |
| RBM48 | 0.04611331 | RNA binding motif protein 48 |
| SUSD5 | 0.04620817 | sushi domain containing 5 |
| HN1L | 0.04621871 | hematological and neurological expressed 1 like |
| EIF4G3 | 0.04627931 | eukaryotic translation initiation factor 4 gamma 3 |
| FGL1 | 0.04647958 | fibrinogen like 1 |
| LIMK2 | 0.04655072 | LIM domain kinase 2 |
| STPG1 | 0.04659025 | sperm tail PG-rich repeat containing 1 |
| SLC40A1 | 0.04659289 | solute carrier family 40 member 1 |
| FAM110B | 0.04663241 | family with sequence similarity 110 member B |
| IRS2 | 0.04667984 | insulin receptor substrate 2 |
| SERPINB6 | 0.04670356 | serpin family B member 6 |
| PLCXD3 | 0.0467141 | phosphatidylinositol specific phospholipase C X domain containing 3 |
| STRIP1 | 0.04685112 | striatin interacting protein 1 |
| TWISTNB | 0.04685903 | TWIST neighbor |
| COX8A | 0.046917 | cytochrome c oxidase subunit 8A |
| VGF | 0.04692227 | VGF nerve growth factor inducible |
| PI4K2A | 0.04700922 | phosphatidylinositol 4-kinase type 2 alpha |
| RNF13 | 0.04702503 | ring finger protein 13 |
| PRDM8 | 0.0470303 | PR/SET domain 8 |
| ZNF474 | 0.04704084 | zinc finger protein 474 |
| F8 | 0.04707773 | coagulation factor VIII |
| SPON1 | 0.04715152 | spondin 1 |
| WBSCR17 | 0.0471726 | Williams-Beuren syndrome chromosome region 17 |
| ANP32B | 0.04717523 | acidic nuclear phosphoprotein 32 family member B |
| RPS6KC1 | 0.0472859 | ribosomal protein S6 kinase C1 |
| RUNX1 | 0.04731225 | runt related transcription factor 1 |
| TAF6L | 0.04745982 | TATA-box binding protein associated factor 6 like |
| DNAAF5 | 0.04745982 | dynein axonemal assembly factor 5 |
| B4GALT1 | 0.04749407 | beta-1,4-galactosyltransferase 1 |
| ATP5S | 0.04751515 | ATP synthase, H+ transporting, mitochondrial Fo complex subunit s (factor B) |
| RDH13 | 0.04752042 | retinol dehydrogenase 13 |
| LRRC18 | 0.04752306 | leucine rich repeat containing 18 |
| PPIC | 0.04761792 | peptidylprolyl isomerase C |
| GTF2A2 | 0.04765217 | general transcription factor IIA subunit 2 |
| ROBO1 | 0.04776548 | roundabout guidance receptor 1 |
| HDX | 0.04778393 | highly divergent homeobox |
| MYH2 | 0.04780764 | myosin heavy chain 2 |
| FAF1 | 0.04783399 | Fas associated factor 1 |
| ARHGDIG | 0.0478498 | Rho GDP dissociation inhibitor gamma |
| NEUROD6 | 0.04786034 | neuronal differentiation 6 |
| NCDN | 0.04798155 | neurochondrin |
| RPRD1A | 0.04808432 | regulation of nuclear pre-mRNA domain containing 1A |
| NLRP3 | 0.04822925 | NLR family pyrin domain containing 3 |
| FBXO36-IT1 | 0.04839526 | FBXO36 intronic transcript 1 |
| POLR1B | 0.04840053 | RNA polymerase I subunit B |
| CHN2 | 0.04843478 | chimerin 2 |
| GRIK5 | 0.04847958 | glutamate ionotropic receptor kainate type subunit 5 |
| MAP7D3 | 0.04849539 | MAP7 domain containing 3 |
| AGPAT1 | 0.04854018 | 1-acylglycerol-3-phosphate O-acyltransferase 1 |
| MAMLD1 | 0.04890382 | mastermind like domain containing 1 |
| CBARP | 0.04891173 | CACN beta subunit associated regulatory protein |
| LYSMD2 | 0.04899868 | LysM domain containing 2 |
| IER3 | 0.04917787 | immediate early response 3 |
| VIPR2 | 0.04919104 | vasoactive intestinal peptide receptor 2 |
| GFOD2 | 0.04923057 | glucose-fructose oxidoreductase domain containing 2 |
| RASA1 | 0.04925955 | RAS p21 protein activator 1 |
| TRAPPC6B | 0.04932016 | trafficking protein particle complex 6B |
| TRDN | 0.04945191 | triadin |
| BCL7A | 0.04948617 | BCL tumor suppressor 7A |
| OR4S1 | 0.04953887 | olfactory receptor family 4 subfamily S member 1 |
| DMD | 0.04966008 | dystrophin |
| PITX2 | 0.04966798 | paired like homeodomain 2 |
| CPE | 0.04972859 | carboxypeptidase E |
| PYGM | 0.04978129 | phosphorylase, glycogen, muscle |
| RPL30 | 0.04979974 | ribosomal protein L30 |
| GUCA1A | 0.04980501 | guanylate cyclase activator 1A |
| CPA3 | 0.04980501 | carboxypeptidase A3 |
| B3GALNT2 | 0.04986298 | beta-1,3-N-acetylgalactosaminyltransferase 2 |
| OGN | 0.04988933 | osteoglycin |
| VNN1 | 0.05003953 | vanin 1 |
| PNLIPRP1 | 0.05023715 | pancreatic lipase related protein 1 |
| FEZ2 | 0.05031094 | fasciculation and elongation protein zeta 2 |
| FLNA | 0.05032148 | filamin A |
| LYRM5 | 0.05032938 | LYR motif containing 5 |
| EIF3F | 0.050361 | eukaryotic translation initiation factor 3 subunit F |
| SDPR | 0.05042688 | serum deprivation response |
| PPP3CB | 0.0504585 | protein phosphatase 3 catalytic subunit beta |
| KIAA0040 | 0.05062714 | KIAA0040 |
| SOBP | 0.05064295 | sine oculis binding protein homolog |
| SOBP | 0.05064295 | sine oculis binding protein homolog |
| LCP2 | 0.05065613 | lymphocyte cytosolic protein 2 |
| TAS2R4 | 0.05067457 | taste 2 receptor member 4 |
| KIAA0930 | 0.05072464 | KIAA0930 |
| ADRB3 | 0.05088801 | adrenoceptor beta 3 |
| UFL1 | 0.05100132 | UFM1 specific ligase 1 |
| SLC24A3 | 0.0510224 | solute carrier family 24 member 3 |
| APOBEC2 | 0.05103821 | apolipoprotein B mRNA editing enzyme catalytic subunit 2 |
| RAB3B | 0.05106192 | RAB3B, member RAS oncogene family |
| ZNF540 | 0.051083 | zinc finger protein 540 |
| GHITM | 0.05117787 | growth hormone inducible transmembrane protein |
| THAP1 | 0.05118577 | THAP domain containing 1 |
| SEP14 | 0.0512859 | septin 14 |
| SIX3 | 0.05130435 | SIX homeobox 3 |
| PGR | 0.05136232 | progesterone receptor |
| KCND3-AS1 | 0.05139657 | KCND3 antisense RNA 1 |
| NLRC5 | 0.05150461 | NLR family CARD domain containing 5 |
| CD8A | 0.05155468 | CD8a molecule |
| IARS | 0.05163373 | isoleucyl-tRNA synthetase |
| BORCS7 | 0.05166798 | BLOC-1 related complex subunit 7 |
| ANGPTL7 | 0.05167589 | angiopoietin like 7 |
| PIFO | 0.05167852 | primary cilia formation |
| BEST3 | 0.05168116 | bestrophin 3 |
| TCP11L2 | 0.05168116 | t-complex 11 like 2 |
| RNPEPL1 | 0.05168379 | arginyl aminopeptidase like 1 |
| ST3GAL6 | 0.05181555 | ST3 beta-galactoside alpha-2,3-sialyltransferase 6 |
| CNTN2 | 0.05185244 | contactin 2 |
| BVES-AS1 | 0.05187879 | BVES antisense RNA 1 |
| DOCK10 | 0.0518946 | dedicator of cytokinesis 10 |
| PLCL1 | 0.05196574 | phospholipase C like 1 |
| RBM15 | 0.05221607 | RNA binding motif protein 15 |
| HIST1H2AM | 0.05222398 | histone cluster 1, H2am |
| C2orf40 | 0.05231094 | chromosome 2 open reading frame 40 |
| LPP | 0.05232411 | LIM domain containing preferred translocation partner in lipoma |
| LPP | 0.05232411 | LIM domain containing preferred translocation partner in lipoma |
| MAML2 | 0.05232938 | mastermind like transcriptional coactivator 2 |
| EBLN2 | 0.052361 | endogenous Bornavirus-like nucleoprotein 2 |
| RCHY1 | 0.05243478 | ring finger and CHY zinc finger domain containing 1 |
| CMAHP | 0.05248221 | cytidine monophospho-N-acetylneuraminic acid hydroxylase, pseudogene |
| SPANXN3 | 0.05251383 | SPANX family member N3 |
| C10orf107 | 0.05251383 | chromosome 10 open reading frame 107 |
| CHMP1A | 0.05257708 | charged multivesicular body protein 1A |
| PLCD4 | 0.05260343 | phospholipase C delta 4 |
| SLC26A1 | 0.05270883 | solute carrier family 26 member 1 |
| WASF3 | 0.05288538 | WAS protein family member 3 |
| KRTAP13-4 | 0.0529249 | keratin associated protein 13-4 |
| STX2 | 0.05308037 | syntaxin 2 |
| C21orf2 | 0.05316469 | chromosome 21 open reading frame 2 |
| SLMAP | 0.05328327 | sarcolemma associated protein |
| HNRNPA1L2 | 0.05329908 | heterogeneous nuclear ribonucleoprotein A1-like 2 |
| ATP1A3 | 0.05338867 | ATPase Na+/K+ transporting subunit alpha 3 |
| BTF3L4 | 0.05338867 | basic transcription factor 3 like 4 |
| ZNF480 | 0.0533913 | zinc finger protein 480 |
| PCNP | 0.05341502 | PEST proteolytic signal containing nuclear protein |
| NAA40 | 0.05349671 | N(alpha)-acetyltransferase 40, NatD catalytic subunit |
| PTPRD | 0.05355204 | protein tyrosine phosphatase, receptor type D |
| EAPP | 0.05355468 | E2F associated phosphoprotein |
| ZNF157 | 0.05359684 | zinc finger protein 157 |
| TMEM35A | 0.05365744 | transmembrane protein 35A |
| STX12 | 0.05369697 | syntaxin 12 |
| LDB1 | 0.05379974 | LIM domain binding 1 |
| HAND2 | 0.05380501 | heart and neural crest derivatives expressed 2 |
| KCNH2 | 0.05382609 | potassium voltage-gated channel subfamily H member 2 |
| LOC388882 | 0.05386561 | uncharacterized LOC388882 |
| PAIP2 | 0.05387615 | poly(A) binding protein interacting protein 2 |
| ZBTB12 | 0.05387879 | zinc finger and BTB domain containing 12 |
| FAM120C | 0.05395784 | family with sequence similarity 120C |
| LOC101927100 | 0.05396047 | uncharacterized LOC101927100 |
| RPS6KA6 | 0.05399473 | ribosomal protein S6 kinase A6 |
| SRF | 0.05400264 | serum response factor |
| CLEC7A | 0.05403426 | C-type lectin domain family 7 member A |
| C1QTNF1-AS1 | 0.05403689 | C1QTNF1 antisense RNA 1 |
| ATP6V1B2 | 0.05405007 | ATPase H+ transporting V1 subunit B2 |
| DUSP9 | 0.05412648 | dual specificity phosphatase 9 |
| NPR1 | 0.05413439 | natriuretic peptide receptor 1 |
| SYNE2 | 0.05413702 | spectrin repeat containing nuclear envelope protein 2 |
| PTK7 | 0.05414493 | protein tyrosine kinase 7 (inactive) |
| EMP3 | 0.05416074 | epithelial membrane protein 3 |
| NPB | 0.05424769 | neuropeptide B |
| WAS | 0.0542635 | Wiskott-Aldrich syndrome |
| FAM155A | 0.05433202 | family with sequence similarity 155 member A |
| FAM155A | 0.05433202 | family with sequence similarity 155 member A |
| TMEM106B | 0.05446113 | transmembrane protein 106B |
| CTU1 | 0.05450856 | cytosolic thiouridylase subunit 1 |
| CYP11B2 | 0.05454018 | cytochrome P450 family 11 subfamily B member 2 |
| CFL2 | 0.05462714 | cofilin 2 |
| PRELP | 0.05472727 | proline and arginine rich end leucine rich repeat protein |
| PPP5C | 0.05480105 | protein phosphatase 5 catalytic subunit |
| C14orf28 | 0.05491173 | chromosome 14 open reading frame 28 |
| EID2B | 0.05491436 | EP300 interacting inhibitor of differentiation 2B |
| PRR7 | 0.05496179 | proline rich 7 (synaptic) |
| HPGDS | 0.05508564 | hematopoietic prostaglandin D synthase |
| PES1 | 0.05511462 | pescadillo ribosomal biogenesis factor 1 |
| NXN | 0.0552253 | nucleoredoxin |
| DGAT1 | 0.05530171 | diacylglycerol O-acyltransferase 1 |
| RNF146 | 0.05534124 | ring finger protein 146 |
| SLC8A3 | 0.05541502 | solute carrier family 8 member A3 |
| KCNG4 | 0.0555415 | potassium voltage-gated channel modifier subfamily G member 4 |
| SLIT3 | 0.05555468 | slit guidance ligand 3 |
| LOC101926941 | 0.05556258 | uncharacterized LOC101926941 |
| SAMD12 | 0.05558366 | sterile alpha motif domain containing 12 |
| PWP2 | 0.05563109 | PWP2 periodic tryptophan protein homolog (yeast) |
| PCDHB18P | 0.05567325 | protocadherin beta 18 pseudogene |
| ANG | 0.05581028 | angiogenin |
| RRAD | 0.05581291 | RRAD, Ras related glycolysis inhibitor and calcium channel regulator |
| ATP2B1 | 0.05593676 | ATPase plasma membrane Ca2+ transporting 1 |
| SENP2 | 0.05599736 | SUMO1/sentrin/SMT3 specific peptidase 2 |
| RFX3 | 0.05607905 | regulatory factor X3 |
| RFX3 | 0.05607905 | regulatory factor X3 |
| ZNF483 | 0.05611594 | zinc finger protein 483 |
| TMCC1 | 0.05632411 | transmembrane and coiled-coil domain family 1 |
| CREB1 | 0.05632675 | cAMP responsive element binding protein 1 |
| GSPT2 | 0.05649539 | G1 to S phase transition 2 |
| NAP1L5 | 0.05649802 | nucleosome assembly protein 1 like 5 |
| ROM1 | 0.0565112 | retinal outer segment membrane protein 1 |
| NUDT14 | 0.05651383 | nudix hydrolase 14 |
| LOC101926934 | 0.05656917 | uncharacterized LOC101926934 |
| EPHX4 | 0.05679578 | epoxide hydrolase 4 |
| MOAP1 | 0.05684848 | modulator of apoptosis 1 |
| MTG2 | 0.05686693 | mitochondrial ribosome associated GTPase 2 |
| GDPD4 | 0.05689328 | glycerophosphodiester phosphodiesterase domain containing 4 |
| FCGR2B | 0.05697497 | Fc fragment of IgG receptor IIb |
| ZSWIM4 | 0.05720949 | zinc finger SWIM-type containing 4 |
| PLAA | 0.0573386 | phospholipase A2 activating protein |
| EIF4G2 | 0.05734914 | eukaryotic translation initiation factor 4 gamma 2 |
| OR2W3 | 0.0573834 | olfactory receptor family 2 subfamily W member 3 |
| NAV3 | 0.05741238 | neuron navigator 3 |
| C4orf3 | 0.05753623 | chromosome 4 open reading frame 3 |
| KLHL36 | 0.05766008 | kelch like family member 36 |
| HIST1H2BO | 0.05777075 | histone cluster 1, H2bo |
| ZEB2 | 0.05781028 | zinc finger E-box binding homeobox 2 |
| ZEB2 | 0.05781028 | zinc finger E-box binding homeobox 2 |
| FAM104B | 0.0578946 | family with sequence similarity 104 member B |
| FAM104B | 0.0578946 | family with sequence similarity 104 member B |
| NFIA | 0.0580527 | nuclear factor I A |
| NFIA | 0.0580527 | nuclear factor I A |
| ECI2 | 0.05821344 | enoyl-CoA delta isomerase 2 |
| SNX2 | 0.05826877 | sorting nexin 2 |
| ACO1 | 0.05831621 | aconitase 1 |
| GNB4 | 0.05844269 | G protein subunit beta 4 |
| IKZF2 | 0.05847431 | IKAROS family zinc finger 2 |
| IKZF2 | 0.05847431 | IKAROS family zinc finger 2 |
| ADNP2 | 0.05847958 | ADNP homeobox 2 |
| ATP8B2 | 0.05850856 | ATPase phospholipid transporting 8B2 |
| CRADD | 0.05852437 | CASP2 and RIPK1 domain containing adaptor with death domain |
| NMNAT3 | 0.05868511 | nicotinamide nucleotide adenylyltransferase 3 |
| MAPK8 | 0.05874835 | mitogen-activated protein kinase 8 |
| GBGT1 | 0.05884321 | globoside alpha-1,3-N-acetylgalactosaminyltransferase 1 |
| BIN1 | 0.05885375 | bridging integrator 1 |
| CYB561D1 | 0.05888538 | cytochrome b561 family member D1 |
| MED7 | 0.05890909 | mediator complex subunit 7 |
| PODN | 0.05896706 | podocan |
| GLI2 | 0.05901186 | GLI family zinc finger 2 |
| KBTBD7 | 0.05905929 | kelch repeat and BTB domain containing 7 |
| ZFP36 | 0.05919368 | ZFP36 ring finger protein |
| ZAK | 0.05920685 | sterile alpha motif and leucine zipper containing kinase AZK |
| SOHLH2 | 0.05947563 | spermatogenesis and oogenesis specific basic helix-loop-helix 2 |
| COLCA2 | 0.0594888 | colorectal cancer associated 2 |
| SNTG2 | 0.05956785 | syntrophin gamma 2 |
| C1orf194 | 0.05970751 | chromosome 1 open reading frame 194 |
| CNIH1 | 0.05981291 | cornichon family AMPA receptor auxiliary protein 1 |
| MTTP | 0.05986561 | microsomal triglyceride transfer protein |
| CACNB2 | 0.05991831 | calcium voltage-gated channel auxiliary subunit beta 2 |
| BCL2 | 0.05995257 | BCL2, apoptosis regulator |
| RBP4 | 0.0599552 | retinol binding protein 4 |
| VEGFA | 0.06001318 | vascular endothelial growth factor A |
| LOC100130458 | 0.06007378 | uncharacterized LOC100130458 |
| ZNF692 | 0.0600975 | zinc finger protein 692 |
| CNTNAP5 | 0.06013175 | contactin associated protein like 5 |
| AGTR2 | 0.06029776 | angiotensin II receptor type 2 |
| AIDA | 0.0603083 | axin interactor, dorsalization associated |
| AP1M1 | 0.06032411 | adaptor related protein complex 1 mu 1 subunit |
| CCDC129 | 0.06033992 | coiled-coil domain containing 129 |
| ATL3 | 0.06037418 | atlastin GTPase 3 |
| MAPT | 0.0604058 | microtubule associated protein tau |
| CHRDL2 | 0.06041897 | chordin like 2 |
| PLEKHH2 | 0.06052174 | pleckstrin homology, MyTH4 and FERM domain containing H2 |
| PHYHD1 | 0.06056653 | phytanoyl-CoA dioxygenase domain containing 1 |
| RASSF8 | 0.0606693 | Ras association domain family member 8 |
| CTTNBP2 | 0.06073254 | cortactin binding protein 2 |
| TLR8 | 0.0607747 | toll like receptor 8 |
| ACSL4 | 0.06078261 | acyl-CoA synthetase long-chain family member 4 |
| RERE | 0.06080896 | arginine-glutamic acid dipeptide repeats |
| OR2W1 | 0.06084058 | olfactory receptor family 2 subfamily W member 1 |
| ATP6AP1 | 0.06084058 | ATPase H+ transporting accessory protein 1 |
| BSCL2 | 0.06084321 | BSCL2, seipin lipid droplet biogenesis associated |
| PHF3 | 0.06088538 | PHD finger protein 3 |
| FAM13C | 0.06089065 | family with sequence similarity 13 member C |
| AIM1 | 0.06104084 | absent in melanoma 1 |
| ZNF625 | 0.06106192 | zinc finger protein 625 |
| RCAN1 | 0.06107246 | regulator of calcineurin 1 |
| ZDHHC14 | 0.06109354 | zinc finger DHHC-type containing 14 |
| TNKS2 | 0.06109618 | tankyrase 2 |
| PLEKHG4B | 0.06115152 | pleckstrin homology and RhoGEF domain containing G4B |
| THAP2 | 0.06119895 | THAP domain containing 2 |
| FOXI2 | 0.06119895 | forkhead box I2 |
| CASD1 | 0.06124111 | CAS1 domain containing 1 |
| MMP16 | 0.06125428 | matrix metallopeptidase 16 |
| RAPGEF4 | 0.0613386 | Rap guanine nucleotide exchange factor 4 |
| CRISP3 | 0.06137022 | cysteine rich secretory protein 3 |
| NCK1 | 0.06137549 | NCK adaptor protein 1 |
| LPAR6 | 0.06140448 | lysophosphatidic acid receptor 6 |
| SOSTDC1 | 0.06143874 | sclerostin domain containing 1 |
| WDR53 | 0.0614888 | WD repeat domain 53 |
| RABL3 | 0.06153096 | RAB, member of RAS oncogene family like 3 |
| BTG4 | 0.06160474 | BTG anti-proliferation factor 4 |
| C22orf39 | 0.06171278 | chromosome 22 open reading frame 39 |
| FOXP2 | 0.06191304 | forkhead box P2 |
| FOXP2 | 0.06191304 | forkhead box P2 |
| CNTLN | 0.06193939 | centlein |
| SLC4A2 | 0.06203689 | solute carrier family 4 member 2 |
| NR3C1 | 0.06208432 | nuclear receptor subfamily 3 group C member 1 |
| PDZD4 | 0.06213702 | PDZ domain containing 4 |
| EPS8 | 0.06213702 | epidermal growth factor receptor pathway substrate 8 |
| C1S | 0.06215547 | complement component 1, s subcomponent |
| CPO | 0.06218709 | carboxypeptidase O |
| STK11 | 0.06220026 | serine/threonine kinase 11 |
| RPP40 | 0.0622029 | ribonuclease P/MRP subunit p40 |
| AKAP13 | 0.06222925 | A-kinase anchoring protein 13 |
| TMOD3 | 0.06260606 | tropomodulin 3 |
| RAP1A | 0.06269565 | RAP1A, member of RAS oncogene family |
| SNAP47 | 0.06270356 | synaptosome associated protein 47 |
| GRAMD3 | 0.0628274 | GRAM domain containing 3 |
| MXD3 | 0.06292227 | MAX dimerization protein 3 |
| NDUFB5 | 0.06295652 | NADH:ubiquinone oxidoreductase subunit B5 |
| GLRB | 0.06298287 | glycine receptor beta |
| LPAR1 | 0.06306192 | lysophosphatidic acid receptor 1 |
| LINC00290 | 0.06308827 | long intergenic non-protein coding RNA 290 |
| NAALAD2 | 0.06309618 | N-acetylated alpha-linked acidic dipeptidase 2 |
| ZSCAN16 | 0.06310145 | zinc finger and SCAN domain containing 16 |
| AP3S1 | 0.06310672 | adaptor related protein complex 3 sigma 1 subunit |
| VARS2 | 0.06313307 | valyl-tRNA synthetase 2, mitochondrial |
| VMAC | 0.06329117 | vimentin-type intermediate filament associated coiled-coil protein |
| GPC6 | 0.06343347 | glypican 6 |
| CHST15 | 0.0635942 | carbohydrate (N-acetylgalactosamine 4-sulfate 6-O) sulfotransferase 15 |
| CARD16 | 0.06364427 | caspase recruitment domain family member 16 |
| EEF1A1 | 0.06364427 | eukaryotic translation elongation factor 1 alpha 1 |
| RNF212 | 0.06366798 | ring finger protein 212 |
| ASL | 0.06374704 | argininosuccinate lyase |
| BRMS1L | 0.06379183 | breast cancer metastasis-suppressor 1-like |
| KIF24 | 0.06382345 | kinesin family member 24 |
| CSRNP3 | 0.06391041 | cysteine and serine rich nuclear protein 3 |
| RAB2B | 0.06393676 | RAB2B, member RAS oncogene family |
| CXCR6 | 0.06404743 | C-X-C motif chemokine receptor 6 |
| CACNG2 | 0.06415547 | calcium voltage-gated channel auxiliary subunit gamma 2 |
| HIBADH | 0.06416337 | 3-hydroxyisobutyrate dehydrogenase |
| FAM175A | 0.06422134 | family with sequence similarity 175 member A |
| CREB3L1 | 0.06428195 | cAMP responsive element binding protein 3 like 1 |
| FNIP1 | 0.06440843 | folliculin interacting protein 1 |
| PSTK | 0.06443742 | phosphoseryl-tRNA kinase |
| TACR2 | 0.06454809 | tachykinin receptor 2 |
| OLFM1 | 0.0645639 | olfactomedin 1 |
| KLF1 | 0.06465613 | Kruppel like factor 1 |
| VCPKMT | 0.06470883 | valosin containing protein lysine methyltransferase |
| TBX21 | 0.06481423 | T-box 21 |
| KCNMA1 | 0.0648722 | potassium calcium-activated channel subfamily M alpha 1 |
| NME8 | 0.06489855 | NME/NM23 family member 8 |
| WDR45 | 0.06495125 | WD repeat domain 45 |
| PDZD3 | 0.06510935 | PDZ domain containing 3 |
| KLF7 | 0.06523057 | Kruppel like factor 7 |
| KLF7 | 0.06523057 | Kruppel like factor 7 |
| SGCD | 0.06538076 | sarcoglycan delta |
| TSSK1B | 0.06547036 | testis specific serine kinase 1B |
| MPP2 | 0.06551515 | membrane palmitoylated protein 2 |
| CYP27B1 | 0.06567325 | cytochrome P450 family 27 subfamily B member 1 |
| LYPLAL1 | 0.06568906 | lysophospholipase like 1 |
| HCRTR2 | 0.06579183 | hypocretin receptor 2 |
| SUV39H2 | 0.06591568 | suppressor of variegation 3-9 homolog 2 |
| EPG5 | 0.06594466 | ectopic P-granules autophagy protein 5 homolog |
| EPG5 | 0.06594466 | ectopic P-granules autophagy protein 5 homolog |
| MBNL1 | 0.06595257 | muscleblind like splicing regulator 1 |
| NSMCE2 | 0.06599736 | NSE2/MMS21 homolog, SMC5-SMC6 complex SUMO ligase |
| COMMD10 | 0.06602108 | COMM domain containing 10 |
| PLEKHA3 | 0.06604216 | pleckstrin homology domain containing A3 |
| CALN1 | 0.0660527 | calneuron 1 |
| PCBD2 | 0.06618445 | pterin-4 alpha-carbinolamine dehydratase 2 |
| GTPBP2 | 0.06620817 | GTP binding protein 2 |
| MAP3K8 | 0.06628722 | mitogen-activated protein kinase kinase kinase 8 |
| RAB27A | 0.0663004 | RAB27A, member RAS oncogene family |
| AMPH | 0.06638208 | amphiphysin |
| CRHR1 | 0.06639789 | corticotropin releasing hormone receptor 1 |
| PPARGC1A | 0.0664137 | PPARG coactivator 1 alpha |
| ZMYM6 | 0.06642424 | zinc finger MYM-type containing 6 |
| TUBE1 | 0.06643742 | tubulin epsilon 1 |
| FAM134C | 0.06645586 | family with sequence similarity 134 member C |
| WWTR1 | 0.06654282 | WW domain containing transcription regulator 1 |
| GHR | 0.06657971 | growth hormone receptor |
| PRSS12 | 0.06658235 | protease, serine 12 |
| TNPO3 | 0.06659025 | transportin 3 |
| PRG4 | 0.06662187 | proteoglycan 4 |
| PRR16 | 0.06665349 | proline rich 16 |
| FXR2 | 0.0667668 | FMR1 autosomal homolog 2 |
| HDAC8 | 0.06696179 | histone deacetylase 8 |
| ZNF441 | 0.06696706 | zinc finger protein 441 |
| NMT2 | 0.06698551 | N-myristoyltransferase 2 |
| PRIMPOL | 0.06704348 | primase and DNA directed polymerase |
| WDR35 | 0.06704348 | WD repeat domain 35 |
| MINK1 | 0.06705665 | misshapen like kinase 1 |
| CEACAM21 | 0.06714361 | carcinoembryonic antigen related cell adhesion molecule 21 |
| ST6GALNAC5 | 0.06722793 | ST6 N-acetylgalactosaminide alpha-2,6-sialyltransferase 5 |
| LINC01349 | 0.06732806 | long intergenic non-protein coding RNA 1349 |
| LOC100419583 | 0.06740448 | ring finger protein 4 pseudogene |
| SRCIN1 | 0.06750198 | SRC kinase signaling inhibitor 1 |
| PDZRN3 | 0.06762582 | PDZ domain containing ring finger 3 |
| ARMC12 | 0.06768643 | armadillo repeat containing 12 |
| C17orf74 | 0.06773386 | chromosome 17 open reading frame 74 |
| TECTA | 0.06784453 | tectorin alpha |
| LYZL1 | 0.06789196 | lysozyme like 1 |
| TACC2 | 0.0679025 | transforming acidic coiled-coil containing protein 2 |
| LIMA1 | 0.06791831 | LIM domain and actin binding 1 |
| DNAJC18 | 0.06792095 | DnaJ heat shock protein family (Hsp40) member C18 |
| CACNB3 | 0.06796311 | calcium voltage-gated channel auxiliary subunit beta 3 |
| SLC3A2 | 0.06805797 | solute carrier family 3 member 2 |
| ANKRA2 | 0.06812121 | ankyrin repeat family A member 2 |
| ZNF624 | 0.06813439 | zinc finger protein 624 |
| ZNF624 | 0.06813439 | zinc finger protein 624 |
| KLC2 | 0.06814229 | kinesin light chain 2 |
| DKK3 | 0.06814493 | dickkopf WNT signaling pathway inhibitor 3 |
| CXCL3 | 0.0681581 | C-X-C motif chemokine ligand 3 |
| ABCA11P | 0.06826087 | ATP binding cassette subfamily A member 11, pseudogene |
| TELO2 | 0.06826614 | telomere maintenance 2 |
| TRIM6 | 0.06836364 | tripartite motif containing 6 |
| TMTC1 | 0.06837154 | transmembrane and tetratricopeptide repeat containing 1 |
| LOC105376351 | 0.06855863 | uncharacterized LOC105376351 |
| FBXL3 | 0.06857181 | F-box and leucine rich repeat protein 3 |
| PHLPP2 | 0.06861924 | PH domain and leucine rich repeat protein phosphatase 2 |
| ZNHIT2 | 0.06865086 | zinc finger HIT-type containing 2 |
| B3GNT9 | 0.06866403 | UDP-GlcNAc:betaGal beta-1,3-N-acetylglucosaminyltransferase 9 |
| WRAP53 | 0.06869829 | WD repeat containing antisense to TP53 |
| STON1-GTF2A1L | 0.06871146 | STON1-GTF2A1L readthrough |
| STON1-GTF2A1L | 0.06871146 | STON1-GTF2A1L readthrough |
| PLK2 | 0.06878788 | polo like kinase 2 |
| LOC101928659 | 0.06879842 | uncharacterized LOC101928659 |
| CLDN17 | 0.06893017 | claudin 17 |
| TIMM13 | 0.06897233 | translocase of inner mitochondrial membrane 13 |
| CRCP | 0.06911989 | CGRP receptor component |
| ALDH1A3 | 0.06916996 | aldehyde dehydrogenase 1 family member A3 |
| CHSY3 | 0.06918841 | chondroitin sulfate synthase 3 |
| SUCLA2 | 0.0693307 | succinate-CoA ligase ADP-forming beta subunit |
| WDR5B | 0.0693834 | WD repeat domain 5B |
| PDPN | 0.06939657 | podoplanin |
| RALB | 0.06948617 | RALB Ras like proto-oncogene B |
| GFRA1 | 0.06952569 | GDNF family receptor alpha 1 |
| TRAPPC9 | 0.06955731 | trafficking protein particle complex 9 |
| STEAP3 | 0.06958103 | STEAP3 metalloreductase |
| MTMR9 | 0.06959684 | myotubularin related protein 9 |
| BHLHE40 | 0.06968643 | basic helix-loop-helix family member e40 |
| UMAD1 | 0.06978393 | UBAP1-MVB12-associated (UMA) domain containing 1 |
| PIP4K2A | 0.06983136 | phosphatidylinositol-5-phosphate 4-kinase type 2 alpha |
| XRN1 | 0.0698419 | 5'-3' exoribonuclease 1 |
| FHL3 | 0.06993412 | four and a half LIM domains 3 |
| PRKCQ | 0.07021607 | protein kinase C theta |
| TBL3 | 0.07022134 | transducin beta like 3 |
| ZNF493 | 0.07022134 | zinc finger protein 493 |
| AAGAB | 0.07024769 | alpha- and gamma-adaptin binding protein |
| ZDHHC5 | 0.07031884 | zinc finger DHHC-type containing 5 |
| SFR1 | 0.07032411 | SWI5 dependent homologous recombination repair protein 1 |
| CDK2AP2 | 0.07049012 | cyclin dependent kinase 2 associated protein 2 |
| SCYL1 | 0.07062451 | SCY1 like pseudokinase 1 |
| GBE1 | 0.07062714 | glucan (1,4-alpha-), branching enzyme 1 |
| TNFSF12 | 0.07065349 | tumor necrosis factor superfamily member 12 |
| GLT8D2 | 0.07080369 | glycosyltransferase 8 domain containing 2 |
| C8orf34 | 0.07090119 | chromosome 8 open reading frame 34 |
| LOC101928714 | 0.07104348 | uncharacterized LOC101928714 |
| E4F1 | 0.07105665 | E4F transcription factor 1 |
| ZBTB7B | 0.07115152 | zinc finger and BTB domain containing 7B |
| FAU | 0.07115679 | FAU, ubiquitin like and ribosomal protein S30 fusion |
| RBMS3 | 0.07123584 | RNA binding motif single stranded interacting protein 3 |
| ZFPM2 | 0.07124111 | zinc finger protein, FOG family member 2 |
| GPRC5B | 0.07139657 | G protein-coupled receptor class C group 5 member B |
| RGS18 | 0.07143874 | regulator of G-protein signaling 18 |
| SPOP | 0.07145191 | speckle type BTB/POZ protein |
| ARMT1 | 0.07145982 | acidic residue methyltransferase 1 |
| PIK3C2B | 0.07150461 | phosphatidylinositol-4-phosphate 3-kinase catalytic subunit type 2 beta |
| LRRC46 | 0.07150461 | leucine rich repeat containing 46 |
| AP5M1 | 0.07161528 | adaptor related protein complex 5 mu 1 subunit |
| FBXL22 | 0.07162055 | F-box and leucine rich repeat protein 22 |
| ALG12 | 0.07186825 | ALG12, alpha-1,6-mannosyltransferase |
| FAM109B | 0.07192358 | family with sequence similarity 109 member B |
| ZCCHC2 | 0.07195257 | zinc finger CCHC-type containing 2 |
| RHEB | 0.07200791 | Ras homolog enriched in brain |
| OR2A1 | 0.07205534 | olfactory receptor family 2 subfamily A member 1 |
| DOK6 | 0.07206061 | docking protein 6 |
| DOK6 | 0.07206061 | docking protein 6 |
| MCOLN3 | 0.07214493 | mucolipin 3 |
| SPAG9 | 0.07225823 | sperm associated antigen 9 |
| TPPP | 0.07238472 | tubulin polymerization promoting protein |
| ESYT2 | 0.07239789 | extended synaptotagmin 2 |
| MRPS18A | 0.07254809 | mitochondrial ribosomal protein S18A |
| HEG1 | 0.07258762 | heart development protein with EGF like domains 1 |
| SYTL3 | 0.0726166 | synaptotagmin like 3 |
| SLC25A4 | 0.07274572 | solute carrier family 25 member 4 |
| AACS | 0.07277734 | acetoacetyl-CoA synthetase |
| NDN | 0.07282213 | necdin, MAGE family member |
| CD200 | 0.07299078 | CD200 molecule |
| ZBTB4 | 0.07300132 | zinc finger and BTB domain containing 4 |
| PLEK | 0.07304084 | pleckstrin |
| LBX2 | 0.07327009 | ladybird homeobox 2 |
| FOLR2 | 0.0732859 | folate receptor beta |
| APOOL | 0.07332279 | apolipoprotein O like |
| KLHL35 | 0.07337022 | kelch like family member 35 |
| EDIL3 | 0.07338603 | EGF like repeats and discoidin domains 3 |
| EEF1AKMT1 | 0.07338603 | eukaryotic translation elongation factor 1 alpha lysine methyltransferase 1 |
| RAMP1 | 0.07342029 | receptor activity modifying protein 1 |
| PLS3 | 0.07342819 | plastin 3 |
| B2M | 0.07351779 | beta-2-microglobulin |
| FCER1A | 0.07352042 | Fc fragment of IgE receptor Ia |
| CGRRF1 | 0.07352833 | cell growth regulator with ring finger domain 1 |
| FAM102A | 0.07360738 | family with sequence similarity 102 member A |
| ZSCAN25 | 0.07372332 | zinc finger and SCAN domain containing 25 |
| MAR9 | 0.0738946 | membrane associated ring-CH-type finger 9 |
| FBXL7 | 0.0739025 | F-box and leucine rich repeat protein 7 |
| TOX | 0.07401054 | thymocyte selection associated high mobility group box |
| ARSI | 0.07404216 | arylsulfatase family member I |
| C2CD3 | 0.07407378 | C2 calcium dependent domain containing 3 |
| STAT4 | 0.07410013 | signal transducer and activator of transcription 4 |
| PCSK1N | 0.07410277 | proprotein convertase subtilisin/kexin type 1 inhibitor |
| FZR1 | 0.07413966 | fizzy/cell division cycle 20 related 1 |
| ARPC3 | 0.07424242 | actin related protein 2/3 complex subunit 3 |
| FGF7 | 0.07426877 | fibroblast growth factor 7 |
| FBXL5 | 0.07428986 | F-box and leucine rich repeat protein 5 |
| TNFRSF10C | 0.07442161 | TNF receptor superfamily member 10c |
| SNORD15B | 0.07443742 | small nucleolar RNA, C/D box 15B |
| CREB3L4 | 0.07476153 | cAMP responsive element binding protein 3 like 4 |
| TMEM132C | 0.07477207 | transmembrane protein 132C |
| RAD21 | 0.07484585 | RAD21 cohesin complex component |
| DCP1B | 0.07495652 | decapping mRNA 1B |
| TMEM236 | 0.0749776 | transmembrane protein 236 |
| PML | 0.07505929 | promyelocytic leukemia |
| PDS5B | 0.07510145 | PDS5 cohesin associated factor B |
| SERPINE2 | 0.07531225 | serpin family E member 2 |
| CYP2U1 | 0.07537022 | cytochrome P450 family 2 subfamily U member 1 |
| SEL1L3 | 0.07537022 | SEL1L family member 3 |
| DECR2 | 0.07540184 | 2,4-dienoyl-CoA reductase 2, peroxisomal |
| CDK18 | 0.07543874 | cyclin dependent kinase 18 |
| HTRA4 | 0.0754809 | HtrA serine peptidase 4 |
| LSM2 | 0.07568643 | LSM2 homolog, U6 small nuclear RNA and mRNA degradation associated |
| SLC25A11 | 0.07575231 | solute carrier family 25 member 11 |
| MPDU1 | 0.07577339 | mannose-P-dolichol utilization defect 1 |
| HPS3 | 0.07590514 | HPS3, biogenesis of lysosomal organelles complex 2 subunit 1 |
| MGLL | 0.07593939 | monoglyceride lipase |
| IDH1 | 0.07597628 | isocitrate dehydrogenase (NADP(+)) 1, cytosolic |
| RPL26 | 0.07607905 | ribosomal protein L26 |
| CHRNB3 | 0.07617391 | cholinergic receptor nicotinic beta 3 subunit |
| ANO4 | 0.07621344 | anoctamin 4 |
| COL12A1 | 0.07635046 | collagen type XII alpha 1 chain |
| MYOC | 0.07635837 | myocilin |
| CLECL1 | 0.07638735 | C-type lectin like 1 |
| FBXW11 | 0.07646904 | F-box and WD repeat domain containing 11 |
| TERF2IP | 0.07654809 | TERF2 interacting protein |
| MAPRE2 | 0.07667984 | microtubule associated protein RP/EB family member 2 |
| COX7C | 0.07675362 | cytochrome c oxidase subunit 7C |
| KSR2 | 0.07677207 | kinase suppressor of ras 2 |
| SNORD33 | 0.07684058 | small nucleolar RNA, C/D box 33 |
| ABCB8 | 0.07685375 | ATP binding cassette subfamily B member 8 |
| EPM2A | 0.07695389 | epilepsy, progressive myoclonus type 2A, Lafora disease (laforin) |
| PAX9 | 0.07708564 | paired box 9 |
| CNP | 0.0771357 | 2',3'-cyclic nucleotide 3' phosphodiesterase |
| STK17B | 0.07719631 | serine/threonine kinase 17b |
| TARSL2 | 0.07738867 | threonyl-tRNA synthetase like 2 |
| STAT5A | 0.07743874 | signal transducer and activator of transcription 5A |
| PKD1 | 0.07750725 | polycystin 1, transient receptor potential channel interacting |
| FABP2 | 0.07750988 | fatty acid binding protein 2 |
| USPL1 | 0.07752569 | ubiquitin specific peptidase like 1 |
| PRPH | 0.07756258 | peripherin |
| ADGRF3 | 0.07761528 | adhesion G protein-coupled receptor F3 |
| SYNGR1 | 0.07780237 | synaptogyrin 1 |
| KANK2 | 0.07800527 | KN motif and ankyrin repeat domains 2 |
| PDE4B | 0.07812121 | phosphodiesterase 4B |
| PIK3CG | 0.07826877 | phosphatidylinositol-4,5-bisphosphate 3-kinase catalytic subunit gamma |
| PIK3CG | 0.07826877 | phosphatidylinositol-4,5-bisphosphate 3-kinase catalytic subunit gamma |
| C5orf30 | 0.07827931 | chromosome 5 open reading frame 30 |
| SLC9A9 | 0.07829513 | solute carrier family 9 member A9 |
| ZNF799 | 0.07831094 | zinc finger protein 799 |
| TSTA3 | 0.07833202 | tissue specific transplantation antigen P35B |
| CUL3 | 0.0784664 | cullin 3 |
| PLPPR5 | 0.07847431 | phospholipid phosphatase related 5 |
| UQCRFS1 | 0.07851383 | ubiquinol-cytochrome c reductase, Rieske iron-sulfur polypeptide 1 |
| HINFP | 0.07857708 | histone H4 transcription factor |
| ZNF784 | 0.07881686 | zinc finger protein 784 |
| SHOX2 | 0.07889592 | short stature homeobox 2 |
| FAM218A | 0.07899078 | family with sequence similarity 218 member A |
| HECTD1 | 0.07901449 | HECT domain E3 ubiquitin protein ligase 1 |
| PMP2 | 0.07915679 | peripheral myelin protein 2 |
| ONECUT2 | 0.07916733 | one cut homeobox 2 |
| ONECUT2 | 0.07916733 | one cut homeobox 2 |
| ONECUT2 | 0.07916733 | one cut homeobox 2 |
| CFHR3 | 0.07931489 | complement factor H related 3 |
| C6orf52 | 0.07951252 | chromosome 6 open reading frame 52 |
| TCEAL3 | 0.07954414 | transcription elongation factor A like 3 |
| YPEL5 | 0.07957839 | yippee like 5 |
| BPNT1 | 0.07962582 | 3'(2'), 5'-bisphosphate nucleotidase 1 |
| CDO1 | 0.07967062 | cysteine dioxygenase type 1 |
| KIAA1551 | 0.07968643 | KIAA1551 |
| QTRT1 | 0.07968643 | queuine tRNA-ribosyltransferase catalytic subunit 1 |
| GPR68 | 0.0796996 | G protein-coupled receptor 68 |
| FAM50B | 0.07970224 | family with sequence similarity 50 member B |
| TMEM223 | 0.07979974 | transmembrane protein 223 |
| RGS7BP | 0.0798498 | regulator of G-protein signaling 7 binding protein |
| IPO7 | 0.0798498 | importin 7 |
| MAPK1IP1L | 0.07992358 | mitogen-activated protein kinase 1 interacting protein 1 like |
| PRKAR1A | 0.08012121 | protein kinase cAMP-dependent type I regulatory subunit alpha |
| LARP7 | 0.08016337 | La ribonucleoprotein domain family member 7 |
| SLAMF8 | 0.08023188 | SLAM family member 8 |
| NEGR1 | 0.08024242 | neuronal growth regulator 1 |
| MIR181A2HG | 0.08032411 | MIR181A2 host gene |
| FBXW7 | 0.08032938 | F-box and WD repeat domain containing 7 |
| HIST2H2AC | 0.08033992 | histone cluster 2, H2ac |
| LOC100128239 | 0.08034783 | uncharacterized LOC100128239 |
| FAM163B | 0.08037418 | family with sequence similarity 163 member B |
| PRKAB2 | 0.08042424 | protein kinase AMP-activated non-catalytic subunit beta 2 |
| LIG4 | 0.08045059 | DNA ligase 4 |
| AFF3 | 0.08053755 | AF4/FMR2 family member 3 |
| KCTD11 | 0.08072464 | potassium channel tetramerization domain containing 11 |
| WDR11 | 0.08076153 | WD repeat domain 11 |
| ZNF202 | 0.08085903 | zinc finger protein 202 |
| ISCA1 | 0.08096706 | iron-sulfur cluster assembly 1 |
| TMEM125 | 0.08098287 | transmembrane protein 125 |
| CTSV | 0.08100659 | cathepsin V |
| IL1RAPL1 | 0.08103294 | interleukin 1 receptor accessory protein like 1 |
| COL24A1 | 0.08104611 | collagen type XXIV alpha 1 chain |
| C7orf50 | 0.08108827 | chromosome 7 open reading frame 50 |
| RARB | 0.08114361 | retinoic acid receptor beta |
| C2CD4B | 0.08116733 | C2 calcium dependent domain containing 4B |
| NFKBIL1 | 0.08119631 | NFKB inhibitor like 1 |
| SLC2A3 | 0.08132279 | solute carrier family 2 member 3 |
| SORCS2 | 0.08133333 | sortilin related VPS10 domain containing receptor 2 |
| HIST1H1D | 0.08136759 | histone cluster 1, H1d |
| HMBS | 0.08139657 | hydroxymethylbilane synthase |
| EIF3H | 0.08169697 | eukaryotic translation initiation factor 3 subunit H |
| FANK1 | 0.08175758 | fibronectin type III and ankyrin repeat domains 1 |
| SLC6A16 | 0.08181555 | solute carrier family 6 member 16 |
| RAB32 | 0.08197892 | RAB32, member RAS oncogene family |
| C9orf163 | 0.08199209 | chromosome 9 open reading frame 163 |
| RHPN1 | 0.08199473 | rhophilin Rho GTPase binding protein 1 |
| KDM3B | 0.08201054 | lysine demethylase 3B |
| TVP23C | 0.08205007 | trans-golgi network vesicle protein 23 homolog C (S. cerevisiae) |
| MT1M | 0.08216074 | metallothionein 1M |
| LINC00354 | 0.08217918 | long intergenic non-protein coding RNA 354 |
| IL6ST | 0.08220817 | interleukin 6 signal transducer |
| IL6ST | 0.08220817 | interleukin 6 signal transducer |
| SMAD5-AS1 | 0.08223715 | SMAD5 antisense RNA 1 |
| OR4C15 | 0.08247958 | olfactory receptor family 4 subfamily C member 15 |
| BCHE | 0.08250593 | butyrylcholinesterase |
| CD86 | 0.08252964 | CD86 molecule |
| CD33 | 0.08253491 | CD33 molecule |
| STX18 | 0.08259552 | syntaxin 18 |
| ZNF821 | 0.0826693 | zinc finger protein 821 |
| RD3 | 0.08286693 | retinal degeneration 3 |
| AKAP11 | 0.08295916 | A-kinase anchoring protein 11 |
| ODC1 | 0.08303557 | ornithine decarboxylase 1 |
| PRDM9 | 0.08308564 | PR/SET domain 9 |
| TSPAN7 | 0.08314361 | tetraspanin 7 |
| LOC100506122 | 0.08327009 | uncharacterized LOC100506122 |
| VN1R5 | 0.08332016 | vomeronasal 1 receptor 5 (gene/pseudogene) |
| VIT | 0.08337022 | vitrin |
| SMCP | 0.08339657 | sperm mitochondria associated cysteine rich protein |
| KLHDC2 | 0.08344928 | kelch domain containing 2 |
| FNDC3A | 0.08345718 | fibronectin type III domain containing 3A |
| LOC101927166 | 0.08346245 | uncharacterized LOC101927166 |
| SHH | 0.08373913 | sonic hedgehog |
| CATIP | 0.08375231 | ciliogenesis associated TTC17 interacting protein |
| COL19A1 | 0.08398946 | collagen type XIX alpha 1 chain |
| PRR3 | 0.08399209 | proline rich 3 |
| C16orf52 | 0.08401581 | chromosome 16 open reading frame 52 |
| ENPP6 | 0.08403953 | ectonucleotide pyrophosphatase/phosphodiesterase 6 |
| STK16 | 0.08405534 | serine/threonine kinase 16 |
| ST7-AS1 | 0.08406851 | ST7 antisense RNA 1 |
| WWC2-AS1 | 0.0842029 | WWC2 antisense RNA 1 |
| SAYSD1 | 0.08423452 | SAYSVFN motif domain containing 1 |
| CLMP | 0.08449275 | CXADR like membrane protein |
| SCARB1 | 0.08461924 | scavenger receptor class B member 1 |
| PLCB4 | 0.08495652 | phospholipase C beta 4 |
| LAYN | 0.08498814 | layilin |
| CNTN3 | 0.08507773 | contactin 3 |
| ATP8B4 | 0.08515679 | ATPase phospholipid transporting 8B4 (putative) |
| MORC2 | 0.08516733 | MORC family CW-type zinc finger 2 |
| KITLG | 0.0851805 | KIT ligand |
| THTPA | 0.08524901 | thiamine triphosphatase |
| LOC100506675 | 0.08525692 | uncharacterized LOC100506675 |
| SACS | 0.08531225 | sacsin molecular chaperone |
| RAB9A | 0.08531752 | RAB9A, member RAS oncogene family |
| RNH1 | 0.08532016 | ribonuclease/angiogenin inhibitor 1 |
| TNFAIP8L3 | 0.08556258 | TNF alpha induced protein 8 like 3 |
| DARS | 0.08567062 | aspartyl-tRNA synthetase |
| POP1 | 0.08568116 | POP1 homolog, ribonuclease P/MRP subunit |
| MYOT | 0.0863083 | myotilin |
| CD209 | 0.08643478 | CD209 molecule |
| PDLIM3 | 0.08644796 | PDZ and LIM domain 3 |
| CDH4 | 0.08651383 | cadherin 4 |
| NANOG | 0.08675889 | Nanog homeobox |
| PORCN | 0.08684321 | porcupine homolog (Drosophila) |
| VWA7 | 0.08711462 | von Willebrand factor A domain containing 7 |
| DIRC3 | 0.08719631 | disrupted in renal carcinoma 3 |
| PTCH1 | 0.08726219 | patched 1 |
| STX1A | 0.08739394 | syntaxin 1A |
| SEZ6L2 | 0.08772069 | seizure related 6 homolog like 2 |
| GNB5 | 0.08772596 | G protein subunit beta 5 |
| GANAB | 0.08777866 | glucosidase II alpha subunit |
| RGS22 | 0.08779447 | regulator of G-protein signaling 22 |
| ZNF565 | 0.08783663 | zinc finger protein 565 |
| OTUB2 | 0.08784717 | OTU deubiquitinase, ubiquitin aldehyde binding 2 |
| PTGER2 | 0.08796047 | prostaglandin E receptor 2 |
| LINC01210 | 0.08802108 | long intergenic non-protein coding RNA 1210 |
| IL36RN | 0.08804216 | interleukin 36 receptor antagonist |
| BAG4 | 0.08811331 | BCL2 associated athanogene 4 |
| PDLIM5 | 0.08822134 | PDZ and LIM domain 5 |
| DCLK2 | 0.08825296 | doublecortin like kinase 2 |
| CNR1 | 0.08835573 | cannabinoid receptor 1 |
| ZBED6CL | 0.08835837 | ZBED6 C-terminal like |
| LHB | 0.08837681 | luteinizing hormone beta polypeptide |
| ANKS1B | 0.08841897 | ankyrin repeat and sterile alpha motif domain containing 1B |
| C10orf95 | 0.08845059 | chromosome 10 open reading frame 95 |
| SYNC | 0.0884585 | syncoilin, intermediate filament protein |
| FCF1 | 0.08849275 | FCF1 rRNA-processing protein |
| MTHFR | 0.08853228 | methylenetetrahydrofolate reductase (NAD(P)H) |
| LRRC41 | 0.0885639 | leucine rich repeat containing 41 |
| TMEM131 | 0.08871146 | transmembrane protein 131 |
| SNORA66 | 0.088722 | small nucleolar RNA, H/ACA box 66 |
| ARFGAP3 | 0.08874835 | ADP ribosylation factor GTPase activating protein 3 |
| P2RX1 | 0.08884848 | purinergic receptor P2X 1 |
| SDHD | 0.08885903 | succinate dehydrogenase complex subunit D |
| KIAA0430 | 0.08898551 | KIAA0430 |
| SLC35E4 | 0.08909091 | solute carrier family 35 member E4 |
| ARMC8 | 0.08910672 | armadillo repeat containing 8 |
| LOC105371907 | 0.08910672 | uncharacterized LOC105371907 |
| PKD1L2 | 0.08936495 | polycystin 1 like 2 (gene/pseudogene) |
| RCOR2 | 0.08946772 | REST corepressor 2 |
| FAS | 0.08957839 | Fas cell surface death receptor |
| MFSD9 | 0.089639 | major facilitator superfamily domain containing 9 |
| TMCC3 | 0.08965217 | transmembrane and coiled-coil domain family 3 |
| TNS4 | 0.08974177 | tensin 4 |
| ZNF143 | 0.0898946 | zinc finger protein 143 |
| L3HYPDH | 0.08996838 | trans-L-3-hydroxyproline dehydratase |
| SCARB2 | 0.09002899 | scavenger receptor class B member 2 |
| ATP10A | 0.09030567 | ATPase phospholipid transporting 10A (putative) |
| NUP62CL | 0.09037681 | nucleoporin 62 C-terminal like |
| TBC1D19 | 0.09042951 | TBC1 domain family member 19 |
| ATF6B | 0.09044005 | activating transcription factor 6 beta |
| ELL2 | 0.09052701 | elongation factor for RNA polymerase II 2 |
| MOCS2 | 0.09080632 | molybdenum cofactor synthesis 2 |
| DNAJC25-GNG10 | 0.09093808 | DNAJC25-GNG10 readthrough |
| GZMA | 0.09098814 | granzyme A |
| PPFIA2 | 0.09104348 | PTPRF interacting protein alpha 2 |
| FAM114A1 | 0.09116733 | family with sequence similarity 114 member A1 |
| SRP19 | 0.09116733 | signal recognition particle 19 |
| MAPK7 | 0.09122266 | mitogen-activated protein kinase 7 |
| ZSCAN9 | 0.09142029 | zinc finger and SCAN domain containing 9 |
| TMPRSS9 | 0.09152833 | transmembrane protease, serine 9 |
| HCG14 | 0.0915415 | HLA complex group 14 (non-protein coding) |
| HOXA11 | 0.09160211 | homeobox A11 |
| PLA2G5 | 0.09177866 | phospholipase A2 group V |
| FBXO30 | 0.09178393 | F-box protein 30 |
| PKIG | 0.09192885 | protein kinase (cAMP-dependent, catalytic) inhibitor gamma |
| ZNF408 | 0.09197892 | zinc finger protein 408 |
| DUSP26 | 0.09201054 | dual specificity phosphatase 26 (putative) |
| CDH19 | 0.09205007 | cadherin 19 |
| RBKS | 0.0920975 | ribokinase |
| C5orf42 | 0.09211858 | chromosome 5 open reading frame 42 |
| ADRB2 | 0.09226877 | adrenoceptor beta 2 |
| SMIM10 | 0.09227668 | small integral membrane protein 10 |
| PPP3CC | 0.09227668 | protein phosphatase 3 catalytic subunit gamma |
| NAP1L3 | 0.09231094 | nucleosome assembly protein 1 like 3 |
| NR2C2 | 0.09236627 | nuclear receptor subfamily 2 group C member 2 |
| SSNA1 | 0.09238999 | SS nuclear autoantigen 1 |
| RANBP3L | 0.0924137 | RAN binding protein 3 like |
| SOS2 | 0.0926693 | SOS Ras/Rho guanine nucleotide exchange factor 2 |
| UXS1 | 0.09267721 | UDP-glucuronate decarboxylase 1 |
| GAS1 | 0.09268248 | growth arrest specific 1 |
| EDAR | 0.09275889 | ectodysplasin A receptor |
| OCIAD1 | 0.0928722 | OCIA domain containing 1 |
| DIRAS3 | 0.09308827 | DIRAS family GTPase 3 |
| SKIV2L2 | 0.09313307 | Ski2 like RNA helicase 2 |
| HSPB7 | 0.0931726 | heat shock protein family B (small) member 7 |
| PHYHIP | 0.093278 | phytanoyl-CoA 2-hydroxylase interacting protein |
| RND1 | 0.09330171 | Rho family GTPase 1 |
| CD58 | 0.0934888 | CD58 molecule |
| RHBDF2 | 0.09356785 | rhomboid 5 homolog 2 |
| TNPO1 | 0.09361792 | transportin 1 |
| TNPO1 | 0.09361792 | transportin 1 |
| MX2 | 0.09376021 | MX dynamin like GTPase 2 |
| ZCCHC8 | 0.09382345 | zinc finger CCHC-type containing 8 |
| KLF10 | 0.09394203 | Kruppel like factor 10 |
| NEIL3 | 0.09413175 | nei like DNA glycosylase 3 |
| STON1 | 0.09417918 | stonin 1 |
| ITIH5 | 0.09428458 | inter-alpha-trypsin inhibitor heavy chain family member 5 |
| SLC16A7 | 0.09437945 | solute carrier family 16 member 7 |
| SLC16A7 | 0.09437945 | solute carrier family 16 member 7 |
| CD302 | 0.09440843 | CD302 molecule |
| GLIS3 | 0.09451647 | GLIS family zinc finger 3 |
| TRPC3 | 0.0946166 | transient receptor potential cation channel subfamily C member 3 |
| ZYX | 0.09462187 | zyxin |
| HERPUD2 | 0.09463768 | HERPUD family member 2 |
| BSDC1 | 0.09469565 | BSD domain containing 1 |
| CTSO | 0.09474835 | cathepsin O |
| USP18 | 0.09480105 | ubiquitin specific peptidase 18 |
| SKA2 | 0.09484585 | spindle and kinetochore associated complex subunit 2 |
| C11orf80 | 0.09503294 | chromosome 11 open reading frame 80 |
| CYP46A1 | 0.0952332 | cytochrome P450 family 46 subfamily A member 1 |
| RUNDC3B | 0.09527009 | RUN domain containing 3B |
| KBTBD4 | 0.09550988 | kelch repeat and BTB domain containing 4 |
| WT1 | 0.09567325 | Wilms tumor 1 |
| C9orf16 | 0.09568643 | chromosome 9 open reading frame 16 |
| INVS | 0.09576021 | inversin |
| IL18RAP | 0.09577602 | interleukin 18 receptor accessory protein |
| LARP4 | 0.0957971 | La ribonucleoprotein domain family member 4 |
| LMO1 | 0.09603426 | LIM domain only 1 |
| FAM117A | 0.09606324 | family with sequence similarity 117 member A |
| PALLD | 0.09643215 | palladin, cytoskeletal associated protein |
| C16orf86 | 0.09648485 | chromosome 16 open reading frame 86 |
| MDFI | 0.09662978 | MyoD family inhibitor |
| GPBP1 | 0.09664559 | GC-rich promoter binding protein 1 |
| POLR3GL | 0.09686957 | RNA polymerase III subunit G like |
| OARD1 | 0.09688274 | O-acyl-ADP-ribose deacylase 1 |
| GREM2 | 0.09690646 | gremlin 2, DAN family BMP antagonist |
| ZEB1 | 0.09691436 | zinc finger E-box binding homeobox 1 |
| ACOT2 | 0.09694598 | acyl-CoA thioesterase 2 |
| BHMT2 | 0.0969697 | betaine--homocysteine S-methyltransferase 2 |
| IFI27L1 | 0.09700395 | interferon alpha inducible protein 27 like 1 |
| PTGFR | 0.09717787 | prostaglandin F receptor |
| DOCK7 | 0.09728854 | dedicator of cytokinesis 7 |
| ZNF227 | 0.09730698 | zinc finger protein 227 |
| MBNL1-AS1///MBNL1 | 0.0973386 | MBNL1 antisense RNA 1///muscleblind like splicing regulator 1 |
| PFKL | 0.09740184 | phosphofructokinase, liver type |
| RBM12B | 0.09765217 | RNA binding motif protein 12B |
| DCTN3 | 0.09766535 | dynactin subunit 3 |
| RAP1GDS1 | 0.09767062 | Rap1 GTPase-GDP dissociation stimulator 1 |
| ZNF394 | 0.09775494 | zinc finger protein 394 |
| OSR2 | 0.09788933 | odd-skipped related transciption factor 2 |
| AGTR1 | 0.09814229 | angiotensin II receptor type 1 |
| C9orf78 | 0.09817128 | chromosome 9 open reading frame 78 |
| UFM1 | 0.09818182 | ubiquitin fold modifier 1 |
| SCRN2 | 0.09827141 | secernin 2 |
| USP35 | 0.0983004 | ubiquitin specific peptidase 35 |
| CFLAR | 0.09831357 | CASP8 and FADD like apoptosis regulator |
| LURAP1 | 0.09835837 | leucine rich adaptor protein 1 |
| SLC47A1 | 0.0985639 | solute carrier family 47 member 1 |
| GPR171 | 0.09865086 | G protein-coupled receptor 171 |
| FAM168B | 0.09865613 | family with sequence similarity 168 member B |
| HIST1H3E | 0.09867721 | histone cluster 1, H3e |
| SEC62 | 0.09903294 | SEC62 homolog, preprotein translocation factor |
| KCNA5 | 0.09917523 | potassium voltage-gated channel subfamily A member 5 |
| PLPPR4 | 0.09927273 | phospholipid phosphatase related 4 |
| SEC22A | 0.09946772 | SEC22 homolog A, vesicle trafficking protein |
| ADGRB3 | 0.09958366 | adhesion G protein-coupled receptor B3 |
| ACSL3 | 0.09970224 | acyl-CoA synthetase long-chain family member 3 |
| STARD3 | 0.09986298 | StAR related lipid transfer domain containing 3 |
| OPA1 | 0.09997101 | OPA1, mitochondrial dynamin like GTPase |
| LOC100505923 | 0.10003953 | uncharacterized LOC100505923 |
| HPD | 0.10004743 | 4-hydroxyphenylpyruvate dioxygenase |
| LINC00382 | 0.1000527 | long intergenic non-protein coding RNA 382 |
| UBXN10 | 0.10012648 | UBX domain protein 10 |
| BBS1 | 0.10013702 | Bardet-Biedl syndrome 1 |
| ALDH6A1 | 0.10015547 | aldehyde dehydrogenase 6 family member A1 |
| SERPINI1 | 0.10019499 | serpin family I member 1 |
| RPS6 | 0.10020026 | ribosomal protein S6 |
| TMOD1 | 0.10022925 | tropomodulin 1 |
| RPS25 | 0.1003083 | ribosomal protein S25 |
| CNTROB | 0.10032148 | centrobin, centriole duplication and spindle assembly protein |
| HIKESHI | 0.100361 | Hikeshi, heat shock protein nuclear import factor |
| WDR47 | 0.10040316 | WD repeat domain 47 |
| CSRP1 | 0.10075889 | cysteine and glycine rich protein 1 |
| TMEM11 | 0.10085639 | transmembrane protein 11 |
| PDE6G | 0.10119104 | phosphodiesterase 6G |
| LOC100507073 | 0.10120422 | uncharacterized LOC100507073 |
| TFEB | 0.1012253 | transcription factor EB |
| NRAV | 0.10123057 | negative regulator of antiviral response (non-protein coding) |
| HIST1H2BN | 0.10135705 | histone cluster 1, H2bn |
| CAMK2A | 0.10135968 | calcium/calmodulin dependent protein kinase II alpha |
| TMEM268 | 0.10136759 | transmembrane protein 268 |
| TMEM14C | 0.1013913 | transmembrane protein 14C |
| FA2H | 0.10144664 | fatty acid 2-hydroxylase |
| GATA5 | 0.10145455 | GATA binding protein 5 |
| PLN | 0.10146772 | phospholamban |
| ZFAND5 | 0.10161001 | zinc finger AN1-type containing 5 |
| NRAS | 0.10168643 | neuroblastoma RAS viral oncogene homolog |
| SPG20 | 0.10173913 | spastic paraplegia 20 (Troyer syndrome) |
| SPTAN1 | 0.10198946 | spectrin alpha, non-erythrocytic 1 |
| CLUAP1 | 0.10226614 | clusterin associated protein 1 |
| WDR26 | 0.10227404 | WD repeat domain 26 |
| HLF | 0.10228986 | HLF, PAR bZIP transcription factor |
| EYA4 | 0.10240843 | EYA transcriptional coactivator and phosphatase 4 |
| RNF38 | 0.10249802 | ring finger protein 38 |
| LOC105274304 | 0.10265086 | uncharacterized LOC105274304 |
| MXRA7 | 0.10269829 | matrix remodeling associated 7 |
| SEC23IP | 0.10271146 | SEC23 interacting protein |
| RYR2 | 0.10279842 | ryanodine receptor 2 |
| CA11 | 0.10284848 | carbonic anhydrase 11 |
| OSBP2 | 0.1028643 | oxysterol binding protein 2 |
| TRADD | 0.10296443 | TNFRSF1A associated via death domain |
| HLA-DMB | 0.10298814 | major histocompatibility complex, class II, DM beta |
| BOD1L1 | 0.10344137 | biorientation of chromosomes in cell division 1 like 1 |
| FAM76B | 0.1034888 | family with sequence similarity 76 member B |
| FAXDC2 | 0.1035336 | fatty acid hydroxylase domain containing 2 |
| PROS1 | 0.10362055 | protein S (alpha) |
| FSHB | 0.10362582 | follicle stimulating hormone beta subunit |
| PPP1R26 | 0.10366798 | protein phosphatase 1 regulatory subunit 26 |
| SLC1A2 | 0.1037444 | solute carrier family 1 member 2 |
| SLC25A12 | 0.10386561 | solute carrier family 25 member 12 |
| PSMD13 | 0.10389723 | proteasome 26S subunit, non-ATPase 13 |
| SMIM3 | 0.10400527 | small integral membrane protein 3 |
| MAR7 | 0.10417391 | membrane associated ring-CH-type finger 7 |
| DCTN6 | 0.10439526 | dynactin subunit 6 |
| RAD54B | 0.10447431 | RAD54 homolog B (S. cerevisiae) |
| HMGXB4 | 0.1045112 | HMG-box containing 4 |
| KCNN2 | 0.10457971 | potassium calcium-activated channel subfamily N member 2 |
| NDFIP1 | 0.10460343 | Nedd4 family interacting protein 1 |
| LOC105369340 | 0.10463768 | uncharacterized LOC105369340 |
| CDH17 | 0.10464295 | cadherin 17 |
| APOL1 | 0.10472727 | apolipoprotein L1 |
| PTS | 0.10475626 | 6-pyruvoyltetrahydropterin synthase |
| SEMA3E | 0.10484321 | semaphorin 3E |
| KCNIP3 | 0.10488801 | potassium voltage-gated channel interacting protein 3 |
| PGM1 | 0.10489855 | phosphoglucomutase 1 |
| CPA6 | 0.10501449 | carboxypeptidase A6 |
| TMEM242 | 0.1050224 | transmembrane protein 242 |
| ANKRD17 | 0.10529117 | ankyrin repeat domain 17 |
| SLC35A1 | 0.10538867 | solute carrier family 35 member A1 |
| OMD | 0.10539394 | osteomodulin |
| LRFN5 | 0.10539921 | leucine rich repeat and fibronectin type III domain containing 5 |
| IFITM3 | 0.10543083 | interferon induced transmembrane protein 3 |
| ADAMTS8 | 0.10550461 | ADAM metallopeptidase with thrombospondin type 1 motif 8 |
| SMIM12 | 0.10572069 | small integral membrane protein 12 |
| TCEAL7 | 0.10593412 | transcription elongation factor A like 7 |
| ANGPT1 | 0.10598946 | angiopoietin 1 |
| SLC2A12 | 0.10607115 | solute carrier family 2 member 12 |
| CFI | 0.10609223 | complement factor I |
| FAM180A | 0.10621344 | family with sequence similarity 180 member A |
| HIST1H3I | 0.10659816 | histone cluster 1, H3i |
| BRD3 | 0.10695652 | bromodomain containing 3 |
| LNP1 | 0.10711462 | leukemia NUP98 fusion partner 1 |
| ZNF330 | 0.10726482 | zinc finger protein 330 |
| FOXO1 | 0.10735178 | forkhead box O1 |
| MCMDC2 | 0.10736495 | minichromosome maintenance domain containing 2 |
| CCL7 | 0.10750988 | C-C motif chemokine ligand 7 |
| CD69 | 0.10775494 | CD69 molecule |
| RUSC2 | 0.10801054 | RUN and SH3 domain containing 2 |
| SLC5A7 | 0.10801845 | solute carrier family 5 member 7 |
| GAB1 | 0.10825033 | GRB2 associated binding protein 1 |
| ARHGAP20 | 0.10834519 | Rho GTPase activating protein 20 |
| ZNF33B | 0.10851383 | zinc finger protein 33B |
| WAPL | 0.1085191 | WAPL cohesin release factor |
| SLC7A2 | 0.10854545 | solute carrier family 7 member 2 |
| RNASE10 | 0.10859552 | ribonuclease A family member 10 (inactive) |
| DOCK2 | 0.1086614 | dedicator of cytokinesis 2 |
| AP4E1 | 0.10870356 | adaptor related protein complex 4 epsilon 1 subunit |
| KLHDC3 | 0.10872727 | kelch domain containing 3 |
| SLC2A14 | 0.10883267 | solute carrier family 2 member 14 |
| DDX3X | 0.10887747 | DEAD-box helicase 3, X-linked |
| CARF | 0.10893808 | calcium responsive transcription factor |
| RNASE4 | 0.10909091 | ribonuclease A family member 4 |
| C5orf24 | 0.1091357 | chromosome 5 open reading frame 24 |
| ANKRD42 | 0.10915152 | ankyrin repeat domain 42 |
| NBPF14 | 0.10926746 | neuroblastoma breakpoint family member 14 |
| CCNH | 0.10965481 | cyclin H |
| PGM5 | 0.10966798 | phosphoglucomutase 5 |
| RGS2 | 0.1096917 | regulator of G-protein signaling 2 |
| ABCA5 | 0.10982609 | ATP binding cassette subfamily A member 5 |
| PRUNE2 | 0.10990777 | prune homolog 2 |
| PTP4A1 | 0.10993939 | protein tyrosine phosphatase type IVA, member 1 |
| SGPP2 | 0.1100527 | sphingosine-1-phosphate phosphatase 2 |
| DACT1 | 0.11007378 | dishevelled binding antagonist of beta catenin 1 |
| PVR | 0.11016601 | poliovirus receptor |
| HIPK3 | 0.11017391 | homeodomain interacting protein kinase 3 |
| ZNF532 | 0.11018182 | zinc finger protein 532 |
| PRRG2 | 0.11028986 | proline rich and Gla domain 2 |
| LYVE1 | 0.11047694 | lymphatic vessel endothelial hyaluronan receptor 1 |
| KLRK1 | 0.11059025 | killer cell lectin like receptor K1 |
| HSD11B1L | 0.11072991 | hydroxysteroid 11-beta dehydrogenase 1 like |
| ZNF146 | 0.11081159 | zinc finger protein 146 |
| CPEB1 | 0.11120949 | cytoplasmic polyadenylation element binding protein 1 |
| MMADHC | 0.11122266 | methylmalonic aciduria and homocystinuria, cblD type |
| FAM150B | 0.11127009 | family with sequence similarity 150 member B |
| ERICH1 | 0.11130962 | glutamate rich 1 |
| DPCD | 0.11137022 | deleted in primary ciliary dyskinesia homolog (mouse) |
| GULP1 | 0.11137022 | GULP, engulfment adaptor PTB domain containing 1 |
| STXBP3 | 0.11177602 | syntaxin binding protein 3 |
| KCMF1 | 0.11211067 | potassium channel modulatory factor 1 |
| SDC2 | 0.11242161 | syndecan 2 |
| ACOT1 | 0.11268511 | acyl-CoA thioesterase 1 |
| EYA1 | 0.11275099 | EYA transcriptional coactivator and phosphatase 1 |
| LOC105379362 | 0.11285112 | uncharacterized LOC105379362 |
| UBAP2 | 0.11287747 | ubiquitin associated protein 2 |
| GALNT2 | 0.11305402 | polypeptide N-acetylgalactosaminyltransferase 2 |
| APCDD1 | 0.11313834 | APC down-regulated 1 |
| NT5DC1 | 0.11319368 | 5'-nucleotidase domain containing 1 |
| MRPL44 | 0.11328327 | mitochondrial ribosomal protein L44 |
| RAVER2 | 0.11333333 | ribonucleoprotein, PTB binding 2 |
| RPL23AP7 | 0.11335968 | ribosomal protein L23a pseudogene 7 |
| WDR83 | 0.11397892 | WD repeat domain 83 |
| AMY2B | 0.11400791 | amylase, alpha 2B (pancreatic) |
| DTNB | 0.11410804 | dystrobrevin beta |
| UPK1A | 0.11420026 | uroplakin 1A |
| RPS6KA4 | 0.11420026 | ribosomal protein S6 kinase A4 |
| GSKIP | 0.11438999 | GSK3B interacting protein |
| RIOK2 | 0.11440316 | RIO kinase 2 |
| PABPC1L2B-AS1 | 0.11469829 | PABPC1L2B antisense RNA 1 (head to head) |
| CORO6 | 0.11490646 | coronin 6 |
| ARHGEF10L | 0.11491173 | Rho guanine nucleotide exchange factor 10 like |
| HTR2B | 0.11495389 | 5-hydroxytryptamine receptor 2B |
| UBLCP1 | 0.11496443 | ubiquitin like domain containing CTD phosphatase 1 |
| ALAD | 0.11499868 | aminolevulinate dehydratase |
| SATB1 | 0.11506983 | SATB homeobox 1 |
| NAB1 | 0.11528327 | NGFI-A binding protein 1 |
| C9orf72 | 0.11532279 | chromosome 9 open reading frame 72 |
| SLC4A4 | 0.11535968 | solute carrier family 4 member 4 |
| EMP1 | 0.11543874 | epithelial membrane protein 1 |
| CMIP | 0.11549144 | c-Maf inducing protein |
| HARS2 | 0.11568906 | histidyl-tRNA synthetase 2, mitochondrial |
| NUDT22 | 0.11575758 | nudix hydrolase 22 |
| NCAM2 | 0.11584453 | neural cell adhesion molecule 2 |
| HRAS | 0.11602108 | HRas proto-oncogene, GTPase |
| TTC26 | 0.11609486 | tetratricopeptide repeat domain 26 |
| AAMDC | 0.11611858 | adipogenesis associated Mth938 domain containing |
| NPAT | 0.11617918 | nuclear protein, coactivator of histone transcription |
| CCDC50 | 0.11629513 | coiled-coil domain containing 50 |
| BDH2 | 0.11632938 | 3-hydroxybutyrate dehydrogenase, type 2 |
| LINC00854 | 0.11635837 | long intergenic non-protein coding RNA 854 |
| YY1 | 0.1166166 | YY1 transcription factor |
| MYEF2 | 0.11668248 | myelin expression factor 2 |
| LGR6 | 0.11679051 | leucine rich repeat containing G protein-coupled receptor 6 |
| HIST1H4H | 0.11693017 | histone cluster 1, H4h |
| SAP18 | 0.1169776 | Sin3A associated protein 18 |
| LOC105372795 | 0.11715942 | uncharacterized LOC105372795 |
| FDX1 | 0.11728327 | ferredoxin 1 |
| KCNN3 | 0.11746245 | potassium calcium-activated channel subfamily N member 3 |
| KCNN3 | 0.11746245 | potassium calcium-activated channel subfamily N member 3 |
| SLC35A5 | 0.11750725 | solute carrier family 35 member A5 |
| DNAH7 | 0.11778393 | dynein axonemal heavy chain 7 |
| ZNF713 | 0.11783136 | zinc finger protein 713 |
| PDIA3P1 | 0.11785771 | protein disulfide isomerase family A member 3 pseudogene 1 |
| RIMBP2 | 0.11817655 | RIMS binding protein 2 |
| ADAMTSL3 | 0.11818445 | ADAMTS like 3 |
| HIF1A | 0.11834783 | hypoxia inducible factor 1 alpha subunit |
| RNF112 | 0.11850593 | ring finger protein 112 |
| MRO | 0.11867457 | maestro |
| RPL13 | 0.11868511 | ribosomal protein L13 |
| EHBP1 | 0.11872727 | EH domain binding protein 1 |
| ATP6V0D1 | 0.11873518 | ATPase H+ transporting V0 subunit d1 |
| TSPAN2 | 0.11892754 | tetraspanin 2 |
| COPS4 | 0.11904348 | COP9 signalosome subunit 4 |
| VPS37B | 0.11904875 | VPS37B, ESCRT-I subunit |
| SEC23A | 0.11908564 | Sec23 homolog A, coat complex II component |
| TLR1 | 0.11910935 | toll like receptor 1 |
| FILIP1L | 0.11926482 | filamin A interacting protein 1 like |
| ZNF304 | 0.11945718 | zinc finger protein 304 |
| CYBB | 0.11949934 | cytochrome b-245 beta chain |
| XPO5 | 0.1196917 | exportin 5 |
| TTLL5 | 0.11987615 | tubulin tyrosine ligase like 5 |
| CALCOCO1 | 0.12025296 | calcium binding and coiled-coil domain 1 |
| OSTC | 0.12039789 | oligosaccharyltransferase complex non-catalytic subunit |
| USP51 | 0.12058235 | ubiquitin specific peptidase 51 |
| PDE4D | 0.12060343 | phosphodiesterase 4D |
| MBD5 | 0.12064822 | methyl-CpG binding domain protein 5 |
| CREB5 | 0.1208722 | cAMP responsive element binding protein 5 |
| ADH5 | 0.12113307 | alcohol dehydrogenase 5 (class III), chi polypeptide |
| AXIN1 | 0.12155995 | axin 1 |
| TPD52L1 | 0.12177602 | tumor protein D52-like 1 |
| NDUFB4 | 0.12180764 | NADH:ubiquinone oxidoreductase subunit B4 |
| PRSS16 | 0.12186561 | protease, serine 16 |
| LOC101928307 | 0.12201845 | uncharacterized LOC101928307 |
| MISP3 | 0.1224664 | MISP family member 3 |
| KLF6 | 0.12254545 | Kruppel like factor 6 |
| ACVR1 | 0.12266403 | activin A receptor type 1 |
| ZNF385B | 0.12269038 | zinc finger protein 385B |
| NR4A3 | 0.12282213 | nuclear receptor subfamily 4 group A member 3 |
| AASDHPPT | 0.12284058 | aminoadipate-semialdehyde dehydrogenase-phosphopantetheinyl transferase |
| BBS2 | 0.1228722 | Bardet-Biedl syndrome 2 |
| MORC3 | 0.12298024 | MORC family CW-type zinc finger 3 |
| SIPA1L1 | 0.12325955 | signal induced proliferation associated 1 like 1 |
| GNE | 0.12346772 | glucosamine (UDP-N-acetyl)-2-epimerase/N-acetylmannosamine kinase |
| WISP2 | 0.12370224 | WNT1 inducible signaling pathway protein 2 |
| MILR1 | 0.1237892 | mast cell immunoglobulin like receptor 1 |
| GNAQ | 0.12379183 | G protein subunit alpha q |
| BVES | 0.12379447 | blood vessel epicardial substance |
| WDR70 | 0.12390777 | WD repeat domain 70 |
| ZNF562 | 0.12399473 | zinc finger protein 562 |
| TARS2 | 0.124 | threonyl-tRNA synthetase 2, mitochondrial (putative) |
| PRMT6 | 0.12401845 | protein arginine methyltransferase 6 |
| SEL1L | 0.12403162 | SEL1L ERAD E3 ligase adaptor subunit |
| SAMSN1 | 0.12403689 | SAM domain, SH3 domain and nuclear localization signals 1 |
| ARSJ | 0.12419499 | arylsulfatase family member J |
| FUT2 | 0.12437945 | fucosyltransferase 2 |
| MAGI2 | 0.12440316 | membrane associated guanylate kinase, WW and PDZ domain containing 2 |
| JKAMP | 0.12451647 | JNK1/MAPK8-associated membrane protein |
| TP53INP2 | 0.12457181 | tumor protein p53 inducible nuclear protein 2 |
| NCOR2 | 0.12486693 | nuclear receptor corepressor 2 |
| CLEC3A | 0.12493017 | C-type lectin domain family 3 member A |
| STAT3 | 0.12505665 | signal transducer and activator of transcription 3 |
| HOXB7 | 0.12506719 | homeobox B7 |
| TSHZ2 | 0.12509881 | teashirt zinc finger homeobox 2 |
| TSHZ2 | 0.12509881 | teashirt zinc finger homeobox 2 |
| DCAF4L1 | 0.12530698 | DDB1 and CUL4 associated factor 4 like 1 |
| REEP5 | 0.12541238 | receptor accessory protein 5 |
| EPS15 | 0.12549144 | epidermal growth factor receptor pathway substrate 15 |
| MIER1 | 0.12567062 | MIER1 transcriptional regulator |
| ERCC6L2 | 0.12573123 | ERCC excision repair 6 like 2 |
| RAB23 | 0.1257365 | RAB23, member RAS oncogene family |
| DSEL | 0.12575231 | dermatan sulfate epimerase-like |
| SEMA3A | 0.12623452 | semaphorin 3A |
| PTPN4 | 0.12644005 | protein tyrosine phosphatase, non-receptor type 4 |
| ADCY9 | 0.12671146 | adenylate cyclase 9 |
| PKD2 | 0.12683531 | polycystin 2, transient receptor potential cation channel |
| MS4A6A | 0.12683531 | membrane spanning 4-domains A6A |
| REV1 | 0.12700132 | REV1, DNA directed polymerase |
| FBXO32 | 0.12700659 | F-box protein 32 |
| IGFBP2 | 0.12705665 | insulin like growth factor binding protein 2 |
| ID3 | 0.12710935 | inhibitor of DNA binding 3, HLH protein |
| PER3 | 0.12713307 | period circadian clock 3 |
| RALGAPA1 | 0.12718577 | Ral GTPase activating protein catalytic alpha subunit 1 |
| NAPA | 0.12719631 | NSF attachment protein alpha |
| EIF4EBP1 | 0.12736759 | eukaryotic translation initiation factor 4E binding protein 1 |
| HOXD3 | 0.12737813 | homeobox D3 |
| ZFAT | 0.12744928 | zinc finger and AT-hook domain containing |
| FUT4 | 0.12747826 | fucosyltransferase 4 |
| BARD1 | 0.12766535 | BRCA1 associated RING domain 1 |
| GCH1 | 0.12781555 | GTP cyclohydrolase 1 |
| LTBP4 | 0.12787088 | latent transforming growth factor beta binding protein 4 |
| VENTX | 0.12814493 | VENT homeobox |
| CYB5D1 | 0.12823452 | cytochrome b5 domain containing 1 |
| AMBRA1 | 0.12833465 | autophagy and beclin 1 regulator 1 |
| TUT1 | 0.12854282 | terminal uridylyl transferase 1, U6 snRNA-specific |
| DHX29 | 0.1285639 | DEAH-box helicase 29 |
| PDZRN4 | 0.1286693 | PDZ domain containing ring finger 4 |
| RAB11FIP2 | 0.12876943 | RAB11 family interacting protein 2 |
| CTSL | 0.12882213 | cathepsin L |
| HIST1H3G | 0.12887484 | histone cluster 1, H3g |
| MTCH2 | 0.12893017 | mitochondrial carrier 2 |
| VPS13D | 0.12899341 | vacuolar protein sorting 13 homolog D |
| SNHG8 | 0.12911726 | small nucleolar RNA host gene 8 |
| CYCS | 0.12925428 | cytochrome c, somatic |
| SLC25A31 | 0.12932806 | solute carrier family 25 member 31 |
| CELF2 | 0.1293307 | CUGBP, Elav-like family member 2 |
| EFNA3 | 0.12947826 | ephrin A3 |
| SKIV2L | 0.1294809 | Ski2 like RNA helicase |
| KCNB1 | 0.12953623 | potassium voltage-gated channel subfamily B member 1 |
| AKNA | 0.12954414 | AT-hook transcription factor |
| PEG3 | 0.12977602 | paternally expressed 3 |
| RHBDD1 | 0.12983399 | rhomboid domain containing 1 |
| HIVEP1 | 0.12996311 | human immunodeficiency virus type I enhancer binding protein 1 |
| NEK6 | 0.12997892 | NIMA related kinase 6 |
| DSE | 0.13010277 | dermatan sulfate epimerase |
| GPR183 | 0.13029776 | G protein-coupled receptor 183 |
| RNF19B | 0.13065349 | ring finger protein 19B |
| ZBED8 | 0.13082213 | zinc finger BED-type containing 8 |
| RBM24 | 0.13119104 | RNA binding motif protein 24 |
| CDKL1 | 0.13137022 | cyclin dependent kinase like 1 |
| CLEC4A | 0.13156785 | C-type lectin domain family 4 member A |
| CHD5 | 0.13160211 | chromodomain helicase DNA binding protein 5 |
| PHF1 | 0.13165744 | PHD finger protein 1 |
| ZNF205 | 0.13167852 | zinc finger protein 205 |
| NWD1 | 0.13173123 | NACHT and WD repeat domain containing 1 |
| NAPG | 0.13183136 | NSF attachment protein gamma |
| RIC3 | 0.13213966 | RIC3 acetylcholine receptor chaperone |
| PIP5K1A | 0.13213966 | phosphatidylinositol-4-phosphate 5-kinase type 1 alpha |
| DOCK8 | 0.13219236 | dedicator of cytokinesis 8 |
| COL4A5 | 0.13254545 | collagen type IV alpha 5 chain |
| UBR4 | 0.13272991 | ubiquitin protein ligase E3 component n-recognin 4 |
| ACVR1B | 0.13272991 | activin A receptor type 1B |
| CYP4V2 | 0.13276943 | cytochrome P450 family 4 subfamily V member 2 |
| RNF169 | 0.13277997 | ring finger protein 169 |
| RHOB | 0.13286693 | ras homolog family member B |
| FAM189A2 | 0.13287484 | family with sequence similarity 189 member A2 |
| MMP2 | 0.132917 | matrix metallopeptidase 2 |
| SLC26A5 | 0.13301976 | solute carrier family 26 member 5 |
| FAM120AOS | 0.13365217 | family with sequence similarity 120A opposite strand |
| FAM213B | 0.13376021 | family with sequence similarity 213 member B |
| ACP2 | 0.13378129 | acid phosphatase 2, lysosomal |
| TSPAN4 | 0.13385771 | tetraspanin 4 |
| IGFL3 | 0.13386561 | IGF like family member 3 |
| DNAJB5 | 0.13406324 | DnaJ heat shock protein family (Hsp40) member B5 |
| SDHAF2 | 0.13413702 | succinate dehydrogenase complex assembly factor 2 |
| TM2D1 | 0.13414493 | TM2 domain containing 1 |
| PRKCB | 0.13425296 | protein kinase C beta |
| PDDC1 | 0.13427404 | Parkinson disease 7 domain containing 1 |
| EIF3M | 0.13448485 | eukaryotic translation initiation factor 3 subunit M |
| BICC1 | 0.1346693 | BicC family RNA binding protein 1 |
| PIGQ | 0.13484585 | phosphatidylinositol glycan anchor biosynthesis class Q |
| B4GAT1 | 0.13487747 | beta-1,4-glucuronyltransferase 1 |
| DAPP1 | 0.13499868 | dual adaptor of phosphotyrosine and 3-phosphoinositides 1 |
| BCL10 | 0.13500659 | B-cell CLL/lymphoma 10 |
| HS3ST5 | 0.13514361 | heparan sulfate-glucosamine 3-sulfotransferase 5 |
| PAPSS2 | 0.13515679 | 3'-phosphoadenosine 5'-phosphosulfate synthase 2 |
| ADH1A | 0.13532806 | alcohol dehydrogenase 1A (class I), alpha polypeptide |
| FHL1 | 0.13537549 | four and a half LIM domains 1 |
| NEK11 | 0.13558103 | NIMA related kinase 11 |
| PDE3B | 0.13558366 | phosphodiesterase 3B |
| TINF2 | 0.13561528 | TERF1 interacting nuclear factor 2 |
| CACNA2D3 | 0.13633465 | calcium voltage-gated channel auxiliary subunit alpha2delta 3 |
| AGTRAP | 0.136361 | angiotensin II receptor associated protein |
| KBTBD3 | 0.13659025 | kelch repeat and BTB domain containing 3 |
| FOXD4L1 | 0.13660079 | forkhead box D4-like 1 |
| MSN | 0.13665086 | moesin |
| PAFAH1B1 | 0.13668248 | platelet activating factor acetylhydrolase 1b regulatory subunit 1 |
| ACTRT1 | 0.13677207 | actin related protein T1 |
| TPD52L2 | 0.13680105 | tumor protein D52 like 2 |
| CALCB | 0.13688274 | calcitonin related polypeptide beta |
| MRPS18C | 0.13715415 | mitochondrial ribosomal protein S18C |
| MYH3 | 0.13731752 | myosin, heavy chain 3, skeletal muscle, embryonic |
| NR5A1 | 0.1373307 | nuclear receptor subfamily 5 group A member 1 |
| ASNA1 | 0.13735968 | arsA arsenite transporter, ATP-binding, homolog 1 (bacterial) |
| CWF19L2 | 0.13746245 | CWF19-like 2, cell cycle control (S. pombe) |
| CCDC102A | 0.13751515 | coiled-coil domain containing 102A |
| CFH | 0.13762319 | complement factor H |
| RASA3 | 0.13769433 | RAS p21 protein activator 3 |
| GNG12 | 0.13770751 | G protein subunit gamma 12 |
| PAPPA | 0.13788933 | pappalysin 1 |
| ANAPC10 | 0.13788933 | anaphase promoting complex subunit 10 |
| DBNDD1 | 0.13799736 | dysbindin domain containing 1 |
| SMPDL3B | 0.13813439 | sphingomyelin phosphodiesterase acid like 3B |
| SIAH2 | 0.1382556 | siah E3 ubiquitin protein ligase 2 |
| RNF217 | 0.13852437 | ring finger protein 217 |
| IL18R1 | 0.13863768 | interleukin 18 receptor 1 |
| FAM8A1 | 0.13898287 | family with sequence similarity 8 member A1 |
| WDR24 | 0.13899605 | WD repeat domain 24 |
| AFF4 | 0.1390303 | AF4/FMR2 family member 4 |
| C14orf169 | 0.13915152 | chromosome 14 open reading frame 169 |
| NEFM | 0.13927536 | neurofilament, medium polypeptide |
| RPL12 | 0.13928327 | ribosomal protein L12 |
| TCEAL1 | 0.13945982 | transcription elongation factor A like 1 |
| MAFF | 0.13950461 | MAF bZIP transcription factor F |
| ATP8A1 | 0.13954677 | ATPase phospholipid transporting 8A1 |
| APCDD1L | 0.13972332 | APC down-regulated 1 like |
| GNAI1 | 0.13972596 | G protein subunit alpha i1 |
| TMEM27 | 0.13981028 | transmembrane protein 27 |
| KIAA0556 | 0.13988142 | KIAA0556 |
| METAP1 | 0.14005007 | methionyl aminopeptidase 1 |
| KLK10 | 0.14008696 | kallikrein related peptidase 10 |
| ALKBH3 | 0.14032148 | alkB homolog 3, alpha-ketoglutaratedependent dioxygenase |
| CSNK1A1 | 0.14038208 | casein kinase 1 alpha 1 |
| MMAA | 0.14062451 | methylmalonic aciduria (cobalamin deficiency) cblA type |
| LINGO2 | 0.14077734 | leucine rich repeat and Ig domain containing 2 |
| GRIN1 | 0.14086693 | glutamate ionotropic receptor NMDA type subunit 1 |
| DYRK3 | 0.14172859 | dual specificity tyrosine phosphorylation regulated kinase 3 |
| TGFBRAP1 | 0.14173123 | transforming growth factor beta receptor associated protein 1 |
| LOC101929523 | 0.14174704 | uncharacterized LOC101929523 |
| RCOR3 | 0.14183926 | REST corepressor 3 |
| KMT2E | 0.1424585 | lysine methyltransferase 2E |
| GSN | 0.14262978 | gelsolin |
| ZC3H7A | 0.14270356 | zinc finger CCCH-type containing 7A |
| ANPEP | 0.14274572 | alanyl aminopeptidase, membrane |
| RNF145 | 0.14274835 | ring finger protein 145 |
| MYO1E | 0.14283531 | myosin IE |
| LYST | 0.14356258 | lysosomal trafficking regulator |
| PPM1K | 0.14375494 | protein phosphatase, Mg2+/Mn2+ dependent 1K |
| RWDD3 | 0.14378656 | RWD domain containing 3 |
| FCER1G | 0.1438419 | Fc fragment of IgE receptor Ig |
| CAB39 | 0.14399736 | calcium binding protein 39 |
| PIWIL4 | 0.14413439 | piwi like RNA-mediated gene silencing 4 |
| POPDC2 | 0.14414756 | popeye domain containing 2 |
| ZMAT2 | 0.14418445 | zinc finger matrin-type 2 |
| GALC | 0.14427141 | galactosylceramidase |
| TTLL11 | 0.1443004 | tubulin tyrosine ligase like 11 |
| TTLL11 | 0.1443004 | tubulin tyrosine ligase like 11 |
| TTLL11 | 0.1443004 | tubulin tyrosine ligase like 11 |
| CBFA2T3 | 0.14432148 | CBFA2/RUNX1 translocation partner 3 |
| ARHGEF6 | 0.14442161 | Rac/Cdc42 guanine nucleotide exchange factor 6 |
| DNAJC25 | 0.14469038 | DnaJ heat shock protein family (Hsp40) member C25 |
| CCSER2 | 0.14491436 | coiled-coil serine rich protein 2 |
| NAT10 | 0.14500132 | N-acetyltransferase 10 |
| HSD17B4 | 0.14534124 | hydroxysteroid 17-beta dehydrogenase 4 |
| OPTN | 0.14557049 | optineurin |
| LDLRAD3 | 0.14576548 | low density lipoprotein receptor class A domain containing 3 |
| BTC | 0.14659816 | betacellulin |
| F12 | 0.14667457 | coagulation factor XII |
| MRAS | 0.14671146 | muscle RAS oncogene homolog |
| SORCS1 | 0.14672991 | sortilin related VPS10 domain containing receptor 1 |
| VPS11 | 0.14699341 | VPS11, CORVET/HOPS core subunit |
| TSC22D1 | 0.14703557 | TSC22 domain family member 1 |
| ASB5 | 0.14705402 | ankyrin repeat and SOCS box containing 5 |
| C11orf96 | 0.14705665 | chromosome 11 open reading frame 96 |
| LGALS4 | 0.14708037 | galectin 4 |
| OR52N2 | 0.14711989 | olfactory receptor family 52 subfamily N member 2 |
| KPTN | 0.14726219 | kaptin, actin binding protein |
| FAM172A | 0.14744401 | family with sequence similarity 172 member A |
| SIRT4 | 0.14745455 | sirtuin 4 |
| BBS9 | 0.14781291 | Bardet-Biedl syndrome 9 |
| ANOS1 | 0.1479025 | anosmin 1 |
| PRTFDC1 | 0.14830303 | phosphoribosyl transferase domain containing 1 |
| NR4A1 | 0.14835046 | nuclear receptor subfamily 4 group A member 1 |
| HIST1H3D | 0.14837681 | histone cluster 1, H3d |
| PTPRN2 | 0.14843215 | protein tyrosine phosphatase, receptor type N2 |
| CERS4 | 0.14881686 | ceramide synthase 4 |
| FILIP1 | 0.14894598 | filamin A interacting protein 1 |
| DOCK11 | 0.14895125 | dedicator of cytokinesis 11 |
| ZMAT1 | 0.14897497 | zinc finger matrin-type 1 |
| STRN3 | 0.14898024 | striatin 3 |
| SULT1E1 | 0.14900659 | sulfotransferase family 1E member 1 |
| PDGFC | 0.14911989 | platelet derived growth factor C |
| NUDT11 | 0.14948617 | nudix hydrolase 11 |
| GATA6-AS1 | 0.14949934 | GATA6 antisense RNA 1 (head to head) |
| SCHIP1 | 0.14957049 | schwannomin interacting protein 1 |
| BANK1 | 0.1496469 | B-cell scaffold protein with ankyrin repeats 1 |
| HIPK2 | 0.14964954 | homeodomain interacting protein kinase 2 |
| EID3 | 0.14974967 | EP300 interacting inhibitor of differentiation 3 |
| ABCA8 | 0.14982609 | ATP binding cassette subfamily A member 8 |
| MTR | 0.14989723 | 5-methyltetrahydrofolate-homocysteine methyltransferase |
| PDP1 | 0.1499025 | pyruvate dehyrogenase phosphatase catalytic subunit 1 |
| NTN1 | 0.14991568 | netrin 1 |
| RPS13 | 0.15000264 | ribosomal protein S13 |
| BSPRY | 0.15003953 | B-box and SPRY domain containing |
| COG3 | 0.15010277 | component of oligomeric golgi complex 3 |
| JRKL | 0.15017128 | JRK-like |
| NACAD | 0.15031357 | NAC alpha domain containing |
| JPH2 | 0.15050066 | junctophilin 2 |
| C1orf116 | 0.15053491 | chromosome 1 open reading frame 116 |
| GNPDA2 | 0.15076943 | glucosamine-6-phosphate deaminase 2 |
| SPA17 | 0.15078788 | sperm autoantigenic protein 17 |
| GPM6B | 0.1508722 | glycoprotein M6B |
| ZNF707 | 0.15100395 | zinc finger protein 707 |
| ADARB1 | 0.15106456 | adenosine deaminase, RNA specific B1 |
| ACTR10 | 0.15113834 | actin-related protein 10 homolog |
| SH3GL3 | 0.1513386 | SH3 domain containing GRB2 like 3, endophilin A3 |
| SURF2 | 0.15134124 | surfeit 2 |
| CCDC178 | 0.15135178 | coiled-coil domain containing 178 |
| CEBPD | 0.15141238 | CCAAT/enhancer binding protein delta |
| TPRG1L | 0.15142556 | tumor protein p63 regulated 1-like |
| YAP1 | 0.15173123 | Yes associated protein 1 |
| CCL2 | 0.15189987 | C-C motif chemokine ligand 2 |
| MICAL1 | 0.15189987 | microtubule associated monooxygenase, calponin and LIM domain containing 1 |
| BNC2 | 0.15199209 | basonuclin 2 |
| SLC25A22 | 0.15228195 | solute carrier family 25 member 22 |
| CYB5R3 | 0.1524058 | cytochrome b5 reductase 3 |
| HBP1 | 0.15261397 | HMG-box transcription factor 1 |
| YTHDF3 | 0.15315415 | YTH N6-methyladenosine RNA binding protein 3 |
| NPAS4 | 0.1531805 | neuronal PAS domain protein 4 |
| ALDH3A1 | 0.15325165 | aldehyde dehydrogenase 3 family member A1 |
| NUP188 | 0.15326482 | nucleoporin 188 |
| TNS1 | 0.15331225 | tensin 1 |
| C19orf45 | 0.153639 | chromosome 19 open reading frame 45 |
| TCEAL2 | 0.15372859 | transcription elongation factor A like 2 |
| SEP12 | 0.15383399 | septin 12 |
| SPAG17 | 0.1538498 | sperm associated antigen 17 |
| HSD17B12 | 0.15414756 | hydroxysteroid 17-beta dehydrogenase 12 |
| TMEM107 | 0.15435046 | transmembrane protein 107 |
| CHMP2B | 0.1544585 | charged multivesicular body protein 2B |
| PIP5KL1 | 0.15454018 | phosphatidylinositol-4-phosphate 5-kinase like 1 |
| AP1M2 | 0.15458762 | adaptor related protein complex 1 mu 2 subunit |
| FMNL2 | 0.1548643 | formin like 2 |
| EXPH5 | 0.15499078 | exophilin 5 |
| LIPA | 0.15500132 | lipase A, lysosomal acid type |
| SPATA33 | 0.1550303 | spermatogenesis associated 33 |
| SRSF8 | 0.15506456 | serine and arginine rich splicing factor 8 |
| ADAMTS9-AS1 | 0.15516206 | ADAMTS9 antisense RNA 1 |
| CSTF2T | 0.15542292 | cleavage stimulation factor subunit 2, tau variant |
| API5 | 0.15544401 | apoptosis inhibitor 5 |
| CLK4 | 0.15601054 | CDC like kinase 4 |
| LINC00112 | 0.15616337 | long intergenic non-protein coding RNA 112 |
| CTNNAL1 | 0.15645586 | catenin alpha like 1 |
| HSPB3 | 0.15662714 | heat shock protein family B (small) member 3 |
| TLN1 | 0.15668775 | talin 1 |
| F13A1 | 0.15670356 | coagulation factor XIII A chain |
| SERINC1 | 0.15670619 | serine incorporator 1 |
| PRSS23 | 0.15674308 | protease, serine 23 |
| ABCA9 | 0.15677207 | ATP binding cassette subfamily A member 9 |
| CNBP | 0.15683531 | CCHC-type zinc finger nucleic acid binding protein |
| SLC22A3 | 0.15705138 | solute carrier family 22 member 3 |
| RORB | 0.15726746 | RAR related orphan receptor B |
| DNAJC22 | 0.15736495 | DnaJ heat shock protein family (Hsp40) member C22 |
| RNASE6 | 0.15754414 | ribonuclease A family member k6 |
| GPR37 | 0.15757576 | G protein-coupled receptor 37 |
| VSIG8 | 0.15757576 | V-set and immunoglobulin domain containing 8 |
| CNTN1 | 0.15760211 | contactin 1 |
| ALDH1B1 | 0.15783663 | aldehyde dehydrogenase 1 family member B1 |
| MRPL17 | 0.15797628 | mitochondrial ribosomal protein L17 |
| PTPN14 | 0.15800791 | protein tyrosine phosphatase, non-receptor type 14 |
| ZHX2 | 0.15815547 | zinc fingers and homeoboxes 2 |
| NSA2 | 0.15820026 | NSA2, ribosome biogenesis homolog |
| FLII | 0.15835837 | FLII, actin remodeling protein |
| CLSPN | 0.1585639 | claspin |
| CLSPN | 0.1585639 | claspin |
| GEM | 0.15860343 | GTP binding protein overexpressed in skeletal muscle |
| SRPX | 0.15867721 | sushi repeat containing protein, X-linked |
| TMEM71 | 0.15868775 | transmembrane protein 71 |
| SNX24 | 0.15869565 | sorting nexin 24 |
| TUBB3 | 0.15898814 | tubulin beta 3 class III |
| SCRG1 | 0.15917787 | stimulator of chondrogenesis 1 |
| LMO3 | 0.15929381 | LIM domain only 3 |
| CRY2 | 0.15970487 | cryptochrome circadian clock 2 |
| CLIC1 | 0.15976548 | chloride intracellular channel 1 |
| KIAA0513 | 0.1597971 | KIAA0513 |
| SUSD6 | 0.1598946 | sushi domain containing 6 |
| ISL1 | 0.1599552 | ISL LIM homeobox 1 |
| MTM1 | 0.16075099 | myotubularin 1 |
| KLF2 | 0.16075362 | Kruppel like factor 2 |
| WBP1L | 0.16081159 | WW domain binding protein 1-like |
| TLR6 | 0.1608274 | toll like receptor 6 |
| OTUD6B-AS1 | 0.16096706 | OTUD6B antisense RNA 1 (head to head) |
| CCDC125 | 0.16107773 | coiled-coil domain containing 125 |
| CCL4 | 0.16110672 | C-C motif chemokine ligand 4 |
| FAM184A | 0.16116733 | family with sequence similarity 184 member A |
| SARAF | 0.16138867 | store-operated calcium entry associated regulatory factor |
| CXCL9 | 0.16147826 | C-X-C motif chemokine ligand 9 |
| RBM43 | 0.16154677 | RNA binding motif protein 43 |
| IGSF21 | 0.16162319 | immunoglobin superfamily member 21 |
| CPEB3 | 0.16167062 | cytoplasmic polyadenylation element binding protein 3 |
| TRAPPC13 | 0.16218972 | trafficking protein particle complex 13 |
| FAM229B | 0.16270619 | family with sequence similarity 229 member B |
| SELE | 0.16288274 | selectin E |
| DTWD1 | 0.16304084 | DTW domain containing 1 |
| FAXC | 0.16354677 | failed axon connections homolog |
| FAXC | 0.16354677 | failed axon connections homolog |
| STAM2 | 0.16362055 | signal transducing adaptor molecule 2 |
| DNAL1 | 0.16375494 | dynein axonemal light chain 1 |
| CYP20A1 | 0.16385507 | cytochrome P450 family 20 subfamily A member 1 |
| GLIPR1 | 0.16388669 | GLI pathogenesis related 1 |
| ACACB | 0.16392622 | acetyl-CoA carboxylase beta |
| APOC1 | 0.16393939 | apolipoprotein C1 |
| SH3D19 | 0.16410277 | SH3 domain containing 19 |
| LRIG3 | 0.16411067 | leucine rich repeats and immunoglobulin like domains 3 |
| ANKRD37 | 0.16441634 | ankyrin repeat domain 37 |
| ZNF385D | 0.16452964 | zinc finger protein 385D |
| TNFRSF10D | 0.16469038 | TNF receptor superfamily member 10d |
| DMRTA1 | 0.16471937 | DMRT like family A1 |
| IL7 | 0.16474835 | interleukin 7 |
| NEXN | 0.16479051 | nexilin F-actin binding protein |
| GPD1 | 0.16484058 | glycerol-3-phosphate dehydrogenase 1 |
| CLINT1 | 0.16487484 | clathrin interactor 1 |
| LXN | 0.16488538 | latexin |
| KLHL9 | 0.16521212 | kelch like family member 9 |
| AUH | 0.16530698 | AU RNA binding methylglutaconyl-CoA hydratase |
| FAM188A | 0.16532806 | family with sequence similarity 188 member A |
| PROC | 0.16533333 | protein C, inactivator of coagulation factors Va and VIIIa |
| MAN1B1 | 0.16533333 | mannosidase alpha class 1B member 1 |
| REXO4 | 0.16549671 | REX4 homolog, 3'-5' exonuclease |
| GABARAPL2 | 0.16552569 | GABA type A receptor associated protein like 2 |
| ARMCX1 | 0.16561001 | armadillo repeat containing, X-linked 1 |
| CDK8 | 0.16563109 | cyclin dependent kinase 8 |
| LONRF3 | 0.16590514 | LON peptidase N-terminal domain and ring finger 3 |
| TGFBR2 | 0.16604216 | transforming growth factor beta receptor 2 |
| TCF21 | 0.16607905 | transcription factor 21 |
| TNIK | 0.1661502 | TRAF2 and NCK interacting kinase |
| ZKSCAN3 | 0.16618972 | zinc finger with KRAB and SCAN domains 3 |
| PARVA | 0.16619499 | parvin alpha |
| KCTD18 | 0.1662635 | potassium channel tetramerization domain containing 18 |
| SAV1 | 0.16643215 | salvador family WW domain containing protein 1 |
| TMEM54 | 0.16680896 | transmembrane protein 54 |
| KLHL41 | 0.16750461 | kelch like family member 41 |
| LOC102467079 | 0.16780237 | uncharacterized LOC102467079 |
| TNFRSF19 | 0.16782609 | TNF receptor superfamily member 19 |
| TMEM56 | 0.16787088 | transmembrane protein 56 |
| MITF | 0.16829249 | melanogenesis associated transcription factor |
| ZP3 | 0.16832938 | zona pellucida glycoprotein 3 (sperm receptor) |
| TBC1D1 | 0.16836891 | TBC1 domain family member 1 |
| MAML3 | 0.1684585 | mastermind like transcriptional coactivator 3 |
| ENAH | 0.16949144 | enabled homolog (Drosophila) |
| CCDC136 | 0.16972596 | coiled-coil domain containing 136 |
| TBCK | 0.16979974 | TBC1 domain containing kinase |
| ASPA | 0.1698498 | aspartoacylase |
| DDX6 | 0.16998682 | DEAD-box helicase 6 |
| BBOF1 | 0.17001845 | basal body orientation factor 1 |
| KCTD12 | 0.17005797 | potassium channel tetramerization domain containing 12 |
| C1orf162 | 0.17018972 | chromosome 1 open reading frame 162 |
| TRAF5 | 0.17090119 | TNF receptor associated factor 5 |
| TRPC1 | 0.17093281 | transient receptor potential cation channel subfamily C member 1 |
| ZNF277 | 0.17094862 | zinc finger protein 277 |
| DUSP3 | 0.17095389 | dual specificity phosphatase 3 |
| ALDH3A2 | 0.17098287 | aldehyde dehydrogenase 3 family member A2 |
| GABARAPL1 | 0.17116733 | GABA type A receptor associated protein like 1 |
| GPRASP2 | 0.17125428 | G protein-coupled receptor associated sorting protein 2 |
| MTUS2 | 0.17138603 | microtubule associated tumor suppressor candidate 2 |
| IL15 | 0.17151252 | interleukin 15 |
| CYSLTR1 | 0.17164427 | cysteinyl leukotriene receptor 1 |
| NCAM1 | 0.17182872 | neural cell adhesion molecule 1 |
| NIFK | 0.17192095 | nucleolar protein interacting with the FHA domain of MKI67 |
| NPHP4 | 0.17202899 | nephronophthisis 4 |
| DNAJC24 | 0.17220026 | DnaJ heat shock protein family (Hsp40) member C24 |
| COMMD6 | 0.17231357 | COMM domain containing 6 |
| PEX14 | 0.17243478 | peroxisomal biogenesis factor 14 |
| ZBTB21 | 0.17244796 | zinc finger and BTB domain containing 21 |
| ELMSAN1 | 0.17248221 | ELM2 and Myb/SANT domain containing 1 |
| MNDA | 0.17275362 | myeloid cell nuclear differentiation antigen |
| BEND6 | 0.17303294 | BEN domain containing 6 |
| HLA-DOA | 0.17316469 | major histocompatibility complex, class II, DO alpha |
| DISP2 | 0.17325428 | dispatched RND transporter family member 2 |
| ARFIP1 | 0.17325692 | ADP ribosylation factor interacting protein 1 |
| PJA2 | 0.17333597 | praja ring finger ubiquitin ligase 2 |
| ZNF626 | 0.17341765 | zinc finger protein 626 |
| ZFAND1 | 0.17382609 | zinc finger AN1-type containing 1 |
| CENPC | 0.17391568 | centromere protein C |
| WDR44 | 0.17398682 | WD repeat domain 44 |
| HCK | 0.17433202 | HCK proto-oncogene, Src family tyrosine kinase |
| CUTC | 0.17433729 | cutC copper transporter |
| ZMAT3 | 0.17445059 | zinc finger matrin-type 3 |
| METTL14 | 0.17447167 | methyltransferase like 14 |
| PAPD4 | 0.17448748 | poly(A) RNA polymerase D4, non-canonical |
| RPS6KA1 | 0.17455072 | ribosomal protein S6 kinase A1 |
| PI16 | 0.1747141 | peptidase inhibitor 16 |
| LMBRD2 | 0.17491436 | LMBR1 domain containing 2 |
| PTTG1IP | 0.1749249 | pituitary tumor-transforming 1 interacting protein |
| KIAA0196 | 0.17533597 | KIAA0196 |
| TIGD2 | 0.17586034 | tigger transposable element derived 2 |
| TTLL7 | 0.17604216 | tubulin tyrosine ligase like 7 |
| TTLL7 | 0.17604216 | tubulin tyrosine ligase like 7 |
| IGIP | 0.1765112 | IgA inducing protein |
| HLA-A | 0.1766166 | major histocompatibility complex, class I, A |
| SRI | 0.17690646 | sorcin |
| ST3GAL3 | 0.17708827 | ST3 beta-galactoside alpha-2,3-sialyltransferase 3 |
| CSF1R | 0.17727536 | colony stimulating factor 1 receptor |
| SBDS | 0.17727536 | SBDS ribosome assembly guanine nucleotide exchange factor |
| ZDHHC2 | 0.17783399 | zinc finger DHHC-type containing 2 |
| KIF13B | 0.17785507 | kinesin family member 13B |
| ARHGEF10 | 0.17846113 | Rho guanine nucleotide exchange factor 10 |
| CREB3 | 0.17862187 | cAMP responsive element binding protein 3 |
| LRCH2 | 0.17875099 | leucine rich repeats and calponin homology domain containing 2 |
| HSD17B6 | 0.17878261 | hydroxysteroid 17-beta dehydrogenase 6 |
| CTR9 | 0.17900132 | CTR9 homolog, Paf1/RNA polymerase II complex component |
| MPC1 | 0.17937813 | mitochondrial pyruvate carrier 1 |
| MYADM | 0.17944137 | myeloid associated differentiation marker |
| CDC42BPA | 0.17983136 | CDC42 binding protein kinase alpha |
| ZFP2 | 0.17984717 | ZFP2 zinc finger protein |
| ATP6V1D | 0.18041107 | ATPase H+ transporting V1 subunit D |
| RRAS2 | 0.18046113 | related RAS viral (r-ras) oncogene homolog 2 |
| NOXO1 | 0.18058762 | NADPH oxidase organizer 1 |
| TRAPPC4 | 0.18061397 | trafficking protein particle complex 4 |
| NFE2L2 | 0.18079842 | nuclear factor, erythroid 2 like 2 |
| HSPA2 | 0.18143874 | heat shock protein family A (Hsp70) member 2 |
| GALNT12 | 0.18163373 | polypeptide N-acetylgalactosaminyltransferase 12 |
| HID1 | 0.1822029 | HID1 domain containing |
| RECK | 0.1822635 | reversion inducing cysteine rich protein with kazal motifs |
| TSPAN10 | 0.18267984 | tetraspanin 10 |
| SLC39A8 | 0.18293017 | solute carrier family 39 member 8 |
| TMEM55A | 0.18301449 | transmembrane protein 55A |
| PACSIN3 | 0.18305665 | protein kinase C and casein kinase substrate in neurons 3 |
| SLCO2B1 | 0.18305665 | solute carrier organic anion transporter family member 2B1 |
| HOOK1 | 0.18335178 | hook microtubule tethering protein 1 |
| SRGAP1 | 0.18342029 | SLIT-ROBO Rho GTPase activating protein 1 |
| TNC | 0.18344664 | tenascin C |
| CCDC146 | 0.18345982 | coiled-coil domain containing 146 |
| PDK4 | 0.18356785 | pyruvate dehydrogenase kinase 4 |
| PPP2CB | 0.18368643 | protein phosphatase 2 catalytic subunit beta |
| TRIM27 | 0.18376285 | tripartite motif containing 27 |
| DUSP5 | 0.18391041 | dual specificity phosphatase 5 |
| TNNT1 | 0.18392622 | troponin T1, slow skeletal type |
| BDKRB1 | 0.1842029 | bradykinin receptor B1 |
| JAZF1 | 0.18421871 | JAZF zinc finger 1 |
| ACVR2A | 0.18426614 | activin A receptor type 2A |
| AK4 | 0.1847668 | adenylate kinase 4 |
| AK4 | 0.1847668 | adenylate kinase 4 |
| KATNAL1 | 0.1852859 | katanin catalytic subunit A1 like 1 |
| EIF1 | 0.18538603 | eukaryotic translation initiation factor 1 |
| SRR | 0.18558366 | serine racemase |
| ARAP2 | 0.18560738 | ArfGAP with RhoGAP domain, ankyrin repeat and PH domain 2 |
| SPATA6 | 0.18566798 | spermatogenesis associated 6 |
| DNAJB4 | 0.18596574 | DnaJ heat shock protein family (Hsp40) member B4 |
| PTAFR | 0.18636364 | platelet activating factor receptor |
| NFIL3 | 0.18658762 | nuclear factor, interleukin 3 regulated |
| COX20 | 0.18739394 | COX20, cytochrome c oxidase assembly factor |
| CHMP5 | 0.18783663 | charged multivesicular body protein 5 |
| SOCS3 | 0.18811067 | suppressor of cytokine signaling 3 |
| COLEC12 | 0.18818709 | collectin subfamily member 12 |
| SPEG | 0.18821344 | SPEG complex locus |
| SLC22A18AS | 0.18831094 | solute carrier family 22 member 18 antisense |
| CIPC | 0.18851647 | CLOCK interacting pacemaker |
| RPS6KA5 | 0.18872727 | ribosomal protein S6 kinase A5 |
| MPP1 | 0.18876416 | membrane palmitoylated protein 1 |
| SERP2 | 0.18880632 | stress-associated endoplasmic reticulum protein family member 2 |
| ETF1 | 0.18901186 | eukaryotic translation termination factor 1 |
| LRTM1 | 0.18920422 | leucine rich repeats and transmembrane domains 1 |
| MFF | 0.18942029 | mitochondrial fission factor |
| CREBRF | 0.18943874 | CREB3 regulatory factor |
| BTN2A1 | 0.18953623 | butyrophilin subfamily 2 member A1 |
| NDUFB6 | 0.19002635 | NADH:ubiquinone oxidoreductase subunit B6 |
| PPP1R16A | 0.19008432 | protein phosphatase 1 regulatory subunit 16A |
| GAS6 | 0.19008696 | growth arrest specific 6 |
| PCDH20 | 0.1901502 | protocadherin 20 |
| SERPING1 | 0.19052964 | serpin family G member 1 |
| POF1B | 0.19119368 | premature ovarian failure, 1B |
| ITGAM | 0.19125165 | integrin subunit alpha M |
| LILRB3 | 0.19157312 | leukocyte immunoglobulin like receptor B3 |
| BEGAIN | 0.19170224 | brain enriched guanylate kinase associated |
| TMED10 | 0.19248748 | transmembrane p24 trafficking protein 10 |
| NPM2 | 0.19264559 | nucleophosmin/nucleoplasmin 2 |
| KLHL30 | 0.19282477 | kelch like family member 30 |
| PIGP | 0.193083 | phosphatidylinositol glycan anchor biosynthesis class P |
| SRD5A2 | 0.19314361 | steroid 5 alpha-reductase 2 |
| SLC45A3 | 0.19351779 | solute carrier family 45 member 3 |
| REEP1 | 0.19383663 | receptor accessory protein 1 |
| AOX1 | 0.19386034 | aldehyde oxidase 1 |
| NIPSNAP3A | 0.19454018 | nipsnap homolog 3A |
| SCARA5 | 0.19461924 | scavenger receptor class A member 5 |
| CCHCR1 | 0.19475362 | coiled-coil alpha-helical rod protein 1 |
| SEMA3B | 0.19485903 | semaphorin 3B |
| LRFN4 | 0.19489065 | leucine rich repeat and fibronectin type III domain containing 4 |
| ITGB2 | 0.19489328 | integrin subunit beta 2 |
| LMO7 | 0.19506456 | LIM domain 7 |
| LRIG1 | 0.19586034 | leucine rich repeats and immunoglobulin like domains 1 |
| C8orf82 | 0.19593676 | chromosome 8 open reading frame 82 |
| WDR7 | 0.19628722 | WD repeat domain 7 |
| CCND2 | 0.19682213 | cyclin D2 |
| TUBB6 | 0.19683531 | tubulin beta 6 class V |
| CUL5 | 0.19708037 | cullin 5 |
| USP47 | 0.1973913 | ubiquitin specific peptidase 47 |
| OAF | 0.19750988 | out at first homolog |
| OSMR | 0.19758893 | oncostatin M receptor |
| HAND2-AS1 | 0.19762582 | HAND2 antisense RNA 1 (head to head) |
| NFKB1 | 0.19790514 | nuclear factor kappa B subunit 1 |
| TSG101 | 0.19820026 | tumor susceptibility 101 |
| SMIM14 | 0.19874572 | small integral membrane protein 14 |
| LRRC8A | 0.19879051 | leucine rich repeat containing 8 family member A |
| TMCO3 | 0.19880105 | transmembrane and coiled-coil domains 3 |
| GNA11 | 0.19909881 | G protein subunit alpha 11 |
| CACNA1H | 0.19946245 | calcium voltage-gated channel subunit alpha1 H |
| KLF13 | 0.19959157 | Kruppel like factor 13 |
| DRAM1 | 0.1996469 | DNA damage regulated autophagy modulator 1 |
| PTGR1 | 0.19989196 | prostaglandin reductase 1 |
| RALGPS1 | 0.19998155 | Ral GEF with PH domain and SH3 binding motif 1 |
| KIAA2026 | 0.20062451 | KIAA2026 |
| NUMB | 0.20074572 | NUMB, endocytic adaptor protein |
| HOXB4 | 0.20086957 | homeobox B4 |
| TMEM64 | 0.20178393 | transmembrane protein 64 |
| FAM13A | 0.20183399 | family with sequence similarity 13 member A |
| ARID1A | 0.20199736 | AT-rich interaction domain 1A |
| RNF150 | 0.20206324 | ring finger protein 150 |
| ASB6 | 0.20227141 | ankyrin repeat and SOCS box containing 6 |
| KANK1 | 0.20253228 | KN motif and ankyrin repeat domains 1 |
| PTDSS2 | 0.20282477 | phosphatidylserine synthase 2 |
| CYBRD1 | 0.20289855 | cytochrome b reductase 1 |
| PCCA | 0.20290119 | propionyl-CoA carboxylase alpha subunit |
| CCDC107 | 0.20301186 | coiled-coil domain containing 107 |
| C11orf63 | 0.2031726 | chromosome 11 open reading frame 63 |
| C14orf79 | 0.20374177 | chromosome 14 open reading frame 79 |
| ARNTL | 0.20414229 | aryl hydrocarbon receptor nuclear translocator like |
| C1QA | 0.20444532 | complement C1q A chain |
| ESD | 0.20479315 | esterase D |
| MAFB | 0.2049776 | MAF bZIP transcription factor B |
| PPP3CA | 0.20556522 | protein phosphatase 3 catalytic subunit alpha |
| P3H2 | 0.20588142 | prolyl 3-hydroxylase 2 |
| HSD17B8 | 0.20596838 | hydroxysteroid 17-beta dehydrogenase 8 |
| FAM46B | 0.20612912 | family with sequence similarity 46 member B |
| TMEM245 | 0.20616337 | transmembrane protein 245 |
| PER1 | 0.20633465 | period circadian clock 1 |
| NANOS3 | 0.20679842 | nanos C2HC-type zinc finger 3 |
| LINC00476 | 0.2068643 | long intergenic non-protein coding RNA 476 |
| ENPP2 | 0.20690382 | ectonucleotide pyrophosphatase/phosphodiesterase 2 |
| FBXO8 | 0.20719104 | F-box protein 8 |
| PTPRR | 0.20732543 | protein tyrosine phosphatase, receptor type R |
| LOC286254 | 0.20750198 | uncharacterized LOC286254 |
| BMP5 | 0.20751779 | bone morphogenetic protein 5 |
| EFR3A | 0.20757312 | EFR3 homolog A |
| GTF2H4 | 0.20780764 | general transcription factor IIH subunit 4 |
| STK38 | 0.20802899 | serine/threonine kinase 38 |
| EEPD1 | 0.20828458 | endonuclease/exonuclease/phosphatase family domain containing 1 |
| ZBTB20 | 0.20840053 | zinc finger and BTB domain containing 20 |
| ZBTB20 | 0.20840053 | zinc finger and BTB domain containing 20 |
| PLGRKT | 0.20851647 | plasminogen receptor with a C-terminal lysine |
| NPHS2 | 0.20903557 | NPHS2, podocin |
| FGF9 | 0.20931225 | fibroblast growth factor 9 |
| FGF9 | 0.20931225 | fibroblast growth factor 9 |
| MAP7D1 | 0.20948353 | MAP7 domain containing 1 |
| ZFHX2 | 0.20956785 | zinc finger homeobox 2 |
| NR3C2 | 0.20959157 | nuclear receptor subfamily 3 group C member 2 |
| GPER1 | 0.20962846 | G protein-coupled estrogen receptor 1 |
| CCPG1 | 0.20970751 | cell cycle progression 1 |
| TFB1M | 0.2097365 | transcription factor B1, mitochondrial |
| GLDN | 0.20997892 | gliomedin |
| ANXA5 | 0.20999473 | annexin A5 |
| PCP4 | 0.21035046 | Purkinje cell protein 4 |
| KLF9 | 0.21059552 | Kruppel like factor 9 |
| SNCA | 0.21063241 | synuclein alpha |
| CFAP45 | 0.21063505 | cilia and flagella associated protein 45 |
| KL | 0.21069302 | klotho |
| FNBP1 | 0.21091963 | formin binding protein 1 |
| RBPMS | 0.21104611 | RNA binding protein with multiple splicing |
| CCDC34 | 0.21111462 | coiled-coil domain containing 34 |
| TJP1 | 0.21126219 | tight junction protein 1 |
| OMA1 | 0.21128063 | OMA1 zinc metallopeptidase |
| COMMD9 | 0.21130698 | COMM domain containing 9 |
| AKAP7 | 0.21183663 | A-kinase anchoring protein 7 |
| WEE1 | 0.21196838 | WEE1 G2 checkpoint kinase |
| PDHX | 0.21201581 | pyruvate dehydrogenase complex component X |
| UBL3 | 0.2120975 | ubiquitin like 3 |
| HHLA3 | 0.21258235 | HERV-H LTR-associating 3 |
| NRP2 | 0.21295916 | neuropilin 2 |
| NRP2 | 0.21295916 | neuropilin 2 |
| INPP4B | 0.21304348 | inositol polyphosphate-4-phosphatase type II B |
| SLC46A2 | 0.21316996 | solute carrier family 46 member 2 |
| BTG2 | 0.21366008 | BTG anti-proliferation factor 2 |
| INF2 | 0.21371805 | inverted formin, FH2 and WH2 domain containing |
| BLACAT1 | 0.21386298 | bladder cancer associated transcript 1 (non-protein coding) |
| CLYBL | 0.21398946 | citrate lyase beta like |
| FLRT2 | 0.21415283 | fibronectin leucine rich transmembrane protein 2 |
| ARHGAP1 | 0.21436364 | Rho GTPase activating protein 1 |
| CCDC88A | 0.2150303 | coiled-coil domain containing 88A |
| EGR2 | 0.21538603 | early growth response 2 |
| SERPINB11 | 0.21553096 | serpin family B member 11 (gene/pseudogene) |
| PPP1R14B | 0.21581291 | protein phosphatase 1 regulatory inhibitor subunit 14B |
| PROSC | 0.21662714 | proline synthetase cotranscribed homolog (bacterial) |
| MGST3 | 0.21748353 | microsomal glutathione S-transferase 3 |
| LOC101928433 | 0.21760211 | uncharacterized LOC101928433 |
| IGFBP6 | 0.21775494 | insulin like growth factor binding protein 6 |
| TBC1D4 | 0.2180448 | TBC1 domain family member 4 |
| CASC1 | 0.21880369 | cancer susceptibility candidate 1 |
| DOCK1 | 0.21891963 | dedicator of cytokinesis 1 |
| HLA-DMA | 0.2192253 | major histocompatibility complex, class II, DM alpha |
| HIST1H2BG | 0.21923057 | histone cluster 1, H2bg |
| FERMT2 | 0.21925955 | fermitin family member 2 |
| GBP4 | 0.21940711 | guanylate binding protein 4 |
| MOB3B | 0.21967325 | MOB kinase activator 3B |
| RABEP1 | 0.21972859 | rabaptin, RAB GTPase binding effector protein 1 |
| AKR1B10 | 0.22100395 | aldo-keto reductase family 1 member B10 |
| TIPARP | 0.22104611 | TCDD inducible poly(ADP-ribose) polymerase |
| KIAA1024L | 0.22124901 | KIAA1024 like |
| MAOB | 0.22125692 | monoamine oxidase B |
| UVRAG | 0.22194203 | UV radiation resistance associated |
| FAM210A | 0.22208696 | family with sequence similarity 210 member A |
| ZFHX4 | 0.22295916 | zinc finger homeobox 4 |
| LANCL1 | 0.22303821 | LanC like 1 |
| GARNL3 | 0.22308827 | GTPase activating Rap/RanGAP domain like 3 |
| PLSCR4 | 0.22322003 | phospholipid scramblase 4 |
| ATXN3 | 0.2235415 | ataxin 3 |
| DENND5A | 0.22358366 | DENN domain containing 5A |
| KCTD3 | 0.2241054 | potassium channel tetramerization domain containing 3 |
| CD14 | 0.22452437 | CD14 molecule |
| PCDH7 | 0.22464822 | protocadherin 7 |
| LRRC49 | 0.22488011 | leucine rich repeat containing 49 |
| FRMD4A | 0.22494335 | FERM domain containing 4A |
| FAM46A | 0.2251726 | family with sequence similarity 46 member A |
| TOR1AIP1 | 0.2256917 | torsin 1A interacting protein 1 |
| LGMN | 0.22580237 | legumain |
| C16orf45 | 0.22583663 | chromosome 16 open reading frame 45 |
| ZC2HC1C | 0.22618972 | zinc finger C2HC-type containing 1C |
| CFD | 0.22633202 | complement factor D |
| FAM174A | 0.22635837 | family with sequence similarity 174 member A |
| CYP27A1 | 0.22655599 | cytochrome P450 family 27 subfamily A member 1 |
| GRB10 | 0.22664822 | growth factor receptor bound protein 10 |
| VAV2 | 0.22729644 | vav guanine nucleotide exchange factor 2 |
| DENND3 | 0.22731752 | DENN domain containing 3 |
| VPS13A | 0.22762055 | vacuolar protein sorting 13 homolog A |
| PLEKHA2 | 0.22779183 | pleckstrin homology domain containing A2 |
| BTG3 | 0.22953096 | BTG anti-proliferation factor 3 |
| ARL6IP6 | 0.22955204 | ADP ribosylation factor like GTPase 6 interacting protein 6 |
| CPQ | 0.22963373 | carboxypeptidase Q |
| PER2 | 0.23033729 | period circadian clock 2 |
| BTN3A3 | 0.23045323 | butyrophilin subfamily 3 member A3 |
| TMEM63B | 0.23077734 | transmembrane protein 63B |
| DYNC1I2 | 0.23109618 | dynein cytoplasmic 1 intermediate chain 2 |
| PNMA1 | 0.23155468 | paraneoplastic Ma antigen 1 |
| COG6 | 0.23176812 | component of oligomeric golgi complex 6 |
| TPP1 | 0.2318946 | tripeptidyl peptidase 1 |
| TLR3 | 0.23189987 | toll like receptor 3 |
| ITPR1 | 0.23312253 | inositol 1,4,5-trisphosphate receptor type 1 |
| HACD1 | 0.23362846 | 3-hydroxyacyl-CoA dehydratase 1 |
| SAMD13 | 0.23367062 | sterile alpha motif domain containing 13 |
| GLE1 | 0.23383663 | GLE1, RNA export mediator |
| FAM134B | 0.23402108 | family with sequence similarity 134 member B |
| BNIP3L | 0.23438208 | BCL2 interacting protein 3 like |
| C15orf41 | 0.2345639 | chromosome 15 open reading frame 41 |
| PDLIM4 | 0.23477734 | PDZ and LIM domain 4 |
| RRN3P1 | 0.23517523 | RRN3 homolog, RNA polymerase I transcription factor pseudogene 1 |
| KLF4 | 0.23588142 | Kruppel like factor 4 |
| CD82 | 0.23613702 | CD82 molecule |
| LINC01278 | 0.23617128 | long intergenic non-protein coding RNA 1278 |
| SEPW1 | 0.23669302 | selenoprotein W, 1 |
| EPS8L1 | 0.23696179 | EPS8 like 1 |
| NEK1 | 0.23772069 | NIMA related kinase 1 |
| SETX | 0.23828458 | senataxin |
| WBP4 | 0.23834519 | WW domain binding protein 4 |
| DIXDC1 | 0.23863768 | DIX domain containing 1 |
| PIBF1 | 0.23914625 | progesterone immunomodulatory binding factor 1 |
| NIPAL3 | 0.23933333 | NIPA like domain containing 3 |
| MTURN | 0.23953887 | maturin, neural progenitor differentiation regulator homolog (Xenopus) |
| HLA-H | 0.23994203 | major histocompatibility complex, class I, H (pseudogene) |
| SH3BGRL | 0.2399473 | SH3 domain binding glutamate rich protein like |
| TXNRD1 | 0.2399473 | thioredoxin reductase 1 |
| GSTA4 | 0.24007905 | glutathione S-transferase alpha 4 |
| NMRK1 | 0.2401502 | nicotinamide riboside kinase 1 |
| SRPK3 | 0.24020817 | SRSF protein kinase 3 |
| DIS3L2 | 0.24041107 | DIS3 like 3'-5' exoribonuclease 2 |
| IFNGR1 | 0.24050593 | interferon gamma receptor 1 |
| IRAK3 | 0.24053491 | interleukin 1 receptor associated kinase 3 |
| IRAK3 | 0.24053491 | interleukin 1 receptor associated kinase 3 |
| LYZ | 0.24169697 | lysozyme |
| TDRP | 0.24172596 | testis development related protein |
| UCHL3 | 0.24266403 | ubiquitin C-terminal hydrolase L3 |
| ENDOV | 0.24298814 | endonuclease V |
| TMEM126B | 0.24380501 | transmembrane protein 126B |
| SEPP1 | 0.24407115 | selenoprotein P, plasma, 1 |
| ANTXR2 | 0.24433729 | anthrax toxin receptor 2 |
| DGAT2 | 0.24476943 | diacylglycerol O-acyltransferase 2 |
| KLHL13 | 0.24500659 | kelch like family member 13 |
| CCL3 | 0.2451278 | C-C motif chemokine ligand 3 |
| CDKN2B | 0.24515942 | cyclin dependent kinase inhibitor 2B |
| AHNAK | 0.24540711 | AHNAK nucleoprotein |
| TWIST1 | 0.24563636 | twist family bHLH transcription factor 1 |
| TANK | 0.2460527 | TRAF family member associated NFKB activator |
| ZNF165 | 0.24640843 | zinc finger protein 165 |
| ALOX5AP | 0.24647431 | arachidonate 5-lipoxygenase activating protein |
| PHOSPHO2 | 0.24655072 | phosphatase, orphan 2 |
| TRIP12 | 0.24665349 | thyroid hormone receptor interactor 12 |
| CASQ2 | 0.24693017 | calsequestrin 2 |
| MICU2 | 0.24700132 | mitochondrial calcium uptake 2 |
| TMEM167B | 0.24714361 | transmembrane protein 167B |
| CACHD1 | 0.24746509 | cache domain containing 1 |
| AHNAK2 | 0.2475863 | AHNAK nucleoprotein 2 |
| CAP2 | 0.24770751 | CAP, adenylate cyclase-associated protein, 2 (yeast) |
| GCLC | 0.24824769 | glutamate-cysteine ligase catalytic subunit |
| NLGN1 | 0.24878524 | neuroligin 1 |
| TNFRSF25 | 0.25155731 | TNF receptor superfamily member 25 |
| GBP1 | 0.25171278 | guanylate binding protein 1 |
| ZC3H12B | 0.25172596 | zinc finger CCCH-type containing 12B |
| HABP4 | 0.25282213 | hyaluronan binding protein 4 |
| SNORD55 | 0.25331225 | small nucleolar RNA, C/D box 55 |
| AAED1 | 0.25339394 | AhpC/TSA antioxidant enzyme domain containing 1 |
| MAF | 0.25369697 | MAF bZIP transcription factor |
| ILK | 0.25432411 | integrin linked kinase |
| CDC37L1 | 0.25488538 | cell division cycle 37 like 1 |
| KCTD9 | 0.25557049 | potassium channel tetramerization domain containing 9 |
| RHBDL3 | 0.25565217 | rhomboid like 3 |
| CD47 | 0.25630567 | CD47 molecule |
| HLA-DRB3 | 0.25639789 | major histocompatibility complex, class II, DR beta 3 |
| IRF1 | 0.25652964 | interferon regulatory factor 1 |
| KLHDC1 | 0.25677734 | kelch domain containing 1 |
| C1QC | 0.25699868 | complement C1q C chain |
| INPP1 | 0.25703294 | inositol polyphosphate-1-phosphatase |
| TMEM246 | 0.25729117 | transmembrane protein 246 |
| ID2 | 0.25880369 | inhibitor of DNA binding 2, HLH protein |
| SIK1 | 0.25910145 | salt inducible kinase 1 |
| RAB31 | 0.25919895 | RAB31, member RAS oncogene family |
| CDC42EP2 | 0.25982345 | CDC42 effector protein 2 |
| ST6GALNAC6 | 0.25986034 | ST6 N-acetylgalactosaminide alpha-2,6-sialyltransferase 6 |
| AASS | 0.26080896 | aminoadipate-semialdehyde synthase |
| KLF12 | 0.26172332 | Kruppel like factor 12 |
| CLN5 | 0.26179974 | ceroid-lipofuscinosis, neuronal 5 |
| SRGN | 0.26180237 | serglycin |
| CPNE8 | 0.26251647 | copine 8 |
| PEX11G | 0.2628195 | peroxisomal biogenesis factor 11 gamma |
| SGPP1 | 0.26432411 | sphingosine-1-phosphate phosphatase 1 |
| PNMA2 | 0.26575231 | paraneoplastic Ma antigen 2 |
| ZNF438 | 0.26581818 | zinc finger protein 438 |
| NBEA | 0.26610277 | neurobeachin |
| DQX1 | 0.26632675 | DEAQ-box RNA dependent ATPase 1 |
| GJC2 | 0.26705929 | gap junction protein gamma 2 |
| SLFN5 | 0.26707773 | schlafen family member 5 |
| GLRX | 0.26716206 | glutaredoxin |
| SIK3 | 0.2679552 | SIK family kinase 3 |
| DPYD | 0.26799473 | dihydropyrimidine dehydrogenase |
| LMTK3 | 0.26982872 | lemur tyrosine kinase 3 |
| MTHFSD | 0.26998682 | methenyltetrahydrofolate synthetase domain containing |
| RAI2 | 0.27016601 | retinoic acid induced 2 |
| ATL1 | 0.27021607 | atlastin GTPase 1 |
| PTGER4 | 0.27022398 | prostaglandin E receptor 4 |
| HIST1H2AE | 0.27151515 | histone cluster 1, H2ae |
| ZSCAN18 | 0.2723004 | zinc finger and SCAN domain containing 18 |
| TMEM241 | 0.27235573 | transmembrane protein 241 |
| CBX7 | 0.27292227 | chromobox 7 |
| ATG13 | 0.27364163 | autophagy related 13 |
| SMAD7 | 0.27366008 | SMAD family member 7 |
| JPH1 | 0.27411594 | junctophilin 1 |
| PELI2 | 0.27437154 | pellino E3 ubiquitin protein ligase family member 2 |
| ADGRE5 | 0.27470092 | adhesion G protein-coupled receptor E5 |
| LITAF | 0.27498287 | lipopolysaccharide induced TNF factor |
| SERGEF | 0.27515152 | secretion regulating guanine nucleotide exchange factor |
| LDOC1 | 0.27531752 | leucine zipper down-regulated in cancer 1 |
| MTAP | 0.27573386 | methylthioadenosine phosphorylase |
| LATS2 | 0.27586034 | large tumor suppressor kinase 2 |
| CAT | 0.27710672 | catalase |
| ANKRD50 | 0.2774888 | ankyrin repeat domain 50 |
| DLX3 | 0.27766008 | distal-less homeobox 3 |
| HOXB6 | 0.27777339 | homeobox B6 |
| SHROOM3 | 0.27784717 | shroom family member 3 |
| HLA-DPA1 | 0.27842161 | major histocompatibility complex, class II, DP alpha 1 |
| STEAP1 | 0.27859552 | six transmembrane epithelial antigen of the prostate 1 |
| CASP1 | 0.27947036 | caspase 1 |
| VWA5A | 0.28002635 | von Willebrand factor A domain containing 5A |
| TANC1 | 0.2805639 | tetratricopeptide repeat, ankyrin repeat and coiled-coil containing 1 |
| OXCT1 | 0.28085112 | 3-oxoacid CoA-transferase 1 |
| NFIB | 0.28101449 | nuclear factor I B |
| NFIB | 0.28101449 | nuclear factor I B |
| ZNF432 | 0.28218182 | zinc finger protein 432 |
| MEIS2 | 0.2822108 | Meis homeobox 2 |
| TMEM41B | 0.28496706 | transmembrane protein 41B |
| TOR4A | 0.28566798 | torsin family 4 member A |
| ELF1 | 0.28567062 | E74 like ETS transcription factor 1 |
| GNG10 | 0.2857971 | G protein subunit gamma 10 |
| GNG10 | 0.2857971 | G protein subunit gamma 10 |
| GADD45A | 0.28601054 | growth arrest and DNA damage inducible alpha |
| PGM2L1 | 0.28612121 | phosphoglucomutase 2 like 1 |
| COLCA1 | 0.28725955 | colorectal cancer associated 1 |
| PHLDB2 | 0.2873834 | pleckstrin homology like domain family B member 2 |
| RSPH1 | 0.28782345 | radial spoke head 1 homolog |
| SLC39A14 | 0.28805007 | solute carrier family 39 member 14 |
| ADH1C | 0.28892754 | alcohol dehydrogenase 1C (class I), gamma polypeptide |
| MATN2 | 0.28893808 | matrilin 2 |
| CD163 | 0.29081159 | CD163 molecule |
| PRAC1 | 0.29095916 | prostate cancer susceptibility candidate 1 |
| PPP1R36 | 0.29098024 | protein phosphatase 1 regulatory subunit 36 |
| CHST9 | 0.29269302 | carbohydrate sulfotransferase 9 |
| HBEGF | 0.29322266 | heparin binding EGF like growth factor |
| FPR3 | 0.29361528 | formyl peptide receptor 3 |
| CAV1 | 0.29470883 | caveolin 1 |
| TPST1 | 0.29475626 | tyrosylprotein sulfotransferase 1 |
| ADAMTSL5 | 0.29513043 | ADAMTS like 5 |
| GAS8 | 0.29579447 | growth arrest specific 8 |
| METTL15 | 0.29727273 | methyltransferase like 15 |
| TLE4 | 0.29807905 | transducin like enhancer of split 4 |
| HIST2H2AA3 | 0.29808696 | histone cluster 2, H2aa3 |
| CRYZ | 0.29837418 | crystallin zeta |
| BTBD10 | 0.29899605 | BTB domain containing 10 |
| DLG2 | 0.29925165 | discs large MAGUK scaffold protein 2 |
| RRAS | 0.299278 | related RAS viral (r-ras) oncogene homolog |
| ZCCHC14 | 0.29963109 | zinc finger CCHC-type containing 14 |
| CLIP4 | 0.29964163 | CAP-Gly domain containing linker protein family member 4 |
| NMU | 0.30050593 | neuromedin U |
| EEF1A2 | 0.30152569 | eukaryotic translation elongation factor 1 alpha 2 |
| TES | 0.302 | testin LIM domain protein |
| HIBCH | 0.30262714 | 3-hydroxyisobutyryl-CoA hydrolase |
| IL6 | 0.30412912 | interleukin 6 |
| HLA-DQB1 | 0.30537549 | major histocompatibility complex, class II, DQ beta 1 |
| SPTBN5 | 0.30569433 | spectrin beta, non-erythrocytic 5 |
| BOK | 0.30618709 | BOK, BCL2 family apoptosis regulator |
| CKB | 0.30773386 | creatine kinase B |
| MAN1A1 | 0.30874308 | mannosidase alpha class 1A member 1 |
| APIP | 0.31020553 | APAF1 interacting protein |
| SYK | 0.31048221 | spleen associated tyrosine kinase |
| UBE2E2 | 0.31071146 | ubiquitin conjugating enzyme E2 E2 |
| SMPDL3A | 0.31173386 | sphingomyelin phosphodiesterase acid like 3A |
| SELENBP1 | 0.31210013 | selenium binding protein 1 |
| TNFSF13B | 0.31215547 | tumor necrosis factor superfamily member 13b |
| ENPP4 | 0.31243742 | ectonucleotide pyrophosphatase/phosphodiesterase 4 (putative) |
| EPDR1 | 0.31306192 | ependymin related 1 |
| GADD45B | 0.31325428 | growth arrest and DNA damage inducible beta |
| TYROBP | 0.31378129 | TYRO protein tyrosine kinase binding protein |
| SERPINB1 | 0.31401845 | serpin family B member 1 |
| CLK1 | 0.3160527 | CDC like kinase 1 |
| JAK2 | 0.31617918 | Janus kinase 2 |
| ETS2 | 0.31685903 | ETS proto-oncogene 2, transcription factor |
| FBXO3 | 0.31714097 | F-box protein 3 |
| SORBS2 | 0.31751252 | sorbin and SH3 domain containing 2 |
| WLS | 0.31760474 | wntless Wnt ligand secretion mediator |
| HOXA2 | 0.31772596 | homeobox A2 |
| ZNF671 | 0.31831884 | zinc finger protein 671 |
| IFNE | 0.31955204 | interferon epsilon |
| OAT | 0.32100132 | ornithine aminotransferase |
| TACC1 | 0.32151252 | transforming acidic coiled-coil containing protein 1 |
| MYOF | 0.32338076 | myoferlin |
| SDCBP | 0.32396047 | syndecan binding protein |
| ZBED5 | 0.3263531 | zinc finger BED-type containing 5 |
| EPHX2 | 0.32782872 | epoxide hydrolase 2 |
| CNTRL | 0.32821871 | centriolin |
| MSRB2 | 0.32884058 | methionine sulfoxide reductase B2 |
| HLA-F | 0.32895652 | major histocompatibility complex, class I, F |
| SPATS2L | 0.3297365 | spermatogenesis associated serine rich 2 like |
| ARL14EP | 0.3299552 | ADP ribosylation factor like GTPase 14 effector protein |
| C5 | 0.33012648 | complement component 5 |
| TLR5 | 0.33066667 | toll like receptor 5 |
| ADAMTS1 | 0.33107773 | ADAM metallopeptidase with thrombospondin type 1 motif 1 |
| NME5 | 0.33149934 | NME/NM23 family member 5 |
| ZNF10 | 0.33171278 | zinc finger protein 10 |
| PLK3 | 0.33248748 | polo like kinase 3 |
| LPXN | 0.33280105 | leupaxin |
| CYSTM1 | 0.33282477 | cysteine rich transmembrane module containing 1 |
| RABGAP1 | 0.33542819 | RAB GTPase activating protein 1 |
| SPIRE1 | 0.33648748 | spire type actin nucleation factor 1 |
| THBS1 | 0.33694071 | thrombospondin 1 |
| CXCL2 | 0.33724638 | C-X-C motif chemokine ligand 2 |
| PRRG1 | 0.33747563 | proline rich and Gla domain 1 |
| KBTBD11 | 0.33904875 | kelch repeat and BTB domain containing 11 |
| PTBP2 | 0.34007905 | polypyrimidine tract binding protein 2 |
| PALM | 0.34269302 | paralemmin |
| CFAP70 | 0.3428643 | cilia and flagella associated protein 70 |
| CENPBD1 | 0.34320685 | CENPB DNA-binding domain containing 1 |
| FAM149A | 0.34350461 | family with sequence similarity 149 member A |
| CSRNP1 | 0.34581818 | cysteine and serine rich nuclear protein 1 |
| SKAP2 | 0.34684321 | src kinase associated phosphoprotein 2 |
| NACC2 | 0.34766535 | NACC family member 2 |
| PAM | 0.34792095 | peptidylglycine alpha-amidating monooxygenase |
| JUN | 0.34837681 | Jun proto-oncogene, AP-1 transcription factor subunit |
| FGL2 | 0.34985507 | fibrinogen like 2 |
| WDR19 | 0.35147563 | WD repeat domain 19 |
| IDNK | 0.35199473 | IDNK, gluconokinase |
| AGR3 | 0.35271937 | anterior gradient 3, protein disulphide isomerase family member |
| MMP28 | 0.35610804 | matrix metallopeptidase 28 |
| MSANTD4 | 0.35621344 | Myb/SANT DNA binding domain containing 4 with coiled-coils |
| SPTSSB | 0.35811594 | serine palmitoyltransferase small subunit B |
| SPTSSB | 0.35811594 | serine palmitoyltransferase small subunit B |
| C1QB | 0.35951515 | complement C1q B chain |
| NCOA7 | 0.35995257 | nuclear receptor coactivator 7 |
| NUPR1 | 0.36183663 | nuclear protein 1, transcriptional regulator |
| STC1 | 0.36213702 | stanniocalcin 1 |
| PDE7B | 0.36230303 | phosphodiesterase 7B |
| ARHGEF37 | 0.36309354 | Rho guanine nucleotide exchange factor 37 |
| IL33 | 0.36320949 | interleukin 33 |
| GLS | 0.36505138 | glutaminase |
| NAPRT | 0.36594466 | nicotinate phosphoribosyltransferase |
| CAV2 | 0.36669829 | caveolin 2 |
| HLA-DRB4 | 0.36813175 | major histocompatibility complex, class II, DR beta 4 |
| HLA-DRB4 | 0.36813175 | major histocompatibility complex, class II, DR beta 4 |
| MMP1 | 0.36909091 | matrix metallopeptidase 1 |
| ACADM | 0.37216864 | acyl-CoA dehydrogenase, C-4 to C-12 straight chain |
| CD74 | 0.37635573 | CD74 molecule |
| AHI1 | 0.3765639 | Abelson helper integration site 1 |
| MXRA5 | 0.3790303 | matrix remodeling associated 5 |
| CYR61 | 0.38001845 | cysteine rich angiogenic inducer 61 |
| STEAP2 | 0.38020553 | STEAP2 metalloreductase |
| ALCAM | 0.38062714 | activated leukocyte cell adhesion molecule |
| SBSPON | 0.38154941 | somatomedin B and thrombospondin type 1 domain containing |
| EXD3 | 0.3819473 | exonuclease 3'-5' domain containing 3 |
| ALDH1A1 | 0.38279051 | aldehyde dehydrogenase 1 family member A1 |
| MPPED2 | 0.38580237 | metallophosphoesterase domain containing 2 |
| COBL | 0.38960474 | cordon-bleu WH2 repeat protein |
| ITGAV | 0.38979183 | integrin subunit alpha V |
| SHTN1 | 0.39244796 | shootin 1 |
| SLC38A5 | 0.39810277 | solute carrier family 38 member 5 |
| SPOCK1 | 0.39864295 | sparc/osteonectin, cwcv and kazal-like domains proteoglycan (testican) 1 |
| POU5F1 | 0.3995863 | POU class 5 homeobox 1 |
| F2RL1 | 0.40113043 | F2R like trypsin receptor 1 |
| SYBU | 0.40250329 | syntabulin |
| HLA-B | 0.40268775 | major histocompatibility complex, class I, B |
| MYOM2 | 0.40293808 | myomesin 2 |
| PPP1R15A | 0.40394993 | protein phosphatase 1 regulatory subunit 15A |
| FAM214B | 0.40816074 | family with sequence similarity 214 member B |
| ATXN1 | 0.41323847 | ataxin 1 |
| C11orf70 | 0.41361528 | chromosome 11 open reading frame 70 |
| TNFAIP3 | 0.41517787 | TNF alpha induced protein 3 |
| AR | 0.41711199 | androgen receptor |
| AR | 0.41711199 | androgen receptor |
| SAMD9L | 0.41762055 | sterile alpha motif domain containing 9 like |
| RASD1 | 0.41782872 | ras related dexamethasone induced 1 |
| ASS1 | 0.41796574 | argininosuccinate synthase 1 |
| SPATA18 | 0.41877997 | spermatogenesis associated 18 |
| CD44 | 0.42442424 | CD44 molecule (Indian blood group) |
| SNX19 | 0.42528063 | sorting nexin 19 |
| MYC | 0.43741502 | v-myc avian myelocytomatosis viral oncogene homolog |
| GPAT3 | 0.43922266 | glycerol-3-phosphate acyltransferase 3 |
| EXT1 | 0.44274308 | exostosin glycosyltransferase 1 |
| IRS1 | 0.44387088 | insulin receptor substrate 1 |
| HLA-DRA | 0.44565217 | major histocompatibility complex, class II, DR alpha |
| N4BP2L2 | 0.44645323 | NEDD4 binding protein 2 like 2 |
| N4BP2L2 | 0.44645323 | NEDD4 binding protein 2 like 2 |
| PRKAG2 | 0.45054545 | protein kinase AMP-activated non-catalytic subunit gamma 2 |
| NR4A2 | 0.45333333 | nuclear receptor subfamily 4 group A member 2 |
| UBD | 0.45449539 | ubiquitin D |
| CFAP69 | 0.45726746 | cilia and flagella associated protein 69 |
| RND3 | 0.4616917 | Rho family GTPase 3 |
| ALDH2 | 0.46193149 | aldehyde dehydrogenase 2 family (mitochondrial) |
| STX11 | 0.46222398 | syntaxin 11 |
| SPAG1 | 0.46604743 | sperm associated antigen 1 |
| CCL20 | 0.47095125 | C-C motif chemokine ligand 20 |
| CD55 | 0.47244005 | CD55 molecule (Cromer blood group) |
| TRIM31 | 0.47819763 | tripartite motif containing 31 |
| SCN11A | 0.48704875 | sodium voltage-gated channel alpha subunit 11 |
| SLC25A23 | 0.49886166 | solute carrier family 25 member 23 |
| HOXB3 | 0.49896179 | homeobox B3 |
| MROH7 | 0.50055336 | maestro heat like repeat family member 7 |
| PLXDC2 | 0.50420026 | plexin domain containing 2 |
| PLXDC2 | 0.50420026 | plexin domain containing 2 |
| RGS1 | 0.50930171 | regulator of G-protein signaling 1 |
| PNPLA7 | 0.51164954 | patatin like phospholipase domain containing 7 |
| TCEA3 | 0.51631357 | transcription elongation factor A3 |
| LAMA3 | 0.5245191 | laminin subunit alpha 3 |
| EGR1 | 0.5290224 | early growth response 1 |
| LRRC6 | 0.53505402 | leucine rich repeat containing 6 |
| HOXB9 | 0.53866667 | homeobox B9 |
| CDK6 | 0.54134651 | cyclin dependent kinase 6 |
| RARRES3 | 0.54322793 | retinoic acid receptor responder 3 |
| SNRPN | 0.54856917 | small nuclear ribonucleoprotein polypeptide N |
| SNRPN | 0.54856917 | small nuclear ribonucleoprotein polypeptide N |
| TRIM22 | 0.5530224 | tripartite motif containing 22 |
| SERPINB5 | 0.56035837 | serpin family B member 5 |
| RDX | 0.5622635 | radixin |
| HIST1H2BD | 0.56368643 | histone cluster 1, H2bd |
| ARL14 | 0.5672859 | ADP ribosylation factor like GTPase 14 |
| CD59 | 0.57104611 | CD59 molecule |
| FOS | 0.5729697 | Fos proto-oncogene, AP-1 transcription factor subunit |
| NCS1 | 0.59515942 | neuronal calcium sensor 1 |
| IGFBP5 | 0.60732806 | insulin like growth factor binding protein 5 |
| CDK14 | 0.62001845 | cyclin dependent kinase 14 |
| HIST1H3I///HIST1H3E | 0.6206166 | histone cluster 1, H3i///histone cluster 1, H3e |
| PLA2G4A | 0.63245586 | phospholipase A2 group IVA |
| PPP2R2B | 0.63517523 | protein phosphatase 2 regulatory subunit Bbeta |
| CLIC6 | 0.63868248 | chloride intracellular channel 6 |
| DUSP2 | 0.6565191 | dual specificity phosphatase 2 |
| DKK1 | 0.69817391 | dickkopf WNT signaling pathway inhibitor 1 |
| ADHFE1 | 0.72716996 | alcohol dehydrogenase, iron containing 1 |
| DUSP1 | 0.73759157 | dual specificity phosphatase 1 |
| SPP1 | 0.78485639 | secreted phosphoprotein 1 |
| FHL2 | 0.78949144 | four and a half LIM domains 2 |
| PTGS2 | 0.88829776 | prostaglandin-endoperoxide synthase 2 |
| FOSB | 0.92949934 | FosB proto-oncogene, AP-1 transcription factor subunit |
| CLCA4 | 1.0651805 | chloride channel accessory 4 |
| FABP4 | 1.33005007 | fatty acid binding protein 4 |
| TSPAN8 | 1.40788669 | tetraspanin 8 |

**Table S32.** The list of up-regulated genes in PBC-RNIT

| ene symbol | logFC | Gene title |
| --- | --- | --- |
| MYH11 | -3.18 | myosin heavy chain 11 |
| MFAP4 | -3.06 | microfibrillar associated protein 4 |
| ACTG2 | -3 | actin, gamma 2, smooth muscle, enteric |
| CNN1 | -2.94 | calponin 1 |
| FLNC | -2.92 | filamin C |
| ACTC1 | -2.68 | actin, alpha, cardiac muscle 1 |
| DES | -2.66 | desmin |
| SRPX | -2.5 | sushi repeat containing protein, X-linked |
| PRAC1 | -2.39 | prostate cancer susceptibility candidate 1 |
| PTGS1 | -2.35 | prostaglandin-endoperoxide synthase 1 |
| CFD | -2.34 | complement factor D |
| LUM | -2.34 | lumican |
| PGM5 | -2.33 | phosphoglucomutase 5 |
| PCP4 | -2.32 | Purkinje cell protein 4 |
| FHL1 | -2.31 | four and a half LIM domains 1 |
| HSPB6 | -2.31 | heat shock protein family B (small) member 6 |
| SPARCL1 | -2.31 | SPARC like 1 |
| SPON1 | -2.29 | spondin 1 |
| MRGPRF | -2.25 | MAS related GPR family member F |
| JCHAIN | -2.24 | joining chain of multimeric IgA and IgM |
| DCN | -2.17 | decorin |
| COL16A1 | -2.15 | collagen type XVI alpha 1 chain |
| SMOC2 | -2.15 | SPARC related modular calcium binding 2 |
| PDLIM3 | -2.14 | PDZ and LIM domain 3 |
| FAM107A | -2.13 | family with sequence similarity 107 member A |
| FAM107A | -2.13 | family with sequence similarity 107 member A |
| FOSB | -2.13 | FosB proto-oncogene, AP-1 transcription factor subunit |
| PRUNE2 | -2.13 | prune homolog 2 |
| CALD1 | -2.05 | caldesmon 1 |
| SYNM | -2.05 | synemin |
| RGS1 | -2.02 | regulator of G-protein signaling 1 |
| CTGF | -2.01 | connective tissue growth factor |
| FOXF1 | -2.01 | forkhead box F1 |
| CRYAB | -1.99 | crystallin alpha B |
| ACTA2 | -1.98 | actin, alpha 2, smooth muscle, aorta |
| C2orf40 | -1.97 | chromosome 2 open reading frame 40 |
| TAGLN | -1.96 | transgelin |
| KCNMB1 | -1.95 | potassium calcium-activated channel subfamily M regulatory beta subunit 1 |
| TMEM119 | -1.94 | transmembrane protein 119 |
| CCND2 | -1.93 | cyclin D2 |
| CPED1 | -1.91 | cadherin like and PC-esterase domain containing 1 |
| P2RX1 | -1.9 | purinergic receptor P2X 1 |
| PALLD | -1.9 | palladin, cytoskeletal associated protein |
| PTGIS | -1.9 | prostaglandin I2 (prostacyclin) synthase |
| RBPMS2 | -1.89 | RNA binding protein with multiple splicing 2 |
| PLAC9 | -1.88 | placenta specific 9 |
| TCEAL2 | -1.86 | transcription elongation factor A like 2 |
| PARM1 | -1.85 | prostate androgen-regulated mucin-like protein 1 |
| SORBS1 | -1.84 | sorbin and SH3 domain containing 1 |
| CASQ2 | -1.83 | calsequestrin 2 |
| MOXD1 | -1.82 | monooxygenase DBH like 1 |
| PDGFRA | -1.81 | platelet derived growth factor receptor alpha |
| ABCA8 | -1.8 | ATP binding cassette subfamily A member 8 |
| ALDH1A1 | -1.8 | aldehyde dehydrogenase 1 family member A1 |
| BIN1 | -1.8 | bridging integrator 1 |
| COX7A1 | -1.8 | cytochrome c oxidase subunit 7A1 |
| RGS2 | -1.8 | regulator of G-protein signaling 2 |
| DUSP1 | -1.78 | dual specificity phosphatase 1 |
| IGFBP5 | -1.78 | insulin like growth factor binding protein 5 |
| ADAMTS8 | -1.77 | ADAM metallopeptidase with thrombospondin type 1 motif 8 |
| SDPR | -1.76 | serum deprivation response |
| ATP1A2 | -1.75 | ATPase Na+/K+ transporting subunit alpha 2 |
| EGR1 | -1.75 | early growth response 1 |
| AEBP1 | -1.74 | AE binding protein 1 |
| C7 | -1.74 | complement component 7 |
| CDH11 | -1.74 | cadherin 11 |
| LAMC3 | -1.74 | laminin subunit gamma 3 |
| SEPP1 | -1.74 | selenoprotein P, plasma, 1 |
| CLIP3 | -1.72 | CAP-Gly domain containing linker protein 3 |
| COL6A1 | -1.72 | collagen type VI alpha 1 chain |
| HLA-DPA1 | -1.72 | major histocompatibility complex, class II, DP alpha 1 |
| RASL12 | -1.72 | RAS like family 12 |
| SELM | -1.72 | selenoprotein M |
| TPM1 | -1.72 | tropomyosin 1 (alpha) |
| EGR2 | -1.71 | early growth response 2 |
| JAM3 | -1.71 | junctional adhesion molecule 3 |
| ADH1A | -1.7 | alcohol dehydrogenase 1A (class I), alpha polypeptide |
| FOS | -1.7 | Fos proto-oncogene, AP-1 transcription factor subunit |
| ADAMTS1 | -1.69 | ADAM metallopeptidase with thrombospondin type 1 motif 1 |
| LMOD1 | -1.69 | leiomodin 1 |
| MGP | -1.69 | matrix Gla protein |
| NDNF | -1.69 | neuron derived neurotrophic factor |
| CYP1B1 | -1.68 | cytochrome P450 family 1 subfamily B member 1 |
| SH3GL2 | -1.68 | SH3 domain containing GRB2 like 2, endophilin A1 |
| MAMDC2 | -1.67 | MAM domain containing 2 |
| TPM2 | -1.67 | tropomyosin 2 (beta) |
| ALDH1A3 | -1.66 | aldehyde dehydrogenase 1 family member A3 |
| DPYSL3 | -1.66 | dihydropyrimidinase like 3 |
| C8orf4 | -1.64 | chromosome 8 open reading frame 4 |
| CLCA4 | -1.64 | chloride channel accessory 4 |
| CYBRD1 | -1.64 | cytochrome b reductase 1 |
| MSRB3 | -1.64 | methionine sulfoxide reductase B3 |
| FABP4 | -1.63 | fatty acid binding protein 4 |
| CRISPLD2 | -1.62 | cysteine rich secretory protein LCCL domain containing 2 |
| HLA-DRA | -1.62 | major histocompatibility complex, class II, DR alpha |
| COL1A2 | -1.61 | collagen type I alpha 2 chain |
| MYL9 | -1.61 | myosin light chain 9 |
| FGL2 | -1.6 | fibrinogen like 2 |
| TCF21 | -1.6 | transcription factor 21 |
| ABI3BP | -1.59 | ABI family member 3 binding protein |
| ANTXR2 | -1.59 | anthrax toxin receptor 2 |
| PDK4 | -1.59 | pyruvate dehydrogenase kinase 4 |
| TNC | -1.59 | tenascin C |
| FXYD6 | -1.58 | FXYD domain containing ion transport regulator 6 |
| ITGA8 | -1.58 | integrin subunit alpha 8 |
| MMP7 | -1.58 | matrix metallopeptidase 7 |
| VIM | -1.58 | vimentin |
| DIXDC1 | -1.56 | DIX domain containing 1 |
| FGF9 | -1.56 | fibroblast growth factor 9 |
| FGF9 | -1.56 | fibroblast growth factor 9 |
| TMOD1 | -1.56 | tropomodulin 1 |
| ISL1 | -1.55 | ISL LIM homeobox 1 |
| JAML | -1.55 | junction adhesion molecule like |
| MYOM1 | -1.55 | myomesin 1 |
| RERGL | -1.55 | RERG like |
| PMP22 | -1.54 | peripheral myelin protein 22 |
| PIGR | -1.53 | polymeric immunoglobulin receptor |
| SGCE | -1.53 | sarcoglycan epsilon |
| WNT5A | -1.53 | Wnt family member 5A |
| HSD17B6 | -1.52 | hydroxysteroid 17-beta dehydrogenase 6 |
| KLF9 | -1.52 | Kruppel like factor 9 |
| DKK1 | -1.51 | dickkopf WNT signaling pathway inhibitor 1 |
| DPT | -1.51 | dermatopontin |
| ACOX2 | -1.5 | acyl-CoA oxidase 2 |
| COL6A2 | -1.5 | collagen type VI alpha 2 chain |
| FILIP1L | -1.5 | filamin A interacting protein 1 like |
| RNF150 | -1.5 | ring finger protein 150 |
| SBSPON | -1.5 | somatomedin B and thrombospondin type 1 domain containing |
| SCARA5 | -1.5 | scavenger receptor class A member 5 |
| C1S | -1.49 | complement component 1, s subcomponent |
| DACT3 | -1.49 | dishevelled binding antagonist of beta catenin 3 |
| EMILIN1 | -1.49 | elastin microfibril interfacer 1 |
| PI16 | -1.49 | peptidase inhibitor 16 |
| PTRF | -1.49 | polymerase I and transcript release factor |
| STON1 | -1.48 | stonin 1 |
| KRT13 | -1.47 | keratin 13 |
| TGFB3 | -1.47 | transforming growth factor beta 3 |
| HLA-DRB4 | -1.46 | major histocompatibility complex, class II, DR beta 4 |
| HLA-DRB4 | -1.46 | major histocompatibility complex, class II, DR beta 4 |
| ALDH2 | -1.45 | aldehyde dehydrogenase 2 family (mitochondrial) |
| EMP3 | -1.45 | epithelial membrane protein 3 |
| FAM129A | -1.45 | family with sequence similarity 129 member A |
| SFRP2 | -1.45 | secreted frizzled related protein 2 |
| ROR2 | -1.44 | receptor tyrosine kinase like orphan receptor 2 |
| HSPB8 | -1.43 | heat shock protein family B (small) member 8 |
| PLA2G4C | -1.43 | phospholipase A2 group IVC |
| THBS2 | -1.43 | thrombospondin 2 |
| MEG3 | -1.42 | maternally expressed 3 (non-protein coding) |
| CNRIP1 | -1.41 | cannabinoid receptor interacting protein 1 |
| GATM | -1.41 | glycine amidinotransferase |
| RGS5 | -1.41 | regulator of G-protein signaling 5 |
| ALOX5AP | -1.4 | arachidonate 5-lipoxygenase activating protein |
| CYR61 | -1.4 | cysteine rich angiogenic inducer 61 |
| CCL19 | -1.39 | C-C motif chemokine ligand 19 |
| FERMT2 | -1.39 | fermitin family member 2 |
| GAS6 | -1.39 | growth arrest specific 6 |
| CPXM2 | -1.38 | carboxypeptidase X, M14 family member 2 |
| EVA1C | -1.38 | eva-1 homolog C |
| GNG11 | -1.38 | G protein subunit gamma 11 |
| PTGDS | -1.38 | prostaglandin D2 synthase |
| A2M | -1.37 | alpha-2-macroglobulin |
| COL6A3 | -1.37 | collagen type VI alpha 3 chain |
| IL6ST | -1.37 | interleukin 6 signal transducer |
| IL6ST | -1.37 | interleukin 6 signal transducer |
| ITM2A | -1.37 | integral membrane protein 2A |
| SERPINE2 | -1.37 | serpin family E member 2 |
| CLEC3B | -1.36 | C-type lectin domain family 3 member B |
| TSHZ3 | -1.36 | teashirt zinc finger homeobox 3 |
| ZAK | -1.36 | sterile alpha motif and leucine zipper containing kinase AZK |
| ZEB2 | -1.36 | zinc finger E-box binding homeobox 2 |
| ZEB2 | -1.36 | zinc finger E-box binding homeobox 2 |
| C11orf96 | -1.35 | chromosome 11 open reading frame 96 |
| SYNPO2 | -1.35 | synaptopodin 2 |
| WFDC1 | -1.35 | WAP four-disulfide core domain 1 |
| ACKR1 | -1.34 | atypical chemokine receptor 1 (Duffy blood group) |
| LHFP | -1.34 | lipoma HMGIC fusion partner |
| OLFML3 | -1.34 | olfactomedin like 3 |
| SRGN | -1.34 | serglycin |
| GLIPR2 | -1.33 | GLI pathogenesis related 2 |
| GPR183 | -1.33 | G protein-coupled receptor 183 |
| LGALS4 | -1.33 | galectin 4 |
| LTBP4 | -1.33 | latent transforming growth factor beta binding protein 4 |
| CYP27A1 | -1.32 | cytochrome P450 family 27 subfamily A member 1 |
| EDNRA | -1.32 | endothelin receptor type A |
| ENPP2 | -1.32 | ectonucleotide pyrophosphatase/phosphodiesterase 2 |
| OSBPL10 | -1.32 | oxysterol binding protein like 10 |
| SERPINF1 | -1.32 | serpin family F member 1 |
| SLIT2 | -1.32 | slit guidance ligand 2 |
| GNG10 | -1.31 | G protein subunit gamma 10 |
| GNG10 | -1.31 | G protein subunit gamma 10 |
| GYPC | -1.31 | glycophorin C (Gerbich blood group) |
| CAV1 | -1.3 | caveolin 1 |
| FNBP1 | -1.3 | formin binding protein 1 |
| LOC100507073 | -1.3 | uncharacterized LOC100507073 |
| LPP | -1.3 | LIM domain containing preferred translocation partner in lipoma |
| LPP | -1.3 | LIM domain containing preferred translocation partner in lipoma |
| PROM1 | -1.3 | prominin 1 |
| UPK3A | -1.3 | uroplakin 3A |
| ZBTB16 | -1.3 | zinc finger and BTB domain containing 16 |
| COLEC12 | -1.29 | collectin subfamily member 12 |
| RARRES2 | -1.29 | retinoic acid receptor responder 2 |
| CCL2 | -1.28 | C-C motif chemokine ligand 2 |
| CXCL12 | -1.28 | C-X-C motif chemokine ligand 12 |
| DPYSL2 | -1.28 | dihydropyrimidinase like 2 |
| FLNA | -1.28 | filamin A |
| LGALS3 | -1.28 | lectin, galactoside binding soluble 3 |
| LGALS3 | -1.28 | lectin, galactoside binding soluble 3 |
| ADGRA2 | -1.27 | adhesion G protein-coupled receptor A2 |
| CLIC6 | -1.27 | chloride intracellular channel 6 |
| KANK2 | -1.27 | KN motif and ankyrin repeat domains 2 |
| NTRK3 | -1.27 | neurotrophic receptor tyrosine kinase 3 |
| NTRK3 | -1.27 | neurotrophic receptor tyrosine kinase 3 |
| PAMR1 | -1.27 | peptidase domain containing associated with muscle regeneration 1 |
| PLPPR4 | -1.27 | phospholipid phosphatase related 4 |
| SERPINA3 | -1.27 | serpin family A member 3 |
| SPARC | -1.27 | secreted protein acidic and cysteine rich |
| GFRA1 | -1.26 | GDNF family receptor alpha 1 |
| IRF8 | -1.26 | interferon regulatory factor 8 |
| NR2F1 | -1.26 | nuclear receptor subfamily 2 group F member 1 |
| PELI2 | -1.26 | pellino E3 ubiquitin protein ligase family member 2 |
| SH3BGRL | -1.26 | SH3 domain binding glutamate rich protein like |
| SMTN | -1.26 | smoothelin |
| ACACB | -1.25 | acetyl-CoA carboxylase beta |
| AXIN2 | -1.25 | axin 2 |
| COL5A1 | -1.25 | collagen type V alpha 1 chain |
| BOC | -1.24 | BOC cell adhesion associated, oncogene regulated |
| DOCK2 | -1.24 | dedicator of cytokinesis 2 |
| HLA-DRB3 | -1.24 | major histocompatibility complex, class II, DR beta 3 |
| PTGS2 | -1.24 | prostaglandin-endoperoxide synthase 2 |
| TIMP2 | -1.24 | TIMP metallopeptidase inhibitor 2 |
| TNNT3 | -1.24 | troponin T3, fast skeletal type |
| EPB41L3 | -1.23 | erythrocyte membrane protein band 4.1 like 3 |
| ITGA5 | -1.23 | integrin subunit alpha 5 |
| PRICKLE2 | -1.23 | prickle planar cell polarity protein 2 |
| PRRT2 | -1.23 | proline rich transmembrane protein 2 |
| SPEG | -1.23 | SPEG complex locus |
| APCDD1 | -1.22 | APC down-regulated 1 |
| ZFP36 | -1.22 | ZFP36 ring finger protein |
| ANGPTL2 | -1.21 | angiopoietin like 2 |
| FCER1A | -1.21 | Fc fragment of IgE receptor Ia |
| PLAT | -1.21 | plasminogen activator, tissue type |
| SORBS2 | -1.21 | sorbin and SH3 domain containing 2 |
| CX3CL1 | -1.2 | C-X3-C motif chemokine ligand 1 |
| GHR | -1.2 | growth hormone receptor |
| OLFM1 | -1.2 | olfactomedin 1 |
| TGFBI | -1.2 | transforming growth factor beta induced |
| ACAP1 | -1.19 | ArfGAP with coiled-coil, ankyrin repeat and PH domains 1 |
| ANXA1 | -1.19 | annexin A1 |
| CACNA1H | -1.19 | calcium voltage-gated channel subunit alpha1 H |
| EPDR1 | -1.19 | ependymin related 1 |
| NBEA | -1.19 | neurobeachin |
| NDN | -1.19 | necdin, MAGE family member |
| PAM | -1.19 | peptidylglycine alpha-amidating monooxygenase |
| PRICKLE1 | -1.19 | prickle planar cell polarity protein 1 |
| RASD1 | -1.19 | ras related dexamethasone induced 1 |
| RERG | -1.19 | RAS like estrogen regulated growth inhibitor |
| TBC1D10C | -1.19 | TBC1 domain family member 10C |
| CSRP1 | -1.18 | cysteine and glycine rich protein 1 |
| APOD | -1.17 | apolipoprotein D |
| C1QA | -1.17 | complement C1q A chain |
| CD2 | -1.17 | CD2 molecule |
| CHRM3 | -1.17 | cholinergic receptor muscarinic 3 |
| CHRM3 | -1.17 | cholinergic receptor muscarinic 3 |
| MAOB | -1.17 | monoamine oxidase B |
| PDGFD | -1.17 | platelet derived growth factor D |
| SGCA | -1.17 | sarcoglycan alpha |
| TGFBR2 | -1.17 | transforming growth factor beta receptor 2 |
| AGR3 | -1.16 | anterior gradient 3, protein disulphide isomerase family member |
| GLT8D2 | -1.16 | glycosyltransferase 8 domain containing 2 |
| GNG7 | -1.16 | G protein subunit gamma 7 |
| KLF2 | -1.16 | Kruppel like factor 2 |
| HDC | -1.15 | histidine decarboxylase |
| JAZF1 | -1.15 | JAZF zinc finger 1 |
| SCUBE2 | -1.15 | signal peptide, CUB domain and EGF like domain containing 2 |
| CD6 | -1.14 | CD6 molecule |
| CKB | -1.14 | creatine kinase B |
| IL7R | -1.14 | interleukin 7 receptor |
| RBP1 | -1.14 | retinol binding protein 1 |
| DDR2 | -1.13 | discoidin domain receptor tyrosine kinase 2 |
| MXRA5 | -1.13 | matrix remodeling associated 5 |
| PDE7B | -1.13 | phosphodiesterase 7B |
| PRKCB | -1.13 | protein kinase C beta |
| CGNL1 | -1.12 | cingulin like 1 |
| HOXA13 | -1.12 | homeobox A13 |
| LINC01451 | -1.12 | long intergenic non-protein coding RNA 1451 |
| PLA2G4A | -1.12 | phospholipase A2 group IVA |
| PPP1R14A | -1.12 | protein phosphatase 1 regulatory inhibitor subunit 14A |
| RNASE4 | -1.12 | ribonuclease A family member 4 |
| SPRR3 | -1.12 | small proline rich protein 3 |
| ZNF521 | -1.12 | zinc finger protein 521 |
| CD48 | -1.11 | CD48 molecule |
| GATA5 | -1.11 | GATA binding protein 5 |
| GSN | -1.11 | gelsolin |
| IGFBP2 | -1.11 | insulin like growth factor binding protein 2 |
| IGFBP6 | -1.11 | insulin like growth factor binding protein 6 |
| NFIB | -1.11 | nuclear factor I B |
| NFIB | -1.11 | nuclear factor I B |
| GAS1 | -1.1 | growth arrest specific 1 |
| PCOLCE2 | -1.1 | procollagen C-endopeptidase enhancer 2 |
| SLC9A9 | -1.1 | solute carrier family 9 member A9 |
| SNRPN | -1.1 | small nuclear ribonucleoprotein polypeptide N |
| SNRPN | -1.1 | small nuclear ribonucleoprotein polypeptide N |
| SOBP | -1.1 | sine oculis binding protein homolog |
| SOBP | -1.1 | sine oculis binding protein homolog |
| ZCCHC24 | -1.1 | zinc finger CCHC-type containing 24 |
| ADTRP | -1.09 | androgen dependent TFPI regulating protein |
| PTGR1 | -1.09 | prostaglandin reductase 1 |
| SCN11A | -1.09 | sodium voltage-gated channel alpha subunit 11 |
| TGFB1I1 | -1.09 | transforming growth factor beta 1 induced transcript 1 |
| UPK1A | -1.09 | uroplakin 1A |
| COL15A1 | -1.08 | collagen type XV alpha 1 chain |
| HAND2-AS1 | -1.08 | HAND2 antisense RNA 1 (head to head) |
| PDE4D | -1.08 | phosphodiesterase 4D |
| PDGFC | -1.08 | platelet derived growth factor C |
| PRDM8 | -1.08 | PR/SET domain 8 |
| SFRP1 | -1.08 | secreted frizzled related protein 1 |
| TRIM22 | -1.08 | tripartite motif containing 22 |
| LIMS2 | -1.07 | LIM zinc finger domain containing 2 |
| MAPRE2 | -1.07 | microtubule associated protein RP/EB family member 2 |
| MS4A6A | -1.07 | membrane spanning 4-domains A6A |
| NEXN | -1.07 | nexilin F-actin binding protein |
| RGS11 | -1.07 | regulator of G-protein signaling 11 |
| STAB1 | -1.07 | stabilin 1 |
| TUBB6 | -1.07 | tubulin beta 6 class V |
| COLCA1 | -1.06 | colorectal cancer associated 1 |
| GPNMB | -1.06 | glycoprotein nmb |
| TSC22D1 | -1.06 | TSC22 domain family member 1 |
| AXL | -1.05 | AXL receptor tyrosine kinase |
| CSF1R | -1.05 | colony stimulating factor 1 receptor |
| CTSK | -1.05 | cathepsin K |
| DEFB1 | -1.05 | defensin beta 1 |
| B2M | -1.04 | beta-2-microglobulin |
| BMP5 | -1.04 | bone morphogenetic protein 5 |
| CAP2 | -1.04 | CAP, adenylate cyclase-associated protein, 2 (yeast) |
| DACT1 | -1.04 | dishevelled binding antagonist of beta catenin 1 |
| EOMES | -1.04 | eomesodermin |
| REEP1 | -1.04 | receptor accessory protein 1 |
| STOM | -1.04 | stomatin |
| AQP1 | -1.03 | aquaporin 1 (Colton blood group) |
| BNC2 | -1.03 | basonuclin 2 |
| COL3A1 | -1.03 | collagen type III alpha 1 chain |
| DOCK11 | -1.03 | dedicator of cytokinesis 11 |
| IDH1 | -1.03 | isocitrate dehydrogenase (NADP(+)) 1, cytosolic |
| MATN2 | -1.03 | matrilin 2 |
| MFAP5 | -1.03 | microfibrillar associated protein 5 |
| NFIX | -1.03 | nuclear factor I X |
| RARRES3 | -1.03 | retinoic acid receptor responder 3 |
| RASAL3 | -1.03 | RAS protein activator like 3 |
| STXBP6 | -1.03 | syntaxin binding protein 6 |
| VIPR2 | -1.03 | vasoactive intestinal peptide receptor 2 |
| CAV2 | -1.02 | caveolin 2 |
| GPX3 | -1.02 | glutathione peroxidase 3 |
| PRKCDBP | -1.02 | protein kinase C delta binding protein |
| SLMAP | -1.02 | sarcolemma associated protein |
| AP1S2 | -1.01 | adaptor related protein complex 1 sigma 2 subunit |
| ARHGAP9 | -1.01 | Rho GTPase activating protein 9 |
| FAM162B | -1.01 | family with sequence similarity 162 member B |
| HLA-DMB | -1.01 | major histocompatibility complex, class II, DM beta |
| LITAF | -1.01 | lipopolysaccharide induced TNF factor |
| PEG3 | -1.01 | paternally expressed 3 |
| PLEK | -1.01 | pleckstrin |
| PODN | -1.01 | podocan |
| RAMP1 | -1.01 | receptor activity modifying protein 1 |
| TNFAIP8L3 | -1.01 | TNF alpha induced protein 8 like 3 |
| AFF3 | -1 | AF4/FMR2 family member 3 |
| HACD1 | -1 | 3-hydroxyacyl-CoA dehydratase 1 |
| PLSCR4 | -1 | phospholipid scramblase 4 |
| TSPYL1 | -1 | TSPY like 1 |
| DENND2A | -0.999 | DENN domain containing 2A |
| WLS | -0.999 | wntless Wnt ligand secretion mediator |
| CPE | -0.998 | carboxypeptidase E |
| DKK3 | -0.997 | dickkopf WNT signaling pathway inhibitor 3 |
| WIPF1 | -0.995 | WAS/WASL interacting protein family member 1 |
| CPVL | -0.993 | carboxypeptidase, vitellogenic like |
| ITGB2 | -0.992 | integrin subunit beta 2 |
| RGL1 | -0.992 | ral guanine nucleotide dissociation stimulator like 1 |
| BTG2 | -0.991 | BTG anti-proliferation factor 2 |
| PAPPA | -0.989 | pappalysin 1 |
| HOXA9 | -0.986 | homeobox A9 |
| BTF3 | -0.984 | basic transcription factor 3 |
| SETBP1 | -0.984 | SET binding protein 1 |
| TYROBP | -0.982 | TYRO protein tyrosine kinase binding protein |
| LGALS1 | -0.981 | galectin 1 |
| TCF4 | -0.981 | transcription factor 4 |
| FAM43A | -0.98 | family with sequence similarity 43 member A |
| CTSG | -0.979 | cathepsin G |
| NFIA | -0.979 | nuclear factor I A |
| NFIA | -0.979 | nuclear factor I A |
| GPAT3 | -0.978 | glycerol-3-phosphate acyltransferase 3 |
| GSTM5 | -0.976 | glutathione S-transferase mu 5 |
| FILIP1 | -0.974 | filamin A interacting protein 1 |
| OLFML1 | -0.973 | olfactomedin like 1 |
| MAP1B | -0.971 | microtubule associated protein 1B |
| HLA-F | -0.97 | major histocompatibility complex, class I, F |
| ESD | -0.969 | esterase D |
| CCL5 | -0.968 | C-C motif chemokine ligand 5 |
| SEMA3A | -0.968 | semaphorin 3A |
| CLIC4 | -0.967 | chloride intracellular channel 4 |
| SGK1 | -0.967 | serum/glucocorticoid regulated kinase 1 |
| NLGN1 | -0.963 | neuroligin 1 |
| RNF145 | -0.963 | ring finger protein 145 |
| PLS3 | -0.962 | plastin 3 |
| COL21A1 | -0.961 | collagen type XXI alpha 1 chain |
| FZD7 | -0.959 | frizzled class receptor 7 |
| ITGAL | -0.959 | integrin subunit alpha L |
| RPS4X | -0.959 | ribosomal protein S4, X-linked |
| GJA1 | -0.958 | gap junction protein alpha 1 |
| SPTSSB | -0.957 | serine palmitoyltransferase small subunit B |
| SPTSSB | -0.957 | serine palmitoyltransferase small subunit B |
| TRIM31 | -0.955 | tripartite motif containing 31 |
| GFPT2 | -0.954 | glutamine-fructose-6-phosphate transaminase 2 |
| LMO3 | -0.954 | LIM domain only 3 |
| P3H2 | -0.951 | prolyl 3-hydroxylase 2 |
| POPDC2 | -0.951 | popeye domain containing 2 |
| AP3S1 | -0.95 | adaptor related protein complex 3 sigma 1 subunit |
| NUPR1 | -0.949 | nuclear protein 1, transcriptional regulator |
| PLPP3 | -0.946 | phospholipid phosphatase 3 |
| BHMT2 | -0.943 | betaine--homocysteine S-methyltransferase 2 |
| WEE1 | -0.943 | WEE1 G2 checkpoint kinase |
| COL13A1 | -0.942 | collagen type XIII alpha 1 chain |
| MGLL | -0.941 | monoglyceride lipase |
| TSC22D3 | -0.941 | TSC22 domain family member 3 |
| ACAT1 | -0.94 | acetyl-CoA acetyltransferase 1 |
| PKDCC | -0.94 | protein kinase domain containing, cytoplasmic |
| C1QB | -0.939 | complement C1q B chain |
| LRCH2 | -0.938 | leucine rich repeats and calponin homology domain containing 2 |
| COL4A5 | -0.937 | collagen type IV alpha 5 chain |
| C3 | -0.936 | complement component 3 |
| C3 | -0.936 | complement component 3 |
| ADH1C | -0.935 | alcohol dehydrogenase 1C (class I), gamma polypeptide |
| JAM2 | -0.935 | junctional adhesion molecule 2 |
| C3orf70 | -0.932 | chromosome 3 open reading frame 70 |
| PNPLA7 | -0.932 | patatin like phospholipase domain containing 7 |
| GVINP1 | -0.931 | GTPase, very large interferon inducible pseudogene 1 |
| FBLN2 | -0.928 | fibulin 2 |
| IGFBP7 | -0.928 | insulin like growth factor binding protein 7 |
| SLC2A3 | -0.927 | solute carrier family 2 member 3 |
| DLG2 | -0.925 | discs large MAGUK scaffold protein 2 |
| CD37 | -0.923 | CD37 molecule |
| ITK | -0.919 | IL2 inducible T-cell kinase |
| TSPAN8 | -0.919 | tetraspanin 8 |
| ALDH1A2 | -0.918 | aldehyde dehydrogenase 1 family member A2 |
| NR4A2 | -0.918 | nuclear receptor subfamily 4 group A member 2 |
| PTH1R | -0.918 | parathyroid hormone 1 receptor |
| AHNAK2 | -0.917 | AHNAK nucleoprotein 2 |
| CD8A | -0.915 | CD8a molecule |
| ITPR1 | -0.913 | inositol 1,4,5-trisphosphate receptor type 1 |
| ASPA | -0.912 | aspartoacylase |
[truncated: 572,953 more chars]
